# Health inequities and clustering of fever, acute respiratory infection, diarrhoea and wasting in children under five in low- and middle-income countries: a Demographic and Health Surveys analysis

**DOI:** 10.1186/s12916-021-02018-0

**Published:** 2021-06-24

**Authors:** Peter Winskill, Alexandra B. Hogan, Julie Thwing, Lazaro Mwandigha, Patrick G. T. Walker, Ben Lambert

**Affiliations:** 1 MRC Centre for Global Infectious Disease Analysis, Imperial College London, London, UK; 2 Malaria Branch, US Centers for Disease Control and Prevention, Atlanta, GA USA; 3 Nuffield Department of Primary Care Health Science, University of Oxford, Oxford, UK

**Keywords:** Malaria, Pneumonia, Diarrhoea, Malnutrition, Clustering, Child health, Integrated community case management

## Abstract

**Background:**

Pneumonia, diarrhoea and malaria are responsible for over one third of all deaths in children under the age of 5 years in low and middle sociodemographic index countries; many of these deaths are also associated with malnutrition. We explore the co-occurrence and clustering of fever, acute respiratory infection, diarrhoea and wasting and their relationship with equity-relevant variables.

**Methods:**

Multilevel, multivariate Bayesian logistic regression models were fitted to Demographic and Health Survey data from over 380,000 children in 39 countries. The relationship between outcome indicators (fever, acute respiratory infection, diarrhoea and wasting) and equity-relevant variables (wealth, access to health care and rurality) was examined. We quantified the geographical clustering and co-occurrence of conditions and a child’s risk of multiple illnesses.

**Results:**

The prevalence of outcomes was very heterogeneous within and between countries. There was marked spatial clustering of conditions and co-occurrence within children. For children in the poorest households and those reporting difficulties accessing healthcare, there were significant increases in the probability of at least one of the conditions in 18 of 21 countries, with estimated increases in the probability of up to 0.23 (95% CrI, 0.06–0.40).

**Conclusions:**

The prevalence of fever, acute respiratory infection, diarrhoea and wasting are associated with equity-relevant variables and cluster together. Via pathways of shared aetiology or risk, those children most disadvantaged disproportionately suffer from these conditions. This highlights the need for horizontal approaches, such as integrated community case management, with a focus on equity and targeted to those most at need.

**Supplementary Information:**

The online version contains supplementary material available at 10.1186/s12916-021-02018-0.

## Background

Over a third of all deaths in children under 5 years old in low and middle sociodemographic index countries are either from lower respiratory infections (including pneumonia) (estimated as 16% of deaths in under 5 s), diarrhoea (11%) or malaria (7%) [1]. Furthermore, around 45% of childhood deaths are associated with undernutrition [2]. In the era of universal health coverage (UHC), many of these deaths could and should be prevented by prompt diagnosis and treatment. Whilst accurate diagnosis and treatment is complicated by multiple aetiologies, there are effective treatments available: antibiotics for pneumonia, oral rehydration therapy for diarrhoea and antimalarials for malaria.

The landscape of child health in these countries is strongly shaped by inequities in risk and access to quality timely healthcare. These inequities often compound: those most at risk are also those who have greatest difficulty accessing timely, high-quality healthcare [3]. Socioeconomic inequalities lead to differing survival rates in children [4], and poverty is known to be associated with fever [5], malaria [6], pneumonia [7], diarrhoea [8] and malnutrition [9]. There is evidence that, presumably due to shared aetiologies or risk factors, these illnesses often co-occur (e.g. [10]) and that inequities in treatment access [11] are also associated with risk and negative health outcomes.

Implementing healthcare packages that account for co-occurrence of illnesses and these sources of inequity is therefore imperative [12], and equity-focussed efforts are being championed by the WHO and UNICEF: including a policy recommendation for integrated community case management (iCCM) [13] where community health workers (CHWs) are trained to diagnose and treat malaria, pneumonia and diarrhoea, or refer cases if severe and also to recognise malnutrition. Vaccination can also target inequality [14], and GAVI-supported distribution of vaccines against rotavirus, pneumococcal disease and Haemophilus influenzae type b (Hib) is bolstering coverage in the poorest countries.

To ensure that horizontal approaches to child health — ones that address a wide range of health issues, such as iCCM — have maximum impact, it is important to understand how co-occurrence, inequality and common risks influence child health in low-resource settings. Demographic and Health Surveys (DHS) are a set of nationally representative household surveys providing linked measures of equality and health outcomes across many countries [15]. Within the standardised questionnaire that surveys mothers about their children under five, there are questions about whether fever, acute respiratory infection (ARI) or diarrhoea occurred in the past 2 weeks [15] and a question aimed at measuring malnutrition (moderate or severe wasting). These outcomes are closely related to the major childhood causes of mortality (malaria, diarrhoea, pneumonia, malnutrition), although there is considerable overlap in the symptoms of specific illnesses. Our study leverages these large, standardised DHS datasets to determine co-occurrence and clustering of fever, diarrhoea, ARI and wasting. It also probes the relationship between their co-occurrence, equity-relevant variables and risk across 39 low- and middle-income countries (LMICs). The analysis explores how these relationships vary between and within countries and across conditions.

## Methods

Summary estimates of prevalence and the occurrence of multiple conditions were calculated from the survey data. Raw estimates were adjusted to account for DHS survey design. We fitted a multilevel, multivariate (four outcome) Bayesian logistic regression to explore the relationship between fever, ARI, diarrhoea and wasting and key equity-related variables of interest, adjusted for many known confounding variables and risk factors. Models were fitted separately for each country since the volume of data in each country meant a pooled analysis fitted in a hierarchical framework across all countries would not meaningfully differ. In the case of India, the survey was so large that, for computational reasons, fitting was performed at the first administrative unit (state) level. We selected DHS surveys with data collected after and including 2010, in countries currently classified as LMICs by The World Bank (N = 39, Additional file 1).

### Response variables

Four response variables of interest were extracted from the DHS datasets. These were (1) fever, (2) ARI, (3) diarrhoea and (4) wasting. These measures are recorded from questions to the child’s mother regarding fever, ARI (fast, short, rapid breaths or difficulty breathing) or diarrhoea and a measure of wasting (moderately or severely wasted: number of children whose weight-for-height z-score is below minus 2 (− 2.0) standard deviations (SD) below the mean on the WHO Child Growth Standards) [15]. All measures were binary, indicating whether a child under the age of five had exhibited symptoms of each condition during the 2 weeks preceding the survey (fever, ARI and diarrhoea) or whether they were wasted. Throughout, we follow DHS convention and refer to the “prevalence” of each condition as the proportion of children exhibiting symptoms during this 2-week window or, in the case of malnutrition, being scored as moderately or severely wasted at the time of the survey.

### Covariates

The model for each country included a set of covariates which can be split into two groups: “covariates of interest” for this analysis (which were equity-focussed) and control covariates shared for all responses (“shared covariates”). Shared covariates were selected on the basis of being known risk factors [16–21] that were available in the DHS data. DHS survey-cluster (where there were typically 25–30 households per cluster) information was included as a group-level (random intercept) covariate.

Covariates of interest were equity-relevant variables: (i) DHS wealth index quintiles (poorest, poor, middle, wealthy and wealthiest), which is a composite measure of a household’s living standard and is estimated on a country-specific basis, accounting for urban/rural differences, and (ii) whether the mother of children reported that distance and/or cost led to serious problems accessing health care when she was ill, which we consider a direct proxy for their child’s healthcare access. Access was coded as a problem if the mother indicated that either distance or cost led to serious problems accessing health care, and (iii) location (urban or rural), which is determined on a country-specific basis and is known to be a complex composite determinant of health [22].

Shared covariates included in the model were (i) child’s age (years), (ii) mother’s education (no education, primary, secondary or higher), (iii) birthweight (below average, average or above average), (iv) mother’s age at birth (10–19, 20–29, 30–39, 40–49, 50–59, 60+ years), (v) sex (female/male), (vi) month of survey, (vii) whether the child slept under a bed net the previous night (not available/collected for all countries, 7 missing), (viii) type of toilet (unimproved or improved), (ix) type of water source (unimproved or improved), (x) cooking fuel used (solid, clean or none) and (xi) proportion of children fully vaccinated (with fully vaccinated defined as those with record of 1 BCG, 3 DTP, 3 Polio and 1 measles vaccination and summarised at the first administrative unit level). For each survey, we included all children aged between 0.5 and 5 years and dropped any covariates with 10% or more missing observations and then selected all children with complete response and covariate data.

### Model formulation

Presence or absence of a given condition was encoded by a binary response variable y. For each condition, h, considered, we modelled the outcome for individual i in cluster j as,

1 $$ {\mathrm{y}}_{\mathrm{hij}}\sim \mathrm{Bernoulli}\left({\mathrm{P}}_{\mathrm{hij}}\right). $$

The logit link function is used to associate probability (P_hij_) with the linear predictor η_hij_,

2 $$ \log \left(\frac{{\mathrm{P}}_{\mathrm{hij}}}{1-{\mathrm{P}}_{\mathrm{hij}}}\right)={\upeta}_{\mathrm{hij}} $$

3 $$ {\upeta}_{\mathrm{hij}}={\upbeta}_{0\mathrm{h}}+{\upmu}_{\mathrm{hj}}+{\sum}_{\mathrm{k}=1}^{\mathrm{q}}{\upbeta}_{\mathrm{k}\mathrm{h}}{\mathrm{x}}_{\mathrm{k}\mathrm{h}\mathrm{ij}}, $$

where β_0h_ is the fixed intercept for response h and μ_hj_ is the group-level intercept for response h in cluster j. For q covariates, β_kh_ an effect size (slope) for covariate k, condition h and x_khij_ is a covariate corresponding to the kth effect size (slope) in the model for condition h in individual i in cluster j. Group-level coefficients are assumed to be correlated between responses, y, such that

4 $$ {\upmu}_{\mathrm{hj}}\sim MVN\left(0,{\Omega}_G\right), $$

where *MVN* is a multivariate normal distribution, with covariance matrix Ω_*G*_ (For further details, see Additional file 2). Model convergence and goodness of fit tests were performed for all models (Additional file 2).

### Predicted difference in the probability of having a condition

To assess the impact of equity-relevant variables in model fits, we estimated the difference in posterior predicted probabilities of a child having a condition between the poorest and wealthiest households, between those with and without difficulties accessing the nearest health facility and between those living in rural and urban locations. Posterior predictions from the model are made based on fitted predictions across posterior samples keeping all covariates apart from the covariate of interest fixed at their median value. We identified trends with strong evidence as those where the probability that there was no difference was less than 1%.

### Clustering

Spatial clustering of conditions at the survey cluster level was quantified by estimating Moran’s I statistic — a measure of how similar estimates close to each other are — from the raw survey data of each condition within countries using the ape package in R [23]. We also estimated the expected probability of having more than one condition under the assumption that conditions were independent (Additional file 2).

## Results

**Fig. 1:**
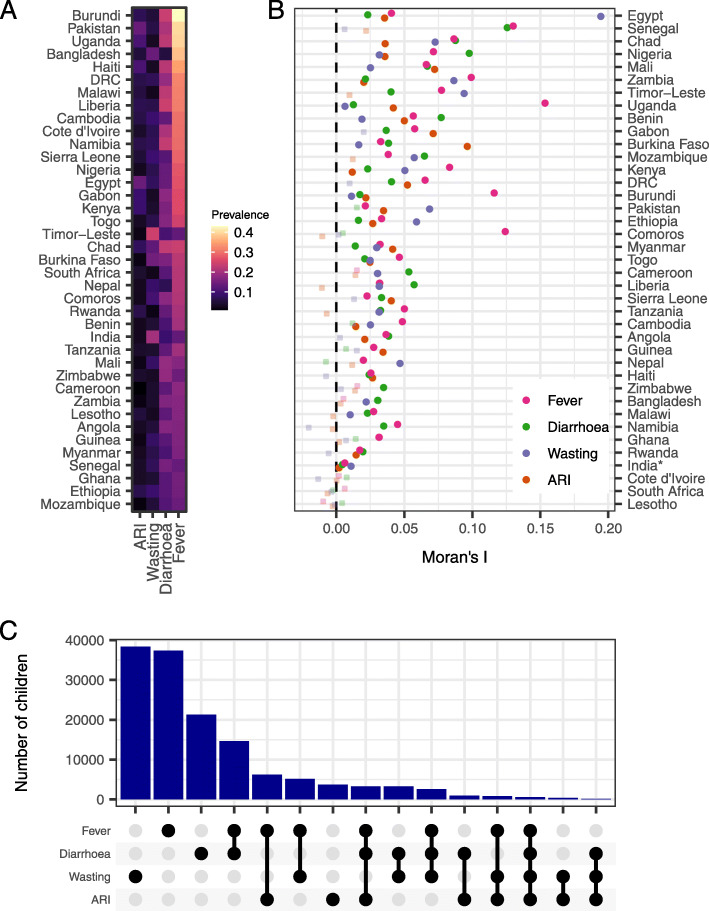
Prevalence, spatial clustering and co-occurrence of conditions. **A** Average prevalence of ARI, wasting, diarrhoea and fever across all studied countries. **B** Estimated spatial clustering as determined by Moran’s I calculated using unadjusted survey data (solid points indicate statistical difference from zero at 5% level; translucent points indicate no statistical difference). **C** Frequency of individual conditions and combinations of conditions in children

A total of 380,357 individual child records from 39 countries were included in the analysis. This sample included 70,625 instances of fever (18.8% prevalence), 46,655 instances of diarrhoea (12.5%) and 16,029 instances of ARI (4.3%) in the 2 weeks preceding the survey and 51,238 instances of wasting (13.5%) (Table 1, Fig. 1A). There was substantial variation in country-level estimates of the prevalence of conditions and the percentage of children reporting multiple conditions (Table 1, Fig. 1A). Conditions were clustered spatially: the majority (> 70%) of estimated Moran’s I values were significantly greater than one, indicating strong spatial clustering (spatial autocorrelation) of conditions across survey-clusters within countries (Fig. 1B). Co-occurrence of conditions was extremely common. The most common pairs of conditions were fever + diarrhoea, fever + ARI and fever + wasting (Fig. 1C), all of which were more frequently observed than ARI alone.

**Table 1:** Summary of survey data. Country-level survey estimates of the prevalence of each condition and the percentage of children reporting N conditions. Here, ranges show 95% binomial confidence intervals (95% CIs)

Country	N	Prevalence (%, 95% CIs)				N conditions (%, 95% CIs)			
		Fever	ARI	Diarrhoea	Wasting	1	2	3	4
Angola	5150	16.6 (15.2, 17.9)	3.4 (2.8, 4.1)	17.0 (15.5, 18.4)	5.2 (4.4, 6.0)	21.9 (20.3, 23.4)	8.0 (7.0, 8.9)	1.4 (0.9, 1.8)	0.0 (0.0, 0.1)
Bangladesh	5900	37.7 (36.1, 39.4)	5.3 (4.5, 6.1)	5.6 (4.7, 6.6)	14.0 (12.9, 15.2)	36.3 (34.7, 38.0)	10.7 (9.6, 11.8)	1.5 (1.2, 1.9)	0.1 (0.0, 0.2)
Benin	10,099	20.1 (19.3, 20.9)	2.9 (2.5, 3.2)	10.9 (10.2, 11.5)	4.8 (4.3, 5.2)	22.4 (21.5, 23.2)	6.2 (5.7, 6.7)	1.1 (0.9, 1.4)	0.1 (0.0, 0.2)
Burkina Faso	5401	22.3 (21.1, 23.5)	1.9 (1.5, 2.3)	16.2 (15.1, 17.2)	14.8 (13.8, 15.8)	26.7 (25.5, 28.0)	10.6 (9.7, 11.5)	2.2 (1.8, 2.6)	0.1 (0.0, 0.3)
Burundi	5316	43.1 (41.7, 44.6)	7.3 (6.6, 8.1)	23.9 (22.7, 25.2)	5.2 (4.5, 5.8)	35.6 (34.1, 37.0)	16.3 (15.2, 17.4)	3.5 (2.9, 4.0)	0.2 (0.1, 0.4)
Cambodia	3850	29.9 (28.1, 31.8)	6.2 (5.3, 7.2)	13.4 (12.0, 14.8)	9.3 (8.2, 10.5)	27.2 (25.4, 29.0)	11.3 (10.0, 12.6)	2.8 (2.1, 3.5)	0.2 (0.0, 0.4)
Cameroon	3672	17.9 (16.5, 19.4)	1.0 (0.7, 1.4)	12.9 (11.6, 14.2)	4.0 (3.3, 4.8)	22.9 (21.3, 24.5)	5.7 (4.8, 6.6)	0.5 (0.3, 0.8)	0.0 (0.0, 0.0)
Chad	8460	24.0 (22.9, 25.2)	7.9 (7.2, 8.7)	23.2 (22.0, 24.4)	13.0 (12.1, 13.8)	25.5 (24.3, 26.6)	13.4 (12.4, 14.4)	4.6 (4.0, 5.2)	0.5 (0.4, 0.7)
Comoros	1869	21.4 (19.1, 23.7)	2.7 (1.9, 3.6)	17.6 (15.5, 19.8)	10.3 (8.7, 11.9)	27.9 (25.4, 30.4)	9.2 (7.6, 10.8)	1.9 (1.1, 2.6)	0.1 (0.0, 0.2)
Cote d’Ivoire	2550	29.8 (27.4, 32.1)	3.9 (3.0, 4.9)	20.9 (18.9, 22.9)	7.1 (5.8, 8.4)	25.6 (23.4, 27.7)	13.7 (11.9, 15.5)	2.7 (1.9, 3.6)	0.1 (0.0, 0.2)
DRC	6209	31.9 (30.3, 33.5)	6.6 (5.8, 7.5)	18.6 (17.3, 20.0)	7.5 (6.6, 8.4)	32.0 (30.4, 33.6)	12.7 (11.6, 13.9)	2.3 (1.8, 2.7)	0.1 (0.0, 0.2)
Egypt	11,706	27.0 (26.0, 27.9)	14.2 (13.5, 15.0)	14.0 (13.3, 14.8)	8.0 (7.5, 8.6)	25.0 (24.0, 25.9)	13.3 (12.5, 14.0)	3.7 (3.3, 4.1)	0.2 (0.1, 0.3)
Ethiopia	7523	15.0 (13.8, 16.1)	6.8 (6.0, 7.6)	12.5 (11.4, 13.6)	9.2 (8.2, 10.2)	21.3 (20.0, 22.7)	6.7 (5.9, 7.5)	2.5 (2.0, 3.1)	0.3 (0.1, 0.4)
Gabon	2569	26.3 (23.5, 29.1)	9.3 (7.4, 11.2)	17.9 (15.5, 20.3)	3.4 (2.3, 4.4)	30.1 (27.2, 33.1)	10.3 (8.4, 12.3)	2.0 (1.0, 2.9)	0.0 (0.0, 0.0)
Ghana	2324	15.2 (13.5, 16.9)	3.7 (2.7, 4.7)	12.4 (10.8, 13.9)	4.6 (3.4, 5.8)	22.4 (20.3, 24.5)	5.3 (4.3, 6.3)	0.9 (0.5, 1.3)	0.0 (0.0, 0.1)
Guinea	2933	16.4 (15.0, 17.9)	2.1 (1.5, 2.7)	12.5 (11.2, 13.8)	8.5 (7.4, 9.6)	25.1 (23.4, 26.8)	6.0 (5.0, 6.9)	0.8 (0.4, 1.2)	0.0 (0.0, 0.0)
Haiti	4858	34.8 (33.2, 36.4)	10.9 (9.8, 11.9)	22.1 (20.7, 23.5)	3.2 (2.7, 3.8)	35.0 (33.4, 36.6)	14.0 (12.8, 15.2)	2.6 (2.1, 3.1)	0.1 (0.0, 0.2)
India	187,195	13.3 (13.1, 13.6)	2.8 (2.7, 2.9)	9.2 (9.0, 9.3)	20.1 (19.8, 20.3)	27.3 (27.0, 27.6)	6.5 (6.3, 6.6)	1.5 (1.4, 1.6)	0.2 (0.1, 0.2)
Kenya	16,508	26.1 (25.3, 27.0)	9.2 (8.6, 9.8)	16.1 (15.3, 16.8)	4.1 (3.7, 4.5)	27.4 (26.5, 28.3)	10.8 (10.2, 11.4)	2.0 (1.7, 2.3)	0.1 (0.1, 0.2)
Lesotho	1083	17.1 (14.3, 19.9)	5.4 (3.8, 7.0)	12.9 (10.5, 15.3)	3.0 (1.9, 4.1)	19.6 (16.7, 22.6)	7.9 (6.0, 9.8)	0.7 (0.2, 1.1)	0.2 (0.0, 0.6)
Liberia	2774	31.2 (28.8, 33.6)	7.0 (5.7, 8.3)	24.3 (22.0, 26.5)	6.6 (5.2, 7.9)	30.2 (27.8, 32.6)	14.4 (12.6, 16.3)	2.8 (2.0, 3.5)	0.4 (0.2, 0.7)
Malawi	4607	31.6 (29.9, 33.2)	5.8 (5.0, 6.7)	22.6 (21.0, 24.1)	2.6 (2.1, 3.2)	30.7 (29.1, 32.4)	12.6 (11.4, 13.8)	2.1 (1.6, 2.6)	0.1 (0.0, 0.2)
Mali	6906	17.1 (16.1, 18.0)	2.2 (1.8, 2.6)	18.7 (17.6, 19.7)	8.6 (7.9, 9.4)	24.0 (22.9, 25.1)	8.1 (7.4, 8.8)	1.9 (1.6, 2.3)	0.1 (0.0, 0.2)
Mozambique	7846	14.3 (13.3, 15.2)	1.6 (1.3, 1.9)	11.8 (10.9, 12.7)	5.2 (4.6, 5.8)	19.7 (18.6, 20.8)	5.2 (4.6, 5.8)	0.8 (0.6, 1.1)	0.0 (0.0, 0.1)
Myanmar	3541	16.2 (14.8, 17.7)	3.1 (2.5, 3.7)	10.9 (9.7, 12.2)	6.5 (5.5, 7.5)	20.7 (19.1, 22.3)	6.3 (5.4, 7.3)	1.0 (0.7, 1.4)	0.1 (0.0, 0.2)
Namibia	1460	29.5 (26.8, 32.2)	6.5 (5.0, 8.0)	23.3 (20.8, 25.8)	7.6 (6.1, 9.1)	26.7 (24.2, 29.3)	15.0 (12.9, 17.2)	2.7 (1.8, 3.6)	0.5 (0.0, 1.0)
Nepal	2003	21.9 (19.9, 24.0)	2.9 (2.1, 3.7)	7.2 (5.9, 8.6)	8.9 (7.6, 10.3)	24.2 (22.0, 26.3)	6.6 (5.4, 7.8)	1.2 (0.7, 1.7)	0.0 (0.0, 0.1)
Nigeria	9088	27.4 (26.3, 28.4)	2.8 (2.4, 3.2)	13.8 (13.0, 14.6)	6.8 (6.2, 7.4)	26.7 (25.6, 27.7)	9.1 (8.4, 9.8)	1.8 (1.5, 2.1)	0.1 (0.1, 0.2)
Pakistan	3509	40.0 (37.5, 42.4)	14.0 (12.3, 15.8)	21.1 (19.0, 23.1)	6.3 (5.1, 7.4)	29.6 (27.3, 31.8)	18.0 (16.0, 20.0)	4.8 (3.7, 6.0)	0.3 (0.1, 0.5)
Rwanda	3160	20.6 (19.1, 22.1)	6.6 (5.7, 7.5)	13.5 (12.3, 14.8)	1.9 (1.4, 2.4)	20.2 (18.7, 21.7)	8.3 (7.3, 9.3)	1.9 (1.4, 2.4)	0.1 (0.0, 0.2)
Senegal	5083	13.7 (12.5, 14.9)	3.8 (3.0, 4.5)	15.6 (14.3, 16.8)	7.7 (6.8, 8.7)	18.9 (17.5, 20.3)	7.9 (6.9, 8.8)	1.7 (1.3, 2.0)	0.3 (0.1, 0.5)
Sierra Leone	3559	28.6 (26.9, 30.4)	5.1 (4.3, 5.9)	12.1 (10.9, 13.3)	9.5 (8.3, 10.6)	27.6 (25.9, 29.3)	10.2 (9.1, 11.4)	2.1 (1.6, 2.7)	0.2 (0.0, 0.4)
South Africa	928	22.0 (18.5, 25.4)	4.3 (2.5, 6.0)	12.3 (9.5, 15.0)	2.4 (1.1, 3.7)	26.5 (22.8, 30.1)	6.1 (4.0, 8.3)	0.7 (0.1, 1.4)	0.0 (0.0, 0.0)
Tanzania	7566	19.8 (18.7, 20.9)	3.9 (3.3, 4.4)	13.1 (12.1, 14.0)	4.3 (3.7, 4.9)	22.0 (20.9, 23.1)	7.6 (6.9, 8.4)	1.3 (1.0, 1.6)	0.0 (0.0, 0.0)
Timor-Leste	5199	13.4 (12.3, 14.5)	2.1 (1.6, 2.5)	10.6 (9.6, 11.6)	24.4 (23.0, 25.8)	29.3 (27.9, 30.8)	7.1 (6.2, 7.9)	2.2 (1.7, 2.7)	0.1 (0.0, 0.2)
Togo	2796	24.5 (22.7, 26.3)	3.4 (2.7, 4.1)	17.1 (15.5, 18.6)	6.8 (5.8, 7.8)	27.5 (25.6, 29.3)	9.6 (8.4, 10.9)	1.5 (1.0, 1.9)	0.2 (0.0, 0.3)
Uganda	3687	39.4 (37.6, 41.3)	10.8 (9.6, 12.0)	22.5 (21.0, 24.1)	3.3 (2.6, 3.9)	32.3 (30.5, 34.0)	15.3 (13.9, 16.6)	4.1 (3.4, 4.8)	0.2 (0.0, 0.4)
Zambia	7247	17.2 (16.1, 18.2)	1.8 (1.4, 2.1)	16.4 (15.4, 17.5)	4.1 (3.6, 4.7)	22.6 (21.4, 23.8)	7.0 (6.3, 7.6)	1.0 (0.7, 1.2)	0.0 (0.0, 0.1)
Zimbabwe	4223	14.4 (13.2, 15.6)	4.0 (3.4, 4.7)	18.4 (17.1, 19.8)	3.4 (2.8, 4.0)	25.4 (23.9, 26.9)	6.5 (5.7, 7.4)	0.6 (0.4, 0.9)	0.0 (0.0, 0.0)
**Total**	**380,357**	**18.8 (18.6, 18.9)**	**4.3 (4.2, 4.4)**	**12.5 (12.4, 12.7)**	**13.5 (13.3, 13.6)**	**26.6 (26.5, 26.8)**	**8.2 (8.1, 8.3)**	**1.8 (1.8, 1.9)**	**0.2 (0.1, 0.2)**

**Fig. 2:**
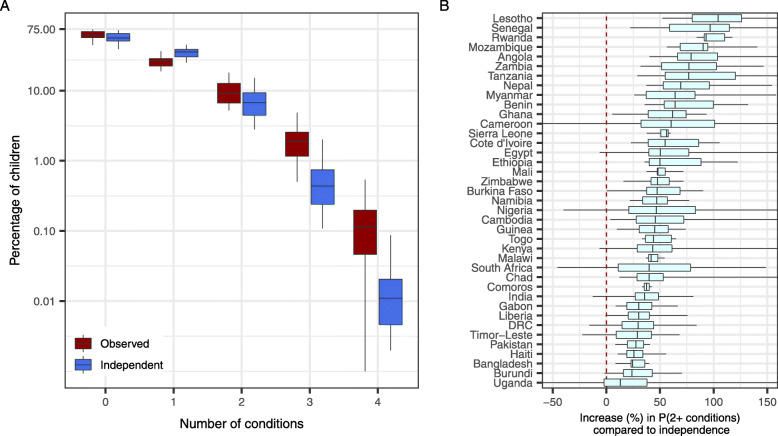
Increased probability of a child reporting two or more conditions. **A** Observed proportions of children with 0, 1, 2, 3 or 4 conditions reported at the country level (red boxes represent values from all 39 countries) compared with the expected distributions if conditions independently occurred (blue boxes). Boxes show the median (mid-line), 25th and 75th percentiles (hinges) and range (whiskers). **B** The percent increase in the observed probability of a child reporting more than one condition when compared with the frequency implied when assuming the conditions occurred independently. Distributions are shown for estimates at the first administrative unit within each country. Boxes show the median (mid-line), 25th and 75th percentiles (hinges) and range (whiskers), where those whiskers reaching the figure margins indicate the range extends beyond the limits shown here

We observed a greater proportion of children reporting more than 1 condition than would be expected if conditions were independent. The proportions of children reporting 2, 3 or 4 conditions were 8.2%, 1.8% and 0.2%, respectively (Table 1, Fig. 2A). There were consistent, large increases (country-level median increase range, 13–104%) in the observed probability of a child reporting more than one condition when compared with the estimated probability under the assumption that conditions occur independently (Fig. 2B). These trends persisted even when fever (the most general and most paired condition) was removed (Additional file 3).

**Fig. 3:**
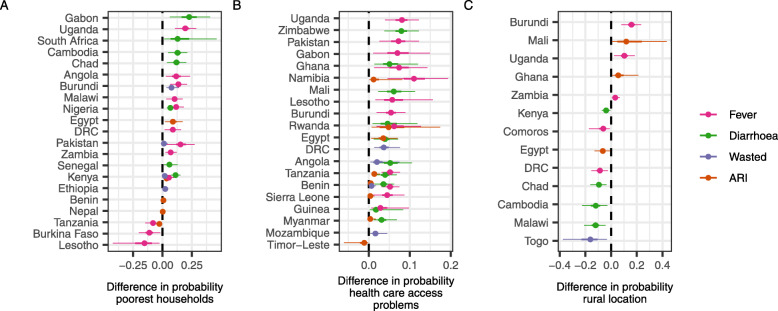
The relationship between child health and equity-relevant variables. Posterior predicted increases in the probability a child has an illness when comparing: **A** poorest households to the wealthiest, **B** children whose mothers reported difficulties in accessing the nearest health facility to those who did not and **C** those living in rural locations to those living in urban locations. Estimates shown had < 1% of the posterior crossing zero (dashed black line). Points show the median; thick lines show the 25–75% quantile range, and thin lines show the 2.5–97.5% quantile range. Countries are ordered by median estimate across conditions. Only trends with strong evidence (those where the probability that there was no difference was less than 1%) are shown for clarity

The impact of household wealth and health facility access on the probability of a children having at least one of the four conditions was marked (Fig. 3, Additional file 4). In 18 countries, children in the poorest households had a higher risk of at least one of the four conditions than those in the wealthiest households; in 3 countries, the relationship was reversed (Fig. 3A). Health facility access also showed a clear pattern: in 18 countries, having difficulty accessing the nearest healthcare facility led to an increased risk of at least one of the four conditions; one country, Timor-Leste, showed the reverse trend (Fig. 3B). Furthermore, access to healthcare was commonly associated with similar trends across multiple conditions (e.g. Rwanda, Egypt). The relationship with rurality was less clear with certain countries showing a higher probability of an illness in rural areas, whilst for others the probability was higher in urban areas (Fig. 3C). Visual inspection suggested some weak evidence of fever being more commonly associated with rural locations and diarrhoea with urban (Fig. 3C, Figure S1C). In some instances, the effect size was large, exceeding a + 20% increase in risk of illness for children in poorer households relative to wealthy households (e.g. Gabon), a + 5% increased risk for children with health facility access issues (e.g. Uganda) and a ± 10% change in risk for rural or urban locations (e.g. Burundi versus Togo).

**Fig. 4:**
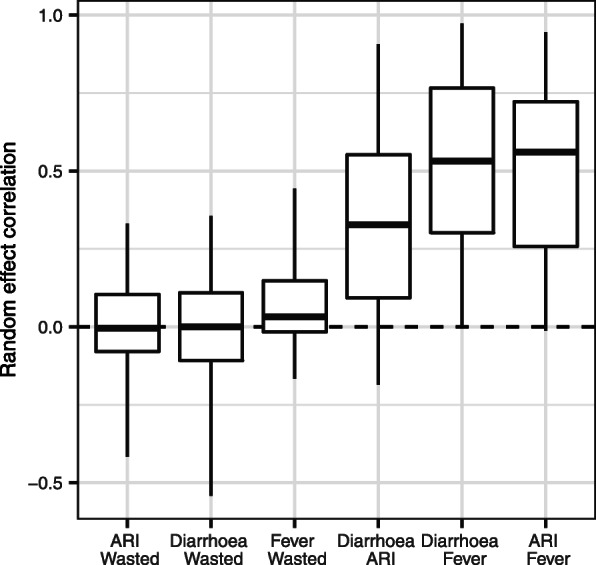
Correlations in adjusted prevalence of conditions across all countries. In each country, correlations in adjusted prevalence across all four conditions were calculated using the modelled cluster-level values, resulting in a single value between each pair for each country. This plot shows the distribution of these values across all countries for each pair of conditions. The boxes show the median (mid-line), 25th and 75th percentiles (hinges) and range (whiskers)

The multivariate structure of the model allows correlations between group-level (i.e. survey-cluster) effects terms to be compared. The intercept (fixed + group-level) intercepts (equation 3) can be interpreted as the cluster-level prevalence of each condition after adjusting for risk factors (“the adjusted prevalence”). For each country, we calculated correlations in adjusted prevalence across all four conditions using these cluster-level estimates. After controlling for all variables included in the regression, there remained consistent positive correlations between the adjusted prevalence of diarrhoea, fever and ARI (Fig. 4). For diarrhoea + fever, ARI + fever and diarrhoea + ARI, the posterior median correlation was positive in 100%, 95% and 91% of comparisons, respectively, and the lower 95% CrI positive in 72%, 70% and 58% of comparisons, respectively. Associations with wasting were less clear: for diarrhoea + wasting, ARI + wasting and fever + wasting the posterior median correlation was positive in 50%, 46% and 64% of comparisons, respectively, and the lower 95% CrI positive in 7%, 1% and 11% of comparisons, respectively.

## Discussion

In low and middle socioeconomic regions, malaria, diarrhoea, pneumonia and malnutrition cause substantial childhood morbidity and mortality. In our study, we found that the prevalence of each of fever, diarrhoea, ARI and wasting (outcomes associated with malaria, pneumonia and malnutrition), remain high whilst also being spatially heterogeneous both within and between countries. The prevalence of fever was higher than for diarrhoea, ARI or wasting for all countries except six (Angola, India, Mali, Senegal, Timor-Leste and Zimbabwe); this is not surprising given the many underlying causes of fever. Burundi recorded the highest prevalence of fever (43.1%), Liberia of diarrhoea (24.3%), Egypt of ARI (14.2%) and Timor-Leste of wasting (24.4%) (Table 1).

The burden of conditions studied was clustered both geographically and within children, with some areas and individuals bearing a disproportionate burden. The data showed significant overlap in the prevalence of fever, diarrhoea, ARI and wasting. Whilst other studies have showed the large overlap in outcomes [24], the consistency and strength of group-level correlations between outcomes, after adjusting for known risk factors, was striking even when considering shared aetiologies. There were consistent and large increases in the probability of a child reporting more than one condition when compared with the estimated probability under the assumption that conditions occur independently (Fig. 2). These increases also differed substantially between and within countries. Co-occurrence of conditions will be driven in part by shared aetiologies; however, the results here indicate the importance of shared determinants — some of which are shown in the equity analysis — in shaping the burden of illness for disadvantaged children.

The multilevel logistic regression showed clear trends of increased risk of fever, diarrhoea, ARI and wasting with respect to poverty for 18 countries (Fig. 2). DHS wealth quintiles are informed by a large set of covariates [25] and are a complex composite indicator, so our analysis does not resolve specific causal pathways. Nonetheless, reasonable links exist for many of the indicators included. Whilst we expected the poorest to experience a higher risk [26], the large effect size and persistence occurrence of such trends for four outcomes and multiple countries highlights the heightened risk felt by the most disadvantaged children. For three countries, there existed counter-intuitive trends in the opposite direction. Whilst it is not clear why these trends are observed, there is the potential for reporting bias in the questions relating to outcomes or for trends to be driven by localised outbreaks of fever, diarrhoea or ARI in specific survey subpopulations. The wealth indicator may also be serving as a simplified proxy for the complicated cascade that links poverty and health and may therefore not account for nuances in this relationship. Furthermore, the wealth index is relative and not directly comparable between countries, although those countries with counter-intuitive trends (Tanzania, Burkina Faso, Lesotho) were not outliers in terms of per-capita GDP.

Access to healthcare showed consistent trends with risk across outcomes and countries. Using a mother’s self-reported difficulties accessing the nearest health facility (physically or due to cost) as a proxy for a child’s access, it is clear that difficulties accessing healthcare are associated with higher risk for all four outcomes (Fig. 3). This may be due to good access being associated with improved treatment rates impacting transmission and reducing the number of onward cases. Access to healthcare could also be acting as a proxy for some other underlying mechanism related to risk (perhaps a general indicator of health system strength or general infrastructure in a location).

The urban/rural split was also significantly associated with risk in many countries (Fig. 3C), although clear uni-directional trends were absent and the complexity in the contextual factors across so many countries and multiple conditions makes it difficult to define key conclusions with respect to location. Previous evidence is also mixed with rural communities being more at risk in east Africa [27] and higher incidence of childhood diarrhoea in urban centres observed in southern India, with increased crowding leading to direct person-to-person transmission a potential urban driver [28].

There remained interesting spatial patterns in the group-level effects, capturing variability in risk not explained by the individual-level covariates. These patterns can be perceived from maps of these effects for a number of countries (e.g. Burundi, Kenya, Nigeria; Additional file 5). The persistence of these spatial relationships across multiple outcomes indicates a non-response specific driver that has not been adequately captured. Whilst purely speculation, some missed aspect of poverty, health system strength or infrastructure seem plausible explanations, although the spatial patterns do not appear to match well with established maps of poverty [29]. Specific factors may play important roles in different regions, for example food security (e.g. Northern Kenya), HIV prevalence and malaria prevalence (e.g. the shores of Lake Victoria) and security issues (e.g. North-Eastern Nigeria).

The countries included in this analysis represent a diverse range of contexts across multiple continents. As such, care should be taken as results may not be representative of all LMICs. However, broad trends are consistent and this report facilitates comparisons between countries as well as within. Of particular interest are countries from similar geographical areas or epidemiological contexts that show different patterns. For example, Uganda and Burundi show higher prevalence of conditions, more spatial clustering and more inequitable distribution than neighbouring Rwanda. Whist identifying specific drivers (for example in this instance these may include: governance, policy implementation strength, use of CHWs) for these differences is beyond the scope of this analysis, comparisons are nonetheless valuable for highlighting differences for future work.

Other limitations include potentially important influential factors that were not captured, such as cultural- or ethnicity-specific practices as well as comorbidities, such as the HIV status of a child. We did not include anaemia, another child health-related variable collected as part of the DHS, as it restricted the sample too greatly. There was indication of spatial clustering of areas of poor model fit (Additional file 6). There was large variation in spatial scale of the analyses, and for larger areas, we may expect greater spatial heterogeneity. Country-level differences also influence covariates, such as wealth and the urban-rural split, which are created or defined on a country-by-county basis. We did not include interaction terms in the model, partly to restrain the computational complexity and partly because of the covariates of interest in the DHS wealth index estimate already have rural-urban differences within the methodology and the interpretation of interaction terms additional to this was not clear. Furthermore, the urban-rural classification in the DHS varies by country and should be interpreted with some caution. Some significant relationships observed, given the large number of country-outcome relationships considered, may be a result of multiple-testing. Validation studies have shown a lack of specificity and sensitivity in correctly assessing child health outcomes [30] and questions regarding the occurrence of fever, diarrhoea or ARI in the last 2 weeks are likely subject to potential recall bias which could interact with outcomes or explanatory variables [31]. The inclusion criteria for covariates and children may lead to bias in the results if children with missing data are systematically associated with outcomes of interest. This issue may be particularly pertinent with respect to this equity analysis, where those most disadvantaged may also be more likely to give incomplete or missing information.

The cycle of inequality and disease risk is a pernicious trap, likely to self-perpetuate without specific intervention. Those most at risk are also those least able to access appropriate healthcare and the impact of morbidity and mortality feeds back to impact future development [12]. The results support calls for investments and improvement in health system infrastructure. The situation as it stands does offer an opportunity for synergistic impacts if horizontal approaches to healthcare can be adequately targeted to those most at need. Clustering of conditions increases the impact of successful targeting of those afflicted. iCCM is a horizontal approach to healthcare and one specifically targeting inequity. CHWs, trained under iCCM guidance to diagnose and treat (or refer) cases of malaria, pneumonia and diarrhoea and recognise malnutrition, will likely be key in the effort to expand and target healthcare coverage [16]. The co-occurrence of illnesses emphasises the need for CHWs to be trained and facilitated to be able to diagnose, treat and record patients presenting with multiple aetiologies to maximise impact.

## Conclusions

Despite reductions in childhood mortality being targeted by the Sustainable Development Goals, the number of children dying before the age of 5 years remains high. Many of these deaths are due to pneumonia, diarrhoea or malaria, are associated with malnutrition and could be prevented if there was access to timely appropriate healthcare. In this study, we show that a number of equity-relevant variables are consistently associated with increased risk of fever, diarrhoea and/or ARI in children under five. Targeting interventions based on equity could therefore play an important role in preventing childhood deaths.

## Supplementary Information

**Additional file 1.** A list of DHS surveys included in the analysis.

**Additional file 2.** Additional methods. Model details, model checking: convergence and goodness of fit and estimating the proportion of children with multiple conditions under the assumption of independence.

**Additional file 3: Figure S1:** P(2+ conditions | ARI, diarrhoea, wasting).

**Additional file 4.** Model parameter tables. Full regression outputs for each model.

**Additional file 5.** Spatial distribution of adjusted prevalence (AP).

**Additional file 6.** Model fit. Posterior predictive checks, AUC and posterior predictive spatial checks.

## Data Availability

The data that support the findings of this study are available online from the DHS program: https://dhsprogram.com/.

## Abbreviations

ARI: Acute respiratory infection
BCG: Bacillus Calmette–Guérin vaccine
CHW: Community health workers
CrI: Credible interval
DHS: Demographic and Health Surveys
DTP: Diphtheria, pertussis and tetanus vaccine
GAVI: The Global Alliance for Vaccines and Immunizations
GDP: Gross domestic product
Hib: Haemophilus influenzae type b
HIV: Human immunodeficiency virus
iCCM: Integrated community case management
LMICs: Low- and middle-income countries
SD: Standard deviation
UHC: Universal health coverage
UNICEF: United Nations Children’s Emergency Fund
WHO: World Health Organization
