# Supplementary material for: Health inequities and clustering of fever, acute respiratory infection, diarrhoea and wasting in children under five in low- and middle-income countries: a Demographic and Health Surveys analysis
Source: BMC Med. 2021 Jun 24;19:144. doi: 10.1186/s12916-021-02018-0 (PMC8223394; doi:10.1186/s12916-021-02018-0)
Supplement: Supplementary file 1 — Additional file 1. A list of DHS surveys included in the analysis. [file 12916_2021_2018_MOESM1_ESM.pdf]

Health inequities and clustering of fever, acute respiratory infection, diarrhoea and wasting in children under five in low- and middle-income countries: A Demographic and Health Surveys analysis.

Supplementary Information: Surveys

[1] “Angola (2015), Bangladesh (2011), Bangladesh (2014), Benin (2012), Benin (2017), Burkina Faso (2010), Burundi (2010), Burundi (2016), Cambodia (2010), Cambodia (2014), Cameroon (2011), Cameroon (2018), Chad (2014), Comoros (2012), Congo (2011), Congo Democratic Republic (2013), Cote d'Ivoire (2012), Egypt (2014), Ethiopia (2011), Ethiopia (2016), Gabon (2012), Gambia (2013), Ghana (2014), Guinea (2012), Guinea (2018), Haiti (2012), Haiti (2016), India (2015), Kenya (2014), Lesotho (2014), Liberia (2013), Malawi (2010), Malawi (2015), Mali (2012), Mali (2018), Mozambique (2011), Myanmar (2016), Namibia (2013), Nepal (2011), Nepal (2016), Niger (2012), Nigeria (2013), Nigeria (2018), Pakistan (2012), Pakistan (2017), Papua New Guinea (2017), Philippines (2013), Philippines (2017), Rwanda (2010), Rwanda (2015), Senegal (2010), Senegal (2012), Senegal (2014), Senegal (2015), Senegal (2016), Senegal (2017), Senegal (2018), Sierra Leone (2013), South Africa (2016), Tanzania (2010), Tanzania (2015), Timor-Leste (2016), Togo (2013), Uganda (2011), Uganda (2016), Zambia (2013), Zambia (2018), Zimbabwe (2010), Zimbabwe (2015),”
