# Supplementary material for: Health inequities and clustering of fever, acute respiratory infection, diarrhoea and wasting in children under five in low- and middle-income countries: a Demographic and Health Surveys analysis
Source: BMC Med. 2021 Jun 24;19:144. doi: 10.1186/s12916-021-02018-0 (PMC8223394; doi:10.1186/s12916-021-02018-0)
Supplement: Supplementary file 2 — Additional file 2. Additional methods. Model details, model checking: convergence and goodness of fit and estimating the proportion of children with multiple conditions under the assumption of independence. [file 12916_2021_2018_MOESM2_ESM.pdf]

Health inequities and clustering of fever, acute respiratory infection, diarrhoea and wasting in children under five in low- and middle-income countries: A Demographic and Health Surveys analysis.

Supplementary Information: Additional methods

Model details

Group-level coefficients are assumed to be correlated between responses, y, such that $\mu_{hj} \sim MVN\left(0, \Omega_G\right)$, where MVN is a multivariate normal distribution, with covariance matrix Ω_G. The model was fitted in a Bayesian framework using the brms R package [1], which is built upon Stan MCMC software that uses the No-U-Turn sampler (NUTS) - a dynamic variant of Hamiltonian Monte Carlo [2]. Non-informative normal priors were assigned to the fixed intercepts, β_0, $(Normal\left(0,\ 10\right))$, vector of effect size coefficients, β, $(Normal\left(0,\ 5\right))$ and group-level intercept standard deviations, μ_{hj}, $(Normal\left(0,\ 10\right))$. Non-informative normal prior standard deviations were chosen to be suitably large based on preliminary model fitting. The covariance matrix Ω_G was decomposed into the product of a correlation matrix Σ_G and a diagonal matrix whose diagonal elements are scale coefficients τ: $\Omega_G = \tau\Sigma_G\tau$. The correlation matrix was assigned an LKJ prior $(\Sigma_G \sim LKJ\left(1\right))$, representing a uniform distribution over all possible (4×4) correlation matrices; each element of the scale vector was assigned independent Student-t priors with mean zero, a scale of 1, and 4 degrees of freedom. The multivariate structure of the model allows correlation coefficients for cluster-level effects to be estimated, while adjusting for other covariates, and also allows for direct comparisons of effect sizes for covariates between outcomes of interest [3].

Model checking: convergence and goodness of fit

All models were fitted with four chains started from random initial positions within parameter space. Models were run for 4000 iterations after 500 "warm-up'' iterations. Convergence was assessed visually as well as quantitatively using the Rhat statistic [4], with model runs with Rhat<1.1 diagnosed as converged.

Model goodness of fit was assessed with posterior predictive checks (PPCs), which compare the distribution of model predictions and observed data [5]. These were conducted at the response level and disaggregated spatially and by covariates of interest. The Bayes p-value was used to summarise fit and is defined as, $Bayes\ p = P\left(y_{response} > y_{data}\right)$ where values $0.05 < Bayes\ p < 0.95$ were considered a reasonable fit. Additionally, receiver operating characteristic (ROC) curves were plotted for each country and response for a range of cut points (which determine the threshold probability where the response flips from 0 to 1). The area under the ROC curve indicates the probability that the model would correctly rank (order the response) a given pair of observations and can be used as a summary statistic of a given model's discriminatory ability [6]. For outputs see sections under Model fit.

1. Bürkner P-C. Advanced Bayesian Multilevel Modeling with the R Package brms. R J [Internet]. 2018;10:395–411. Available from: http://arxiv.org/abs/1705.11123

2. Carpenter B, Gelman A, Hoffman MD, Lee D, Goodrich B, Betancourt M, et al. Stan : A Probabilistic Programming Language. J Stat Softw [Internet]. 2017;76. Available from: http://www.jstatsoft.org/v76/i01/

3. Baldwin SA, Imel ZE, Braithwaite SR, Atkins DC. Analyzing Multiple Outcomes in Clinical Research Using Multivariate Multilevel Models. J Consult Clin Psychol. 2014;82:920–30.

4. Gelman A, Rubin D. Inference from Iterative Simulation Using Multiple Sequences. Stat Sci. 1992;7:457–511.

5. Lambert B. A Students Guide to Bayesian Statistics. SAGE Publications Ltd; 2018.

6. Steyerberg EW, Vickers AJ, Cook NR, Gerds T, Gonen M, Obuchowski N, et al. Prediction models: a framework for some traditional and novel measures. Epidemiology. 2013;21:128–38.

Estimating the proportion of children with multiple conditions under the assumption of independence

Assume that the probabilities of fever, p_f, diarrhoea, p_d, ARI, p_a and wasting p_w, are independent and set at the average probability across all data sets. The probability of a child having a single, double or triple condition is therefore:

$p\left(1 \text{ condition}|\ p_f, p_d, p_a, p_w\right) = p_f\left(1-p_d\right)\left(1-p_a\right)\left(1-p_w\right)+\left(1-p_f\right)p_d\left(1-p_a\right)\left(1-p_w\right)+\left(1-p_f\right)\left(1-p_d\right)p_a\left(1-p_w\right)+\left(1-p_f\right)\left(1-p_d\right)\left(1-p_a\right)p_w$

$p\left(2 \text{ conditions}|p_f, p_d, p_a, p_w\right) = p_f p_d\left(1-p_a\right)\left(1-p_w\right) + p_f\left(1-p_d\right)p_a\left(1-p_w\right) + \left(1-p_f\right)p_d p_a\left(1-p_w\right) + p_f\left(1-p_d\right)\left(1-p_a\right)p_w + \left(1-p_f\right)p_d\left(1-p_a\right)p_w + \left(1-p_f\right)\left(1-p_d\right)p_a p_w$

$p\left(3 \text{ conditions}|p_f, p_d, p_a, p_w\right) = \left(1-p_f\right)p_d p_a p_w + p_f\left(1-p_d\right)p_a p_w + p_f p_d\left(1-p_a\right)p_w + p_f p_d p_w\left(1-p_w\right)$

$p\left(4 \text{ conditions}|\ p_f p_d p_a p_w\right) = p_f p_d p_a p_w$
