# Supplementary material for: Health inequities and clustering of fever, acute respiratory infection, diarrhoea and wasting in children under five in low- and middle-income countries: a Demographic and Health Surveys analysis
Source: BMC Med. 2021 Jun 24;19:144. doi: 10.1186/s12916-021-02018-0 (PMC8223394; doi:10.1186/s12916-021-02018-0)

Health inequities and clustering of fever, acute respiratory infection, diarrhoea and wasting in children under five in low- and middle-income countries: A Demographic and Health Surveys analysis.

Supplementary Information: P(2+ conditions | ari, diarrhoea, wasting)

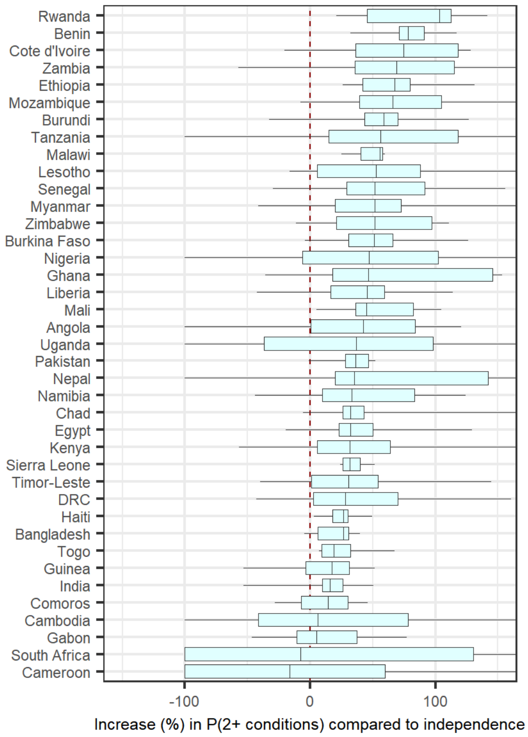

Supplement: Supplementary file 3 — Additional file 3: Figure S1: P(2+ conditions | ARI, diarrhoea, wasting). [file 12916_2021_2018_MOESM3_ESM.pdf]
