# Supplementary material for: Health inequities and clustering of fever, acute respiratory infection, diarrhoea and wasting in children under five in low- and middle-income countries: a Demographic and Health Surveys analysis
Source: BMC Med. 2021 Jun 24;19:144. doi: 10.1186/s12916-021-02018-0 (PMC8223394; doi:10.1186/s12916-021-02018-0)
Supplement: Supplementary file 4 — Additional file 4. Model parameter tables. Full regression outputs for each model. [file 12916_2021_2018_MOESM4_ESM.pdf]

Health inequities and clustering of fever, acute respiratory infection, diarrhoea and wasting in children under five in low- and middle-income countries: A Demographic and Health Surveys analysis.

Supplementary Information: Model parameter tables

All fixed effect coefficient estimates are shown in SI: Coefficient_Tables.

Name	Response	Group	Level	Estimate	Error	lci	uci
Andaman & Nicobar Islands	ari	Intercept	-	−17.16	8.72	−34.68	−0.44
Andaman & Nicobar Islands	ari	Age	1-2	−1.00	3.91	−8.93	6.31
Andaman & Nicobar Islands	ari	Age	2-3	1.18	3.81	−6.53	8.43
Andaman & Nicobar Islands	ari	Age	3-4	−0.59	4.03	−8.98	7.00
Andaman & Nicobar Islands	ari	Age	4-5	1.08	3.92	−6.99	8.73
Andaman & Nicobar Islands	ari	Month	May	−1.80	4.05	−10.03	5.81
Andaman & Nicobar Islands	ari	Month	June	−1.08	3.98	−9.17	6.62
Andaman & Nicobar Islands	ari	Month	July	2.01	3.87	−5.57	9.62
Andaman & Nicobar Islands	ari	Wealth	Poorer	0.10	4.43	−8.87	8.39
Andaman & Nicobar Islands	ari	Wealth	Middle	−0.58	4.30	−9.12	7.68
Andaman & Nicobar Islands	ari	Wealth	Richer	0.83	3.90	−6.95	8.44
Andaman & Nicobar Islands	ari	Wealth	Richest	0.27	4.12	−7.87	8.30
Andaman & Nicobar Islands	ari	Mothers education	Primary	−1.10	4.61	−10.37	7.69
Andaman & Nicobar Islands	ari	Mothers education	Secondary	0.21	3.95	−7.43	8.15
Andaman & Nicobar Islands	ari	Mothers education	Higher	1.01	4.19	−7.08	9.32
Andaman & Nicobar Islands	ari	Mothers age	20-30	1.09	3.89	−6.53	8.94
Andaman & Nicobar Islands	ari	Mothers age	30-40	−0.28	4.07	−8.38	7.49
Andaman & Nicobar Islands	ari	Sex	Female	−0.04	3.48	−6.89	6.73
Andaman & Nicobar Islands	ari	Birth weight	Average	−3.20	3.50	−10.03	3.73
Andaman & Nicobar Islands	ari	Birth weight	Below Average	−1.14	4.40	−10.07	7.11
Andaman & Nicobar Islands	ari	Breastfed	No	−2.81	3.80	−10.47	4.29
Andaman & Nicobar Islands	ari	Toilet	Unimproved	−1.08	4.36	−9.79	7.13
Andaman & Nicobar Islands	ari	Toilet	Improved	−0.52	4.56	−9.84	8.05
Andaman & Nicobar Islands	ari	Location	Urban	1.33	3.66	−5.89	8.51
Andaman & Nicobar Islands	ari	Cookign fuel	Solid	−1.28	3.90	−9.04	6.36
Andaman & Nicobar Islands	ari	Household size	05-Oct	3.47	3.61	−3.39	10.85
Andaman & Nicobar Islands	ari	Household size	10+	−0.42	4.54	−9.64	8.20
Andaman & Nicobar Islands	ari	LLIN	Yes	0.54	3.82	−7.27	7.82
Andaman & Nicobar Islands	ari	Access	Significant problem	0.09	4.32	−8.60	8.26
Andaman & Nicobar Islands	ari	Vaccination	-	−0.01	4.88	−9.60	9.57

Name	Response	Group	Level	Estimate	Error	lci	uci
Angola	dia	Intercept	-	−2.10	0.38	−2.85	−1.34
Angola	dia	Age	1-2	−0.17	0.13	−0.43	0.09
Angola	dia	Age	2-3	−0.63	0.14	−0.90	−0.35
Angola	dia	Age	3-4	−1.60	0.17	−1.94	−1.26
Angola	dia	Age	4-5	−1.79	0.19	−2.16	−1.42
Angola	dia	Month	February	−0.26	0.22	−0.70	0.16
Angola	dia	Month	March	−0.76	0.35	−1.45	−0.09
Angola	dia	Month	October	0.96	0.26	0.45	1.46
Angola	dia	Month	November	0.58	0.18	0.22	0.94
Angola	dia	Month	December	0.08	0.20	−0.31	0.46
Angola	dia	Wealth	Poorer	0.19	0.16	−0.11	0.49
Angola	dia	Wealth	Middle	0.38	0.23	−0.08	0.84
Angola	dia	Wealth	Richer	0.77	0.28	0.23	1.33
Angola	dia	Wealth	Richest	0.60	0.31	0.01	1.21
Angola	dia	Mothers education	Primary	0.12	0.12	−0.12	0.36
Angola	dia	Mothers education	Secondary	−0.13	0.15	−0.43	0.16
Angola	dia	Mothers education	Higher	−1.38	0.48	−2.40	−0.50
Angola	dia	Mothers age	20-30	−0.15	0.12	−0.38	0.10
Angola	dia	Mothers age	30-40	−0.23	0.15	−0.52	0.06
Angola	dia	Mothers age	40+	−0.75	0.27	−1.29	−0.25
Angola	dia	Sex	Female	−0.31	0.09	−0.49	−0.13
Angola	dia	Birth weight	Average	0.11	0.11	−0.09	0.32
Angola	dia	Birth weight	Below Average	0.43	0.16	0.12	0.74
Angola	dia	Breastfed	No	−0.37	0.11	−0.58	−0.15
Angola	dia	Toilet	Improved	0.04	0.13	−0.22	0.30
Angola	dia	Location	Urban	−0.10	0.19	−0.48	0.27
Angola	dia	Cookign fuel	Solid	0.53	0.17	0.19	0.87
Angola	dia	Household size	05-Oct	0.11	0.12	−0.12	0.33
Angola	dia	Household size	10+	−0.42	0.20	−0.81	−0.04
Angola	dia	LLIN	Yes	0.06	0.11	−0.15	0.28
Angola	dia	Access	Significant problem	0.25	0.11	0.04	0.47
Angola	dia	Vaccination	-	0.95	0.70	−0.41	2.31

Name	Response	Group	Level	Estimate	Error	lci	uci
Goa	wasted	Intercept	-	−1.98	4.64	−11.05	7.09
Goa	wasted	Age	1-2	0.22	0.98	−1.64	2.22
Goa	wasted	Age	2-3	0.53	1.00	−1.34	2.65
Goa	wasted	Age	3-4	0.31	1.01	−1.61	2.35
Goa	wasted	Age	4-5	−0.90	1.09	−2.99	1.29
Goa	wasted	Month	February	0.34	1.25	−1.99	2.96
Goa	wasted	Month	March	3.23	2.17	−0.70	7.84
Goa	wasted	Month	April	3.64	2.27	−0.56	8.30
Goa	wasted	Wealth	Middle	−2.33	1.30	−5.01	0.08
Goa	wasted	Wealth	Richer	−1.07	1.24	−3.63	1.31
Goa	wasted	Wealth	Richest	−1.15	1.34	−3.81	1.46
Goa	wasted	Mothers education	Primary	−0.70	1.21	−3.12	1.56
Goa	wasted	Mothers education	Secondary	0.23	0.90	−1.52	2.02
Goa	wasted	Mothers education	Higher	−0.59	1.14	−2.83	1.62
Goa	wasted	Mothers age	20-30	0.04	1.15	−2.20	2.41
Goa	wasted	Mothers age	30-40	−0.00	1.24	−2.39	2.50
Goa	wasted	Mothers age	40+	2.32	2.12	−1.85	6.62
Goa	wasted	Sex	Female	−0.27	0.51	−1.28	0.74
Goa	wasted	Birth weight	Average	−0.53	0.56	−1.63	0.60
Goa	wasted	Birth weight	Below Average	−0.11	0.90	−1.92	1.60
Goa	wasted	Breastfed	No	0.81	0.58	−0.32	1.95
Goa	wasted	Toilet	Unimproved	1.70	0.92	−0.10	3.55
Goa	wasted	Toilet	Improved	−3.04	3.49	−10.71	2.55
Goa	wasted	Location	Urban	−1.65	1.96	−5.96	1.78
Goa	wasted	Cookign fuel	Solid	−1.16	1.02	−3.29	0.74
Goa	wasted	Household size	05-Oct	0.20	0.54	−0.87	1.24
Goa	wasted	Household size	10+	−0.48	1.26	−3.06	1.83
Goa	wasted	LLIN	Yes	−1.73	2.29	−6.81	2.03
Goa	wasted	Access	Significant problem	−0.75	0.88	−2.57	0.88
Goa	wasted	Vaccination	-	−0.03	5.08	−9.93	9.67

Name	Response	Group	Level	Estimate	Error	lci	uci
Guinea	ari	Intercept	-	−3.61	2.21	−8.30	0.36
Guinea	ari	Age	1-2	−0.05	0.55	−1.08	1.07
Guinea	ari	Age	2-3	−0.07	0.56	−1.12	1.04
Guinea	ari	Age	3-4	0.03	0.56	−1.02	1.21
Guinea	ari	Age	4-5	−1.14	0.67	−2.46	0.16
Guinea	ari	Month	April	0.32	1.14	−1.76	2.71
Guinea	ari	Month	May	0.71	1.14	−1.39	3.11
Guinea	ari	Month	June	−1.23	1.90	−5.33	2.21
Guinea	ari	Wealth	Poorer	0.62	0.49	−0.32	1.61
Guinea	ari	Wealth	Middle	0.19	0.55	−0.87	1.26
Guinea	ari	Wealth	Richer	−0.67	0.71	−2.09	0.72
Guinea	ari	Wealth	Richest	−1.09	0.90	−2.89	0.66
Guinea	ari	Mothers education	Primary	0.06	0.50	−0.96	0.99
Guinea	ari	Mothers education	Secondary	−0.30	0.61	−1.55	0.83
Guinea	ari	Mothers education	Higher	−4.08	3.15	−11.25	0.69
Guinea	ari	Mothers age	20-30	−0.33	0.40	−1.09	0.46
Guinea	ari	Mothers age	30-40	−0.45	0.47	−1.35	0.46
Guinea	ari	Mothers age	40+	−1.07	0.84	−2.85	0.41
Guinea	ari	Sex	Female	−0.22	0.30	−0.81	0.38
Guinea	ari	Birth weight	Average	−0.35	0.36	−1.07	0.36
Guinea	ari	Birth weight	Below Average	−0.35	0.54	−1.47	0.63
Guinea	ari	Breastfed	No	−0.28	0.35	−0.94	0.40
Guinea	ari	Toilet	Unimproved	−0.27	0.37	−1.00	0.47
Guinea	ari	Toilet	Improved	−0.90	0.51	−1.93	0.04
Guinea	ari	Location	Urban	0.70	0.61	−0.50	1.88
Guinea	ari	Cookign fuel	Solid	0.35	1.66	−2.30	4.12
Guinea	ari	Household size	05-Oct	−0.69	0.42	−1.49	0.13
Guinea	ari	Household size	10+	0.22	0.46	−0.66	1.15
Guinea	ari	LLIN	Yes	−0.39	0.39	−1.17	0.35
Guinea	ari	Access	Significant problem	−0.37	0.36	−1.06	0.33
Guinea	ari	Vaccination	-	−3.55	2.69	−8.90	1.67

Name	Response	Group	Level	Estimate	Error	lci	uci
Guinea	dia	Intercept	-	−0.60	0.82	−2.24	1.02
Guinea	dia	Age	1-2	−0.20	0.19	−0.58	0.18
Guinea	dia	Age	2-3	−0.80	0.22	−1.23	−0.37
Guinea	dia	Age	3-4	−1.00	0.22	−1.44	−0.56
Guinea	dia	Age	4-5	−1.32	0.25	−1.81	−0.84
Guinea	dia	Month	April	−0.89	0.40	−1.68	−0.10
Guinea	dia	Month	May	−0.90	0.41	−1.71	−0.09
Guinea	dia	Month	June	−1.12	0.56	−2.24	−0.04
Guinea	dia	Wealth	Poorer	0.15	0.19	−0.21	0.52
Guinea	dia	Wealth	Middle	−0.06	0.22	−0.50	0.37
Guinea	dia	Wealth	Richer	0.41	0.28	−0.14	0.96
Guinea	dia	Wealth	Richest	0.61	0.37	−0.12	1.34
Guinea	dia	Mothers education	Primary	−0.06	0.21	−0.47	0.34
Guinea	dia	Mothers education	Secondary	0.05	0.23	−0.40	0.49
Guinea	dia	Mothers education	Higher	−0.57	0.56	−1.75	0.44
Guinea	dia	Mothers age	20-30	−0.29	0.18	−0.63	0.05
Guinea	dia	Mothers age	30-40	−0.19	0.20	−0.58	0.20
Guinea	dia	Mothers age	40+	−0.49	0.32	−1.13	0.12
Guinea	dia	Sex	Female	0.32	0.13	0.07	0.58
Guinea	dia	Birth weight	Average	−0.13	0.14	−0.41	0.14
Guinea	dia	Birth weight	Below Average	−0.39	0.21	−0.83	0.03
Guinea	dia	Breastfed	No	−0.53	0.15	−0.84	−0.22
Guinea	dia	Toilet	Unimproved	0.45	0.17	0.11	0.79
Guinea	dia	Toilet	Improved	−0.35	0.18	−0.70	0.01
Guinea	dia	Location	Urban	−0.41	0.28	−0.95	0.15
Guinea	dia	Cookign fuel	Solid	0.34	0.62	−0.80	1.63
Guinea	dia	Household size	05-Oct	−0.30	0.17	−0.63	0.04
Guinea	dia	Household size	10+	−0.46	0.21	−0.87	−0.05
Guinea	dia	LLIN	Yes	0.08	0.15	−0.21	0.36
Guinea	dia	Access	Significant problem	0.54	0.17	0.20	0.87
Guinea	dia	Vaccination	-	−2.42	1.24	−4.89	0.04

Name	Response	Group	Level	Estimate	Error	lci	uci
Guinea	fever	Intercept	-	−1.17	0.67	−2.48	0.15
Guinea	fever	Age	1-2	0.25	0.19	−0.11	0.62
Guinea	fever	Age	2-3	−0.06	0.20	−0.45	0.33
Guinea	fever	Age	3-4	−0.69	0.21	−1.11	−0.28
Guinea	fever	Age	4-5	−0.59	0.22	−1.02	−0.15
Guinea	fever	Month	April	0.10	0.36	−0.59	0.81
Guinea	fever	Month	May	0.12	0.37	−0.59	0.85
Guinea	fever	Month	June	0.15	0.48	−0.79	1.08
Guinea	fever	Wealth	Poorer	0.14	0.17	−0.18	0.47
Guinea	fever	Wealth	Middle	0.02	0.20	−0.37	0.39
Guinea	fever	Wealth	Richer	0.15	0.25	−0.34	0.65
Guinea	fever	Wealth	Richest	0.48	0.32	−0.14	1.12
Guinea	fever	Mothers education	Primary	0.18	0.18	−0.17	0.53
Guinea	fever	Mothers education	Secondary	−0.10	0.20	−0.51	0.29
Guinea	fever	Mothers education	Higher	−0.94	0.52	−2.05	0.02
Guinea	fever	Mothers age	20-30	−0.11	0.16	−0.42	0.21
Guinea	fever	Mothers age	30-40	0.26	0.17	−0.08	0.61
Guinea	fever	Mothers age	40+	0.08	0.27	−0.45	0.60
Guinea	fever	Sex	Female	−0.14	0.11	−0.35	0.08
Guinea	fever	Birth weight	Average	−0.08	0.12	−0.32	0.16
Guinea	fever	Birth weight	Below Average	0.29	0.17	−0.05	0.61
Guinea	fever	Breastfed	No	−0.15	0.13	−0.40	0.10
Guinea	fever	Toilet	Unimproved	−0.03	0.15	−0.32	0.26
Guinea	fever	Toilet	Improved	−0.24	0.16	−0.56	0.07
Guinea	fever	Location	Urban	0.05	0.23	−0.41	0.51
Guinea	fever	Cookign fuel	Solid	−0.26	0.48	−1.15	0.71
Guinea	fever	Household size	05-Oct	−0.28	0.15	−0.58	0.03
Guinea	fever	Household size	10+	−0.56	0.19	−0.93	−0.20
Guinea	fever	LLIN	Yes	0.01	0.13	−0.24	0.26
Guinea	fever	Access	Significant problem	0.61	0.14	0.33	0.89
Guinea	fever	Vaccination	-	−2.33	1.05	−4.39	−0.30

Name	Response	Group	Level	Estimate	Error	lci	uci
Guinea	wasted	Intercept	-	−2.39	0.88	−4.18	−0.73
Guinea	wasted	Age	1-2	−0.14	0.26	−0.63	0.37
Guinea	wasted	Age	2-3	−0.04	0.27	−0.57	0.48
Guinea	wasted	Age	3-4	−0.37	0.28	−0.91	0.17
Guinea	wasted	Age	4-5	−0.22	0.28	−0.78	0.33
Guinea	wasted	Month	April	0.18	0.49	−0.75	1.17
Guinea	wasted	Month	May	0.26	0.50	−0.70	1.28
Guinea	wasted	Month	June	0.47	0.65	−0.78	1.80
Guinea	wasted	Wealth	Poorer	0.24	0.23	−0.20	0.70
Guinea	wasted	Wealth	Middle	0.40	0.26	−0.11	0.91
Guinea	wasted	Wealth	Richer	0.07	0.34	−0.59	0.73
Guinea	wasted	Wealth	Richest	−0.12	0.42	−0.95	0.70
Guinea	wasted	Mothers education	Primary	−0.45	0.27	−0.99	0.07
Guinea	wasted	Mothers education	Secondary	−0.29	0.28	−0.84	0.24
Guinea	wasted	Mothers education	Higher	−0.07	0.56	−1.22	0.96
Guinea	wasted	Mothers age	20-30	0.31	0.23	−0.13	0.76
Guinea	wasted	Mothers age	30-40	−0.02	0.26	−0.51	0.48
Guinea	wasted	Mothers age	40+	0.10	0.37	−0.64	0.83
Guinea	wasted	Sex	Female	−0.00	0.14	−0.30	0.29
Guinea	wasted	Birth weight	Average	0.04	0.16	−0.27	0.36
Guinea	wasted	Birth weight	Below Average	0.30	0.23	−0.16	0.76
Guinea	wasted	Breastfed	No	−0.24	0.18	−0.58	0.11
Guinea	wasted	Toilet	Unimproved	0.11	0.20	−0.27	0.50
Guinea	wasted	Toilet	Improved	−0.04	0.21	−0.46	0.37
Guinea	wasted	Location	Urban	0.28	0.32	−0.34	0.91
Guinea	wasted	Cookign fuel	Solid	−0.67	0.58	−1.79	0.49
Guinea	wasted	Household size	05-Oct	−0.06	0.21	−0.46	0.35
Guinea	wasted	Household size	10+	−0.42	0.25	−0.93	0.08
Guinea	wasted	LLIN	Yes	−0.19	0.18	−0.54	0.15
Guinea	wasted	Access	Significant problem	−0.05	0.18	−0.40	0.31
Guinea	wasted	Vaccination	-	0.79	1.38	−1.93	3.53

Name	Response	Group	Level	Estimate	Error	lci	uci
Gujarat	ari	Intercept	-	−5.79	2.48	−10.59	−1.02
Gujarat	ari	Age	1-2	0.64	0.45	−0.22	1.56
Gujarat	ari	Age	2-3	0.77	0.45	−0.07	1.68
Gujarat	ari	Age	3-4	0.29	0.45	−0.56	1.19
Gujarat	ari	Age	4-5	0.16	0.47	−0.72	1.10
Gujarat	ari	Month	March	−0.23	0.52	−1.24	0.79
Gujarat	ari	Month	April	−0.38	0.53	−1.43	0.67
Gujarat	ari	Month	May	−3.90	1.04	−6.16	−2.12
Gujarat	ari	Month	June	−1.73	0.71	−3.19	−0.42
Gujarat	ari	Wealth	Poorer	0.19	0.50	−0.76	1.20
Gujarat	ari	Wealth	Middle	0.19	0.56	−0.88	1.34
Gujarat	ari	Wealth	Richer	−0.72	0.71	−2.11	0.68
Gujarat	ari	Wealth	Richest	−1.06	0.77	−2.57	0.49
Gujarat	ari	Mothers education	Primary	−0.28	0.48	−1.26	0.62
Gujarat	ari	Mothers education	Secondary	0.46	0.37	−0.25	1.19
Gujarat	ari	Mothers education	Higher	1.30	0.51	0.30	2.33
Gujarat	ari	Mothers age	20-30	−0.02	0.44	−0.84	0.88
Gujarat	ari	Mothers age	30-40	0.10	0.53	−0.91	1.15
Gujarat	ari	Sex	Female	−0.16	0.24	−0.63	0.31
Gujarat	ari	Birth weight	Average	0.02	0.28	−0.51	0.58
Gujarat	ari	Birth weight	Below Average	−0.41	0.42	−1.25	0.39
Gujarat	ari	Breastfed	No	0.02	0.25	−0.47	0.51
Gujarat	ari	Toilet	Unimproved	0.14	0.40	−0.64	0.94
Gujarat	ari	Toilet	Improved	0.42	0.53	−0.63	1.43
Gujarat	ari	Location	Urban	0.42	0.49	−0.54	1.39
Gujarat	ari	Cookign fuel	Solid	−0.58	0.49	−1.52	0.41
Gujarat	ari	Household size	05-Oct	−1.04	0.29	−1.61	−0.49
Gujarat	ari	Household size	10+	−0.83	0.40	−1.64	−0.07
Gujarat	ari	LLIN	Yes	−2.80	1.91	−7.27	0.11
Gujarat	ari	Access	Significant problem	0.66	0.27	0.14	1.18
Gujarat	ari	Vaccination	-	0.03	5.12	−9.84	10.01

Name	Response	Group	Level	Estimate	Error	lci	uci
Gujarat	dia	Intercept	-	−0.89	2.18	−5.07	3.37
Gujarat	dia	Age	1-2	−0.71	0.15	−0.99	−0.42
Gujarat	dia	Age	2-3	−1.21	0.16	−1.52	−0.89
Gujarat	dia	Age	3-4	−1.86	0.17	−2.19	−1.52
Gujarat	dia	Age	4-5	−2.47	0.21	−2.88	−2.07
Gujarat	dia	Month	March	−0.29	0.24	−0.75	0.17
Gujarat	dia	Month	April	−0.63	0.24	−1.10	−0.15
Gujarat	dia	Month	May	−0.76	0.25	−1.24	−0.28
Gujarat	dia	Month	June	−0.96	0.28	−1.50	−0.42
Gujarat	dia	Wealth	Poorer	−0.38	0.20	−0.77	0.03
Gujarat	dia	Wealth	Middle	−0.41	0.23	−0.86	0.03
Gujarat	dia	Wealth	Richer	−0.39	0.29	−0.95	0.16
Gujarat	dia	Wealth	Richest	−0.73	0.34	−1.41	−0.07
Gujarat	dia	Mothers education	Primary	0.67	0.17	0.32	1.01
Gujarat	dia	Mothers education	Secondary	0.20	0.15	−0.10	0.50
Gujarat	dia	Mothers education	Higher	−0.74	0.29	−1.32	−0.18
Gujarat	dia	Mothers age	20-30	−0.09	0.18	−0.43	0.28
Gujarat	dia	Mothers age	30-40	0.01	0.22	−0.42	0.44
Gujarat	dia	Sex	Female	−0.18	0.10	−0.38	0.02
Gujarat	dia	Birth weight	Average	−0.04	0.13	−0.28	0.22
Gujarat	dia	Birth weight	Below Average	−0.24	0.18	−0.60	0.10
Gujarat	dia	Breastfed	No	0.04	0.12	−0.19	0.27
Gujarat	dia	Toilet	Unimproved	−0.58	0.17	−0.92	−0.26
Gujarat	dia	Toilet	Improved	−0.10	0.25	−0.60	0.40
Gujarat	dia	Location	Urban	−0.15	0.20	−0.53	0.23
Gujarat	dia	Cookign fuel	Solid	0.07	0.19	−0.30	0.46
Gujarat	dia	Household size	05-Oct	−0.06	0.14	−0.34	0.21
Gujarat	dia	Household size	10+	0.13	0.19	−0.24	0.49
Gujarat	dia	LLIN	Yes	−1.19	0.68	−2.64	0.01
Gujarat	dia	Access	Significant problem	0.30	0.12	0.07	0.53
Gujarat	dia	Vaccination	-	0.04	4.94	−9.46	9.57

Name	Response	Group	Level	Estimate	Error	lci	uci
Gujarat	fever	Intercept	-	−2.97	2.22	−7.35	1.40
Gujarat	fever	Age	1-2	0.34	0.16	0.02	0.66
Gujarat	fever	Age	2-3	−0.41	0.18	−0.76	−0.05
Gujarat	fever	Age	3-4	−0.60	0.17	−0.93	−0.26
Gujarat	fever	Age	4-5	−0.59	0.18	−0.95	−0.23
Gujarat	fever	Month	March	−0.46	0.24	−0.94	0.01
Gujarat	fever	Month	April	−0.59	0.25	−1.07	−0.09
Gujarat	fever	Month	May	−1.23	0.27	−1.76	−0.72
Gujarat	fever	Month	June	−1.47	0.30	−2.06	−0.89
Gujarat	fever	Wealth	Poorer	0.05	0.21	−0.36	0.46
Gujarat	fever	Wealth	Middle	0.01	0.23	−0.45	0.47
Gujarat	fever	Wealth	Richer	0.53	0.28	−0.03	1.08
Gujarat	fever	Wealth	Richest	0.29	0.33	−0.35	0.94
Gujarat	fever	Mothers education	Primary	0.56	0.18	0.22	0.90
Gujarat	fever	Mothers education	Secondary	0.31	0.16	0.01	0.63
Gujarat	fever	Mothers education	Higher	−0.27	0.25	−0.77	0.23
Gujarat	fever	Mothers age	20-30	0.29	0.19	−0.06	0.66
Gujarat	fever	Mothers age	30-40	0.30	0.22	−0.14	0.74
Gujarat	fever	Sex	Female	−0.05	0.10	−0.25	0.15
Gujarat	fever	Birth weight	Average	0.47	0.13	0.22	0.72
Gujarat	fever	Birth weight	Below Average	0.39	0.17	0.04	0.73
Gujarat	fever	Breastfed	No	−0.31	0.11	−0.53	−0.09
Gujarat	fever	Toilet	Unimproved	−0.28	0.16	−0.60	0.03
Gujarat	fever	Toilet	Improved	0.55	0.24	0.09	1.02
Gujarat	fever	Location	Urban	−0.25	0.20	−0.65	0.14
Gujarat	fever	Cookign fuel	Solid	0.21	0.19	−0.15	0.57
Gujarat	fever	Household size	05-Oct	−0.28	0.13	−0.54	−0.02
Gujarat	fever	Household size	10+	−0.03	0.18	−0.39	0.32
Gujarat	fever	LLIN	Yes	−0.56	0.57	−1.75	0.47
Gujarat	fever	Access	Significant problem	0.13	0.12	−0.10	0.36
Gujarat	fever	Vaccination	-	−0.01	5.06	−9.89	9.83

Name	Response	Group	Level	Estimate	Error	lci	uci
Gujarat	wasted	Intercept	-	−0.55	2.19	−4.85	3.67
Gujarat	wasted	Age	1-2	−0.40	0.11	−0.62	−0.18
Gujarat	wasted	Age	2-3	−0.50	0.11	−0.72	−0.28
Gujarat	wasted	Age	3-4	−0.47	0.11	−0.68	−0.25
Gujarat	wasted	Age	4-5	−0.78	0.12	−1.00	−0.55
Gujarat	wasted	Month	March	0.30	0.16	−0.03	0.62
Gujarat	wasted	Month	April	0.19	0.17	−0.13	0.51
Gujarat	wasted	Month	May	0.27	0.16	−0.05	0.58
Gujarat	wasted	Month	June	0.39	0.18	0.04	0.74
Gujarat	wasted	Wealth	Poorer	0.10	0.13	−0.14	0.35
Gujarat	wasted	Wealth	Middle	−0.13	0.14	−0.40	0.14
Gujarat	wasted	Wealth	Richer	−0.30	0.18	−0.64	0.04
Gujarat	wasted	Wealth	Richest	−0.27	0.20	−0.66	0.13
Gujarat	wasted	Mothers education	Primary	−0.03	0.11	−0.24	0.18
Gujarat	wasted	Mothers education	Secondary	−0.10	0.09	−0.28	0.08
Gujarat	wasted	Mothers education	Higher	−0.07	0.15	−0.37	0.22
Gujarat	wasted	Mothers age	20-30	−0.10	0.12	−0.33	0.12
Gujarat	wasted	Mothers age	30-40	−0.23	0.14	−0.50	0.04
Gujarat	wasted	Sex	Female	−0.18	0.06	−0.30	−0.06
Gujarat	wasted	Birth weight	Average	0.15	0.08	−0.01	0.30
Gujarat	wasted	Birth weight	Below Average	0.48	0.11	0.27	0.69
Gujarat	wasted	Breastfed	No	−0.05	0.07	−0.19	0.10
Gujarat	wasted	Toilet	Unimproved	−0.03	0.10	−0.23	0.17
Gujarat	wasted	Toilet	Improved	−0.08	0.16	−0.40	0.24
Gujarat	wasted	Location	Urban	−0.32	0.12	−0.56	−0.09
Gujarat	wasted	Cookign fuel	Solid	−0.06	0.12	−0.29	0.16
Gujarat	wasted	Household size	05-Oct	−0.06	0.08	−0.21	0.10
Gujarat	wasted	Household size	10+	−0.30	0.12	−0.54	−0.07
Gujarat	wasted	LLIN	Yes	0.15	0.29	−0.44	0.69
Gujarat	wasted	Access	Significant problem	−0.09	0.07	−0.23	0.05
Gujarat	wasted	Vaccination	-	0.06	5.01	−9.67	9.97

Name	Response	Group	Level	Estimate	Error	lci	uci
Haiti	ari	Intercept	-	−2.99	0.63	−4.23	−1.77
Haiti	ari	Age	1-2	−0.01	0.18	−0.37	0.36
Haiti	ari	Age	2-3	0.09	0.18	−0.26	0.46
Haiti	ari	Age	3-4	−0.05	0.19	−0.42	0.33
Haiti	ari	Age	4-5	−0.09	0.19	−0.46	0.30
Haiti	ari	Month	February	0.00	0.23	−0.47	0.46
Haiti	ari	Month	March	−0.12	0.21	−0.53	0.29
Haiti	ari	Month	April	0.35	0.26	−0.17	0.86
Haiti	ari	Month	December	0.44	0.21	0.03	0.85
Haiti	ari	Wealth	Poorer	−0.01	0.16	−0.33	0.30
Haiti	ari	Wealth	Middle	−0.15	0.20	−0.54	0.24
Haiti	ari	Wealth	Richer	0.01	0.24	−0.46	0.48
Haiti	ari	Wealth	Richest	0.09	0.28	−0.47	0.65
Haiti	ari	Mothers education	Primary	0.60	0.16	0.29	0.91
Haiti	ari	Mothers education	Secondary	0.34	0.19	−0.02	0.71
Haiti	ari	Mothers education	Higher	0.73	0.33	0.09	1.35
Haiti	ari	Mothers age	20-30	0.02	0.18	−0.33	0.37
Haiti	ari	Mothers age	30-40	0.11	0.19	−0.26	0.48
Haiti	ari	Mothers age	40+	0.66	0.25	0.17	1.14
Haiti	ari	Sex	Female	−0.09	0.10	−0.29	0.12
Haiti	ari	Birth weight	Average	−0.05	0.13	−0.30	0.21
Haiti	ari	Birth weight	Below Average	0.42	0.14	0.14	0.70
Haiti	ari	Breastfed	No	−0.13	0.11	−0.36	0.09
Haiti	ari	Toilet	Unimproved	−0.13	0.14	−0.40	0.14
Haiti	ari	Location	Urban	−0.27	0.20	−0.67	0.11
Haiti	ari	Cookign fuel	Solid	0.40	0.35	−0.26	1.12
Haiti	ari	Household size	05-Oct	0.17	0.12	−0.07	0.40
Haiti	ari	Household size	10+	0.13	0.21	−0.28	0.53
Haiti	ari	LLIN	Yes	−0.00	0.14	−0.28	0.27
Haiti	ari	Access	Significant problem	−0.09	0.14	−0.36	0.19
Haiti	ari	Vaccination	-	−0.76	0.94	−2.61	1.10

Name	Response	Group	Level	Estimate	Error	lci	uci
Angola	fever	Intercept	-	−1.61	0.37	−2.35	−0.88
Angola	fever	Age	1-2	−0.08	0.14	−0.35	0.21
Angola	fever	Age	2-3	−0.28	0.15	−0.58	0.02
Angola	fever	Age	3-4	−0.50	0.16	−0.80	−0.17
Angola	fever	Age	4-5	−0.67	0.17	−1.01	−0.35
Angola	fever	Month	February	−0.49	0.21	−0.90	−0.07
Angola	fever	Month	March	−1.17	0.36	−1.89	−0.49
Angola	fever	Month	October	0.15	0.26	−0.36	0.67
Angola	fever	Month	November	0.02	0.18	−0.33	0.38
Angola	fever	Month	December	−0.33	0.19	−0.70	0.04
Angola	fever	Wealth	Poorer	−0.05	0.15	−0.34	0.24
Angola	fever	Wealth	Middle	−0.43	0.23	−0.87	0.01
Angola	fever	Wealth	Richer	−0.31	0.28	−0.87	0.24
Angola	fever	Wealth	Richest	−0.75	0.31	−1.37	−0.15
Angola	fever	Mothers education	Primary	0.22	0.12	−0.01	0.45
Angola	fever	Mothers education	Secondary	0.28	0.15	−0.02	0.59
Angola	fever	Mothers education	Higher	−0.21	0.39	−0.99	0.53
Angola	fever	Mothers age	20-30	−0.10	0.12	−0.34	0.14
Angola	fever	Mothers age	30-40	−0.07	0.15	−0.36	0.21
Angola	fever	Mothers age	40+	0.47	0.22	0.05	0.89
Angola	fever	Sex	Female	0.02	0.09	−0.15	0.19
Angola	fever	Birth weight	Average	0.07	0.10	−0.13	0.27
Angola	fever	Birth weight	Below Average	0.31	0.16	0.01	0.61
Angola	fever	Breastfed	No	−0.13	0.10	−0.34	0.07
Angola	fever	Toilet	Improved	0.09	0.13	−0.17	0.34
Angola	fever	Location	Urban	0.03	0.19	−0.32	0.41
Angola	fever	Cookign fuel	Solid	0.10	0.18	−0.25	0.46
Angola	fever	Household size	05-Oct	−0.09	0.11	−0.30	0.13
Angola	fever	Household size	10+	−0.52	0.19	−0.90	−0.15
Angola	fever	LLIN	Yes	−0.14	0.11	−0.36	0.08
Angola	fever	Access	Significant problem	0.12	0.11	−0.09	0.33
Angola	fever	Vaccination	-	0.89	0.70	−0.43	2.29

Name	Response	Group	Level	Estimate	Error	lci	uci
Haiti	dia	Intercept	-	−1.37	0.50	−2.37	−0.43
Haiti	dia	Age	1-2	−0.25	0.12	−0.49	−0.02
Haiti	dia	Age	2-3	−0.85	0.13	−1.11	−0.60
Haiti	dia	Age	3-4	−1.41	0.14	−1.68	−1.12
Haiti	dia	Age	4-5	−1.91	0.15	−2.20	−1.60
Haiti	dia	Month	February	−0.03	0.17	−0.37	0.30
Haiti	dia	Month	March	−0.35	0.15	−0.64	−0.06
Haiti	dia	Month	April	−0.14	0.19	−0.52	0.24
Haiti	dia	Month	December	0.12	0.15	−0.18	0.42
Haiti	dia	Wealth	Poorer	−0.20	0.12	−0.44	0.05
Haiti	dia	Wealth	Middle	−0.04	0.15	−0.32	0.25
Haiti	dia	Wealth	Richer	0.14	0.18	−0.21	0.49
Haiti	dia	Wealth	Richest	−0.27	0.22	−0.70	0.17
Haiti	dia	Mothers education	Primary	0.12	0.12	−0.11	0.35
Haiti	dia	Mothers education	Secondary	0.18	0.14	−0.08	0.45
Haiti	dia	Mothers education	Higher	−0.15	0.29	−0.72	0.40
Haiti	dia	Mothers age	20-30	−0.04	0.13	−0.30	0.23
Haiti	dia	Mothers age	30-40	−0.12	0.14	−0.40	0.16
Haiti	dia	Mothers age	40+	−0.04	0.20	−0.44	0.36
Haiti	dia	Sex	Female	−0.08	0.08	−0.23	0.07
Haiti	dia	Birth weight	Average	−0.16	0.10	−0.34	0.03
Haiti	dia	Birth weight	Below Average	−0.21	0.11	−0.44	0.01
Haiti	dia	Breastfed	No	−0.12	0.09	−0.30	0.06
Haiti	dia	Toilet	Unimproved	0.19	0.10	−0.01	0.39
Haiti	dia	Location	Urban	0.16	0.15	−0.13	0.44
Haiti	dia	Cookign fuel	Solid	0.75	0.32	0.13	1.43
Haiti	dia	Household size	05-Oct	−0.16	0.09	−0.33	0.02
Haiti	dia	Household size	10+	0.08	0.15	−0.21	0.38
Haiti	dia	LLIN	Yes	−0.08	0.10	−0.28	0.13
Haiti	dia	Access	Significant problem	0.23	0.11	0.02	0.44
Haiti	dia	Vaccination	-	0.43	0.68	−0.91	1.76

Name	Response	Group	Level	Estimate	Error	lci	uci
Haiti	fever	Intercept	-	−0.77	0.43	−1.62	0.07
Haiti	fever	Age	1-2	−0.06	0.12	−0.30	0.18
Haiti	fever	Age	2-3	−0.17	0.12	−0.41	0.08
Haiti	fever	Age	3-4	−0.36	0.13	−0.61	−0.11
Haiti	fever	Age	4-5	−0.57	0.13	−0.82	−0.32
Haiti	fever	Month	February	−0.11	0.16	−0.43	0.21
Haiti	fever	Month	March	−0.04	0.14	−0.31	0.24
Haiti	fever	Month	April	−0.08	0.19	−0.44	0.28
Haiti	fever	Month	December	0.72	0.15	0.44	1.01
Haiti	fever	Wealth	Poorer	−0.01	0.11	−0.22	0.20
Haiti	fever	Wealth	Middle	−0.01	0.13	−0.27	0.24
Haiti	fever	Wealth	Richer	0.25	0.16	−0.06	0.55
Haiti	fever	Wealth	Richest	−0.11	0.19	−0.49	0.27
Haiti	fever	Mothers education	Primary	−0.23	0.10	−0.42	−0.04
Haiti	fever	Mothers education	Secondary	−0.25	0.12	−0.48	−0.02
Haiti	fever	Mothers education	Higher	−0.17	0.24	−0.63	0.29
Haiti	fever	Mothers age	20-30	0.06	0.12	−0.18	0.30
Haiti	fever	Mothers age	30-40	−0.01	0.13	−0.25	0.24
Haiti	fever	Mothers age	40+	−0.07	0.18	−0.42	0.28
Haiti	fever	Sex	Female	−0.05	0.07	−0.18	0.09
Haiti	fever	Birth weight	Average	−0.04	0.09	−0.21	0.13
Haiti	fever	Birth weight	Below Average	0.11	0.10	−0.08	0.31
Haiti	fever	Breastfed	No	−0.27	0.08	−0.43	−0.12
Haiti	fever	Toilet	Unimproved	0.08	0.09	−0.10	0.26
Haiti	fever	Location	Urban	−0.17	0.14	−0.44	0.09
Haiti	fever	Cookign fuel	Solid	0.57	0.24	0.11	1.06
Haiti	fever	Household size	05-Oct	−0.11	0.08	−0.26	0.05
Haiti	fever	Household size	10+	−0.12	0.14	−0.40	0.15
Haiti	fever	LLIN	Yes	−0.01	0.09	−0.20	0.18
Haiti	fever	Access	Significant problem	0.10	0.09	−0.08	0.28
Haiti	fever	Vaccination	-	−0.39	0.65	−1.66	0.85

Name	Response	Group	Level	Estimate	Error	lci	uci
Haiti	wasted	Intercept	-	−3.08	0.96	−5.00	−1.23
Haiti	wasted	Age	1-2	−0.64	0.24	−1.10	−0.16
Haiti	wasted	Age	2-3	−1.29	0.27	−1.83	−0.77
Haiti	wasted	Age	3-4	−1.87	0.33	−2.53	−1.26
Haiti	wasted	Age	4-5	−1.69	0.31	−2.31	−1.09
Haiti	wasted	Month	February	−0.14	0.38	−0.90	0.59
Haiti	wasted	Month	March	−0.04	0.32	−0.68	0.58
Haiti	wasted	Month	April	−0.18	0.44	−1.06	0.66
Haiti	wasted	Month	December	0.34	0.32	−0.29	0.96
Haiti	wasted	Wealth	Poorer	−0.23	0.28	−0.79	0.30
Haiti	wasted	Wealth	Middle	−0.44	0.35	−1.12	0.24
Haiti	wasted	Wealth	Richer	0.04	0.40	−0.76	0.84
Haiti	wasted	Wealth	Richest	0.04	0.49	−0.92	1.00
Haiti	wasted	Mothers education	Primary	0.43	0.28	−0.10	0.99
Haiti	wasted	Mothers education	Secondary	0.28	0.33	−0.35	0.94
Haiti	wasted	Mothers education	Higher	0.35	0.60	−0.86	1.51
Haiti	wasted	Mothers age	20-30	−0.43	0.28	−0.97	0.11
Haiti	wasted	Mothers age	30-40	−0.65	0.30	−1.24	−0.06
Haiti	wasted	Mothers age	40+	−0.00	0.40	−0.80	0.78
Haiti	wasted	Sex	Female	−0.20	0.18	−0.56	0.15
Haiti	wasted	Birth weight	Average	0.54	0.26	0.05	1.08
Haiti	wasted	Birth weight	Below Average	0.61	0.29	0.06	1.18
Haiti	wasted	Breastfed	No	−0.02	0.21	−0.43	0.39
Haiti	wasted	Toilet	Unimproved	0.29	0.25	−0.19	0.77
Haiti	wasted	Location	Urban	0.25	0.33	−0.39	0.90
Haiti	wasted	Cookign fuel	Solid	−0.04	0.55	−1.07	1.09
Haiti	wasted	Household size	05-Oct	−0.02	0.21	−0.42	0.39
Haiti	wasted	Household size	10+	0.26	0.33	−0.40	0.90
Haiti	wasted	LLIN	Yes	−0.65	0.28	−1.21	−0.12
Haiti	wasted	Access	Significant problem	0.11	0.25	−0.37	0.61
Haiti	wasted	Vaccination	-	−0.33	1.43	−3.18	2.53

Name	Response	Group	Level	Estimate	Error	lci	uci
Haryana	ari	Intercept	-	−3.29	2.97	−9.07	2.59
Haryana	ari	Age	1-2	−0.22	0.32	−0.83	0.40
Haryana	ari	Age	2-3	−0.29	0.32	−0.92	0.33
Haryana	ari	Age	3-4	−0.80	0.35	−1.48	−0.12
Haryana	ari	Age	4-5	−0.76	0.35	−1.44	−0.08
Haryana	ari	Month	March	−0.09	0.31	−0.70	0.53
Haryana	ari	Month	April	−0.78	0.35	−1.46	−0.09
Haryana	ari	Month	May	−1.27	0.41	−2.08	−0.47
Haryana	ari	Month	June	−0.87	0.46	−1.81	0.00
Haryana	ari	Wealth	Poorer	0.00	0.50	−0.93	1.03
Haryana	ari	Wealth	Middle	−0.11	0.49	−1.03	0.89
Haryana	ari	Wealth	Richer	−0.05	0.53	−1.05	1.01
Haryana	ari	Wealth	Richest	−0.23	0.57	−1.32	0.92
Haryana	ari	Mothers education	Primary	−0.10	0.33	−0.74	0.52
Haryana	ari	Mothers education	Secondary	−0.14	0.27	−0.65	0.38
Haryana	ari	Mothers education	Higher	−0.51	0.42	−1.36	0.29
Haryana	ari	Mothers age	20-30	0.10	0.36	−0.57	0.84
Haryana	ari	Mothers age	30-40	0.28	0.43	−0.56	1.16
Haryana	ari	Sex	Female	−0.18	0.19	−0.56	0.19
Haryana	ari	Birth weight	Average	−0.24	0.26	−0.73	0.28
Haryana	ari	Birth weight	Below Average	0.28	0.36	−0.44	0.98
Haryana	ari	Breastfed	No	0.17	0.21	−0.23	0.58
Haryana	ari	Toilet	Unimproved	0.15	0.29	−0.42	0.72
Haryana	ari	Toilet	Improved	0.12	0.56	−1.04	1.16
Haryana	ari	Location	Urban	0.08	0.27	−0.44	0.62
Haryana	ari	Cookign fuel	Solid	0.01	0.27	−0.51	0.56
Haryana	ari	Household size	05-Oct	0.13	0.28	−0.42	0.71
Haryana	ari	Household size	10+	0.40	0.35	−0.28	1.10
Haryana	ari	LLIN	Yes	1.11	0.53	0.02	2.08
Haryana	ari	Access	Significant problem	0.37	0.21	−0.05	0.78
Haryana	ari	Vaccination	-	−0.02	5.10	−10.09	9.90

Name	Response	Group	Level	Estimate	Error	lci	uci
Haryana	dia	Intercept	-	−1.28	2.83	−6.84	4.21
Haryana	dia	Age	1-2	−0.37	0.18	−0.72	−0.01
Haryana	dia	Age	2-3	−0.86	0.20	−1.23	−0.47
Haryana	dia	Age	3-4	−1.12	0.21	−1.53	−0.71
Haryana	dia	Age	4-5	−1.57	0.24	−2.04	−1.10
Haryana	dia	Month	March	−0.08	0.22	−0.52	0.37
Haryana	dia	Month	April	−0.38	0.24	−0.85	0.09
Haryana	dia	Month	May	0.29	0.23	−0.16	0.75
Haryana	dia	Month	June	−0.05	0.28	−0.59	0.50
Haryana	dia	Wealth	Poorer	−0.48	0.38	−1.23	0.25
Haryana	dia	Wealth	Middle	−0.93	0.38	−1.65	−0.19
Haryana	dia	Wealth	Richer	−1.04	0.39	−1.83	−0.26
Haryana	dia	Wealth	Richest	−0.65	0.41	−1.45	0.14
Haryana	dia	Mothers education	Primary	−0.13	0.23	−0.59	0.32
Haryana	dia	Mothers education	Secondary	−0.05	0.18	−0.41	0.32
Haryana	dia	Mothers education	Higher	−0.15	0.25	−0.63	0.34
Haryana	dia	Mothers age	20-30	−0.10	0.24	−0.55	0.38
Haryana	dia	Mothers age	30-40	0.05	0.30	−0.53	0.63
Haryana	dia	Sex	Female	−0.02	0.13	−0.27	0.22
Haryana	dia	Birth weight	Average	0.04	0.18	−0.31	0.41
Haryana	dia	Birth weight	Below Average	0.60	0.25	0.10	1.06
Haryana	dia	Breastfed	No	−0.14	0.14	−0.41	0.13
Haryana	dia	Toilet	Unimproved	−0.17	0.22	−0.61	0.25
Haryana	dia	Toilet	Improved	−0.56	0.54	−1.72	0.39
Haryana	dia	Location	Urban	0.11	0.17	−0.23	0.43
Haryana	dia	Cookign fuel	Solid	0.11	0.17	−0.22	0.45
Haryana	dia	Household size	05-Oct	−0.01	0.17	−0.34	0.32
Haryana	dia	Household size	10+	0.36	0.22	−0.07	0.80
Haryana	dia	LLIN	Yes	0.64	0.34	−0.05	1.29
Haryana	dia	Access	Significant problem	0.17	0.14	−0.11	0.44
Haryana	dia	Vaccination	-	−0.03	4.93	−9.74	9.83

Name	Response	Group	Level	Estimate	Error	lci	uci
Haryana	fever	Intercept	-	−1.39	2.89	−7.14	4.36
Haryana	fever	Age	1-2	−0.14	0.19	−0.51	0.23
Haryana	fever	Age	2-3	−0.49	0.20	−0.88	−0.10
Haryana	fever	Age	3-4	−0.60	0.20	−0.99	−0.20
Haryana	fever	Age	4-5	−0.81	0.21	−1.22	−0.39
Haryana	fever	Month	March	−0.37	0.21	−0.80	0.04
Haryana	fever	Month	April	−0.70	0.22	−1.13	−0.27
Haryana	fever	Month	May	−0.78	0.23	−1.24	−0.33
Haryana	fever	Month	June	−1.14	0.29	−1.73	−0.58
Haryana	fever	Wealth	Poorer	−0.16	0.36	−0.86	0.54
Haryana	fever	Wealth	Middle	−0.29	0.36	−0.98	0.42
Haryana	fever	Wealth	Richer	−0.32	0.37	−1.03	0.43
Haryana	fever	Wealth	Richest	−0.29	0.40	−1.06	0.50
Haryana	fever	Mothers education	Primary	0.54	0.19	0.16	0.92
Haryana	fever	Mothers education	Secondary	0.06	0.18	−0.29	0.40
Haryana	fever	Mothers education	Higher	−0.20	0.24	−0.67	0.27
Haryana	fever	Mothers age	20-30	0.15	0.23	−0.29	0.62
Haryana	fever	Mothers age	30-40	−0.05	0.29	−0.62	0.54
Haryana	fever	Sex	Female	−0.23	0.12	−0.47	−0.00
Haryana	fever	Birth weight	Average	−0.29	0.16	−0.60	0.02
Haryana	fever	Birth weight	Below Average	0.09	0.23	−0.37	0.53
Haryana	fever	Breastfed	No	−0.12	0.13	−0.37	0.14
Haryana	fever	Toilet	Unimproved	0.16	0.19	−0.23	0.54
Haryana	fever	Toilet	Improved	−0.22	0.41	−1.05	0.54
Haryana	fever	Location	Urban	−0.04	0.17	−0.38	0.29
Haryana	fever	Cookign fuel	Solid	−0.20	0.17	−0.52	0.13
Haryana	fever	Household size	05-Oct	0.24	0.17	−0.08	0.57
Haryana	fever	Household size	10+	0.44	0.22	0.02	0.86
Haryana	fever	LLIN	Yes	0.36	0.38	−0.42	1.07
Haryana	fever	Access	Significant problem	0.07	0.13	−0.19	0.33
Haryana	fever	Vaccination	-	−0.07	5.07	−10.13	9.82

Name	Response	Group	Level	Estimate	Error	lci	uci
Haryana	wasted	Intercept	-	−1.62	2.82	−7.19	3.78
Haryana	wasted	Age	1-2	−0.34	0.15	−0.64	−0.05
Haryana	wasted	Age	2-3	−0.22	0.14	−0.51	0.07
Haryana	wasted	Age	3-4	−0.34	0.15	−0.63	−0.05
Haryana	wasted	Age	4-5	−0.36	0.15	−0.66	−0.07
Haryana	wasted	Month	March	−0.24	0.17	−0.57	0.11
Haryana	wasted	Month	April	0.10	0.17	−0.23	0.43
Haryana	wasted	Month	May	0.54	0.17	0.21	0.86
Haryana	wasted	Month	June	0.33	0.20	−0.04	0.72
Haryana	wasted	Wealth	Poorer	0.40	0.34	−0.25	1.07
Haryana	wasted	Wealth	Middle	0.46	0.34	−0.19	1.13
Haryana	wasted	Wealth	Richer	0.45	0.35	−0.20	1.14
Haryana	wasted	Wealth	Richest	0.20	0.36	−0.48	0.92
Haryana	wasted	Mothers education	Primary	−0.06	0.15	−0.35	0.21
Haryana	wasted	Mothers education	Secondary	−0.07	0.12	−0.31	0.17
Haryana	wasted	Mothers education	Higher	−0.17	0.17	−0.51	0.17
Haryana	wasted	Mothers age	20-30	0.01	0.16	−0.30	0.32
Haryana	wasted	Mothers age	30-40	0.08	0.20	−0.31	0.48
Haryana	wasted	Sex	Female	−0.05	0.08	−0.22	0.11
Haryana	wasted	Birth weight	Average	0.01	0.12	−0.23	0.24
Haryana	wasted	Birth weight	Below Average	0.18	0.18	−0.17	0.52
Haryana	wasted	Breastfed	No	−0.13	0.09	−0.31	0.04
Haryana	wasted	Toilet	Unimproved	0.26	0.14	−0.00	0.54
Haryana	wasted	Toilet	Improved	−0.20	0.34	−0.89	0.44
Haryana	wasted	Location	Urban	0.11	0.12	−0.12	0.35
Haryana	wasted	Cookign fuel	Solid	−0.21	0.11	−0.43	0.01
Haryana	wasted	Household size	05-Oct	0.07	0.11	−0.14	0.28
Haryana	wasted	Household size	10+	−0.14	0.16	−0.45	0.18
Haryana	wasted	LLIN	Yes	0.38	0.26	−0.14	0.88
Haryana	wasted	Access	Significant problem	0.15	0.10	−0.04	0.34
Haryana	wasted	Vaccination	-	−0.01	4.94	−9.45	9.71

Name	Response	Group	Level	Estimate	Error	lci	uci
Himachal Pradesh	ari	Intercept	-	−6.09	4.43	−15.00	2.48
Himachal Pradesh	ari	Age	1-2	0.30	0.98	−1.51	2.39
Himachal Pradesh	ari	Age	2-3	−0.54	1.14	−2.76	1.79
Himachal Pradesh	ari	Age	3-4	−0.04	1.04	−2.00	2.14
Himachal Pradesh	ari	Age	4-5	−0.77	1.14	−3.01	1.53
Himachal Pradesh	ari	Month	March	−0.13	0.76	−1.61	1.37
Himachal Pradesh	ari	Month	April	−1.12	1.01	−3.24	0.76
Himachal Pradesh	ari	Month	May	−2.08	1.56	−5.66	0.44
Himachal Pradesh	ari	Month	June	−0.75	0.94	−2.66	1.01
Himachal Pradesh	ari	Month	July	−1.96	2.42	−7.58	1.79
Himachal Pradesh	ari	Wealth	Poorer	−0.06	1.85	−3.62	3.79
Himachal Pradesh	ari	Wealth	Middle	0.44	1.67	−2.55	4.05
Himachal Pradesh	ari	Wealth	Richer	0.71	1.68	−2.29	4.36
Himachal Pradesh	ari	Wealth	Richest	1.23	1.77	−1.91	5.06
Himachal Pradesh	ari	Mothers education	Primary	1.13	1.96	−2.42	5.35
Himachal Pradesh	ari	Mothers education	Secondary	1.39	1.77	−1.57	5.43
Himachal Pradesh	ari	Mothers education	Higher	1.42	1.89	−1.84	5.58
Himachal Pradesh	ari	Mothers age	20-30	−0.99	1.02	−2.81	1.19
Himachal Pradesh	ari	Mothers age	30-40	−1.87	1.42	−4.79	0.87
Himachal Pradesh	ari	Sex	Female	0.30	0.57	−0.81	1.44
Himachal Pradesh	ari	Birth weight	Average	−0.08	1.05	−1.88	2.22
Himachal Pradesh	ari	Birth weight	Below Average	0.52	1.17	−1.59	3.00
Himachal Pradesh	ari	Breastfed	No	0.10	0.62	−1.11	1.29
Himachal Pradesh	ari	Toilet	Unimproved	0.56	0.80	−1.10	2.06
Himachal Pradesh	ari	Toilet	Improved	−0.10	1.39	−3.31	2.18
Himachal Pradesh	ari	Location	Urban	−0.34	1.70	−4.27	2.45
Himachal Pradesh	ari	Cookign fuel	Solid	1.00	0.91	−0.66	2.94
Himachal Pradesh	ari	Household size	05-Oct	−0.44	0.65	−1.69	0.89
Himachal Pradesh	ari	Household size	10+	−3.14	2.00	−7.87	−0.04
Himachal Pradesh	ari	LLIN	Yes	−1.61	4.06	−10.32	5.35
Himachal Pradesh	ari	Access	Significant problem	0.45	0.62	−0.82	1.62
Himachal Pradesh	ari	Vaccination	-	−0.11	4.99	−9.84	9.73

Name	Response	Group	Level	Estimate	Error	lci	uci
Himachal Pradesh	dia	Intercept	-	−5.21	3.96	−13.16	2.37
Himachal Pradesh	dia	Age	1-2	−0.37	0.44	−1.22	0.53
Himachal Pradesh	dia	Age	2-3	−0.64	0.47	−1.54	0.30
Himachal Pradesh	dia	Age	3-4	−2.05	0.64	−3.40	−0.88
Himachal Pradesh	dia	Age	4-5	−1.71	0.58	−2.88	−0.58
Himachal Pradesh	dia	Month	March	0.15	0.48	−0.77	1.10
Himachal Pradesh	dia	Month	April	−0.69	0.59	−1.87	0.47
Himachal Pradesh	dia	Month	May	0.12	0.56	−0.97	1.22
Himachal Pradesh	dia	Month	June	−0.42	0.54	−1.52	0.63
Himachal Pradesh	dia	Month	July	−0.19	1.06	−2.52	1.64
Himachal Pradesh	dia	Wealth	Poorer	−0.74	1.08	−2.76	1.51
Himachal Pradesh	dia	Wealth	Middle	−0.55	1.02	−2.41	1.59
Himachal Pradesh	dia	Wealth	Richer	−0.54	1.03	−2.39	1.64
Himachal Pradesh	dia	Wealth	Richest	−0.08	1.09	−2.08	2.17
Himachal Pradesh	dia	Mothers education	Primary	3.36	1.85	0.34	7.55
Himachal Pradesh	dia	Mothers education	Secondary	2.83	1.80	−0.06	6.91
Himachal Pradesh	dia	Mothers education	Higher	2.92	1.84	−0.08	7.11
Himachal Pradesh	dia	Mothers age	20-30	0.32	0.89	−1.18	2.27
Himachal Pradesh	dia	Mothers age	30-40	0.14	0.97	−1.59	2.23
Himachal Pradesh	dia	Sex	Female	−0.17	0.32	−0.82	0.46
Himachal Pradesh	dia	Birth weight	Average	0.29	0.64	−0.87	1.62
Himachal Pradesh	dia	Birth weight	Below Average	0.94	0.70	−0.35	2.37
Himachal Pradesh	dia	Breastfed	No	0.11	0.35	−0.58	0.78
Himachal Pradesh	dia	Toilet	Unimproved	0.40	0.47	−0.54	1.28
Himachal Pradesh	dia	Toilet	Improved	0.44	0.68	−1.00	1.66
Himachal Pradesh	dia	Location	Urban	0.21	0.60	−1.04	1.34
Himachal Pradesh	dia	Cookign fuel	Solid	−0.34	0.44	−1.21	0.52
Himachal Pradesh	dia	Household size	05-Oct	0.09	0.41	−0.71	0.92
Himachal Pradesh	dia	Household size	10+	0.34	0.59	−0.83	1.48
Himachal Pradesh	dia	LLIN	Yes	2.09	2.13	−2.40	6.02
Himachal Pradesh	dia	Access	Significant problem	0.23	0.36	−0.51	0.90
Himachal Pradesh	dia	Vaccination	-	0.03	5.06	−9.87	9.93

Name	Response	Group	Level	Estimate	Error	lci	uci
Himachal Pradesh	fever	Intercept	-	−2.84	3.55	−9.87	4.14
Himachal Pradesh	fever	Age	1-2	−0.24	0.38	−0.98	0.51
Himachal Pradesh	fever	Age	2-3	−0.22	0.38	−0.97	0.55
Himachal Pradesh	fever	Age	3-4	−0.12	0.37	−0.85	0.63
Himachal Pradesh	fever	Age	4-5	−0.70	0.42	−1.52	0.11
Himachal Pradesh	fever	Month	March	−0.01	0.34	−0.67	0.66
Himachal Pradesh	fever	Month	April	−0.35	0.39	−1.11	0.39
Himachal Pradesh	fever	Month	May	−0.50	0.46	−1.41	0.36
Himachal Pradesh	fever	Month	June	−0.18	0.37	−0.92	0.56
Himachal Pradesh	fever	Month	July	−0.15	0.72	−1.69	1.17
Himachal Pradesh	fever	Wealth	Poorer	−1.27	0.82	−2.83	0.40
Himachal Pradesh	fever	Wealth	Middle	−0.75	0.76	−2.18	0.80
Himachal Pradesh	fever	Wealth	Richer	−0.70	0.77	−2.14	0.87
Himachal Pradesh	fever	Wealth	Richest	−0.61	0.81	−2.16	1.01
Himachal Pradesh	fever	Mothers education	Primary	1.36	1.11	−0.61	3.78
Himachal Pradesh	fever	Mothers education	Secondary	1.90	1.04	0.13	4.17
Himachal Pradesh	fever	Mothers education	Higher	1.51	1.08	−0.37	3.86
Himachal Pradesh	fever	Mothers age	20-30	0.11	0.58	−0.92	1.34
Himachal Pradesh	fever	Mothers age	30-40	0.14	0.64	−1.04	1.45
Himachal Pradesh	fever	Sex	Female	−0.11	0.23	−0.55	0.32
Himachal Pradesh	fever	Birth weight	Average	−0.37	0.39	−1.11	0.41
Himachal Pradesh	fever	Birth weight	Below Average	0.43	0.44	−0.43	1.29
Himachal Pradesh	fever	Breastfed	No	−0.01	0.24	−0.48	0.45
Himachal Pradesh	fever	Toilet	Unimproved	0.23	0.33	−0.43	0.85
Himachal Pradesh	fever	Toilet	Improved	0.09	0.52	−0.98	1.06
Himachal Pradesh	fever	Location	Urban	0.19	0.46	−0.76	1.06
Himachal Pradesh	fever	Cookign fuel	Solid	−0.09	0.31	−0.70	0.53
Himachal Pradesh	fever	Household size	05-Oct	0.25	0.30	−0.33	0.85
Himachal Pradesh	fever	Household size	10+	0.27	0.43	−0.59	1.12
Himachal Pradesh	fever	LLIN	Yes	−2.89	3.54	−10.85	2.67
Himachal Pradesh	fever	Access	Significant problem	0.27	0.25	−0.23	0.76
Himachal Pradesh	fever	Vaccination	-	0.03	5.08	−10.09	10.00

Name	Response	Group	Level	Estimate	Error	lci	uci
Angola	wasted	Intercept	-	−2.98	0.57	−4.13	−1.87
Angola	wasted	Age	1-2	−0.43	0.20	−0.82	−0.04
Angola	wasted	Age	2-3	−0.88	0.23	−1.33	−0.43
Angola	wasted	Age	3-4	−1.91	0.29	−2.49	−1.35
Angola	wasted	Age	4-5	−1.30	0.26	−1.81	−0.78
Angola	wasted	Month	February	−0.03	0.28	−0.59	0.52
Angola	wasted	Month	March	−0.22	0.43	−1.10	0.59
Angola	wasted	Month	October	−0.49	0.40	−1.30	0.29
Angola	wasted	Month	November	−0.25	0.26	−0.74	0.26
Angola	wasted	Month	December	−0.53	0.28	−1.10	0.01
Angola	wasted	Wealth	Poorer	−0.31	0.24	−0.80	0.17
Angola	wasted	Wealth	Middle	−0.48	0.37	−1.22	0.24
Angola	wasted	Wealth	Richer	−0.70	0.45	−1.59	0.17
Angola	wasted	Wealth	Richest	−0.67	0.48	−1.62	0.27
Angola	wasted	Mothers education	Primary	−0.13	0.18	−0.48	0.22
Angola	wasted	Mothers education	Secondary	−0.48	0.24	−0.95	−0.01
Angola	wasted	Mothers education	Higher	−0.75	0.64	−2.10	0.42
Angola	wasted	Mothers age	20-30	0.34	0.21	−0.06	0.77
Angola	wasted	Mothers age	30-40	0.24	0.25	−0.24	0.74
Angola	wasted	Mothers age	40+	0.03	0.41	−0.80	0.78
Angola	wasted	Sex	Female	−0.46	0.15	−0.75	−0.18
Angola	wasted	Birth weight	Average	0.71	0.19	0.34	1.10
Angola	wasted	Birth weight	Below Average	0.93	0.27	0.40	1.45
Angola	wasted	Breastfed	No	0.35	0.17	0.01	0.70
Angola	wasted	Toilet	Improved	−0.17	0.21	−0.57	0.24
Angola	wasted	Location	Urban	0.47	0.28	−0.06	1.02
Angola	wasted	Cookign fuel	Solid	−0.01	0.28	−0.57	0.53
Angola	wasted	Household size	05-Oct	−0.13	0.18	−0.48	0.22
Angola	wasted	Household size	10+	−0.24	0.29	−0.82	0.32
Angola	wasted	LLIN	Yes	−0.22	0.19	−0.60	0.14
Angola	wasted	Access	Significant problem	0.53	0.18	0.18	0.89
Angola	wasted	Vaccination	-	−0.00	0.99	−1.93	1.96

Name	Response	Group	Level	Estimate	Error	lci	uci
Himachal Pradesh	wasted	Intercept	-	−1.30	3.42	−7.97	5.33
Himachal Pradesh	wasted	Age	1-2	−0.49	0.38	−1.21	0.27
Himachal Pradesh	wasted	Age	2-3	−0.44	0.38	−1.18	0.32
Himachal Pradesh	wasted	Age	3-4	−0.71	0.39	−1.45	0.06
Himachal Pradesh	wasted	Age	4-5	−0.58	0.39	−1.33	0.18
Himachal Pradesh	wasted	Month	March	−0.41	0.38	−1.15	0.37
Himachal Pradesh	wasted	Month	April	0.15	0.38	−0.57	0.91
Himachal Pradesh	wasted	Month	May	0.53	0.41	−0.26	1.36
Himachal Pradesh	wasted	Month	June	0.10	0.38	−0.63	0.86
Himachal Pradesh	wasted	Month	July	−0.06	0.72	−1.54	1.25
Himachal Pradesh	wasted	Wealth	Poorer	0.62	0.88	−0.99	2.50
Himachal Pradesh	wasted	Wealth	Middle	0.01	0.89	−1.62	1.87
Himachal Pradesh	wasted	Wealth	Richer	0.07	0.90	−1.52	1.97
Himachal Pradesh	wasted	Wealth	Richest	−0.24	0.94	−1.95	1.68
Himachal Pradesh	wasted	Mothers education	Primary	−0.30	0.69	−1.65	1.08
Himachal Pradesh	wasted	Mothers education	Secondary	0.24	0.60	−0.85	1.45
Himachal Pradesh	wasted	Mothers education	Higher	−0.07	0.67	−1.35	1.29
Himachal Pradesh	wasted	Mothers age	20-30	−0.25	0.53	−1.22	0.84
Himachal Pradesh	wasted	Mothers age	30-40	0.09	0.58	−1.01	1.26
Himachal Pradesh	wasted	Sex	Female	0.07	0.22	−0.37	0.51
Himachal Pradesh	wasted	Birth weight	Average	−0.09	0.39	−0.82	0.72
Himachal Pradesh	wasted	Birth weight	Below Average	−0.02	0.46	−0.90	0.89
Himachal Pradesh	wasted	Breastfed	No	−0.09	0.24	−0.58	0.39
Himachal Pradesh	wasted	Toilet	Unimproved	−0.20	0.33	−0.86	0.43
Himachal Pradesh	wasted	Toilet	Improved	0.05	0.51	−1.01	0.99
Himachal Pradesh	wasted	Location	Urban	0.39	0.41	−0.43	1.17
Himachal Pradesh	wasted	Cookign fuel	Solid	−0.14	0.32	−0.76	0.50
Himachal Pradesh	wasted	Household size	05-Oct	−0.15	0.28	−0.68	0.41
Himachal Pradesh	wasted	Household size	10+	−0.16	0.42	−1.01	0.64
Himachal Pradesh	wasted	LLIN	Yes	−3.23	3.39	−10.93	2.11
Himachal Pradesh	wasted	Access	Significant problem	−0.01	0.26	−0.53	0.49
Himachal Pradesh	wasted	Vaccination	-	0.00	4.98	−9.58	9.80

Name	Response	Group	Level	Estimate	Error	lci	uci
Jammu & Kashmir	ari	Intercept	-	−1.95	3.57	−8.99	5.13
Jammu & Kashmir	ari	Age	1-2	−0.08	0.43	−0.89	0.80
Jammu & Kashmir	ari	Age	2-3	−0.47	0.45	−1.33	0.42
Jammu & Kashmir	ari	Age	3-4	−0.53	0.47	−1.44	0.39
Jammu & Kashmir	ari	Age	4-5	−0.63	0.47	−1.56	0.29
Jammu & Kashmir	ari	Month	March	0.09	0.40	−0.68	0.89
Jammu & Kashmir	ari	Month	April	0.12	0.42	−0.70	0.96
Jammu & Kashmir	ari	Month	May	0.11	0.44	−0.75	0.97
Jammu & Kashmir	ari	Month	June	−0.28	0.68	−1.71	0.96
Jammu & Kashmir	ari	Month	July	−0.78	1.17	−3.35	1.18
Jammu & Kashmir	ari	Month	October	−2.00	1.86	−6.44	0.80
Jammu & Kashmir	ari	Month	November	−0.58	0.74	−2.10	0.80
Jammu & Kashmir	ari	Wealth	Poorer	0.07	0.55	−0.98	1.20
Jammu & Kashmir	ari	Wealth	Middle	0.06	0.57	−1.03	1.22
Jammu & Kashmir	ari	Wealth	Richer	−0.17	0.62	−1.35	1.06
Jammu & Kashmir	ari	Wealth	Richest	−0.49	0.70	−1.85	0.94
Jammu & Kashmir	ari	Mothers education	Primary	−0.18	0.61	−1.48	0.95
Jammu & Kashmir	ari	Mothers education	Secondary	0.03	0.33	−0.62	0.70
Jammu & Kashmir	ari	Mothers education	Higher	0.39	0.50	−0.60	1.37
Jammu & Kashmir	ari	Mothers age	20-30	−0.45	0.52	−1.41	0.61
Jammu & Kashmir	ari	Mothers age	30-40	−0.26	0.56	−1.32	0.87
Jammu & Kashmir	ari	Mothers age	40+	−1.42	1.51	−4.89	1.00
Jammu & Kashmir	ari	Sex	Female	−0.24	0.25	−0.75	0.25
Jammu & Kashmir	ari	Birth weight	Average	−0.04	0.44	−0.84	0.85
Jammu & Kashmir	ari	Birth weight	Below Average	0.38	0.54	−0.65	1.46
Jammu & Kashmir	ari	Breastfed	No	0.29	0.28	−0.27	0.84
Jammu & Kashmir	ari	Toilet	Unimproved	−0.37	0.33	−1.01	0.27
Jammu & Kashmir	ari	Toilet	Improved	−0.44	0.41	−1.28	0.32
Jammu & Kashmir	ari	Location	Urban	−0.68	0.42	−1.53	0.10
Jammu & Kashmir	ari	Cookign fuel	Solid	−0.20	0.35	−0.90	0.48
Jammu & Kashmir	ari	Household size	05-Oct	−0.26	0.30	−0.84	0.34
Jammu & Kashmir	ari	Household size	10+	−0.56	0.51	−1.61	0.39
Jammu & Kashmir	ari	LLIN	Yes	−0.01	2.85	−6.77	4.63
Jammu & Kashmir	ari	Access	Significant problem	0.54	0.28	−0.00	1.10
Jammu & Kashmir	ari	Vaccination	-	−0.03	4.95	−9.83	9.75

Name	Response	Group	Level	Estimate	Error	lci	uci
Jammu & Kashmir	dia	Intercept	-	−1.98	3.61	−9.02	4.98
Jammu & Kashmir	dia	Age	1-2	−0.16	0.33	−0.80	0.51
Jammu & Kashmir	dia	Age	2-3	−0.61	0.35	−1.29	0.06
Jammu & Kashmir	dia	Age	3-4	−1.25	0.41	−2.04	−0.46
Jammu & Kashmir	dia	Age	4-5	−1.03	0.39	−1.77	−0.26
Jammu & Kashmir	dia	Month	March	0.02	0.35	−0.66	0.72
Jammu & Kashmir	dia	Month	April	0.33	0.35	−0.33	1.03
Jammu & Kashmir	dia	Month	May	0.47	0.36	−0.23	1.18
Jammu & Kashmir	dia	Month	June	−0.05	0.56	−1.18	1.02
Jammu & Kashmir	dia	Month	July	0.32	0.73	−1.25	1.66
Jammu & Kashmir	dia	Month	October	−4.54	3.01	−11.71	−0.16
Jammu & Kashmir	dia	Month	November	−0.39	0.62	−1.65	0.77
Jammu & Kashmir	dia	Wealth	Poorer	−0.27	0.39	−1.02	0.48
Jammu & Kashmir	dia	Wealth	Middle	−0.84	0.44	−1.71	0.02
Jammu & Kashmir	dia	Wealth	Richer	−0.96	0.48	−1.91	−0.02
Jammu & Kashmir	dia	Wealth	Richest	−0.89	0.54	−1.95	0.19
Jammu & Kashmir	dia	Mothers education	Primary	−0.21	0.47	−1.19	0.67
Jammu & Kashmir	dia	Mothers education	Secondary	−0.14	0.27	−0.65	0.39
Jammu & Kashmir	dia	Mothers education	Higher	−0.21	0.44	−1.09	0.63
Jammu & Kashmir	dia	Mothers age	20-30	0.30	0.56	−0.72	1.48
Jammu & Kashmir	dia	Mothers age	30-40	0.17	0.59	−0.92	1.41
Jammu & Kashmir	dia	Mothers age	40+	0.24	0.94	−1.71	1.99
Jammu & Kashmir	dia	Sex	Female	0.02	0.21	−0.40	0.43
Jammu & Kashmir	dia	Birth weight	Average	0.32	0.40	−0.41	1.16
Jammu & Kashmir	dia	Birth weight	Below Average	0.53	0.49	−0.42	1.52
Jammu & Kashmir	dia	Breastfed	No	0.04	0.25	−0.45	0.52
Jammu & Kashmir	dia	Toilet	Unimproved	−0.18	0.28	−0.73	0.37
Jammu & Kashmir	dia	Toilet	Improved	−0.40	0.34	−1.09	0.23
Jammu & Kashmir	dia	Location	Urban	−0.37	0.33	−1.02	0.26
Jammu & Kashmir	dia	Cookign fuel	Solid	−0.34	0.31	−0.94	0.28
Jammu & Kashmir	dia	Household size	05-Oct	0.20	0.28	−0.33	0.76
Jammu & Kashmir	dia	Household size	10+	0.30	0.40	−0.51	1.06
Jammu & Kashmir	dia	LLIN	Yes	−0.28	2.64	−6.26	4.00
Jammu & Kashmir	dia	Access	Significant problem	0.37	0.24	−0.10	0.83
Jammu & Kashmir	dia	Vaccination	-	−0.00	5.05	−9.82	9.83

Name	Response	Group	Level	Estimate	Error	lci	uci
Jammu & Kashmir	fever	Intercept	-	−0.68	3.54	−7.66	6.15
Jammu & Kashmir	fever	Age	1-2	−0.07	0.28	−0.60	0.49
Jammu & Kashmir	fever	Age	2-3	−0.31	0.28	−0.88	0.25
Jammu & Kashmir	fever	Age	3-4	−0.57	0.31	−1.17	0.02
Jammu & Kashmir	fever	Age	4-5	−0.72	0.31	−1.33	−0.11
Jammu & Kashmir	fever	Month	March	−0.16	0.26	−0.67	0.34
Jammu & Kashmir	fever	Month	April	−0.02	0.27	−0.54	0.49
Jammu & Kashmir	fever	Month	May	−0.01	0.28	−0.56	0.53
Jammu & Kashmir	fever	Month	June	−0.55	0.44	−1.42	0.26
Jammu & Kashmir	fever	Month	July	0.03	0.58	−1.14	1.12
Jammu & Kashmir	fever	Month	October	−2.26	1.30	−5.28	−0.24
Jammu & Kashmir	fever	Month	November	−0.76	0.45	−1.69	0.09
Jammu & Kashmir	fever	Wealth	Poorer	−0.05	0.34	−0.72	0.64
Jammu & Kashmir	fever	Wealth	Middle	−0.17	0.36	−0.91	0.53
Jammu & Kashmir	fever	Wealth	Richer	−0.34	0.40	−1.13	0.42
Jammu & Kashmir	fever	Wealth	Richest	−0.64	0.45	−1.52	0.24
Jammu & Kashmir	fever	Mothers education	Primary	0.09	0.35	−0.63	0.75
Jammu & Kashmir	fever	Mothers education	Secondary	−0.15	0.21	−0.57	0.28
Jammu & Kashmir	fever	Mothers education	Higher	0.04	0.33	−0.61	0.69
Jammu & Kashmir	fever	Mothers age	20-30	−0.21	0.39	−0.96	0.60
Jammu & Kashmir	fever	Mothers age	30-40	−0.08	0.42	−0.87	0.78
Jammu & Kashmir	fever	Mothers age	40+	−0.18	0.73	−1.70	1.17
Jammu & Kashmir	fever	Sex	Female	−0.06	0.17	−0.38	0.26
Jammu & Kashmir	fever	Birth weight	Average	0.08	0.29	−0.46	0.67
Jammu & Kashmir	fever	Birth weight	Below Average	0.40	0.36	−0.29	1.12
Jammu & Kashmir	fever	Breastfed	No	−0.00	0.19	−0.38	0.37
Jammu & Kashmir	fever	Toilet	Unimproved	−0.28	0.21	−0.71	0.12
Jammu & Kashmir	fever	Toilet	Improved	−0.48	0.28	−1.03	0.05
Jammu & Kashmir	fever	Location	Urban	−0.49	0.26	−1.01	−0.00
Jammu & Kashmir	fever	Cookign fuel	Solid	−0.22	0.24	−0.67	0.25
Jammu & Kashmir	fever	Household size	05-Oct	0.10	0.21	−0.30	0.53
Jammu & Kashmir	fever	Household size	10+	−0.05	0.33	−0.70	0.59
Jammu & Kashmir	fever	LLIN	Yes	2.40	2.04	−1.63	6.75
Jammu & Kashmir	fever	Access	Significant problem	0.07	0.18	−0.29	0.43
Jammu & Kashmir	fever	Vaccination	-	−0.05	5.03	−9.63	9.82

Name	Response	Group	Level	Estimate	Error	lci	uci
Jammu & Kashmir	wasted	Intercept	-	−2.61	3.54	−9.61	4.35
Jammu & Kashmir	wasted	Age	1-2	−0.38	0.30	−0.96	0.21
Jammu & Kashmir	wasted	Age	2-3	−0.47	0.30	−1.06	0.12
Jammu & Kashmir	wasted	Age	3-4	−0.72	0.33	−1.37	−0.07
Jammu & Kashmir	wasted	Age	4-5	−0.54	0.31	−1.15	0.08
Jammu & Kashmir	wasted	Month	March	0.55	0.30	−0.02	1.14
Jammu & Kashmir	wasted	Month	April	0.15	0.33	−0.50	0.79
Jammu & Kashmir	wasted	Month	May	0.04	0.35	−0.65	0.73
Jammu & Kashmir	wasted	Month	June	0.09	0.50	−0.94	1.04
Jammu & Kashmir	wasted	Month	July	−0.09	0.78	−1.77	1.31
Jammu & Kashmir	wasted	Month	October	1.12	0.54	0.02	2.16
Jammu & Kashmir	wasted	Month	November	1.41	0.39	0.65	2.19
Jammu & Kashmir	wasted	Wealth	Poorer	−0.39	0.35	−1.07	0.29
Jammu & Kashmir	wasted	Wealth	Middle	−0.54	0.38	−1.27	0.21
Jammu & Kashmir	wasted	Wealth	Richer	−0.45	0.41	−1.26	0.38
Jammu & Kashmir	wasted	Wealth	Richest	−0.22	0.46	−1.12	0.70
Jammu & Kashmir	wasted	Mothers education	Primary	−0.03	0.36	−0.75	0.66
Jammu & Kashmir	wasted	Mothers education	Secondary	−0.45	0.22	−0.88	0.00
Jammu & Kashmir	wasted	Mothers education	Higher	−0.61	0.35	−1.32	0.06
Jammu & Kashmir	wasted	Mothers age	20-30	0.92	0.57	−0.08	2.12
Jammu & Kashmir	wasted	Mothers age	30-40	0.67	0.59	−0.39	1.91
Jammu & Kashmir	wasted	Mothers age	40+	0.56	0.89	−1.26	2.30
Jammu & Kashmir	wasted	Sex	Female	−0.02	0.17	−0.35	0.32
Jammu & Kashmir	wasted	Birth weight	Average	0.40	0.32	−0.20	1.08
Jammu & Kashmir	wasted	Birth weight	Below Average	0.36	0.41	−0.43	1.18
Jammu & Kashmir	wasted	Breastfed	No	−0.03	0.20	−0.43	0.37
Jammu & Kashmir	wasted	Toilet	Unimproved	0.06	0.22	−0.38	0.50
Jammu & Kashmir	wasted	Toilet	Improved	0.52	0.25	0.03	1.01
Jammu & Kashmir	wasted	Location	Urban	0.30	0.24	−0.18	0.77
Jammu & Kashmir	wasted	Cookign fuel	Solid	0.18	0.27	−0.35	0.70
Jammu & Kashmir	wasted	Household size	05-Oct	−0.11	0.21	−0.52	0.32
Jammu & Kashmir	wasted	Household size	10+	0.08	0.33	−0.57	0.72
Jammu & Kashmir	wasted	LLIN	Yes	−1.55	3.09	−8.66	3.33
Jammu & Kashmir	wasted	Access	Significant problem	−0.27	0.19	−0.64	0.11
Jammu & Kashmir	wasted	Vaccination	-	−0.01	4.99	−9.93	9.79

Name	Response	Group	Level	Estimate	Error	lci	uci
Jharkhand	ari	Intercept	-	−4.09	3.09	−10.06	2.01
Jharkhand	ari	Age	1-2	−0.19	0.28	−0.72	0.36
Jharkhand	ari	Age	2-3	−0.40	0.29	−0.96	0.17
Jharkhand	ari	Age	3-4	−0.56	0.30	−1.15	0.04
Jharkhand	ari	Age	4-5	−0.68	0.32	−1.30	−0.04
Jharkhand	ari	Month	May	−1.02	0.45	−1.92	−0.18
Jharkhand	ari	Month	June	−1.41	0.52	−2.50	−0.44
Jharkhand	ari	Month	July	−0.58	0.39	−1.35	0.16
Jharkhand	ari	Month	August	−0.88	0.37	−1.60	−0.17
Jharkhand	ari	Month	September	0.38	0.30	−0.19	0.98
Jharkhand	ari	Month	October	−0.01	0.32	−0.62	0.63
Jharkhand	ari	Month	November	−0.24	0.72	−1.74	1.07
Jharkhand	ari	Wealth	Poorer	0.16	0.23	−0.29	0.61
Jharkhand	ari	Wealth	Middle	0.40	0.29	−0.18	0.98
Jharkhand	ari	Wealth	Richer	0.66	0.42	−0.17	1.49
Jharkhand	ari	Wealth	Richest	−0.16	0.65	−1.47	1.09
Jharkhand	ari	Mothers education	Primary	0.09	0.26	−0.42	0.59
Jharkhand	ari	Mothers education	Secondary	−0.00	0.22	−0.43	0.42
Jharkhand	ari	Mothers education	Higher	0.25	0.42	−0.60	1.07
Jharkhand	ari	Mothers age	20-30	0.04	0.25	−0.43	0.55
Jharkhand	ari	Mothers age	30-40	−0.14	0.36	−0.83	0.55
Jharkhand	ari	Mothers age	40+	−0.36	1.02	−2.64	1.33
Jharkhand	ari	Sex	Female	−0.34	0.17	−0.66	−0.01
Jharkhand	ari	Birth weight	Average	0.45	0.29	−0.10	1.03
Jharkhand	ari	Birth weight	Below Average	1.21	0.35	0.54	1.91
Jharkhand	ari	Breastfed	No	−0.22	0.19	−0.60	0.15
Jharkhand	ari	Toilet	Unimproved	−0.07	0.27	−0.59	0.46
Jharkhand	ari	Toilet	Improved	0.03	0.20	−0.37	0.42
Jharkhand	ari	Location	Urban	−0.02	0.28	−0.58	0.51
Jharkhand	ari	Cookign fuel	Solid	−0.08	0.34	−0.74	0.61
Jharkhand	ari	Household size	05-Oct	0.15	0.21	−0.26	0.57
Jharkhand	ari	Household size	10+	0.25	0.29	−0.34	0.81
Jharkhand	ari	LLIN	Yes	−1.11	0.67	−2.56	0.06
Jharkhand	ari	Access	Significant problem	0.36	0.19	−0.00	0.73
Jharkhand	ari	Vaccination	-	0.02	4.98	−9.95	9.70

Name	Response	Group	Level	Estimate	Error	lci	uci
Jharkhand	dia	Intercept	-	−1.30	3.07	−7.28	4.71
Jharkhand	dia	Age	1-2	−0.17	0.17	−0.50	0.15
Jharkhand	dia	Age	2-3	−0.92	0.19	−1.28	−0.55
Jharkhand	dia	Age	3-4	−1.27	0.21	−1.68	−0.86
Jharkhand	dia	Age	4-5	−1.38	0.22	−1.81	−0.95
Jharkhand	dia	Month	May	−0.75	0.23	−1.21	−0.30
Jharkhand	dia	Month	June	−0.99	0.25	−1.51	−0.51
Jharkhand	dia	Month	July	−0.42	0.22	−0.85	0.00
Jharkhand	dia	Month	August	−1.08	0.23	−1.52	−0.64
Jharkhand	dia	Month	September	−1.19	0.22	−1.64	−0.76
Jharkhand	dia	Month	October	−1.33	0.24	−1.80	−0.88
Jharkhand	dia	Month	November	−0.68	0.48	−1.67	0.19
Jharkhand	dia	Wealth	Poorer	0.12	0.16	−0.19	0.44
Jharkhand	dia	Wealth	Middle	−0.01	0.23	−0.46	0.43
Jharkhand	dia	Wealth	Richer	0.02	0.35	−0.67	0.69
Jharkhand	dia	Wealth	Richest	−0.08	0.46	−0.99	0.81
Jharkhand	dia	Mothers education	Primary	0.04	0.18	−0.31	0.39
Jharkhand	dia	Mothers education	Secondary	−0.18	0.14	−0.46	0.10
Jharkhand	dia	Mothers education	Higher	0.10	0.29	−0.49	0.64
Jharkhand	dia	Mothers age	20-30	0.09	0.17	−0.24	0.44
Jharkhand	dia	Mothers age	30-40	−0.17	0.24	−0.64	0.30
Jharkhand	dia	Mothers age	40+	0.51	0.52	−0.60	1.47
Jharkhand	dia	Sex	Female	−0.14	0.11	−0.36	0.09
Jharkhand	dia	Birth weight	Average	−0.19	0.16	−0.51	0.13
Jharkhand	dia	Birth weight	Below Average	0.31	0.22	−0.12	0.74
Jharkhand	dia	Breastfed	No	−0.18	0.13	−0.44	0.08
Jharkhand	dia	Toilet	Unimproved	0.01	0.20	−0.38	0.40
Jharkhand	dia	Toilet	Improved	−0.07	0.14	−0.35	0.20
Jharkhand	dia	Location	Urban	−0.01	0.21	−0.42	0.41
Jharkhand	dia	Cookign fuel	Solid	−0.18	0.27	−0.71	0.37
Jharkhand	dia	Household size	05-Oct	0.08	0.14	−0.19	0.35
Jharkhand	dia	Household size	10+	0.06	0.20	−0.34	0.46
Jharkhand	dia	LLIN	Yes	−0.45	0.40	−1.28	0.30
Jharkhand	dia	Access	Significant problem	0.37	0.13	0.13	0.62
Jharkhand	dia	Vaccination	-	−0.03	5.00	−9.85	9.65

Name	Response	Group	Level	Estimate	Error	lci	uci
Jharkhand	fever	Intercept	-	−1.17	3.07	−7.25	4.69
Jharkhand	fever	Age	1-2	−0.19	0.14	−0.47	0.09
Jharkhand	fever	Age	2-3	−0.65	0.15	−0.95	−0.35
Jharkhand	fever	Age	3-4	−0.84	0.16	−1.15	−0.52
Jharkhand	fever	Age	4-5	−1.06	0.17	−1.40	−0.72
Jharkhand	fever	Month	May	−0.40	0.21	−0.80	0.01
Jharkhand	fever	Month	June	−1.15	0.25	−1.64	−0.66
Jharkhand	fever	Month	July	−0.19	0.20	−0.59	0.20
Jharkhand	fever	Month	August	−0.24	0.19	−0.62	0.13
Jharkhand	fever	Month	September	−0.16	0.18	−0.52	0.20
Jharkhand	fever	Month	October	−0.36	0.19	−0.73	0.01
Jharkhand	fever	Month	November	−0.82	0.48	−1.80	0.08
Jharkhand	fever	Wealth	Poorer	0.20	0.13	−0.05	0.45
Jharkhand	fever	Wealth	Middle	0.42	0.16	0.10	0.74
Jharkhand	fever	Wealth	Richer	0.56	0.24	0.09	1.02
Jharkhand	fever	Wealth	Richest	0.17	0.33	−0.46	0.81
Jharkhand	fever	Mothers education	Primary	0.01	0.14	−0.27	0.28
Jharkhand	fever	Mothers education	Secondary	−0.08	0.12	−0.31	0.15
Jharkhand	fever	Mothers education	Higher	−0.15	0.22	−0.60	0.28
Jharkhand	fever	Mothers age	20-30	−0.04	0.13	−0.29	0.22
Jharkhand	fever	Mothers age	30-40	−0.17	0.18	−0.52	0.18
Jharkhand	fever	Mothers age	40+	0.15	0.46	−0.80	1.01
Jharkhand	fever	Sex	Female	−0.16	0.09	−0.34	0.01
Jharkhand	fever	Birth weight	Average	−0.03	0.13	−0.29	0.23
Jharkhand	fever	Birth weight	Below Average	0.40	0.18	0.06	0.75
Jharkhand	fever	Breastfed	No	0.00	0.10	−0.19	0.20
Jharkhand	fever	Toilet	Unimproved	−0.16	0.14	−0.44	0.13
Jharkhand	fever	Toilet	Improved	0.06	0.11	−0.16	0.27
Jharkhand	fever	Location	Urban	0.15	0.15	−0.14	0.44
Jharkhand	fever	Cookign fuel	Solid	−0.17	0.19	−0.54	0.20
Jharkhand	fever	Household size	05-Oct	−0.02	0.11	−0.23	0.19
Jharkhand	fever	Household size	10+	−0.31	0.16	−0.64	0.01
Jharkhand	fever	LLIN	Yes	−0.37	0.28	−0.95	0.16
Jharkhand	fever	Access	Significant problem	0.17	0.10	−0.02	0.36
Jharkhand	fever	Vaccination	-	−0.01	5.01	−9.69	9.87

Name	Response	Group	Level	Estimate	Error	lci	uci
Jharkhand	wasted	Intercept	-	−0.83	3.07	−7.01	5.27
Jharkhand	wasted	Age	1-2	−0.24	0.11	−0.45	−0.02
Jharkhand	wasted	Age	2-3	−0.37	0.11	−0.59	−0.15
Jharkhand	wasted	Age	3-4	−0.67	0.12	−0.91	−0.43
Jharkhand	wasted	Age	4-5	−0.42	0.12	−0.66	−0.19
Jharkhand	wasted	Month	May	0.18	0.14	−0.09	0.45
Jharkhand	wasted	Month	June	0.34	0.14	0.07	0.61
Jharkhand	wasted	Month	July	0.56	0.13	0.29	0.82
Jharkhand	wasted	Month	August	0.25	0.13	−0.00	0.49
Jharkhand	wasted	Month	September	−0.01	0.12	−0.25	0.22
Jharkhand	wasted	Month	October	0.10	0.12	−0.14	0.35
Jharkhand	wasted	Month	November	−0.58	0.32	−1.22	0.04
Jharkhand	wasted	Wealth	Poorer	−0.19	0.09	−0.36	−0.01
Jharkhand	wasted	Wealth	Middle	−0.44	0.12	−0.68	−0.20
Jharkhand	wasted	Wealth	Richer	−0.09	0.19	−0.46	0.27
Jharkhand	wasted	Wealth	Richest	−0.37	0.26	−0.86	0.14
Jharkhand	wasted	Mothers education	Primary	−0.10	0.10	−0.30	0.09
Jharkhand	wasted	Mothers education	Secondary	−0.12	0.08	−0.28	0.04
Jharkhand	wasted	Mothers education	Higher	−0.01	0.17	−0.35	0.31
Jharkhand	wasted	Mothers age	20-30	0.03	0.10	−0.15	0.22
Jharkhand	wasted	Mothers age	30-40	0.10	0.13	−0.15	0.36
Jharkhand	wasted	Mothers age	40+	0.16	0.31	−0.44	0.75
Jharkhand	wasted	Sex	Female	−0.13	0.06	−0.26	−0.00
Jharkhand	wasted	Birth weight	Average	−0.01	0.09	−0.19	0.17
Jharkhand	wasted	Birth weight	Below Average	0.34	0.13	0.09	0.60
Jharkhand	wasted	Breastfed	No	−0.18	0.07	−0.32	−0.04
Jharkhand	wasted	Toilet	Unimproved	0.19	0.11	−0.02	0.41
Jharkhand	wasted	Toilet	Improved	0.04	0.08	−0.11	0.18
Jharkhand	wasted	Location	Urban	0.18	0.11	−0.04	0.39
Jharkhand	wasted	Cookign fuel	Solid	0.21	0.15	−0.09	0.51
Jharkhand	wasted	Household size	05-Oct	−0.11	0.08	−0.26	0.04
Jharkhand	wasted	Household size	10+	−0.05	0.11	−0.27	0.17
Jharkhand	wasted	LLIN	Yes	−0.16	0.20	−0.57	0.23
Jharkhand	wasted	Access	Significant problem	0.06	0.07	−0.08	0.19
Jharkhand	wasted	Vaccination	-	−0.01	5.03	−10.00	10.01

Name	Response	Group	Level	Estimate	Error	lci	uci
Karnataka	ari	Intercept	-	−9.60	3.57	−16.73	−2.72
Karnataka	ari	Age	1-2	−0.50	0.46	−1.38	0.40
Karnataka	ari	Age	2-3	−0.83	0.49	−1.79	0.15
Karnataka	ari	Age	3-4	−0.64	0.50	−1.63	0.33
Karnataka	ari	Age	4-5	−0.22	0.48	−1.16	0.74
Karnataka	ari	Month	March	3.04	1.64	0.11	6.50
Karnataka	ari	Month	April	0.80	1.71	−2.25	4.40
Karnataka	ari	Month	May	1.94	1.69	−1.12	5.45
Karnataka	ari	Month	June	1.90	1.68	−1.11	5.43
Karnataka	ari	Month	July	1.14	1.79	−2.12	4.88
Karnataka	ari	Wealth	Poorer	−0.04	0.72	−1.41	1.42
Karnataka	ari	Wealth	Middle	−0.05	0.73	−1.45	1.41
Karnataka	ari	Wealth	Richer	0.55	0.81	−1.02	2.16
Karnataka	ari	Wealth	Richest	−0.52	0.99	−2.48	1.39
Karnataka	ari	Mothers education	Primary	0.21	0.70	−1.16	1.62
Karnataka	ari	Mothers education	Secondary	0.29	0.56	−0.78	1.45
Karnataka	ari	Mothers education	Higher	1.06	0.70	−0.29	2.46
Karnataka	ari	Mothers age	20-30	0.25	0.47	−0.64	1.20
Karnataka	ari	Mothers age	30-40	0.09	0.62	−1.12	1.30
Karnataka	ari	Sex	Female	−0.18	0.28	−0.72	0.37
Karnataka	ari	Birth weight	Average	−0.64	0.32	−1.27	−0.02
Karnataka	ari	Birth weight	Below Average	−1.77	0.75	−3.37	−0.40
Karnataka	ari	Breastfed	No	−0.57	0.33	−1.23	0.05
Karnataka	ari	Toilet	Unimproved	−0.48	0.43	−1.35	0.36
Karnataka	ari	Toilet	Improved	1.02	0.55	−0.07	2.08
Karnataka	ari	Location	Urban	−0.28	0.52	−1.35	0.74
Karnataka	ari	Cookign fuel	Solid	0.43	0.44	−0.42	1.29
Karnataka	ari	Household size	05-Oct	−0.66	0.34	−1.34	0.00
Karnataka	ari	Household size	10+	−0.34	0.49	−1.30	0.61
Karnataka	ari	LLIN	Yes	1.33	0.58	0.19	2.46
Karnataka	ari	Access	Significant problem	0.99	0.36	0.31	1.70
Karnataka	ari	Vaccination	-	−0.00	4.97	−9.75	9.75

Name	Response	Group	Level	Estimate	Error	lci	uci
Arunachal Pradesh	ari	Intercept	-	−12.78	5.33	−23.59	−2.79
Arunachal Pradesh	ari	Age	1-2	1.58	2.45	−3.17	6.46
Arunachal Pradesh	ari	Age	2-3	−0.07	2.62	−5.39	5.05
Arunachal Pradesh	ari	Age	3-4	0.33	2.52	−4.67	5.26
Arunachal Pradesh	ari	Age	4-5	0.01	2.58	−5.10	5.04
Arunachal Pradesh	ari	Month	May	−0.09	2.81	−5.92	5.23
Arunachal Pradesh	ari	Month	June	−0.90	4.41	−10.12	7.14
Arunachal Pradesh	ari	Month	August	−0.60	2.96	−6.90	4.86
Arunachal Pradesh	ari	Month	September	1.48	2.64	−3.99	6.49
Arunachal Pradesh	ari	Month	October	1.39	2.51	−3.70	6.21
Arunachal Pradesh	ari	Month	November	1.86	2.49	−3.00	6.71
Arunachal Pradesh	ari	Wealth	Poorer	−0.04	2.29	−4.74	4.32
Arunachal Pradesh	ari	Wealth	Middle	0.93	2.28	−3.66	5.31
Arunachal Pradesh	ari	Wealth	Richer	0.13	2.81	−5.56	5.49
Arunachal Pradesh	ari	Wealth	Richest	−1.28	3.66	−8.83	5.47
Arunachal Pradesh	ari	Mothers education	Primary	−0.13	2.50	−5.47	4.42
Arunachal Pradesh	ari	Mothers education	Secondary	0.18	1.93	−3.56	4.08
Arunachal Pradesh	ari	Mothers education	Higher	−0.35	3.34	−7.43	5.78
Arunachal Pradesh	ari	Mothers age	20-30	1.85	2.48	−2.53	7.11
Arunachal Pradesh	ari	Mothers age	30-40	1.14	2.81	−4.39	6.79
Arunachal Pradesh	ari	Mothers age	40+	−0.48	4.18	−9.12	7.17
Arunachal Pradesh	ari	Sex	Female	0.29	1.64	−2.98	3.54
Arunachal Pradesh	ari	Birth weight	Average	1.94	2.44	−2.32	7.25
Arunachal Pradesh	ari	Birth weight	Below Average	−1.15	3.39	−8.23	5.13
Arunachal Pradesh	ari	Breastfed	No	1.56	1.78	−1.88	5.13
Arunachal Pradesh	ari	Toilet	Unimproved	1.46	1.92	−2.38	5.21
Arunachal Pradesh	ari	Toilet	Improved	0.11	2.18	−4.57	4.06
Arunachal Pradesh	ari	Location	Urban	−2.19	2.50	−7.67	2.30
Arunachal Pradesh	ari	Cookign fuel	Solid	−1.34	2.20	−5.67	2.92
Arunachal Pradesh	ari	Household size	05-Oct	−0.91	1.71	−4.27	2.50
Arunachal Pradesh	ari	Household size	10+	−1.02	3.27	−8.08	4.85
Arunachal Pradesh	ari	LLIN	Yes	−0.80	1.83	−4.60	2.59
Arunachal Pradesh	ari	Access	Significant problem	−0.56	1.70	−3.92	2.82
Arunachal Pradesh	ari	Vaccination	-	0.03	5.01	−9.98	9.71

Name	Response	Group	Level	Estimate	Error	lci	uci
Karnataka	dia	Intercept	-	−2.99	3.00	−8.89	2.83
Karnataka	dia	Age	1-2	−0.02	0.22	−0.45	0.40
Karnataka	dia	Age	2-3	−0.64	0.23	−1.10	−0.18
Karnataka	dia	Age	3-4	−1.30	0.26	−1.80	−0.79
Karnataka	dia	Age	4-5	−1.09	0.26	−1.61	−0.58
Karnataka	dia	Month	March	0.31	0.64	−0.89	1.59
Karnataka	dia	Month	April	−0.40	0.65	−1.65	0.92
Karnataka	dia	Month	May	−0.01	0.66	−1.26	1.31
Karnataka	dia	Month	June	−0.72	0.65	−1.96	0.60
Karnataka	dia	Month	July	−1.79	0.72	−3.19	−0.34
Karnataka	dia	Wealth	Poorer	0.21	0.33	−0.41	0.86
Karnataka	dia	Wealth	Middle	0.05	0.33	−0.59	0.71
Karnataka	dia	Wealth	Richer	0.35	0.40	−0.41	1.14
Karnataka	dia	Wealth	Richest	0.24	0.46	−0.64	1.15
Karnataka	dia	Mothers education	Primary	−0.45	0.29	−1.03	0.10
Karnataka	dia	Mothers education	Secondary	−0.38	0.21	−0.80	0.03
Karnataka	dia	Mothers education	Higher	−1.65	0.36	−2.37	−0.95
Karnataka	dia	Mothers age	20-30	0.22	0.24	−0.25	0.71
Karnataka	dia	Mothers age	30-40	−0.48	0.34	−1.16	0.18
Karnataka	dia	Sex	Female	−0.19	0.14	−0.46	0.07
Karnataka	dia	Birth weight	Average	−0.14	0.16	−0.46	0.17
Karnataka	dia	Birth weight	Below Average	−0.67	0.33	−1.35	−0.04
Karnataka	dia	Breastfed	No	−0.09	0.16	−0.40	0.22
Karnataka	dia	Toilet	Unimproved	−0.07	0.20	−0.46	0.33
Karnataka	dia	Toilet	Improved	−0.45	0.38	−1.22	0.26
Karnataka	dia	Location	Urban	0.12	0.24	−0.34	0.59
Karnataka	dia	Cookign fuel	Solid	0.20	0.23	−0.25	0.65
Karnataka	dia	Household size	05-Oct	−0.28	0.16	−0.60	0.04
Karnataka	dia	Household size	10+	−0.55	0.26	−1.06	−0.05
Karnataka	dia	LLIN	Yes	0.72	0.30	0.11	1.31
Karnataka	dia	Access	Significant problem	0.38	0.17	0.04	0.71
Karnataka	dia	Vaccination	-	−0.05	4.99	−9.78	9.67

Name	Response	Group	Level	Estimate	Error	lci	uci
Karnataka	fever	Intercept	-	−1.81	2.97	−7.70	4.03
Karnataka	fever	Age	1-2	0.12	0.19	−0.25	0.47
Karnataka	fever	Age	2-3	−0.20	0.20	−0.59	0.17
Karnataka	fever	Age	3-4	−0.15	0.20	−0.54	0.24
Karnataka	fever	Age	4-5	−0.21	0.20	−0.61	0.19
Karnataka	fever	Month	March	0.31	0.43	−0.53	1.16
Karnataka	fever	Month	April	−0.92	0.46	−1.80	0.01
Karnataka	fever	Month	May	−0.71	0.46	−1.62	0.21
Karnataka	fever	Month	June	−0.60	0.46	−1.48	0.29
Karnataka	fever	Month	July	−1.19	0.50	−2.15	−0.20
Karnataka	fever	Wealth	Poorer	−0.91	0.25	−1.40	−0.41
Karnataka	fever	Wealth	Middle	−0.48	0.25	−0.97	0.02
Karnataka	fever	Wealth	Richer	−0.30	0.30	−0.89	0.28
Karnataka	fever	Wealth	Richest	−0.41	0.35	−1.09	0.27
Karnataka	fever	Mothers education	Primary	0.02	0.25	−0.46	0.50
Karnataka	fever	Mothers education	Secondary	0.29	0.20	−0.09	0.69
Karnataka	fever	Mothers education	Higher	0.17	0.28	−0.36	0.71
Karnataka	fever	Mothers age	20-30	−0.27	0.17	−0.60	0.07
Karnataka	fever	Mothers age	30-40	−0.29	0.24	−0.76	0.17
Karnataka	fever	Sex	Female	−0.20	0.11	−0.41	0.01
Karnataka	fever	Birth weight	Average	−0.26	0.12	−0.49	−0.03
Karnataka	fever	Birth weight	Below Average	0.22	0.20	−0.18	0.63
Karnataka	fever	Breastfed	No	−0.49	0.12	−0.74	−0.25
Karnataka	fever	Toilet	Unimproved	−0.35	0.16	−0.66	−0.04
Karnataka	fever	Toilet	Improved	0.55	0.22	0.10	0.98
Karnataka	fever	Location	Urban	−0.24	0.18	−0.59	0.13
Karnataka	fever	Cookign fuel	Solid	0.40	0.16	0.08	0.71
Karnataka	fever	Household size	05-Oct	−0.07	0.14	−0.33	0.19
Karnataka	fever	Household size	10+	−0.07	0.19	−0.45	0.31
Karnataka	fever	LLIN	Yes	0.82	0.23	0.36	1.26
Karnataka	fever	Access	Significant problem	0.06	0.13	−0.20	0.31
Karnataka	fever	Vaccination	-	−0.02	5.00	−9.84	9.87

Name	Response	Group	Level	Estimate	Error	lci	uci
Karnataka	wasted	Intercept	-	−1.60	2.89	−7.07	4.01
Karnataka	wasted	Age	1-2	−0.19	0.11	−0.41	0.04
Karnataka	wasted	Age	2-3	−0.13	0.11	−0.35	0.10
Karnataka	wasted	Age	3-4	−0.19	0.12	−0.44	0.04
Karnataka	wasted	Age	4-5	0.11	0.12	−0.12	0.34
Karnataka	wasted	Month	March	0.08	0.33	−0.55	0.75
Karnataka	wasted	Month	April	0.36	0.33	−0.28	1.01
Karnataka	wasted	Month	May	0.60	0.33	−0.05	1.27
Karnataka	wasted	Month	June	0.80	0.33	0.17	1.47
Karnataka	wasted	Month	July	0.82	0.34	0.16	1.51
Karnataka	wasted	Wealth	Poorer	−0.02	0.14	−0.30	0.27
Karnataka	wasted	Wealth	Middle	−0.11	0.15	−0.40	0.19
Karnataka	wasted	Wealth	Richer	−0.02	0.19	−0.38	0.34
Karnataka	wasted	Wealth	Richest	0.03	0.21	−0.39	0.45
Karnataka	wasted	Mothers education	Primary	−0.26	0.12	−0.50	−0.01
Karnataka	wasted	Mothers education	Secondary	−0.17	0.10	−0.37	0.02
Karnataka	wasted	Mothers education	Higher	−0.67	0.15	−0.97	−0.37
Karnataka	wasted	Mothers age	20-30	0.01	0.11	−0.21	0.22
Karnataka	wasted	Mothers age	30-40	−0.03	0.15	−0.33	0.26
Karnataka	wasted	Sex	Female	−0.08	0.06	−0.20	0.04
Karnataka	wasted	Birth weight	Average	−0.14	0.07	−0.28	0.00
Karnataka	wasted	Birth weight	Below Average	−0.09	0.13	−0.35	0.17
Karnataka	wasted	Breastfed	No	−0.12	0.07	−0.26	0.02
Karnataka	wasted	Toilet	Unimproved	−0.08	0.10	−0.27	0.12
Karnataka	wasted	Toilet	Improved	−0.09	0.17	−0.42	0.24
Karnataka	wasted	Location	Urban	0.10	0.11	−0.11	0.32
Karnataka	wasted	Cookign fuel	Solid	0.19	0.10	−0.01	0.40
Karnataka	wasted	Household size	05-Oct	0.08	0.08	−0.08	0.23
Karnataka	wasted	Household size	10+	−0.17	0.12	−0.40	0.06
Karnataka	wasted	LLIN	Yes	0.03	0.18	−0.31	0.39
Karnataka	wasted	Access	Significant problem	0.10	0.08	−0.05	0.26
Karnataka	wasted	Vaccination	-	0.06	4.93	−9.45	9.57

Name	Response	Group	Level	Estimate	Error	lci	uci
Kenya	ari	Intercept	-	−2.05	0.33	−2.71	−1.39
Kenya	ari	Age	1-2	−0.11	0.10	−0.30	0.08
Kenya	ari	Age	2-3	−0.25	0.10	−0.45	−0.06
Kenya	ari	Age	3-4	−0.37	0.10	−0.58	−0.17
Kenya	ari	Age	4-5	−0.39	0.11	−0.59	−0.18
Kenya	ari	Month	June	−0.07	0.13	−0.33	0.19
Kenya	ari	Month	July	0.09	0.13	−0.16	0.34
Kenya	ari	Month	August	0.11	0.13	−0.14	0.36
Kenya	ari	Month	September	−0.36	0.14	−0.62	−0.09
Kenya	ari	Month	October	−1.13	0.26	−1.65	−0.64
Kenya	ari	Wealth	Poorer	−0.09	0.10	−0.28	0.10
Kenya	ari	Wealth	Middle	0.06	0.10	−0.15	0.26
Kenya	ari	Wealth	Richer	−0.12	0.12	−0.35	0.12
Kenya	ari	Wealth	Richest	−0.38	0.15	−0.68	−0.07
Kenya	ari	Mothers education	Primary	0.36	0.13	0.12	0.61
Kenya	ari	Mothers education	Secondary	0.24	0.14	−0.04	0.52
Kenya	ari	Mothers education	Higher	0.20	0.18	−0.15	0.57
Kenya	ari	Mothers age	20-30	−0.05	0.09	−0.24	0.13
Kenya	ari	Mothers age	30-40	−0.14	0.11	−0.35	0.07
Kenya	ari	Mothers age	40+	−0.08	0.19	−0.45	0.29
Kenya	ari	Sex	Female	−0.05	0.06	−0.16	0.07
Kenya	ari	Breastfed	No	−0.11	0.06	−0.24	0.02
Kenya	ari	Toilet	Unimproved	0.01	0.07	−0.14	0.15
Kenya	ari	Toilet	Improved	−0.09	0.08	−0.24	0.06
Kenya	ari	Location	Urban	−0.04	0.10	−0.24	0.15
Kenya	ari	Cookign fuel	Solid	−0.24	0.16	−0.55	0.07
Kenya	ari	Household size	05-Oct	0.01	0.07	−0.12	0.15
Kenya	ari	Household size	10+	−0.12	0.13	−0.38	0.14
Kenya	ari	LLIN	Yes	−0.06	0.06	−0.19	0.06
Kenya	ari	Vaccination	-	0.13	0.35	−0.52	0.85

Name	Response	Group	Level	Estimate	Error	lci	uci
Kenya	dia	Intercept	-	−0.21	0.28	−0.77	0.32
Kenya	dia	Age	1-2	−0.14	0.07	−0.28	0.01
Kenya	dia	Age	2-3	−0.67	0.08	−0.82	−0.51
Kenya	dia	Age	3-4	−1.34	0.09	−1.51	−1.18
Kenya	dia	Age	4-5	−1.70	0.09	−1.89	−1.51
Kenya	dia	Month	June	−0.15	0.11	−0.36	0.07
Kenya	dia	Month	July	−0.24	0.11	−0.46	−0.01
Kenya	dia	Month	August	−0.20	0.11	−0.42	0.02
Kenya	dia	Month	September	−0.49	0.11	−0.71	−0.26
Kenya	dia	Month	October	−0.62	0.18	−0.97	−0.26
Kenya	dia	Wealth	Poorer	0.02	0.08	−0.14	0.17
Kenya	dia	Wealth	Middle	−0.18	0.09	−0.35	0.00
Kenya	dia	Wealth	Richer	−0.16	0.10	−0.36	0.04
Kenya	dia	Wealth	Richest	−0.67	0.13	−0.93	−0.41
Kenya	dia	Mothers education	Primary	0.04	0.09	−0.15	0.22
Kenya	dia	Mothers education	Secondary	0.03	0.11	−0.19	0.24
Kenya	dia	Mothers education	Higher	−0.45	0.15	−0.75	−0.16
Kenya	dia	Mothers age	20-30	−0.24	0.08	−0.38	−0.09
Kenya	dia	Mothers age	30-40	−0.32	0.09	−0.48	−0.14
Kenya	dia	Mothers age	40+	−0.30	0.15	−0.60	0.01
Kenya	dia	Sex	Female	−0.15	0.05	−0.24	−0.06
Kenya	dia	Breastfed	No	−0.01	0.05	−0.11	0.09
Kenya	dia	Toilet	Unimproved	0.03	0.06	−0.09	0.15
Kenya	dia	Toilet	Improved	0.05	0.06	−0.08	0.17
Kenya	dia	Location	Urban	0.21	0.08	0.04	0.37
Kenya	dia	Cookign fuel	Solid	−0.10	0.14	−0.38	0.17
Kenya	dia	Household size	05-Oct	−0.20	0.06	−0.31	−0.08
Kenya	dia	Household size	10+	−0.30	0.11	−0.53	−0.08
Kenya	dia	LLIN	Yes	−0.03	0.05	−0.14	0.07
Kenya	dia	Vaccination	-	−0.17	0.30	−0.74	0.43

Name	Response	Group	Level	Estimate	Error	lci	uci
Kenya	fever	Intercept	-	−0.79	0.25	−1.27	−0.32
Kenya	fever	Age	1-2	−0.04	0.07	−0.17	0.10
Kenya	fever	Age	2-3	−0.32	0.07	−0.46	−0.18
Kenya	fever	Age	3-4	−0.49	0.07	−0.63	−0.35
Kenya	fever	Age	4-5	−0.55	0.07	−0.69	−0.40
Kenya	fever	Month	June	−0.02	0.10	−0.21	0.19
Kenya	fever	Month	July	−0.08	0.10	−0.28	0.12
Kenya	fever	Month	August	−0.20	0.10	−0.39	0.01
Kenya	fever	Month	September	−0.69	0.10	−0.89	−0.48
Kenya	fever	Month	October	−0.73	0.17	−1.05	−0.41
Kenya	fever	Wealth	Poorer	0.03	0.07	−0.11	0.16
Kenya	fever	Wealth	Middle	−0.03	0.08	−0.18	0.11
Kenya	fever	Wealth	Richer	−0.06	0.09	−0.22	0.11
Kenya	fever	Wealth	Richest	−0.27	0.11	−0.48	−0.06
Kenya	fever	Mothers education	Primary	0.47	0.09	0.30	0.65
Kenya	fever	Mothers education	Secondary	0.40	0.10	0.20	0.59
Kenya	fever	Mothers education	Higher	0.41	0.13	0.17	0.66
Kenya	fever	Mothers age	20-30	0.01	0.07	−0.12	0.13
Kenya	fever	Mothers age	30-40	0.07	0.08	−0.08	0.21
Kenya	fever	Mothers age	40+	0.13	0.13	−0.12	0.38
Kenya	fever	Sex	Female	−0.03	0.04	−0.11	0.05
Kenya	fever	Breastfed	No	−0.03	0.04	−0.12	0.06
Kenya	fever	Toilet	Unimproved	0.00	0.05	−0.10	0.10
Kenya	fever	Toilet	Improved	0.01	0.06	−0.09	0.12
Kenya	fever	Location	Urban	−0.06	0.08	−0.20	0.09
Kenya	fever	Cookign fuel	Solid	0.29	0.11	0.06	0.51
Kenya	fever	Household size	05-Oct	−0.07	0.05	−0.17	0.02
Kenya	fever	Household size	10+	−0.09	0.09	−0.27	0.09
Kenya	fever	LLIN	Yes	−0.00	0.05	−0.09	0.09
Kenya	fever	Vaccination	-	−0.66	0.27	−1.19	−0.13

Name	Response	Group	Level	Estimate	Error	lci	uci
Kenya	wasted	Intercept	-	−0.73	0.47	−1.63	0.20
Kenya	wasted	Age	1-2	−0.34	0.13	−0.59	−0.07
Kenya	wasted	Age	2-3	−0.92	0.14	−1.21	−0.64
Kenya	wasted	Age	3-4	−0.92	0.15	−1.20	−0.63
Kenya	wasted	Age	4-5	−0.77	0.14	−1.05	−0.49
Kenya	wasted	Month	June	−0.27	0.20	−0.66	0.12
Kenya	wasted	Month	July	−0.10	0.19	−0.46	0.27
Kenya	wasted	Month	August	−0.12	0.19	−0.48	0.24
Kenya	wasted	Month	September	−0.09	0.18	−0.45	0.28
Kenya	wasted	Month	October	0.23	0.26	−0.30	0.73
Kenya	wasted	Wealth	Poorer	−0.43	0.15	−0.72	−0.15
Kenya	wasted	Wealth	Middle	−0.28	0.16	−0.59	0.02
Kenya	wasted	Wealth	Richer	−0.80	0.19	−1.19	−0.43
Kenya	wasted	Wealth	Richest	−0.91	0.23	−1.37	−0.46
Kenya	wasted	Mothers education	Primary	−0.60	0.14	−0.87	−0.33
Kenya	wasted	Mothers education	Secondary	−0.56	0.17	−0.90	−0.22
Kenya	wasted	Mothers education	Higher	−1.19	0.29	−1.77	−0.63
Kenya	wasted	Mothers age	20-30	0.17	0.15	−0.12	0.47
Kenya	wasted	Mothers age	30-40	0.16	0.17	−0.17	0.48
Kenya	wasted	Mothers age	40+	0.14	0.28	−0.41	0.68
Kenya	wasted	Sex	Female	−0.16	0.09	−0.33	0.01
Kenya	wasted	Breastfed	No	0.19	0.10	−0.00	0.38
Kenya	wasted	Toilet	Unimproved	−0.13	0.11	−0.36	0.08
Kenya	wasted	Toilet	Improved	0.03	0.11	−0.19	0.25
Kenya	wasted	Location	Urban	0.16	0.14	−0.12	0.44
Kenya	wasted	Cookign fuel	Solid	−0.18	0.27	−0.70	0.35
Kenya	wasted	Household size	05-Oct	0.05	0.11	−0.15	0.26
Kenya	wasted	Household size	10+	−0.09	0.20	−0.47	0.30
Kenya	wasted	LLIN	Yes	−0.21	0.10	−0.40	−0.02
Kenya	wasted	Vaccination	-	−1.76	0.44	−2.63	−0.92

Name	Response	Group	Level	Estimate	Error	lci	uci
Kerala	ari	Intercept	-	−23.85	7.28	−38.42	−10.16
Kerala	ari	Age	1-2	−4.22	1.76	−7.73	−0.86
Kerala	ari	Age	2-3	0.67	1.45	−2.04	3.60
Kerala	ari	Age	3-4	2.82	1.55	−0.07	6.03
Kerala	ari	Age	4-5	0.54	1.61	−2.56	3.77
Kerala	ari	Month	April	0.66	2.21	−3.69	4.92
Kerala	ari	Month	May	−0.68	2.41	−5.46	4.07
Kerala	ari	Month	June	−1.22	2.54	−6.33	3.65
Kerala	ari	Month	July	1.93	2.65	−3.34	7.26
Kerala	ari	Wealth	Middle	5.24	2.45	0.54	10.12
Kerala	ari	Wealth	Richer	2.12	2.46	−2.63	6.95
Kerala	ari	Wealth	Richest	2.90	2.59	−2.10	7.97
Kerala	ari	Mothers education	Secondary	0.76	3.22	−5.29	7.21
Kerala	ari	Mothers education	Higher	0.87	3.25	−5.39	7.28
Kerala	ari	Mothers age	20-30	−0.16	1.33	−2.75	2.49
Kerala	ari	Mothers age	30-40	2.28	1.45	−0.40	5.33
Kerala	ari	Sex	Female	−0.85	0.81	−2.43	0.75
Kerala	ari	Birth weight	Average	−0.65	0.95	−2.55	1.21
Kerala	ari	Birth weight	Below Average	0.74	1.40	−2.04	3.43
Kerala	ari	Breastfed	No	−1.15	0.84	−2.86	0.47
Kerala	ari	Toilet	Unimproved	−2.44	2.96	−8.30	3.36
Kerala	ari	Toilet	Improved	3.78	1.58	0.74	6.94
Kerala	ari	Location	Urban	−1.03	1.97	−5.04	2.82
Kerala	ari	Cookign fuel	Solid	0.92	1.01	−1.03	2.94
Kerala	ari	Household size	05-Oct	−1.52	0.90	−3.33	0.23
Kerala	ari	Household size	10+	1.80	1.98	−2.17	5.59
Kerala	ari	LLIN	Yes	−2.36	3.89	−10.60	4.49
Kerala	ari	Access	Significant problem	3.67	1.71	0.48	7.25
Kerala	ari	Vaccination	-	−0.01	4.94	−9.81	9.79

Name	Response	Group	Level	Estimate	Error	lci	uci
Kerala	dia	Intercept	-	−3.80	4.57	−12.90	5.04
Kerala	dia	Age	1-2	−0.37	0.43	−1.22	0.48
Kerala	dia	Age	2-3	−1.92	0.45	−2.81	−1.05
Kerala	dia	Age	3-4	−3.02	0.59	−4.22	−1.89
Kerala	dia	Age	4-5	−2.66	0.54	−3.74	−1.62
Kerala	dia	Month	April	0.32	0.84	−1.30	1.99
Kerala	dia	Month	May	−0.41	0.89	−2.17	1.32
Kerala	dia	Month	June	0.71	0.82	−0.87	2.39
Kerala	dia	Month	July	1.55	0.94	−0.28	3.45
Kerala	dia	Wealth	Middle	0.65	1.12	−1.48	2.91
Kerala	dia	Wealth	Richer	0.40	1.11	−1.68	2.65
Kerala	dia	Wealth	Richest	−1.06	1.17	−3.30	1.27
Kerala	dia	Mothers education	Secondary	0.31	1.40	−2.19	3.25
Kerala	dia	Mothers education	Higher	1.07	1.43	−1.45	4.07
Kerala	dia	Mothers age	20-30	−2.20	0.57	−3.33	−1.13
Kerala	dia	Mothers age	30-40	−2.52	0.65	−3.84	−1.29
Kerala	dia	Sex	Female	−0.30	0.29	−0.87	0.25
Kerala	dia	Birth weight	Average	0.15	0.38	−0.57	0.93
Kerala	dia	Birth weight	Below Average	0.63	0.59	−0.53	1.79
Kerala	dia	Breastfed	No	0.27	0.39	−0.50	1.03
Kerala	dia	Toilet	Unimproved	3.07	1.39	0.27	5.78
Kerala	dia	Toilet	Improved	1.31	0.59	0.14	2.45
Kerala	dia	Location	Urban	−1.04	0.62	−2.34	0.13
Kerala	dia	Cookign fuel	Solid	−0.25	0.36	−0.96	0.49
Kerala	dia	Household size	05-Oct	0.00	0.35	−0.66	0.68
Kerala	dia	Household size	10+	0.44	0.62	−0.80	1.65
Kerala	dia	LLIN	Yes	−0.05	0.63	−1.30	1.14
Kerala	dia	Access	Significant problem	−0.19	0.63	−1.46	1.01
Kerala	dia	Vaccination	-	−0.05	5.04	−9.86	9.93

Name	Response	Group	Level	Estimate	Error	lci	uci
Kerala	fever	Intercept	-	−0.65	4.24	−8.97	7.71
Kerala	fever	Age	1-2	0.90	0.29	0.34	1.48
Kerala	fever	Age	2-3	0.14	0.29	−0.43	0.70
Kerala	fever	Age	3-4	0.11	0.30	−0.48	0.70
Kerala	fever	Age	4-5	0.13	0.31	−0.46	0.73
Kerala	fever	Month	April	−0.70	0.36	−1.41	0.00
Kerala	fever	Month	May	−1.17	0.37	−1.91	−0.46
Kerala	fever	Month	June	−1.52	0.39	−2.29	−0.79
Kerala	fever	Month	July	−1.05	0.47	−1.98	−0.15
Kerala	fever	Wealth	Middle	0.25	0.64	−0.97	1.52
Kerala	fever	Wealth	Richer	−0.49	0.63	−1.74	0.75
Kerala	fever	Wealth	Richest	−0.95	0.66	−2.24	0.36
Kerala	fever	Mothers education	Secondary	−1.77	0.65	−3.02	−0.47
Kerala	fever	Mothers education	Higher	−2.04	0.67	−3.32	−0.73
Kerala	fever	Mothers age	20-30	0.78	0.43	−0.04	1.65
Kerala	fever	Mothers age	30-40	0.65	0.46	−0.20	1.55
Kerala	fever	Sex	Female	0.09	0.15	−0.19	0.37
Kerala	fever	Birth weight	Average	−0.32	0.19	−0.68	0.05
Kerala	fever	Birth weight	Below Average	0.33	0.29	−0.24	0.88
Kerala	fever	Breastfed	No	−0.39	0.19	−0.77	−0.01
Kerala	fever	Toilet	Unimproved	−2.86	1.51	−6.24	−0.30
Kerala	fever	Toilet	Improved	0.54	0.30	−0.07	1.13
Kerala	fever	Location	Urban	−0.11	0.27	−0.63	0.42
Kerala	fever	Cookign fuel	Solid	−0.24	0.20	−0.63	0.13
Kerala	fever	Household size	05-Oct	−0.03	0.17	−0.34	0.30
Kerala	fever	Household size	10+	0.11	0.34	−0.56	0.76
Kerala	fever	LLIN	Yes	0.56	0.41	−0.27	1.36
Kerala	fever	Access	Significant problem	0.22	0.35	−0.47	0.88
Kerala	fever	Vaccination	-	0.03	5.04	−9.96	9.95

Name	Response	Group	Level	Estimate	Error	lci	uci
Arunachal Pradesh	dia	Intercept	-	−4.00	3.25	−10.54	2.14
Arunachal Pradesh	dia	Age	1-2	−0.04	1.46	−2.92	2.90
Arunachal Pradesh	dia	Age	2-3	−0.47	1.48	−3.34	2.49
Arunachal Pradesh	dia	Age	3-4	−0.76	1.53	−3.76	2.32
Arunachal Pradesh	dia	Age	4-5	−0.98	1.54	−3.97	2.14
Arunachal Pradesh	dia	Month	May	0.14	1.33	−2.42	2.79
Arunachal Pradesh	dia	Month	June	−0.52	3.64	−8.28	5.94
Arunachal Pradesh	dia	Month	August	−0.52	1.54	−3.69	2.42
Arunachal Pradesh	dia	Month	September	−0.41	1.62	−3.72	2.69
Arunachal Pradesh	dia	Month	October	−0.80	1.50	−3.77	2.18
Arunachal Pradesh	dia	Month	November	−0.65	1.62	−3.96	2.51
Arunachal Pradesh	dia	Wealth	Poorer	0.33	1.28	−2.22	2.90
Arunachal Pradesh	dia	Wealth	Middle	−0.49	1.51	−3.51	2.42
Arunachal Pradesh	dia	Wealth	Richer	−0.11	1.78	−3.64	3.38
Arunachal Pradesh	dia	Wealth	Richest	−1.84	2.43	−6.92	2.68
Arunachal Pradesh	dia	Mothers education	Primary	0.44	1.40	−2.42	3.12
Arunachal Pradesh	dia	Mothers education	Secondary	0.54	1.13	−1.58	2.85
Arunachal Pradesh	dia	Mothers education	Higher	0.53	1.95	−3.67	4.06
Arunachal Pradesh	dia	Mothers age	20-30	0.54	1.32	−1.85	3.43
Arunachal Pradesh	dia	Mothers age	30-40	0.04	1.58	−3.03	3.27
Arunachal Pradesh	dia	Mothers age	40+	0.29	2.89	−5.97	5.31
Arunachal Pradesh	dia	Sex	Female	0.05	0.83	−1.63	1.67
Arunachal Pradesh	dia	Birth weight	Average	0.15	1.10	−1.89	2.48
Arunachal Pradesh	dia	Birth weight	Below Average	0.22	1.45	−2.65	3.07
Arunachal Pradesh	dia	Breastfed	No	0.68	0.93	−1.14	2.51
Arunachal Pradesh	dia	Toilet	Unimproved	0.46	1.06	−1.69	2.54
Arunachal Pradesh	dia	Toilet	Improved	−0.41	1.31	−3.29	1.92
Arunachal Pradesh	dia	Location	Urban	−0.17	1.13	−2.47	1.96
Arunachal Pradesh	dia	Cookign fuel	Solid	−0.77	1.33	−3.43	1.85
Arunachal Pradesh	dia	Household size	05-Oct	−0.14	0.92	−1.90	1.70
Arunachal Pradesh	dia	Household size	10+	1.12	1.73	−2.50	4.31
Arunachal Pradesh	dia	LLIN	Yes	0.04	0.98	−1.96	1.91
Arunachal Pradesh	dia	Access	Significant problem	−0.01	0.91	−1.75	1.80
Arunachal Pradesh	dia	Vaccination	-	−0.03	4.99	−9.71	9.68

Name	Response	Group	Level	Estimate	Error	lci	uci
Kerala	wasted	Intercept	-	−3.21	4.18	−11.41	5.02
Kerala	wasted	Age	1-2	−0.96	0.23	−1.41	−0.52
Kerala	wasted	Age	2-3	−0.60	0.22	−1.03	−0.18
Kerala	wasted	Age	3-4	−1.26	0.24	−1.72	−0.80
Kerala	wasted	Age	4-5	−1.12	0.24	−1.58	−0.65
Kerala	wasted	Month	April	0.08	0.32	−0.54	0.72
Kerala	wasted	Month	May	0.42	0.32	−0.23	1.05
Kerala	wasted	Month	June	−0.19	0.34	−0.85	0.48
Kerala	wasted	Month	July	−0.82	0.45	−1.70	0.03
Kerala	wasted	Wealth	Middle	0.23	0.53	−0.78	1.32
Kerala	wasted	Wealth	Richer	−0.48	0.52	−1.47	0.58
Kerala	wasted	Wealth	Richest	−0.50	0.54	−1.54	0.60
Kerala	wasted	Mothers education	Secondary	1.33	1.07	−0.56	3.61
Kerala	wasted	Mothers education	Higher	0.91	1.08	−1.02	3.20
Kerala	wasted	Mothers age	20-30	0.36	0.32	−0.25	0.99
Kerala	wasted	Mothers age	30-40	1.21	0.34	0.56	1.90
Kerala	wasted	Sex	Female	0.06	0.13	−0.18	0.32
Kerala	wasted	Birth weight	Average	0.06	0.19	−0.31	0.43
Kerala	wasted	Birth weight	Below Average	1.23	0.27	0.73	1.75
Kerala	wasted	Breastfed	No	0.08	0.17	−0.25	0.41
Kerala	wasted	Toilet	Unimproved	0.03	0.82	−1.65	1.55
Kerala	wasted	Toilet	Improved	0.58	0.26	0.06	1.08
Kerala	wasted	Location	Urban	−0.12	0.23	−0.58	0.34
Kerala	wasted	Cookign fuel	Solid	−0.49	0.16	−0.82	−0.18
Kerala	wasted	Household size	05-Oct	0.41	0.16	0.11	0.73
Kerala	wasted	Household size	10+	0.38	0.32	−0.27	1.00
Kerala	wasted	LLIN	Yes	0.28	0.42	−0.58	1.10
Kerala	wasted	Access	Significant problem	−0.18	0.32	−0.80	0.45
Kerala	wasted	Vaccination	-	−0.04	4.94	−9.74	9.76

Name	Response	Group	Level	Estimate	Error	lci	uci
Lakshadweep	ari	Intercept	-	−16.43	11.55	−39.25	5.56
Lakshadweep	ari	Age	1-2	0.30	4.57	−8.81	9.16
Lakshadweep	ari	Age	2-3	0.73	4.57	−8.29	9.50
Lakshadweep	ari	Age	3-4	−0.50	4.55	−9.66	8.22
Lakshadweep	ari	Age	4-5	−0.35	4.68	−9.52	8.61
Lakshadweep	ari	Month	August	−0.90	4.55	−9.75	7.94
Lakshadweep	ari	Month	September	−0.39	4.76	−9.73	8.99
Lakshadweep	ari	Wealth	Richer	0.01	4.33	−8.45	8.58
Lakshadweep	ari	Wealth	Richest	0.36	4.45	−8.32	9.03
Lakshadweep	ari	Mothers education	Secondary	−0.46	4.38	−8.97	8.31
Lakshadweep	ari	Mothers education	Higher	0.03	4.53	−8.89	8.74
Lakshadweep	ari	Mothers age	30-40	−0.27	4.28	−8.48	7.76
Lakshadweep	ari	Mothers age	40+	−0.06	5.01	−9.63	9.53
Lakshadweep	ari	Sex	Female	−0.32	4.32	−8.71	8.30
Lakshadweep	ari	Birth weight	Average	−0.25	4.25	−8.55	7.97
Lakshadweep	ari	Birth weight	Below Average	0.84	4.44	−8.11	9.48
Lakshadweep	ari	Breastfed	No	0.11	4.37	−8.53	8.38
Lakshadweep	ari	Toilet	Improved	0.37	4.63	−8.85	9.38
Lakshadweep	ari	Location	Urban	0.52	4.76	−8.65	10.20
Lakshadweep	ari	Cookign fuel	Solid	0.25	4.42	−8.27	8.89
Lakshadweep	ari	Household size	05-Oct	−1.02	4.37	−9.46	7.66
Lakshadweep	ari	Household size	10+	−0.04	4.52	−9.11	8.70
Lakshadweep	ari	LLIN	Yes	0.00	4.97	−9.55	9.73
Lakshadweep	ari	Access	Significant problem	−0.68	4.36	−9.48	7.89
Lakshadweep	ari	Vaccination	-	0.03	4.99	−9.62	9.73

Name	Response	Group	Level	Estimate	Error	lci	uci
Lakshadweep	dia	Intercept	-	−9.38	10.55	−30.58	10.43
Lakshadweep	dia	Age	1-2	0.06	3.88	−7.65	7.60
Lakshadweep	dia	Age	2-3	−2.06	4.13	−10.44	5.82
Lakshadweep	dia	Age	3-4	−1.79	4.04	−9.98	5.80
Lakshadweep	dia	Age	4-5	0.05	3.93	−7.80	7.52
Lakshadweep	dia	Month	August	1.73	4.30	−6.67	10.25
Lakshadweep	dia	Month	September	−0.63	4.69	−9.99	8.22
Lakshadweep	dia	Wealth	Richer	0.21	3.93	−7.52	7.98
Lakshadweep	dia	Wealth	Richest	−0.98	4.10	−9.03	7.06
Lakshadweep	dia	Mothers education	Secondary	−0.02	3.99	−7.72	8.03
Lakshadweep	dia	Mothers education	Higher	−0.15	4.33	−8.65	8.24
Lakshadweep	dia	Mothers age	30-40	1.60	3.57	−5.27	8.66
Lakshadweep	dia	Mothers age	40+	0.61	4.85	−8.91	9.89
Lakshadweep	dia	Sex	Female	0.27	3.64	−7.02	7.41
Lakshadweep	dia	Birth weight	Average	−2.27	3.67	−9.57	4.87
Lakshadweep	dia	Birth weight	Below Average	−0.04	3.99	−8.07	7.70
Lakshadweep	dia	Breastfed	No	−1.06	3.97	−9.05	6.38
Lakshadweep	dia	Toilet	Improved	−1.11	4.19	−9.68	6.78
Lakshadweep	dia	Location	Urban	−2.19	4.21	−10.38	6.19
Lakshadweep	dia	Cookign fuel	Solid	0.44	4.00	−7.26	8.52
Lakshadweep	dia	Household size	05-Oct	1.24	3.96	−6.38	9.18
Lakshadweep	dia	Household size	10+	−0.01	4.12	−8.35	8.03
Lakshadweep	dia	LLIN	Yes	−0.12	4.91	−9.76	9.48
Lakshadweep	dia	Access	Significant problem	−0.59	3.73	−8.14	6.66
Lakshadweep	dia	Vaccination	-	−0.02	5.06	−9.99	9.92

Name	Response	Group	Level	Estimate	Error	lci	uci
Lakshadweep	fever	Intercept	-	−2.32	9.41	−21.08	15.69
Lakshadweep	fever	Age	1-2	−0.23	3.55	−7.29	6.61
Lakshadweep	fever	Age	2-3	1.94	3.42	−4.82	8.65
Lakshadweep	fever	Age	3-4	−1.48	3.61	−8.82	5.53
Lakshadweep	fever	Age	4-5	−0.89	3.74	−8.35	6.10
Lakshadweep	fever	Month	August	−0.10	3.89	−7.54	7.55
Lakshadweep	fever	Month	September	−0.42	4.46	−9.35	8.16
Lakshadweep	fever	Wealth	Richer	1.34	3.77	−5.89	8.73
Lakshadweep	fever	Wealth	Richest	0.79	3.75	−6.34	8.15
Lakshadweep	fever	Mothers education	Secondary	−0.86	3.59	−7.90	6.30
Lakshadweep	fever	Mothers education	Higher	−1.03	3.89	−8.70	6.58
Lakshadweep	fever	Mothers age	30-40	−2.22	3.23	−8.75	3.96
Lakshadweep	fever	Mothers age	40+	0.13	4.75	−9.50	9.28
Lakshadweep	fever	Sex	Female	−1.02	3.11	−7.26	4.92
Lakshadweep	fever	Birth weight	Average	−1.37	3.24	−7.58	5.12
Lakshadweep	fever	Birth weight	Below Average	1.34	3.67	−5.93	8.45
Lakshadweep	fever	Breastfed	No	−2.24	3.58	−9.52	4.38
Lakshadweep	fever	Toilet	Improved	−0.45	3.85	−8.25	6.69
Lakshadweep	fever	Location	Urban	−0.39	3.90	−7.82	7.39
Lakshadweep	fever	Cookign fuel	Solid	−0.56	3.47	−7.38	6.41
Lakshadweep	fever	Household size	05-Oct	−1.17	3.41	−7.76	5.44
Lakshadweep	fever	Household size	10+	−1.69	3.81	−9.34	5.68
Lakshadweep	fever	LLIN	Yes	−0.15	5.06	−10.03	9.69
Lakshadweep	fever	Access	Significant problem	−1.08	3.19	−7.58	5.13
Lakshadweep	fever	Vaccination	-	0.07	4.93	−9.79	9.90

Name	Response	Group	Level	Estimate	Error	lci	uci
Lakshadweep	wasted	Intercept	-	−9.28	9.69	−28.37	9.21
Lakshadweep	wasted	Age	1-2	0.42	3.68	−6.81	7.54
Lakshadweep	wasted	Age	2-3	0.48	3.71	−6.96	7.49
Lakshadweep	wasted	Age	3-4	0.67	3.56	−6.38	7.52
Lakshadweep	wasted	Age	4-5	0.14	3.74	−7.39	7.30
Lakshadweep	wasted	Month	August	0.51	3.91	−6.99	8.27
Lakshadweep	wasted	Month	September	0.78	4.41	−7.90	9.43
Lakshadweep	wasted	Wealth	Richer	1.10	3.75	−6.22	8.53
Lakshadweep	wasted	Wealth	Richest	−0.20	3.85	−7.97	7.21
Lakshadweep	wasted	Mothers education	Secondary	1.07	3.89	−6.38	8.79
Lakshadweep	wasted	Mothers education	Higher	−2.55	4.19	−10.93	5.52
Lakshadweep	wasted	Mothers age	30-40	0.98	3.15	−5.25	7.17
Lakshadweep	wasted	Mothers age	40+	−0.55	4.74	−9.93	8.55
Lakshadweep	wasted	Sex	Female	−2.14	3.13	−8.39	3.95
Lakshadweep	wasted	Birth weight	Average	0.87	3.39	−5.48	7.71
Lakshadweep	wasted	Birth weight	Below Average	1.43	3.70	−5.95	8.70
Lakshadweep	wasted	Breastfed	No	−0.34	3.47	−7.40	6.19
Lakshadweep	wasted	Toilet	Improved	−0.79	3.87	−8.53	6.49
Lakshadweep	wasted	Location	Urban	−1.25	3.95	−8.65	6.80
Lakshadweep	wasted	Cookign fuel	Solid	0.14	3.67	−6.76	7.59
Lakshadweep	wasted	Household size	05-Oct	1.29	3.53	−5.47	8.47
Lakshadweep	wasted	Household size	10+	−1.04	3.91	−8.81	6.54
Lakshadweep	wasted	LLIN	Yes	−0.21	4.94	−9.88	9.47
Lakshadweep	wasted	Access	Significant problem	0.55	3.15	−5.64	6.70
Lakshadweep	wasted	Vaccination	-	0.02	4.96	−9.86	9.89

Name	Response	Group	Level	Estimate	Error	lci	uci
Lesotho	ari	Intercept	-	−8.43	2.63	−13.86	−3.56
Lesotho	ari	Age	1-2	0.73	0.63	−0.50	2.04
Lesotho	ari	Age	2-3	−0.02	0.71	−1.42	1.39
Lesotho	ari	Age	3-4	0.82	0.68	−0.50	2.17
Lesotho	ari	Age	4-5	0.34	0.71	−1.06	1.73
Lesotho	ari	Month	October	0.34	0.79	−1.14	1.94
Lesotho	ari	Month	November	0.15	0.78	−1.33	1.74
Lesotho	ari	Month	December	0.27	1.19	−2.16	2.55
Lesotho	ari	Wealth	Poorer	0.26	0.58	−0.86	1.40
Lesotho	ari	Wealth	Middle	0.45	0.70	−0.92	1.82
Lesotho	ari	Wealth	Richer	1.56	0.75	0.08	3.05
Lesotho	ari	Wealth	Richest	1.56	0.98	−0.34	3.48
Lesotho	ari	Mothers education	Primary	0.75	1.52	−1.89	4.17
Lesotho	ari	Mothers education	Secondary	−0.03	1.54	−2.73	3.37
Lesotho	ari	Mothers education	Higher	−0.87	1.79	−4.26	2.83
Lesotho	ari	Mothers age	20-30	0.62	0.60	−0.50	1.87
Lesotho	ari	Mothers age	30-40	1.08	0.64	−0.11	2.39
Lesotho	ari	Mothers age	40+	0.41	0.95	−1.48	2.22
Lesotho	ari	Sex	Female	−0.23	0.36	−0.94	0.47
Lesotho	ari	Birth weight	Average	−0.85	0.44	−1.73	0.04
Lesotho	ari	Birth weight	Below Average	0.08	0.58	−1.08	1.22
Lesotho	ari	Breastfed	No	−0.72	0.47	−1.64	0.16
Lesotho	ari	Toilet	Unimproved	0.50	0.51	−0.52	1.51
Lesotho	ari	Toilet	Improved	−1.20	0.58	−2.41	−0.15
Lesotho	ari	Location	Urban	−0.02	0.66	−1.33	1.28
Lesotho	ari	Cookign fuel	Solid	1.23	0.65	0.01	2.60
Lesotho	ari	Household size	05-Oct	−0.13	0.41	−0.91	0.69
Lesotho	ari	Household size	10+	−0.51	0.78	−2.15	0.91
Lesotho	ari	Access	Significant problem	0.07	0.39	−0.68	0.83
Lesotho	ari	Vaccination	-	2.48	2.63	−2.60	7.80

Name	Response	Group	Level	Estimate	Error	lci	uci
Lesotho	dia	Intercept	-	−5.67	2.50	−10.89	−1.14
Lesotho	dia	Age	1-2	−0.05	0.32	−0.67	0.56
Lesotho	dia	Age	2-3	−1.32	0.39	−2.11	−0.56
Lesotho	dia	Age	3-4	−2.18	0.49	−3.20	−1.26
Lesotho	dia	Age	4-5	−2.26	0.50	−3.26	−1.30
Lesotho	dia	Month	October	0.07	0.49	−0.86	1.05
Lesotho	dia	Month	November	−0.49	0.50	−1.46	0.50
Lesotho	dia	Month	December	−0.23	0.82	−1.86	1.32
Lesotho	dia	Wealth	Poorer	−0.06	0.37	−0.81	0.67
Lesotho	dia	Wealth	Middle	−0.49	0.46	−1.40	0.39
Lesotho	dia	Wealth	Richer	−1.20	0.58	−2.39	−0.08
Lesotho	dia	Wealth	Richest	−0.78	0.67	−2.12	0.51
Lesotho	dia	Mothers education	Primary	2.51	2.03	−0.88	6.94
Lesotho	dia	Mothers education	Secondary	2.46	2.03	−0.96	6.87
Lesotho	dia	Mothers education	Higher	0.46	2.17	−3.38	5.03
Lesotho	dia	Mothers age	20-30	−0.49	0.30	−1.09	0.11
Lesotho	dia	Mothers age	30-40	−0.84	0.39	−1.62	−0.10
Lesotho	dia	Mothers age	40+	−0.67	0.67	−2.03	0.56
Lesotho	dia	Sex	Female	−0.01	0.24	−0.48	0.46
Lesotho	dia	Birth weight	Average	−0.24	0.33	−0.88	0.42
Lesotho	dia	Birth weight	Below Average	0.30	0.42	−0.53	1.13
Lesotho	dia	Breastfed	No	−0.23	0.32	−0.87	0.39
Lesotho	dia	Toilet	Unimproved	0.19	0.36	−0.51	0.90
Lesotho	dia	Toilet	Improved	0.28	0.33	−0.37	0.93
Lesotho	dia	Location	Urban	0.47	0.45	−0.41	1.35
Lesotho	dia	Cookign fuel	Solid	−0.51	0.46	−1.42	0.40
Lesotho	dia	Household size	05-Oct	−0.05	0.28	−0.59	0.50
Lesotho	dia	Household size	10+	−0.56	0.52	−1.60	0.43
Lesotho	dia	Access	Significant problem	−0.01	0.26	−0.54	0.50
Lesotho	dia	Vaccination	-	4.54	1.93	0.82	8.38

Name	Response	Group	Level	Estimate	Error	lci	uci
Lesotho	fever	Intercept	-	−3.32	1.62	−6.61	−0.31
Lesotho	fever	Age	1-2	0.07	0.34	−0.58	0.75
Lesotho	fever	Age	2-3	0.16	0.36	−0.53	0.87
Lesotho	fever	Age	3-4	0.07	0.39	−0.70	0.86
Lesotho	fever	Age	4-5	−0.22	0.39	−0.98	0.54
Lesotho	fever	Month	October	−0.11	0.44	−0.96	0.77
Lesotho	fever	Month	November	−0.37	0.45	−1.27	0.51
Lesotho	fever	Month	December	−0.86	0.75	−2.35	0.55
Lesotho	fever	Wealth	Poorer	0.20	0.35	−0.48	0.89
Lesotho	fever	Wealth	Middle	0.67	0.41	−0.12	1.49
Lesotho	fever	Wealth	Richer	1.17	0.47	0.24	2.11
Lesotho	fever	Wealth	Richest	1.43	0.57	0.34	2.58
Lesotho	fever	Mothers education	Primary	−0.19	0.91	−1.88	1.68
Lesotho	fever	Mothers education	Secondary	−0.12	0.92	−1.84	1.79
Lesotho	fever	Mothers education	Higher	−1.85	1.09	−3.93	0.35
Lesotho	fever	Mothers age	20-30	0.47	0.32	−0.14	1.11
Lesotho	fever	Mothers age	30-40	0.53	0.35	−0.15	1.23
Lesotho	fever	Mothers age	40+	0.61	0.52	−0.42	1.65
Lesotho	fever	Sex	Female	−0.18	0.21	−0.59	0.22
Lesotho	fever	Birth weight	Average	−0.13	0.28	−0.69	0.44
Lesotho	fever	Birth weight	Below Average	0.50	0.36	−0.20	1.22
Lesotho	fever	Breastfed	No	−0.56	0.26	−1.08	−0.05
Lesotho	fever	Toilet	Unimproved	0.20	0.31	−0.41	0.80
Lesotho	fever	Toilet	Improved	−0.48	0.32	−1.13	0.13
Lesotho	fever	Location	Urban	0.41	0.40	−0.37	1.20
Lesotho	fever	Cookign fuel	Solid	0.73	0.36	0.03	1.44
Lesotho	fever	Household size	05-Oct	0.50	0.26	−0.00	1.02
Lesotho	fever	Household size	10+	0.04	0.46	−0.86	0.92
Lesotho	fever	Access	Significant problem	0.78	0.24	0.33	1.26
Lesotho	fever	Vaccination	-	−0.79	1.72	−4.09	2.60

Name	Response	Group	Level	Estimate	Error	lci	uci
Lesotho	wasted	Intercept	-	−18.10	5.20	−28.88	−8.64
Lesotho	wasted	Age	1-2	0.17	0.93	−1.56	2.11
Lesotho	wasted	Age	2-3	−1.65	1.04	−3.80	0.33
Lesotho	wasted	Age	3-4	−5.61	1.84	−9.71	−2.39
Lesotho	wasted	Age	4-5	−3.17	1.25	−5.80	−0.90
Lesotho	wasted	Month	October	−0.95	1.51	−3.87	2.18
Lesotho	wasted	Month	November	0.17	1.48	−2.61	3.23
Lesotho	wasted	Month	December	−2.31	2.76	−8.09	2.83
Lesotho	wasted	Wealth	Poorer	1.06	0.91	−0.63	2.94
Lesotho	wasted	Wealth	Middle	−1.08	1.44	−4.15	1.51
Lesotho	wasted	Wealth	Richer	1.32	1.57	−1.75	4.45
Lesotho	wasted	Wealth	Richest	−2.97	2.74	−8.77	1.95
Lesotho	wasted	Mothers education	Primary	2.47	2.62	−2.25	7.93
Lesotho	wasted	Mothers education	Secondary	0.32	2.64	−4.45	5.78
Lesotho	wasted	Mothers education	Higher	−0.59	3.33	−7.34	5.86
Lesotho	wasted	Mothers age	20-30	0.69	0.98	−1.14	2.70
Lesotho	wasted	Mothers age	30-40	−0.42	1.19	−2.85	1.84
Lesotho	wasted	Mothers age	40+	1.25	1.58	−1.75	4.42
Lesotho	wasted	Sex	Female	0.70	0.68	−0.60	2.08
Lesotho	wasted	Birth weight	Average	1.76	1.18	−0.31	4.34
Lesotho	wasted	Birth weight	Below Average	3.29	1.38	0.90	6.34
Lesotho	wasted	Breastfed	No	0.96	0.82	−0.67	2.57
Lesotho	wasted	Toilet	Unimproved	2.11	1.05	0.21	4.43
Lesotho	wasted	Toilet	Improved	0.79	0.99	−1.01	2.93
Lesotho	wasted	Location	Urban	2.06	1.47	−0.68	5.33
Lesotho	wasted	Cookign fuel	Solid	2.33	1.72	−0.76	6.03
Lesotho	wasted	Household size	05-Oct	0.44	0.82	−1.15	2.08
Lesotho	wasted	Household size	10+	−1.03	1.67	−4.52	2.04
Lesotho	wasted	Access	Significant problem	0.75	0.84	−0.77	2.60
Lesotho	wasted	Vaccination	-	4.37	3.90	−3.55	11.71

Name	Response	Group	Level	Estimate	Error	lci	uci
Liberia	ari	Intercept	-	−1.47	0.67	−2.79	−0.15
Liberia	ari	Age	1-2	−0.32	0.26	−0.82	0.18
Liberia	ari	Age	2-3	−0.84	0.29	−1.41	−0.27
Liberia	ari	Age	3-4	−0.79	0.30	−1.37	−0.21
Liberia	ari	Age	4-5	−1.41	0.33	−2.08	−0.77
Liberia	ari	Month	April	−0.77	0.46	−1.67	0.12
Liberia	ari	Month	May	−0.08	0.53	−1.12	0.96
Liberia	ari	Month	June	0.36	0.56	−0.73	1.46
Liberia	ari	Month	July	0.22	0.71	−1.19	1.61
Liberia	ari	Wealth	Poorer	−0.06	0.27	−0.59	0.46
Liberia	ari	Wealth	Middle	−0.56	0.36	−1.29	0.14
Liberia	ari	Wealth	Richer	−0.29	0.43	−1.14	0.54
Liberia	ari	Wealth	Richest	−0.10	0.48	−1.05	0.83
Liberia	ari	Mothers education	Primary	−0.43	0.24	−0.90	0.03
Liberia	ari	Mothers education	Secondary	0.12	0.27	−0.41	0.63
Liberia	ari	Mothers age	20-30	−0.19	0.27	−0.70	0.34
Liberia	ari	Mothers age	30-40	−0.09	0.30	−0.67	0.50
Liberia	ari	Mothers age	40+	0.29	0.43	−0.56	1.13
Liberia	ari	Sex	Female	−0.26	0.19	−0.63	0.10
Liberia	ari	Birth weight	Average	−0.15	0.21	−0.57	0.27
Liberia	ari	Birth weight	Below Average	0.17	0.24	−0.30	0.65
Liberia	ari	Breastfed	No	0.07	0.21	−0.35	0.48
Liberia	ari	Toilet	Unimproved	0.08	0.23	−0.38	0.55
Liberia	ari	Toilet	Improved	0.23	0.24	−0.24	0.72
Liberia	ari	Location	Urban	−0.30	0.32	−0.93	0.34
Liberia	ari	Household size	05-Oct	0.14	0.23	−0.30	0.58
Liberia	ari	Household size	10+	0.17	0.31	−0.45	0.77
Liberia	ari	LLIN	Yes	0.16	0.20	−0.22	0.55
Liberia	ari	Access	Significant problem	0.22	0.22	−0.21	0.65
Liberia	ari	Vaccination	-	−1.81	1.52	−4.76	1.17

Name	Response	Group	Level	Estimate	Error	lci	uci
Arunachal Pradesh	fever	Intercept	-	−2.99	2.71	−8.35	2.24
Arunachal Pradesh	fever	Age	1-2	−0.10	1.16	−2.36	2.22
Arunachal Pradesh	fever	Age	2-3	0.07	1.13	−2.07	2.39
Arunachal Pradesh	fever	Age	3-4	−0.50	1.19	−2.83	1.88
Arunachal Pradesh	fever	Age	4-5	−1.01	1.25	−3.48	1.47
Arunachal Pradesh	fever	Month	May	−0.56	1.15	−2.84	1.76
Arunachal Pradesh	fever	Month	June	−1.80	3.69	−9.83	4.49
Arunachal Pradesh	fever	Month	August	−0.29	1.19	−2.70	2.02
Arunachal Pradesh	fever	Month	September	−0.16	1.23	−2.65	2.17
Arunachal Pradesh	fever	Month	October	0.06	1.08	−2.06	2.19
Arunachal Pradesh	fever	Month	November	−0.73	1.29	−3.33	1.71
Arunachal Pradesh	fever	Wealth	Poorer	0.26	0.97	−1.64	2.18
Arunachal Pradesh	fever	Wealth	Middle	−0.04	1.16	−2.35	2.25
Arunachal Pradesh	fever	Wealth	Richer	0.22	1.48	−2.66	3.16
Arunachal Pradesh	fever	Wealth	Richest	0.04	1.83	−3.67	3.50
Arunachal Pradesh	fever	Mothers education	Primary	−0.12	1.03	−2.23	1.84
Arunachal Pradesh	fever	Mothers education	Secondary	−0.20	0.83	−1.80	1.45
Arunachal Pradesh	fever	Mothers education	Higher	−0.74	1.71	−4.35	2.33
Arunachal Pradesh	fever	Mothers age	20-30	0.56	1.09	−1.39	2.97
Arunachal Pradesh	fever	Mothers age	30-40	0.27	1.26	−2.12	2.92
Arunachal Pradesh	fever	Mothers age	40+	−0.27	2.62	−6.14	4.28
Arunachal Pradesh	fever	Sex	Female	0.03	0.64	−1.25	1.30
Arunachal Pradesh	fever	Birth weight	Average	0.10	0.87	−1.52	1.93
Arunachal Pradesh	fever	Birth weight	Below Average	0.31	1.16	−2.03	2.59
Arunachal Pradesh	fever	Breastfed	No	0.05	0.72	−1.40	1.44
Arunachal Pradesh	fever	Toilet	Unimproved	0.32	0.81	−1.28	1.91
Arunachal Pradesh	fever	Toilet	Improved	0.21	0.90	−1.65	1.89
Arunachal Pradesh	fever	Location	Urban	0.00	0.92	−1.80	1.75
Arunachal Pradesh	fever	Cookign fuel	Solid	−0.27	1.06	−2.29	1.87
Arunachal Pradesh	fever	Household size	05-Oct	0.36	0.74	−1.03	1.83
Arunachal Pradesh	fever	Household size	10+	0.44	1.54	−2.79	3.22
Arunachal Pradesh	fever	LLIN	Yes	−0.12	0.73	−1.61	1.29
Arunachal Pradesh	fever	Access	Significant problem	−0.20	0.71	−1.58	1.21
Arunachal Pradesh	fever	Vaccination	-	0.08	4.91	−9.56	9.70

Name	Response	Group	Level	Estimate	Error	lci	uci
Liberia	dia	Intercept	-	0.34	0.41	−0.47	1.15
Liberia	dia	Age	1-2	0.10	0.17	−0.23	0.42
Liberia	dia	Age	2-3	−0.25	0.18	−0.60	0.09
Liberia	dia	Age	3-4	−0.58	0.19	−0.95	−0.21
Liberia	dia	Age	4-5	−0.94	0.20	−1.33	−0.55
Liberia	dia	Month	April	0.62	0.28	0.07	1.17
Liberia	dia	Month	May	0.37	0.33	−0.27	0.99
Liberia	dia	Month	June	0.32	0.34	−0.34	1.00
Liberia	dia	Month	July	−0.47	0.46	−1.38	0.43
Liberia	dia	Wealth	Poorer	0.29	0.17	−0.04	0.62
Liberia	dia	Wealth	Middle	0.41	0.21	0.00	0.81
Liberia	dia	Wealth	Richer	0.46	0.26	−0.04	0.96
Liberia	dia	Wealth	Richest	0.61	0.30	0.00	1.20
Liberia	dia	Mothers education	Primary	−0.03	0.14	−0.30	0.24
Liberia	dia	Mothers education	Secondary	−0.10	0.16	−0.41	0.22
Liberia	dia	Mothers age	20-30	−0.03	0.15	−0.32	0.28
Liberia	dia	Mothers age	30-40	−0.26	0.17	−0.59	0.09
Liberia	dia	Mothers age	40+	−0.95	0.32	−1.58	−0.34
Liberia	dia	Sex	Female	0.01	0.11	−0.20	0.23
Liberia	dia	Birth weight	Average	0.05	0.13	−0.19	0.30
Liberia	dia	Birth weight	Below Average	0.08	0.15	−0.21	0.36
Liberia	dia	Breastfed	No	−0.26	0.13	−0.52	−0.01
Liberia	dia	Toilet	Unimproved	−0.05	0.14	−0.32	0.23
Liberia	dia	Toilet	Improved	0.53	0.15	0.24	0.84
Liberia	dia	Location	Urban	−0.11	0.19	−0.47	0.26
Liberia	dia	Household size	05-Oct	−0.10	0.13	−0.36	0.16
Liberia	dia	Household size	10+	−0.06	0.18	−0.43	0.30
Liberia	dia	LLIN	Yes	−0.10	0.12	−0.33	0.14
Liberia	dia	Access	Significant problem	−0.24	0.13	−0.49	0.01
Liberia	dia	Vaccination	-	−3.34	0.92	−5.11	−1.55

Name	Response	Group	Level	Estimate	Error	lci	uci
Liberia	fever	Intercept	-	0.43	0.40	−0.34	1.21
Liberia	fever	Age	1-2	−0.18	0.16	−0.50	0.14
Liberia	fever	Age	2-3	−0.44	0.17	−0.76	−0.12
Liberia	fever	Age	3-4	−0.66	0.17	−1.01	−0.32
Liberia	fever	Age	4-5	−0.67	0.18	−1.02	−0.31
Liberia	fever	Month	April	−0.39	0.27	−0.93	0.14
Liberia	fever	Month	May	−0.13	0.31	−0.74	0.48
Liberia	fever	Month	June	0.22	0.33	−0.43	0.86
Liberia	fever	Month	July	−0.30	0.42	−1.14	0.53
Liberia	fever	Wealth	Poorer	−0.14	0.16	−0.44	0.17
Liberia	fever	Wealth	Middle	−0.02	0.19	−0.39	0.36
Liberia	fever	Wealth	Richer	−0.38	0.24	−0.86	0.09
Liberia	fever	Wealth	Richest	−0.51	0.28	−1.07	0.04
Liberia	fever	Mothers education	Primary	0.26	0.13	0.01	0.51
Liberia	fever	Mothers education	Secondary	0.63	0.15	0.35	0.92
Liberia	fever	Mothers age	20-30	0.10	0.14	−0.18	0.38
Liberia	fever	Mothers age	30-40	0.23	0.16	−0.09	0.57
Liberia	fever	Mothers age	40+	0.45	0.26	−0.05	0.96
Liberia	fever	Sex	Female	−0.12	0.10	−0.33	0.08
Liberia	fever	Birth weight	Average	−0.24	0.12	−0.46	−0.01
Liberia	fever	Birth weight	Below Average	−0.03	0.14	−0.30	0.23
Liberia	fever	Breastfed	No	−0.21	0.12	−0.44	0.02
Liberia	fever	Toilet	Unimproved	−0.15	0.13	−0.41	0.11
Liberia	fever	Toilet	Improved	0.37	0.14	0.09	0.64
Liberia	fever	Location	Urban	−0.38	0.18	−0.74	−0.03
Liberia	fever	Household size	05-Oct	−0.11	0.12	−0.35	0.13
Liberia	fever	Household size	10+	0.12	0.17	−0.21	0.45
Liberia	fever	LLIN	Yes	0.22	0.11	0.01	0.44
Liberia	fever	Access	Significant problem	−0.14	0.12	−0.38	0.09
Liberia	fever	Vaccination	-	−1.30	0.88	−2.99	0.45

Name	Response	Group	Level	Estimate	Error	lci	uci
Liberia	wasted	Intercept	-	−3.07	0.75	−4.55	−1.64
Liberia	wasted	Age	1-2	−0.64	0.24	−1.11	−0.17
Liberia	wasted	Age	2-3	−1.41	0.29	−1.99	−0.86
Liberia	wasted	Age	3-4	−2.19	0.35	−2.92	−1.53
Liberia	wasted	Age	4-5	−2.12	0.37	−2.86	−1.40
Liberia	wasted	Month	April	0.59	0.48	−0.35	1.55
Liberia	wasted	Month	May	0.99	0.55	−0.10	2.08
Liberia	wasted	Month	June	0.93	0.58	−0.25	2.08
Liberia	wasted	Month	July	1.13	0.73	−0.32	2.56
Liberia	wasted	Wealth	Poorer	0.25	0.30	−0.34	0.87
Liberia	wasted	Wealth	Middle	0.24	0.36	−0.48	0.94
Liberia	wasted	Wealth	Richer	0.01	0.43	−0.83	0.88
Liberia	wasted	Wealth	Richest	−0.10	0.51	−1.13	0.90
Liberia	wasted	Mothers education	Primary	−0.11	0.25	−0.59	0.38
Liberia	wasted	Mothers education	Secondary	0.17	0.28	−0.38	0.72
Liberia	wasted	Mothers age	20-30	0.23	0.27	−0.29	0.79
Liberia	wasted	Mothers age	30-40	0.26	0.31	−0.35	0.88
Liberia	wasted	Mothers age	40+	−0.44	0.64	−1.79	0.74
Liberia	wasted	Sex	Female	−0.27	0.19	−0.65	0.10
Liberia	wasted	Birth weight	Average	0.94	0.24	0.48	1.41
Liberia	wasted	Birth weight	Below Average	1.57	0.25	1.07	2.05
Liberia	wasted	Breastfed	No	0.12	0.23	−0.34	0.58
Liberia	wasted	Toilet	Unimproved	0.01	0.24	−0.46	0.49
Liberia	wasted	Toilet	Improved	0.21	0.27	−0.32	0.72
Liberia	wasted	Location	Urban	0.07	0.31	−0.54	0.67
Liberia	wasted	Household size	05-Oct	0.40	0.25	−0.07	0.90
Liberia	wasted	Household size	10+	0.87	0.32	0.25	1.50
Liberia	wasted	LLIN	Yes	0.12	0.21	−0.28	0.53
Liberia	wasted	Access	Significant problem	−0.17	0.23	−0.60	0.28
Liberia	wasted	Vaccination	-	−2.37	1.50	−5.35	0.58

Name	Response	Group	Level	Estimate	Error	lci	uci
Madhya Pradesh	ari	Intercept	-	−4.16	2.31	−8.56	0.32
Madhya Pradesh	ari	Age	1-2	0.26	0.22	−0.18	0.70
Madhya Pradesh	ari	Age	2-3	−0.03	0.24	−0.50	0.44
Madhya Pradesh	ari	Age	3-4	−0.23	0.25	−0.71	0.26
Madhya Pradesh	ari	Age	4-5	−0.40	0.26	−0.91	0.11
Madhya Pradesh	ari	Month	March	−0.52	0.20	−0.91	−0.11
Madhya Pradesh	ari	Month	April	−0.88	0.23	−1.32	−0.44
Madhya Pradesh	ari	Month	May	−1.09	0.23	−1.54	−0.62
Madhya Pradesh	ari	Month	June	−1.70	0.30	−2.30	−1.14
Madhya Pradesh	ari	Month	July	−2.34	0.59	−3.63	−1.33
Madhya Pradesh	ari	Wealth	Poorer	0.06	0.18	−0.29	0.39
Madhya Pradesh	ari	Wealth	Middle	0.25	0.23	−0.21	0.70
Madhya Pradesh	ari	Wealth	Richer	0.30	0.33	−0.34	0.93
Madhya Pradesh	ari	Wealth	Richest	0.70	0.40	−0.10	1.47
Madhya Pradesh	ari	Mothers education	Primary	0.31	0.19	−0.06	0.68
Madhya Pradesh	ari	Mothers education	Secondary	0.18	0.18	−0.16	0.54
Madhya Pradesh	ari	Mothers education	Higher	0.05	0.37	−0.70	0.77
Madhya Pradesh	ari	Mothers age	20-30	−0.05	0.22	−0.46	0.37
Madhya Pradesh	ari	Mothers age	30-40	0.23	0.28	−0.33	0.78
Madhya Pradesh	ari	Sex	Female	0.15	0.13	−0.10	0.41
Madhya Pradesh	ari	Birth weight	Average	−0.10	0.17	−0.44	0.24
Madhya Pradesh	ari	Birth weight	Below Average	0.24	0.22	−0.20	0.67
Madhya Pradesh	ari	Breastfed	No	−0.17	0.15	−0.46	0.12
Madhya Pradesh	ari	Toilet	Unimproved	0.48	0.23	0.04	0.93
Madhya Pradesh	ari	Toilet	Improved	−0.36	0.21	−0.78	0.04
Madhya Pradesh	ari	Location	Urban	−0.51	0.23	−0.97	−0.06
Madhya Pradesh	ari	Cookign fuel	Solid	0.20	0.27	−0.32	0.74
Madhya Pradesh	ari	Household size	05-Oct	−0.08	0.16	−0.39	0.25
Madhya Pradesh	ari	Household size	10+	−0.39	0.25	−0.91	0.09
Madhya Pradesh	ari	LLIN	Yes	−0.91	0.65	−2.34	0.22
Madhya Pradesh	ari	Access	Significant problem	−0.05	0.14	−0.33	0.23
Madhya Pradesh	ari	Vaccination	-	−0.05	4.97	−9.75	9.44

Name	Response	Group	Level	Estimate	Error	lci	uci
Madhya Pradesh	dia	Intercept	-	−1.75	2.30	−6.17	2.80
Madhya Pradesh	dia	Age	1-2	−0.21	0.10	−0.39	−0.02
Madhya Pradesh	dia	Age	2-3	−0.84	0.11	−1.05	−0.63
Madhya Pradesh	dia	Age	3-4	−1.24	0.12	−1.46	−1.00
Madhya Pradesh	dia	Age	4-5	−1.44	0.13	−1.69	−1.20
Madhya Pradesh	dia	Month	March	−0.37	0.12	−0.61	−0.13
Madhya Pradesh	dia	Month	April	−0.18	0.12	−0.42	0.06
Madhya Pradesh	dia	Month	May	−0.09	0.12	−0.33	0.14
Madhya Pradesh	dia	Month	June	−0.35	0.13	−0.61	−0.10
Madhya Pradesh	dia	Month	July	−0.44	0.19	−0.81	−0.07
Madhya Pradesh	dia	Wealth	Poorer	0.08	0.09	−0.11	0.26
Madhya Pradesh	dia	Wealth	Middle	0.12	0.12	−0.12	0.35
Madhya Pradesh	dia	Wealth	Richer	−0.06	0.16	−0.38	0.25
Madhya Pradesh	dia	Wealth	Richest	−0.04	0.19	−0.42	0.34
Madhya Pradesh	dia	Mothers education	Primary	0.19	0.10	−0.00	0.38
Madhya Pradesh	dia	Mothers education	Secondary	0.10	0.09	−0.07	0.28
Madhya Pradesh	dia	Mothers education	Higher	−0.06	0.17	−0.40	0.27
Madhya Pradesh	dia	Mothers age	20-30	0.12	0.12	−0.11	0.36
Madhya Pradesh	dia	Mothers age	30-40	0.12	0.15	−0.18	0.42
Madhya Pradesh	dia	Sex	Female	−0.07	0.07	−0.19	0.06
Madhya Pradesh	dia	Birth weight	Average	−0.10	0.09	−0.26	0.07
Madhya Pradesh	dia	Birth weight	Below Average	0.25	0.12	0.02	0.48
Madhya Pradesh	dia	Breastfed	No	−0.18	0.08	−0.33	−0.04
Madhya Pradesh	dia	Toilet	Unimproved	0.03	0.11	−0.19	0.23
Madhya Pradesh	dia	Toilet	Improved	−0.15	0.10	−0.35	0.04
Madhya Pradesh	dia	Location	Urban	0.11	0.11	−0.10	0.31
Madhya Pradesh	dia	Cookign fuel	Solid	−0.09	0.13	−0.34	0.15
Madhya Pradesh	dia	Household size	05-Oct	0.07	0.08	−0.09	0.24
Madhya Pradesh	dia	Household size	10+	0.03	0.12	−0.21	0.26
Madhya Pradesh	dia	LLIN	Yes	−0.04	0.22	−0.50	0.39
Madhya Pradesh	dia	Access	Significant problem	0.10	0.07	−0.04	0.24
Madhya Pradesh	dia	Vaccination	-	0.08	5.03	−9.78	9.82

Name	Response	Group	Level	Estimate	Error	lci	uci
Madhya Pradesh	fever	Intercept	-	−1.40	2.27	−5.89	3.19
Madhya Pradesh	fever	Age	1-2	0.05	0.10	−0.15	0.25
Madhya Pradesh	fever	Age	2-3	−0.31	0.11	−0.51	−0.10
Madhya Pradesh	fever	Age	3-4	−0.34	0.11	−0.56	−0.13
Madhya Pradesh	fever	Age	4-5	−0.57	0.11	−0.78	−0.34
Madhya Pradesh	fever	Month	March	−0.31	0.11	−0.51	−0.10
Madhya Pradesh	fever	Month	April	−0.52	0.11	−0.74	−0.31
Madhya Pradesh	fever	Month	May	−0.61	0.11	−0.82	−0.39
Madhya Pradesh	fever	Month	June	−1.13	0.13	−1.38	−0.89
Madhya Pradesh	fever	Month	July	−0.80	0.17	−1.15	−0.46
Madhya Pradesh	fever	Wealth	Poorer	0.15	0.09	−0.02	0.31
Madhya Pradesh	fever	Wealth	Middle	0.19	0.11	−0.03	0.40
Madhya Pradesh	fever	Wealth	Richer	0.20	0.14	−0.08	0.49
Madhya Pradesh	fever	Wealth	Richest	0.14	0.18	−0.20	0.49
Madhya Pradesh	fever	Mothers education	Primary	0.23	0.09	0.06	0.41
Madhya Pradesh	fever	Mothers education	Secondary	0.25	0.08	0.09	0.41
Madhya Pradesh	fever	Mothers education	Higher	0.22	0.16	−0.09	0.52
Madhya Pradesh	fever	Mothers age	20-30	−0.09	0.10	−0.28	0.12
Madhya Pradesh	fever	Mothers age	30-40	0.10	0.13	−0.16	0.35
Madhya Pradesh	fever	Sex	Female	0.02	0.06	−0.10	0.14
Madhya Pradesh	fever	Birth weight	Average	−0.16	0.08	−0.31	−0.00
Madhya Pradesh	fever	Birth weight	Below Average	0.28	0.10	0.08	0.48
Madhya Pradesh	fever	Breastfed	No	−0.34	0.07	−0.47	−0.20
Madhya Pradesh	fever	Toilet	Unimproved	0.02	0.10	−0.18	0.22
Madhya Pradesh	fever	Toilet	Improved	−0.13	0.09	−0.31	0.04
Madhya Pradesh	fever	Location	Urban	−0.25	0.10	−0.46	−0.05
Madhya Pradesh	fever	Cookign fuel	Solid	−0.13	0.11	−0.35	0.09
Madhya Pradesh	fever	Household size	05-Oct	−0.05	0.07	−0.20	0.10
Madhya Pradesh	fever	Household size	10+	−0.12	0.11	−0.32	0.09
Madhya Pradesh	fever	LLIN	Yes	−0.07	0.20	−0.47	0.32
Madhya Pradesh	fever	Access	Significant problem	0.05	0.07	−0.08	0.18
Madhya Pradesh	fever	Vaccination	-	0.02	4.97	−9.83	9.92

Name	Response	Group	Level	Estimate	Error	lci	uci
Madhya Pradesh	wasted	Intercept	-	−0.98	2.26	−5.36	3.39
Madhya Pradesh	wasted	Age	1-2	−0.26	0.07	−0.40	−0.12
Madhya Pradesh	wasted	Age	2-3	−0.48	0.08	−0.63	−0.34
Madhya Pradesh	wasted	Age	3-4	−0.63	0.08	−0.78	−0.48
Madhya Pradesh	wasted	Age	4-5	−0.69	0.08	−0.85	−0.54
Madhya Pradesh	wasted	Month	March	0.28	0.07	0.14	0.43
Madhya Pradesh	wasted	Month	April	0.30	0.08	0.15	0.45
Madhya Pradesh	wasted	Month	May	0.61	0.07	0.46	0.76
Madhya Pradesh	wasted	Month	June	0.61	0.08	0.46	0.77
Madhya Pradesh	wasted	Month	July	0.41	0.11	0.19	0.62
Madhya Pradesh	wasted	Wealth	Poorer	−0.08	0.06	−0.20	0.03
Madhya Pradesh	wasted	Wealth	Middle	−0.18	0.08	−0.33	−0.03
Madhya Pradesh	wasted	Wealth	Richer	−0.11	0.10	−0.32	0.09
Madhya Pradesh	wasted	Wealth	Richest	−0.40	0.13	−0.66	−0.15
Madhya Pradesh	wasted	Mothers education	Primary	−0.12	0.06	−0.25	0.00
Madhya Pradesh	wasted	Mothers education	Secondary	−0.05	0.06	−0.16	0.06
Madhya Pradesh	wasted	Mothers education	Higher	−0.12	0.12	−0.35	0.10
Madhya Pradesh	wasted	Mothers age	20-30	0.01	0.07	−0.13	0.16
Madhya Pradesh	wasted	Mothers age	30-40	0.08	0.09	−0.10	0.27
Madhya Pradesh	wasted	Sex	Female	−0.12	0.04	−0.20	−0.04
Madhya Pradesh	wasted	Birth weight	Average	0.02	0.06	−0.10	0.13
Madhya Pradesh	wasted	Birth weight	Below Average	0.44	0.08	0.29	0.58
Madhya Pradesh	wasted	Breastfed	No	−0.11	0.05	−0.20	−0.01
Madhya Pradesh	wasted	Toilet	Unimproved	0.15	0.07	0.01	0.29
Madhya Pradesh	wasted	Toilet	Improved	0.04	0.06	−0.08	0.15
Madhya Pradesh	wasted	Location	Urban	−0.08	0.07	−0.22	0.05
Madhya Pradesh	wasted	Cookign fuel	Solid	0.05	0.08	−0.12	0.21
Madhya Pradesh	wasted	Household size	05-Oct	−0.04	0.05	−0.15	0.06
Madhya Pradesh	wasted	Household size	10+	−0.02	0.08	−0.17	0.13
Madhya Pradesh	wasted	LLIN	Yes	−0.25	0.15	−0.55	0.04
Madhya Pradesh	wasted	Access	Significant problem	0.08	0.05	−0.01	0.17
Madhya Pradesh	wasted	Vaccination	-	−0.04	4.97	−9.70	9.52

Name	Response	Group	Level	Estimate	Error	lci	uci
Maharashtra	ari	Intercept	-	−7.31	2.73	−12.71	−2.01
Maharashtra	ari	Age	1-2	−0.16	0.20	−0.56	0.23
Maharashtra	ari	Age	2-3	−0.55	0.20	−0.95	−0.14
Maharashtra	ari	Age	3-4	−0.67	0.22	−1.12	−0.24
Maharashtra	ari	Age	4-5	−0.36	0.21	−0.77	0.05
Maharashtra	ari	Month	May	−1.23	0.53	−2.29	−0.21
Maharashtra	ari	Month	June	−1.34	0.51	−2.36	−0.32
Maharashtra	ari	Month	July	−1.39	0.53	−2.41	−0.34
Maharashtra	ari	Month	August	−2.16	0.63	−3.43	−0.95
Maharashtra	ari	Month	September	−2.41	0.77	−4.00	−0.92
Maharashtra	ari	Wealth	Poorer	−0.18	0.29	−0.75	0.42
Maharashtra	ari	Wealth	Middle	0.25	0.32	−0.36	0.88
Maharashtra	ari	Wealth	Richer	0.82	0.36	0.14	1.54
Maharashtra	ari	Wealth	Richest	0.83	0.41	0.04	1.65
Maharashtra	ari	Mothers education	Primary	1.78	0.38	1.05	2.54
Maharashtra	ari	Mothers education	Secondary	1.18	0.35	0.53	1.88
Maharashtra	ari	Mothers education	Higher	0.73	0.40	−0.03	1.52
Maharashtra	ari	Mothers age	20-30	−0.03	0.20	−0.42	0.38
Maharashtra	ari	Mothers age	30-40	−0.72	0.28	−1.27	−0.17
Maharashtra	ari	Sex	Female	−0.28	0.13	−0.53	−0.04
Maharashtra	ari	Birth weight	Average	−0.19	0.17	−0.51	0.15
Maharashtra	ari	Birth weight	Below Average	−0.24	0.23	−0.70	0.22
Maharashtra	ari	Breastfed	No	−1.16	0.17	−1.50	−0.83
Maharashtra	ari	Toilet	Unimproved	0.21	0.18	−0.15	0.57
Maharashtra	ari	Toilet	Improved	−0.03	0.29	−0.62	0.55
Maharashtra	ari	Location	Urban	0.22	0.38	−0.53	0.94
Maharashtra	ari	Cookign fuel	Solid	0.89	0.22	0.45	1.32
Maharashtra	ari	Household size	05-Oct	0.24	0.17	−0.08	0.57
Maharashtra	ari	Household size	10+	−0.37	0.23	−0.81	0.09
Maharashtra	ari	LLIN	Yes	0.00	0.26	−0.51	0.50
Maharashtra	ari	Access	Significant problem	0.10	0.16	−0.21	0.40
Maharashtra	ari	Vaccination	-	0.02	4.89	−9.51	9.78

Name	Response	Group	Level	Estimate	Error	lci	uci
Maharashtra	dia	Intercept	-	−2.63	2.70	−7.82	2.74
Maharashtra	dia	Age	1-2	−0.30	0.10	−0.50	−0.10
Maharashtra	dia	Age	2-3	−1.15	0.11	−1.38	−0.93
Maharashtra	dia	Age	3-4	−1.18	0.12	−1.41	−0.95
Maharashtra	dia	Age	4-5	−1.64	0.13	−1.89	−1.39
Maharashtra	dia	Month	May	−0.68	0.25	−1.18	−0.17
Maharashtra	dia	Month	June	−0.29	0.23	−0.76	0.16
Maharashtra	dia	Month	July	0.18	0.24	−0.28	0.66
Maharashtra	dia	Month	August	0.22	0.27	−0.30	0.74
Maharashtra	dia	Month	September	−1.43	0.36	−2.14	−0.74
Maharashtra	dia	Wealth	Poorer	0.30	0.15	0.01	0.59
Maharashtra	dia	Wealth	Middle	0.25	0.16	−0.06	0.56
Maharashtra	dia	Wealth	Richer	0.23	0.18	−0.14	0.58
Maharashtra	dia	Wealth	Richest	−0.00	0.22	−0.43	0.42
Maharashtra	dia	Mothers education	Primary	0.19	0.17	−0.14	0.51
Maharashtra	dia	Mothers education	Secondary	0.14	0.15	−0.14	0.43
Maharashtra	dia	Mothers education	Higher	0.49	0.19	0.12	0.86
Maharashtra	dia	Mothers age	20-30	−0.26	0.11	−0.47	−0.05
Maharashtra	dia	Mothers age	30-40	−0.13	0.16	−0.43	0.19
Maharashtra	dia	Sex	Female	−0.01	0.07	−0.15	0.13
Maharashtra	dia	Birth weight	Average	0.18	0.09	−0.00	0.36
Maharashtra	dia	Birth weight	Below Average	0.54	0.13	0.29	0.79
Maharashtra	dia	Breastfed	No	−0.54	0.09	−0.70	−0.36
Maharashtra	dia	Toilet	Unimproved	0.24	0.10	0.04	0.45
Maharashtra	dia	Toilet	Improved	0.12	0.15	−0.17	0.41
Maharashtra	dia	Location	Urban	−0.16	0.16	−0.47	0.17
Maharashtra	dia	Cookign fuel	Solid	0.01	0.11	−0.21	0.22
Maharashtra	dia	Household size	05-Oct	−0.04	0.09	−0.22	0.14
Maharashtra	dia	Household size	10+	0.06	0.13	−0.18	0.31
Maharashtra	dia	LLIN	Yes	0.23	0.16	−0.08	0.54
Maharashtra	dia	Access	Significant problem	0.39	0.08	0.23	0.55
Maharashtra	dia	Vaccination	-	0.07	5.03	−9.79	9.71

Name	Response	Group	Level	Estimate	Error	lci	uci
Maharashtra	fever	Intercept	-	−2.69	2.65	−8.00	2.57
Maharashtra	fever	Age	1-2	−0.21	0.10	−0.41	−0.03
Maharashtra	fever	Age	2-3	−0.79	0.10	−0.99	−0.59
Maharashtra	fever	Age	3-4	−0.54	0.10	−0.74	−0.34
Maharashtra	fever	Age	4-5	−0.71	0.10	−0.91	−0.51
Maharashtra	fever	Month	May	−0.69	0.24	−1.17	−0.22
Maharashtra	fever	Month	June	−0.75	0.23	−1.20	−0.32
Maharashtra	fever	Month	July	−0.64	0.24	−1.10	−0.17
Maharashtra	fever	Month	August	−0.76	0.26	−1.27	−0.26
Maharashtra	fever	Month	September	−1.32	0.31	−1.94	−0.71
Maharashtra	fever	Wealth	Poorer	0.48	0.14	0.20	0.75
Maharashtra	fever	Wealth	Middle	0.41	0.15	0.13	0.70
Maharashtra	fever	Wealth	Richer	0.65	0.16	0.33	0.97
Maharashtra	fever	Wealth	Richest	0.37	0.19	−0.00	0.73
Maharashtra	fever	Mothers education	Primary	0.81	0.15	0.52	1.11
Maharashtra	fever	Mothers education	Secondary	0.51	0.14	0.24	0.79
Maharashtra	fever	Mothers education	Higher	1.02	0.17	0.68	1.36
Maharashtra	fever	Mothers age	20-30	−0.04	0.10	−0.23	0.15
Maharashtra	fever	Mothers age	30-40	−0.24	0.14	−0.51	0.02
Maharashtra	fever	Sex	Female	−0.16	0.06	−0.28	−0.05
Maharashtra	fever	Birth weight	Average	0.16	0.08	0.01	0.31
Maharashtra	fever	Birth weight	Below Average	−0.24	0.11	−0.46	−0.01
Maharashtra	fever	Breastfed	No	−0.71	0.07	−0.84	−0.57
Maharashtra	fever	Toilet	Unimproved	0.25	0.08	0.09	0.41
Maharashtra	fever	Toilet	Improved	0.03	0.13	−0.23	0.28
Maharashtra	fever	Location	Urban	−0.11	0.16	−0.42	0.20
Maharashtra	fever	Cookign fuel	Solid	−0.04	0.10	−0.22	0.15
Maharashtra	fever	Household size	05-Oct	0.13	0.08	−0.02	0.28
Maharashtra	fever	Household size	10+	−0.02	0.11	−0.23	0.20
Maharashtra	fever	LLIN	Yes	−0.15	0.13	−0.42	0.11
Maharashtra	fever	Access	Significant problem	0.34	0.07	0.20	0.48
Maharashtra	fever	Vaccination	-	−0.03	4.94	−9.85	9.91

Name	Response	Group	Level	Estimate	Error	lci	uci
Arunachal Pradesh	wasted	Intercept	-	−3.60	2.65	−8.88	1.46
Arunachal Pradesh	wasted	Age	1-2	−0.13	1.15	−2.38	2.16
Arunachal Pradesh	wasted	Age	2-3	0.53	1.10	−1.55	2.79
Arunachal Pradesh	wasted	Age	3-4	0.28	1.12	−1.84	2.62
Arunachal Pradesh	wasted	Age	4-5	0.17	1.13	−2.01	2.51
Arunachal Pradesh	wasted	Month	May	0.01	1.28	−2.47	2.59
Arunachal Pradesh	wasted	Month	June	0.23	3.20	−6.67	6.08
Arunachal Pradesh	wasted	Month	August	0.61	1.24	−1.77	3.10
Arunachal Pradesh	wasted	Month	September	1.05	1.22	−1.26	3.53
Arunachal Pradesh	wasted	Month	October	0.89	1.16	−1.27	3.27
Arunachal Pradesh	wasted	Month	November	0.92	1.21	−1.32	3.38
Arunachal Pradesh	wasted	Wealth	Poorer	0.14	0.82	−1.47	1.78
Arunachal Pradesh	wasted	Wealth	Middle	−0.23	1.01	−2.24	1.74
Arunachal Pradesh	wasted	Wealth	Richer	−0.23	1.37	−3.00	2.41
Arunachal Pradesh	wasted	Wealth	Richest	−0.81	1.84	−4.62	2.59
Arunachal Pradesh	wasted	Mothers education	Primary	0.15	0.87	−1.57	1.87
Arunachal Pradesh	wasted	Mothers education	Secondary	−0.29	0.74	−1.73	1.15
Arunachal Pradesh	wasted	Mothers education	Higher	0.10	1.47	−2.90	2.88
Arunachal Pradesh	wasted	Mothers age	20-30	0.51	0.95	−1.21	2.50
Arunachal Pradesh	wasted	Mothers age	30-40	0.37	1.10	−1.71	2.57
Arunachal Pradesh	wasted	Mothers age	40+	−1.10	2.72	−7.26	3.50
Arunachal Pradesh	wasted	Sex	Female	−0.34	0.57	−1.47	0.78
Arunachal Pradesh	wasted	Birth weight	Average	0.51	0.80	−0.97	2.15
Arunachal Pradesh	wasted	Birth weight	Below Average	0.31	1.07	−1.82	2.38
Arunachal Pradesh	wasted	Breastfed	No	−0.04	0.63	−1.28	1.17
Arunachal Pradesh	wasted	Toilet	Unimproved	0.20	0.70	−1.20	1.54
Arunachal Pradesh	wasted	Toilet	Improved	−0.07	0.80	−1.69	1.42
Arunachal Pradesh	wasted	Location	Urban	−0.41	0.90	−2.24	1.28
Arunachal Pradesh	wasted	Cookign fuel	Solid	−0.09	0.96	−1.93	1.79
Arunachal Pradesh	wasted	Household size	05-Oct	−0.10	0.63	−1.32	1.18
Arunachal Pradesh	wasted	Household size	10+	−0.57	1.58	−4.02	2.21
Arunachal Pradesh	wasted	LLIN	Yes	0.02	0.64	−1.23	1.25
Arunachal Pradesh	wasted	Access	Significant problem	0.13	0.64	−1.10	1.41
Arunachal Pradesh	wasted	Vaccination	-	0.05	5.05	−9.99	10.18

Name	Response	Group	Level	Estimate	Error	lci	uci
Maharashtra	wasted	Intercept	-	−0.90	2.67	−6.18	4.29
Maharashtra	wasted	Age	1-2	−0.64	0.08	−0.80	−0.48
Maharashtra	wasted	Age	2-3	−0.67	0.08	−0.83	−0.51
Maharashtra	wasted	Age	3-4	−0.60	0.08	−0.76	−0.43
Maharashtra	wasted	Age	4-5	−0.71	0.08	−0.88	−0.55
Maharashtra	wasted	Month	May	0.31	0.17	−0.02	0.65
Maharashtra	wasted	Month	June	0.55	0.16	0.24	0.87
Maharashtra	wasted	Month	July	0.41	0.17	0.07	0.75
Maharashtra	wasted	Month	August	0.38	0.19	0.01	0.74
Maharashtra	wasted	Month	September	0.74	0.21	0.34	1.15
Maharashtra	wasted	Wealth	Poorer	−0.13	0.09	−0.31	0.05
Maharashtra	wasted	Wealth	Middle	−0.49	0.10	−0.69	−0.30
Maharashtra	wasted	Wealth	Richer	−0.55	0.12	−0.78	−0.33
Maharashtra	wasted	Wealth	Richest	−0.97	0.14	−1.24	−0.70
Maharashtra	wasted	Mothers education	Primary	−0.02	0.10	−0.22	0.17
Maharashtra	wasted	Mothers education	Secondary	0.06	0.09	−0.10	0.23
Maharashtra	wasted	Mothers education	Higher	−0.25	0.12	−0.49	−0.01
Maharashtra	wasted	Mothers age	20-30	0.26	0.07	0.12	0.41
Maharashtra	wasted	Mothers age	30-40	0.55	0.10	0.35	0.76
Maharashtra	wasted	Sex	Female	−0.00	0.05	−0.09	0.09
Maharashtra	wasted	Birth weight	Average	0.04	0.06	−0.07	0.16
Maharashtra	wasted	Birth weight	Below Average	0.39	0.09	0.21	0.56
Maharashtra	wasted	Breastfed	No	0.02	0.05	−0.09	0.12
Maharashtra	wasted	Toilet	Unimproved	−0.02	0.07	−0.15	0.11
Maharashtra	wasted	Toilet	Improved	0.06	0.10	−0.13	0.26
Maharashtra	wasted	Location	Urban	−0.11	0.11	−0.32	0.10
Maharashtra	wasted	Cookign fuel	Solid	−0.12	0.08	−0.27	0.03
Maharashtra	wasted	Household size	05-Oct	−0.19	0.06	−0.31	−0.07
Maharashtra	wasted	Household size	10+	−0.25	0.08	−0.42	−0.09
Maharashtra	wasted	LLIN	Yes	−0.20	0.12	−0.43	0.03
Maharashtra	wasted	Access	Significant problem	−0.13	0.06	−0.25	−0.02
Maharashtra	wasted	Vaccination	-	0.02	4.98	−9.61	9.89

Name	Response	Group	Level	Estimate	Error	lci	uci
Malawi	ari	Intercept	-	−5.99	2.53	−11.19	−1.34
Malawi	ari	Age	1-2	−0.18	0.25	−0.66	0.31
Malawi	ari	Age	2-3	0.18	0.24	−0.30	0.66
Malawi	ari	Age	3-4	−0.02	0.26	−0.53	0.48
Malawi	ari	Age	4-5	0.24	0.26	−0.28	0.75
Malawi	ari	Month	February	0.51	0.47	−0.46	1.40
Malawi	ari	Month	October	0.67	0.26	0.15	1.18
Malawi	ari	Month	November	0.36	0.23	−0.09	0.81
Malawi	ari	Month	December	−0.26	0.30	−0.86	0.32
Malawi	ari	Wealth	Poorer	−0.17	0.21	−0.57	0.24
Malawi	ari	Wealth	Middle	0.19	0.21	−0.21	0.60
Malawi	ari	Wealth	Richer	−0.07	0.22	−0.52	0.37
Malawi	ari	Wealth	Richest	−0.30	0.31	−0.92	0.31
Malawi	ari	Mothers education	Primary	0.18	0.22	−0.24	0.60
Malawi	ari	Mothers education	Secondary	0.20	0.28	−0.36	0.74
Malawi	ari	Mothers education	Higher	−1.22	1.04	−3.51	0.53
Malawi	ari	Mothers age	20-30	0.17	0.21	−0.24	0.60
Malawi	ari	Mothers age	30-40	0.33	0.24	−0.14	0.81
Malawi	ari	Mothers age	40+	0.62	0.39	−0.16	1.37
Malawi	ari	Sex	Female	−0.15	0.14	−0.43	0.12
Malawi	ari	Birth weight	Average	−0.08	0.16	−0.39	0.22
Malawi	ari	Birth weight	Below Average	0.13	0.20	−0.26	0.52
Malawi	ari	Breastfed	No	−1.03	0.22	−1.47	−0.62
Malawi	ari	Toilet	Unimproved	0.31	0.17	−0.03	0.65
Malawi	ari	Toilet	Improved	0.24	0.21	−0.17	0.63
Malawi	ari	Location	Urban	0.23	0.32	−0.40	0.84
Malawi	ari	Cookign fuel	Solid	1.96	1.55	−0.41	5.60
Malawi	ari	Household size	05-Oct	0.34	0.17	0.01	0.67
Malawi	ari	Household size	10+	−0.03	0.43	−0.92	0.80
Malawi	ari	LLIN	Yes	−0.12	0.15	−0.41	0.17
Malawi	ari	Access	Significant problem	−0.05	0.16	−0.37	0.27
Malawi	ari	Vaccination	-	0.14	2.96	−5.68	5.82

Name	Response	Group	Level	Estimate	Error	lci	uci
Malawi	dia	Intercept	-	1.62	1.43	−1.17	4.50
Malawi	dia	Age	1-2	−0.29	0.13	−0.54	−0.04
Malawi	dia	Age	2-3	−1.10	0.14	−1.37	−0.84
Malawi	dia	Age	3-4	−1.47	0.14	−1.75	−1.19
Malawi	dia	Age	4-5	−1.91	0.17	−2.24	−1.58
Malawi	dia	Month	February	0.42	0.28	−0.15	0.98
Malawi	dia	Month	October	0.30	0.16	−0.01	0.61
Malawi	dia	Month	November	0.07	0.13	−0.19	0.33
Malawi	dia	Month	December	−0.17	0.16	−0.49	0.15
Malawi	dia	Wealth	Poorer	−0.07	0.12	−0.30	0.16
Malawi	dia	Wealth	Middle	−0.21	0.13	−0.47	0.05
Malawi	dia	Wealth	Richer	−0.23	0.14	−0.50	0.05
Malawi	dia	Wealth	Richest	−0.14	0.18	−0.49	0.20
Malawi	dia	Mothers education	Primary	0.42	0.14	0.15	0.70
Malawi	dia	Mothers education	Secondary	0.21	0.17	−0.13	0.54
Malawi	dia	Mothers education	Higher	−0.47	0.45	−1.36	0.41
Malawi	dia	Mothers age	20-30	−0.05	0.11	−0.27	0.18
Malawi	dia	Mothers age	30-40	−0.58	0.14	−0.86	−0.29
Malawi	dia	Mothers age	40+	−0.75	0.27	−1.29	−0.23
Malawi	dia	Sex	Female	−0.32	0.08	−0.49	−0.16
Malawi	dia	Birth weight	Average	−0.15	0.09	−0.32	0.03
Malawi	dia	Birth weight	Below Average	−0.04	0.12	−0.28	0.21
Malawi	dia	Breastfed	No	−0.57	0.13	−0.82	−0.33
Malawi	dia	Toilet	Unimproved	0.05	0.11	−0.17	0.27
Malawi	dia	Toilet	Improved	0.02	0.13	−0.23	0.27
Malawi	dia	Location	Urban	0.60	0.18	0.24	0.96
Malawi	dia	Cookign fuel	Solid	0.01	0.45	−0.84	0.92
Malawi	dia	Household size	05-Oct	0.10	0.10	−0.09	0.29
Malawi	dia	Household size	10+	−0.09	0.25	−0.59	0.39
Malawi	dia	LLIN	Yes	−0.11	0.09	−0.29	0.06
Malawi	dia	Access	Significant problem	0.10	0.10	−0.09	0.29
Malawi	dia	Vaccination	-	−3.08	2.06	−7.16	0.93

Name	Response	Group	Level	Estimate	Error	lci	uci
Malawi	fever	Intercept	-	−0.35	1.31	−2.93	2.26
Malawi	fever	Age	1-2	0.06	0.13	−0.19	0.31
Malawi	fever	Age	2-3	−0.07	0.13	−0.32	0.18
Malawi	fever	Age	3-4	−0.20	0.13	−0.45	0.06
Malawi	fever	Age	4-5	−0.57	0.14	−0.85	−0.29
Malawi	fever	Month	February	0.39	0.27	−0.14	0.93
Malawi	fever	Month	October	0.71	0.15	0.42	1.00
Malawi	fever	Month	November	0.26	0.13	0.01	0.51
Malawi	fever	Month	December	0.11	0.15	−0.18	0.41
Malawi	fever	Wealth	Poorer	−0.15	0.11	−0.36	0.06
Malawi	fever	Wealth	Middle	−0.16	0.11	−0.39	0.06
Malawi	fever	Wealth	Richer	−0.11	0.12	−0.34	0.13
Malawi	fever	Wealth	Richest	−0.49	0.16	−0.81	−0.17
Malawi	fever	Mothers education	Primary	0.23	0.12	0.01	0.45
Malawi	fever	Mothers education	Secondary	0.19	0.15	−0.10	0.48
Malawi	fever	Mothers education	Higher	0.40	0.36	−0.31	1.10
Malawi	fever	Mothers age	20-30	0.10	0.11	−0.10	0.31
Malawi	fever	Mothers age	30-40	−0.06	0.13	−0.31	0.19
Malawi	fever	Mothers age	40+	0.00	0.22	−0.44	0.43
Malawi	fever	Sex	Female	0.05	0.07	−0.09	0.19
Malawi	fever	Birth weight	Average	−0.13	0.08	−0.28	0.03
Malawi	fever	Birth weight	Below Average	0.17	0.11	−0.04	0.38
Malawi	fever	Breastfed	No	−0.70	0.10	−0.91	−0.50
Malawi	fever	Toilet	Unimproved	0.14	0.10	−0.05	0.33
Malawi	fever	Toilet	Improved	0.18	0.11	−0.04	0.40
Malawi	fever	Location	Urban	0.12	0.17	−0.22	0.45
Malawi	fever	Cookign fuel	Solid	−0.22	0.39	−0.96	0.56
Malawi	fever	Household size	05-Oct	0.15	0.09	−0.02	0.32
Malawi	fever	Household size	10+	0.02	0.21	−0.40	0.42
Malawi	fever	LLIN	Yes	−0.24	0.08	−0.39	−0.09
Malawi	fever	Access	Significant problem	0.05	0.09	−0.12	0.22
Malawi	fever	Vaccination	-	−0.71	1.89	−4.41	3.00

Name	Response	Group	Level	Estimate	Error	lci	uci
Malawi	wasted	Intercept	-	1.51	2.54	−3.45	6.54
Malawi	wasted	Age	1-2	−0.28	0.32	−0.89	0.34
Malawi	wasted	Age	2-3	−0.62	0.34	−1.30	0.04
Malawi	wasted	Age	3-4	−0.94	0.36	−1.66	−0.24
Malawi	wasted	Age	4-5	−0.77	0.38	−1.52	−0.04
Malawi	wasted	Month	February	0.79	0.53	−0.28	1.80
Malawi	wasted	Month	October	−0.65	0.38	−1.42	0.08
Malawi	wasted	Month	November	−1.08	0.34	−1.75	−0.43
Malawi	wasted	Month	December	−0.03	0.33	−0.68	0.61
Malawi	wasted	Wealth	Poorer	−0.16	0.31	−0.78	0.44
Malawi	wasted	Wealth	Middle	0.05	0.31	−0.57	0.66
Malawi	wasted	Wealth	Richer	−0.37	0.36	−1.07	0.33
Malawi	wasted	Wealth	Richest	−0.34	0.44	−1.24	0.51
Malawi	wasted	Mothers education	Primary	0.34	0.37	−0.35	1.07
Malawi	wasted	Mothers education	Secondary	0.56	0.44	−0.30	1.44
Malawi	wasted	Mothers education	Higher	1.02	0.81	−0.60	2.57
Malawi	wasted	Mothers age	20-30	−0.44	0.27	−0.96	0.09
Malawi	wasted	Mothers age	30-40	−0.50	0.34	−1.16	0.15
Malawi	wasted	Mothers age	40+	0.25	0.53	−0.83	1.26
Malawi	wasted	Sex	Female	−0.70	0.21	−1.11	−0.30
Malawi	wasted	Birth weight	Average	0.33	0.25	−0.17	0.82
Malawi	wasted	Birth weight	Below Average	1.06	0.30	0.47	1.66
Malawi	wasted	Breastfed	No	−0.09	0.30	−0.68	0.48
Malawi	wasted	Toilet	Unimproved	0.05	0.28	−0.49	0.58
Malawi	wasted	Toilet	Improved	0.25	0.32	−0.39	0.85
Malawi	wasted	Location	Urban	0.26	0.41	−0.57	1.05
Malawi	wasted	Cookign fuel	Solid	−0.81	0.75	−2.23	0.72
Malawi	wasted	Household size	05-Oct	0.08	0.24	−0.40	0.57
Malawi	wasted	Household size	10+	1.34	0.45	0.44	2.20
Malawi	wasted	LLIN	Yes	−0.24	0.22	−0.68	0.18
Malawi	wasted	Access	Significant problem	0.16	0.25	−0.32	0.66
Malawi	wasted	Vaccination	-	−6.57	3.58	−13.57	0.45

Name	Response	Group	Level	Estimate	Error	lci	uci
Mali	ari	Intercept	-	−9.03	1.64	−12.38	−5.90
Mali	ari	Age	1-2	0.26	0.30	−0.33	0.86
Mali	ari	Age	2-3	0.06	0.32	−0.56	0.70
Mali	ari	Age	3-4	−0.14	0.34	−0.82	0.53
Mali	ari	Age	4-5	−0.19	0.36	−0.90	0.53
Mali	ari	Month	September	−0.20	0.31	−0.82	0.42
Mali	ari	Month	October	−0.20	0.36	−0.92	0.50
Mali	ari	Month	November	−1.25	1.07	−3.54	0.58
Mali	ari	Wealth	Poorer	−0.32	0.26	−0.82	0.18
Mali	ari	Wealth	Middle	−0.55	0.31	−1.16	0.06
Mali	ari	Wealth	Richer	0.27	0.36	−0.45	0.96
Mali	ari	Wealth	Richest	0.55	0.53	−0.47	1.59
Mali	ari	Mothers education	Primary	−0.80	0.36	−1.55	−0.15
Mali	ari	Mothers education	Secondary	−0.30	0.31	−0.94	0.29
Mali	ari	Mothers education	Higher	−0.35	0.89	−2.29	1.20
Mali	ari	Mothers age	20-30	0.13	0.30	−0.44	0.74
Mali	ari	Mothers age	30-40	−0.05	0.33	−0.68	0.61
Mali	ari	Mothers age	40+	0.52	0.46	−0.42	1.37
Mali	ari	Sex	Female	0.05	0.17	−0.29	0.40
Mali	ari	Birth weight	Average	−0.40	0.19	−0.77	−0.03
Mali	ari	Birth weight	Below Average	−0.39	0.26	−0.92	0.11
Mali	ari	Breastfed	No	0.13	0.22	−0.31	0.56
Mali	ari	Toilet	Unimproved	0.08	0.23	−0.37	0.52
Mali	ari	Toilet	Improved	−0.12	0.23	−0.56	0.32
Mali	ari	Location	Urban	−1.56	0.49	−2.56	−0.61
Mali	ari	Cookign fuel	Solid	0.41	1.02	−1.32	2.64
Mali	ari	Household size	05-Oct	0.09	0.27	−0.41	0.63
Mali	ari	Household size	10+	0.15	0.31	−0.46	0.76
Mali	ari	LLIN	Yes	0.28	0.26	−0.20	0.79
Mali	ari	Access	Significant problem	0.06	0.19	−0.31	0.43
Mali	ari	Vaccination	-	10.56	3.00	4.61	16.37
