# Supplementary material for: Health inequities and clustering of fever, acute respiratory infection, diarrhoea and wasting in children under five in low- and middle-income countries: a Demographic and Health Surveys analysis
Source: BMC Med. 2021 Jun 24;19:144. doi: 10.1186/s12916-021-02018-0 (PMC8223394; doi:10.1186/s12916-021-02018-0)

Health inequities and clustering of fever, acute respiratory infection, diarrhoea and wasting in children under five in low- and middle-income countries: A Demographic and Health Surveys analysis.

Supplementary Information: Spatial distribution of adjusted prevalence (AP)

Note, APs are higher than average observed prevalence in the raw data as we present those for the reference group, which is often high risk (for example the reference group include the Poorest households, no vaccination, lowest levels of mother's education etc). We do not show uncertainty in these estimates, which is some cases is large.

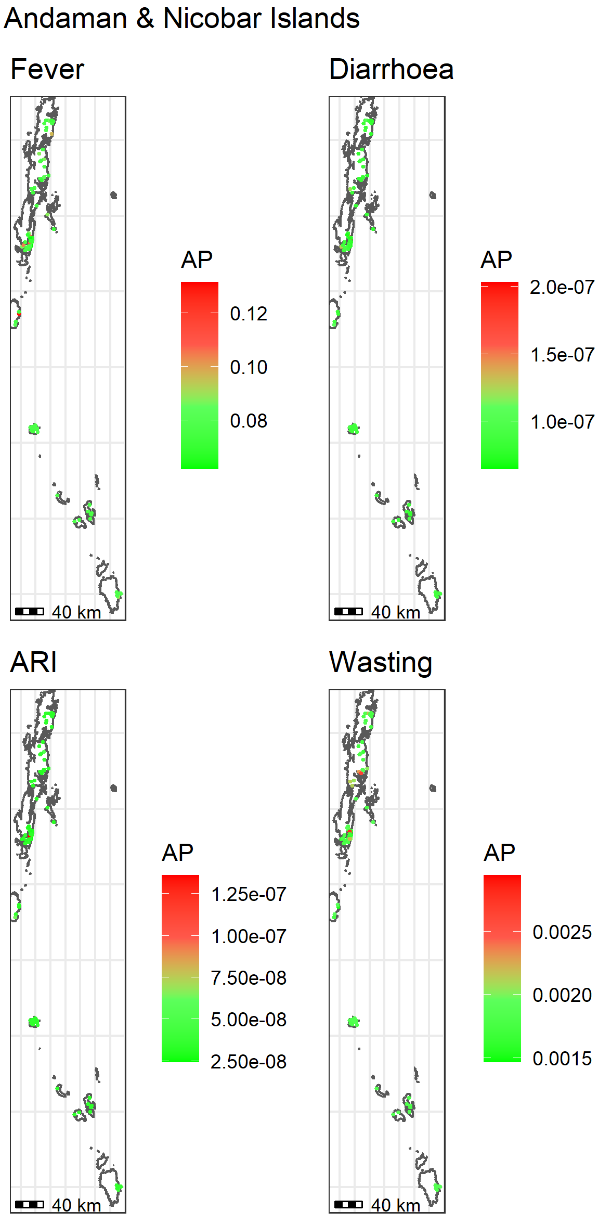

Burundi

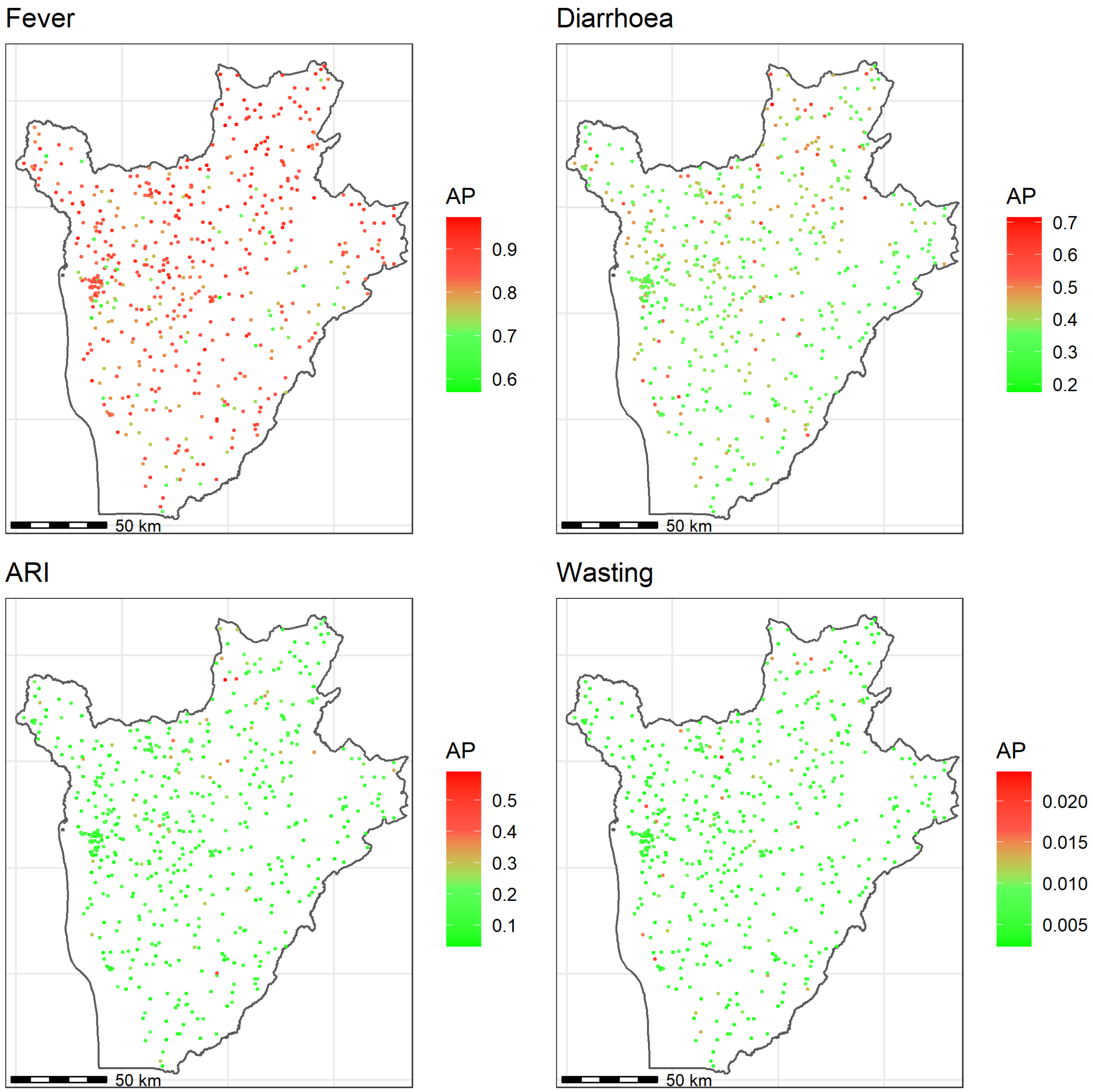
Fever
AP
0.9
0.8
0.7
0.6
50 km
Diarrhoea
AP
0.7
0.6
0.5
0.4
0.3
0.2
50 km
ARI
AP
0.5
0.4
0.3
0.2
0.1
50 km
Wasting
AP
0.020
0.015
0.010
0.005
50 km

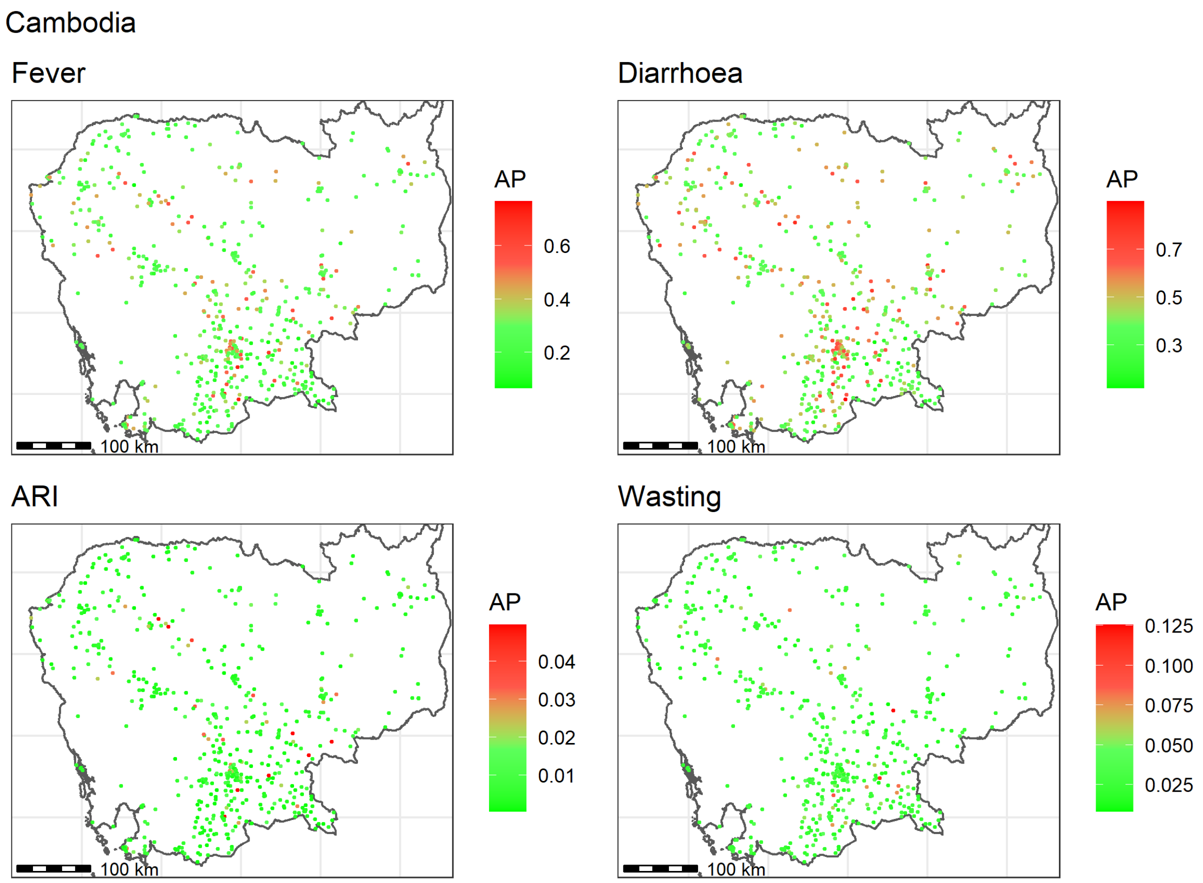
Cambodia
Fever
AP
0.6
0.4
0.2
100 km
Diarrhoea
AP
0.7
0.5
0.3
100 km
ARI
AP
0.04
0.03
0.02
0.01
100 km
Wasting
AP
0.125
0.100
0.075
0.050
0.025
100 km

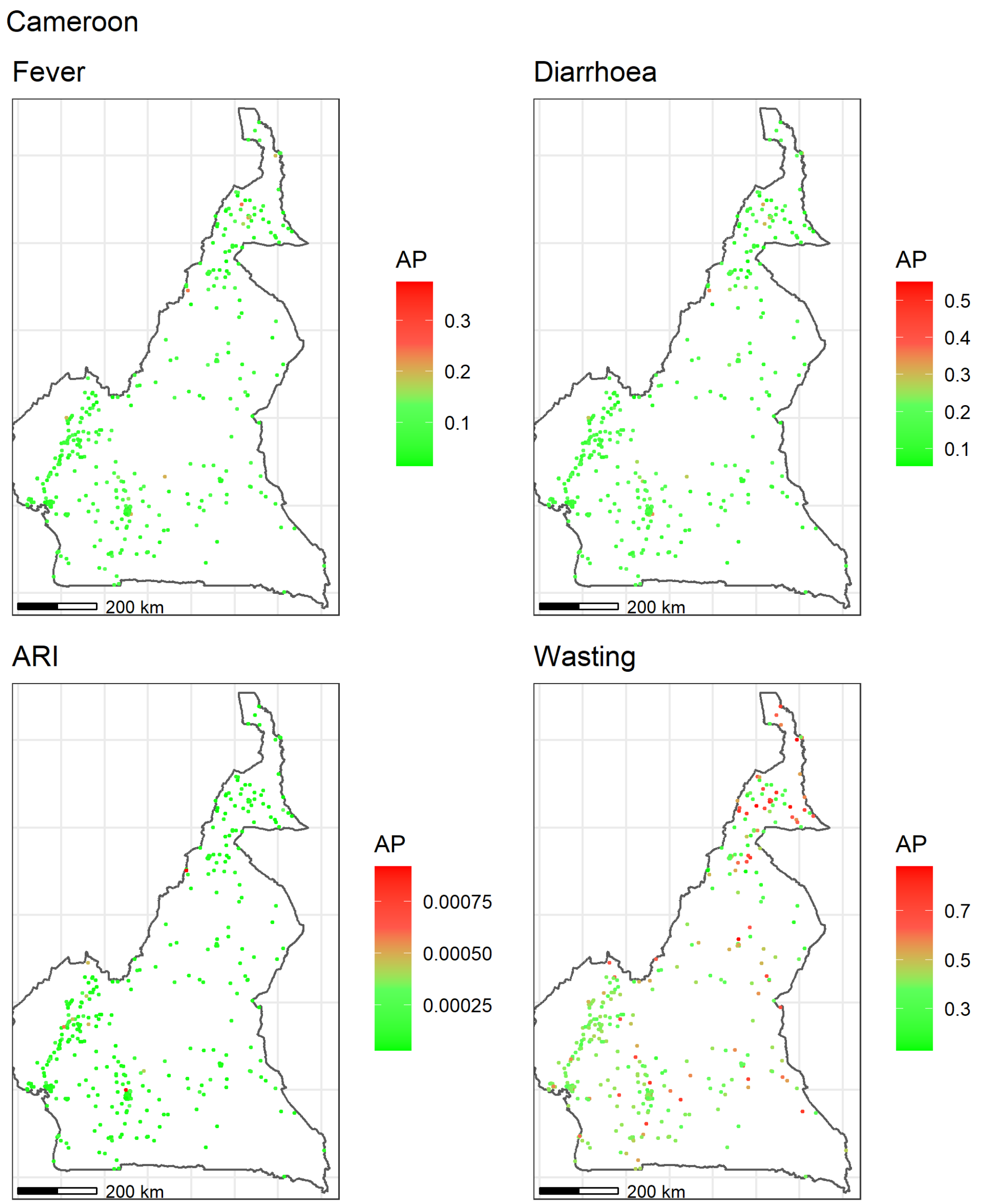
Cameroon
Fever
AP
0.3
0.2
0.1
200 km
Diarrhoea
AP
0.5
0.4
0.3
0.2
0.1
200 km
ARI
AP
0.00075
0.00050
0.00025
200 km
Wasting
AP
0.7
0.5
0.3
200 km

Chad

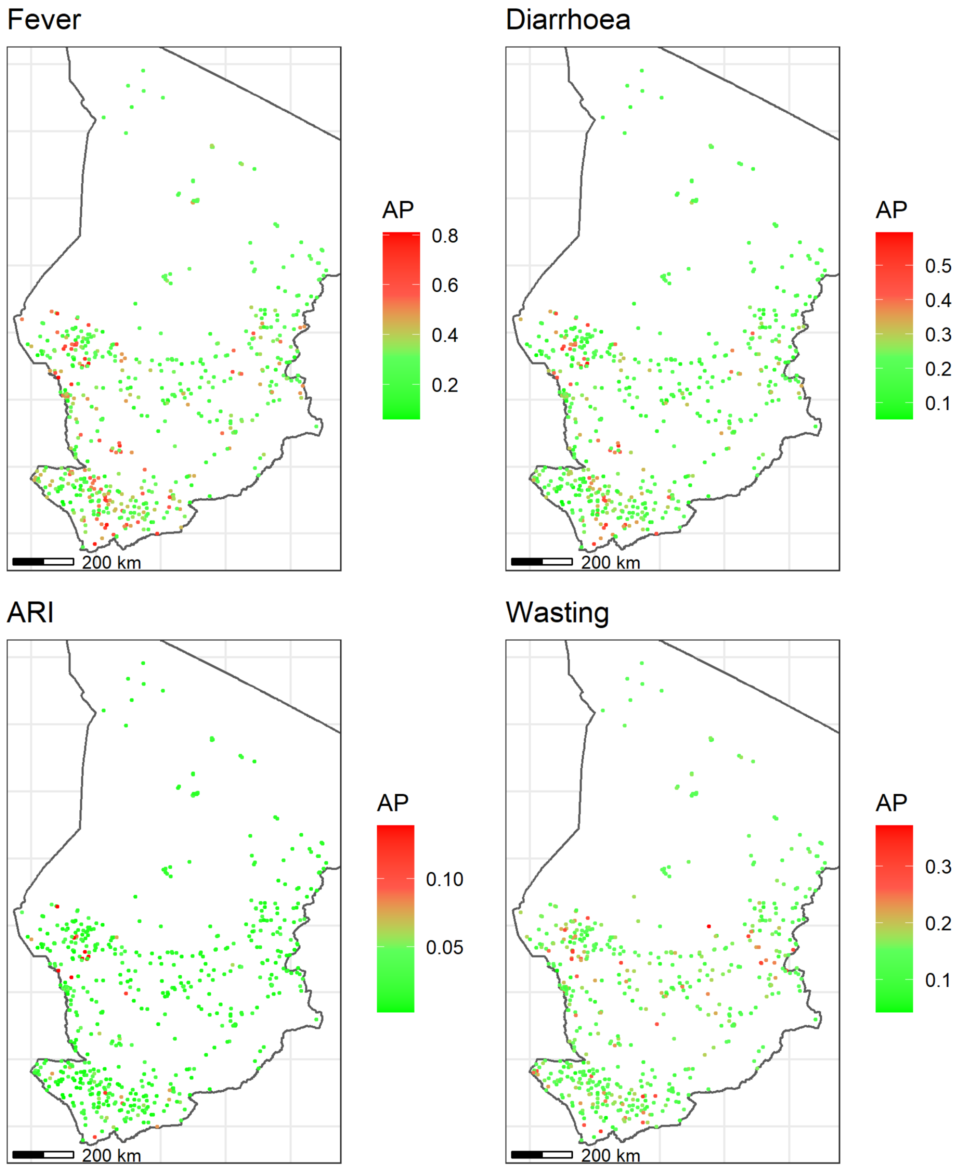

Fever
AP
0.8
0.6
0.4
0.2
200 km
Diarrhoea
AP
0.5
0.4
0.3
0.2
0.1
200 km
ARI
AP
0.10
0.05
200 km
Wasting
AP
0.3
0.2
0.1
200 km

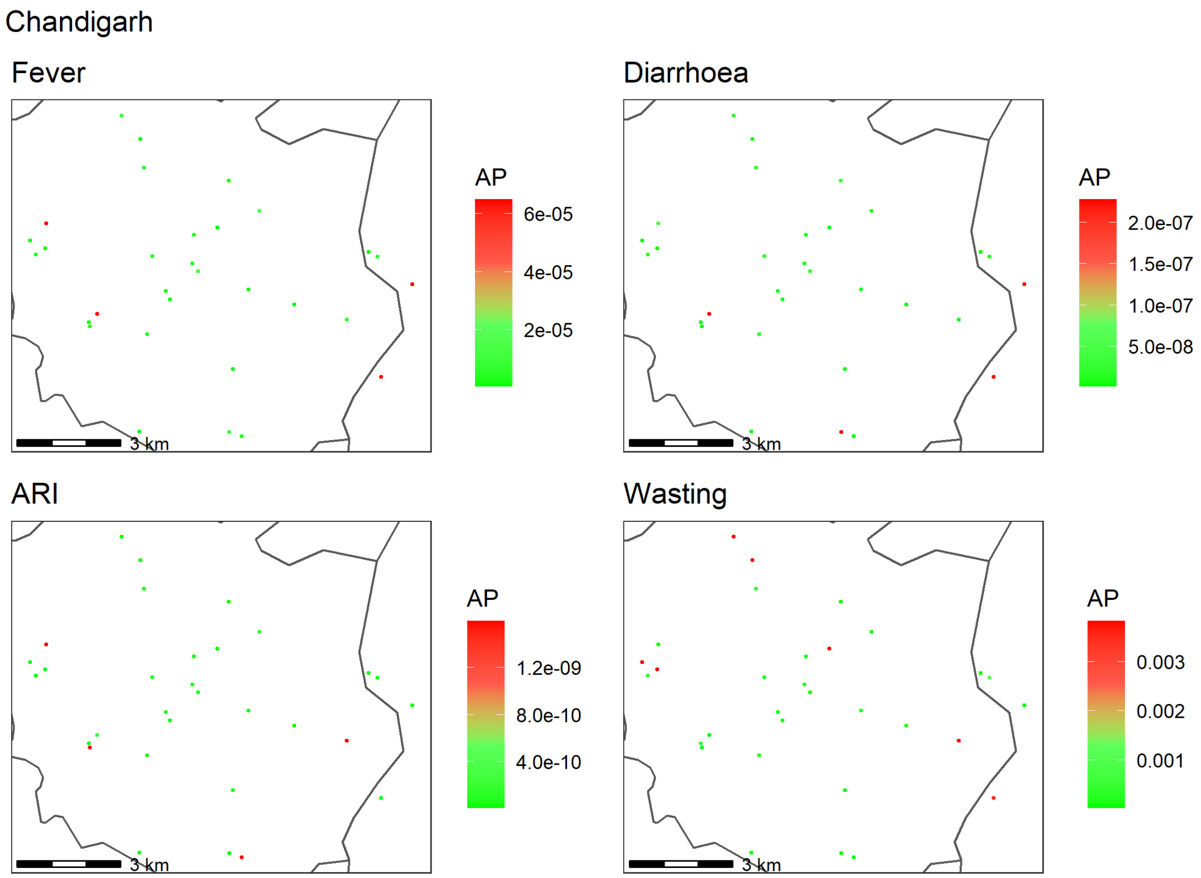
Chandigarh
Fever
AP
6e-05
4e-05
2e-05
3 km
Diarrhoea
AP
2.0e-07
1.5e-07
1.0e-07
5.0e-08
3 km
ARI
AP
1.2e-09
8.0e-10
4.0e-10
3 km
Wasting
AP
0.003
0.002
0.001
3 km

Chhattisgarh

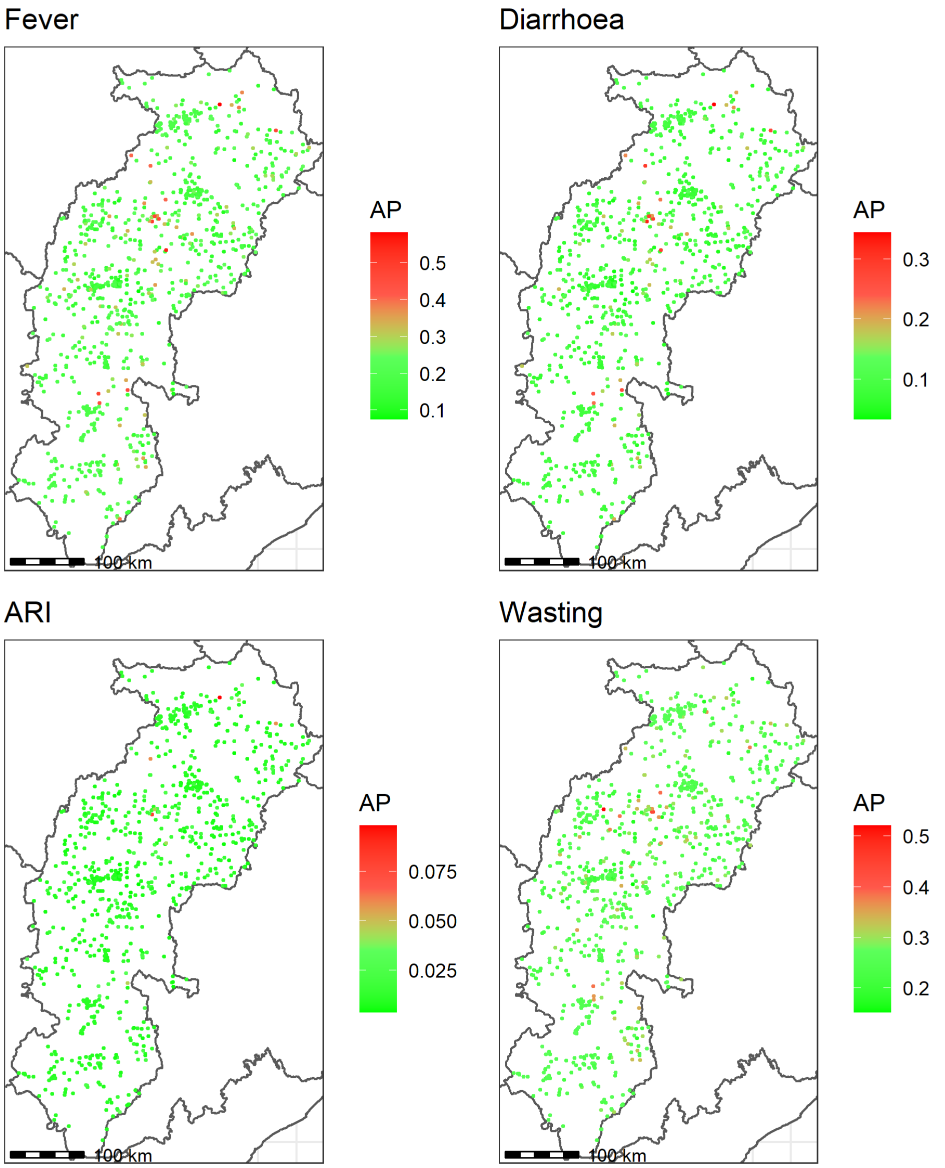

Comoros

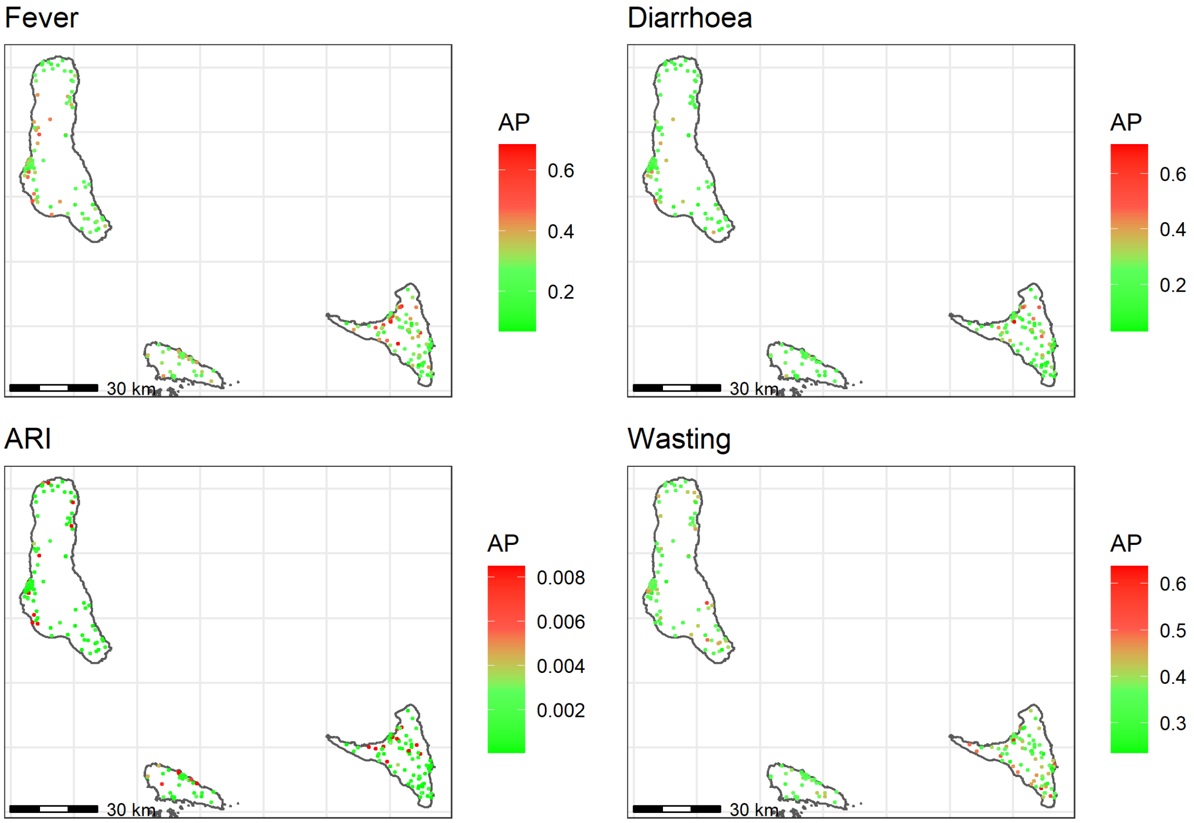

Fever
AP
0.6
0.4
0.2
30 km
Diarrhoea
AP
0.6
0.4
0.2
30 km
ARI
AP
0.008
0.006
0.004
0.002
30 km
Wasting
AP
0.6
0.5
0.4
0.3
30 km

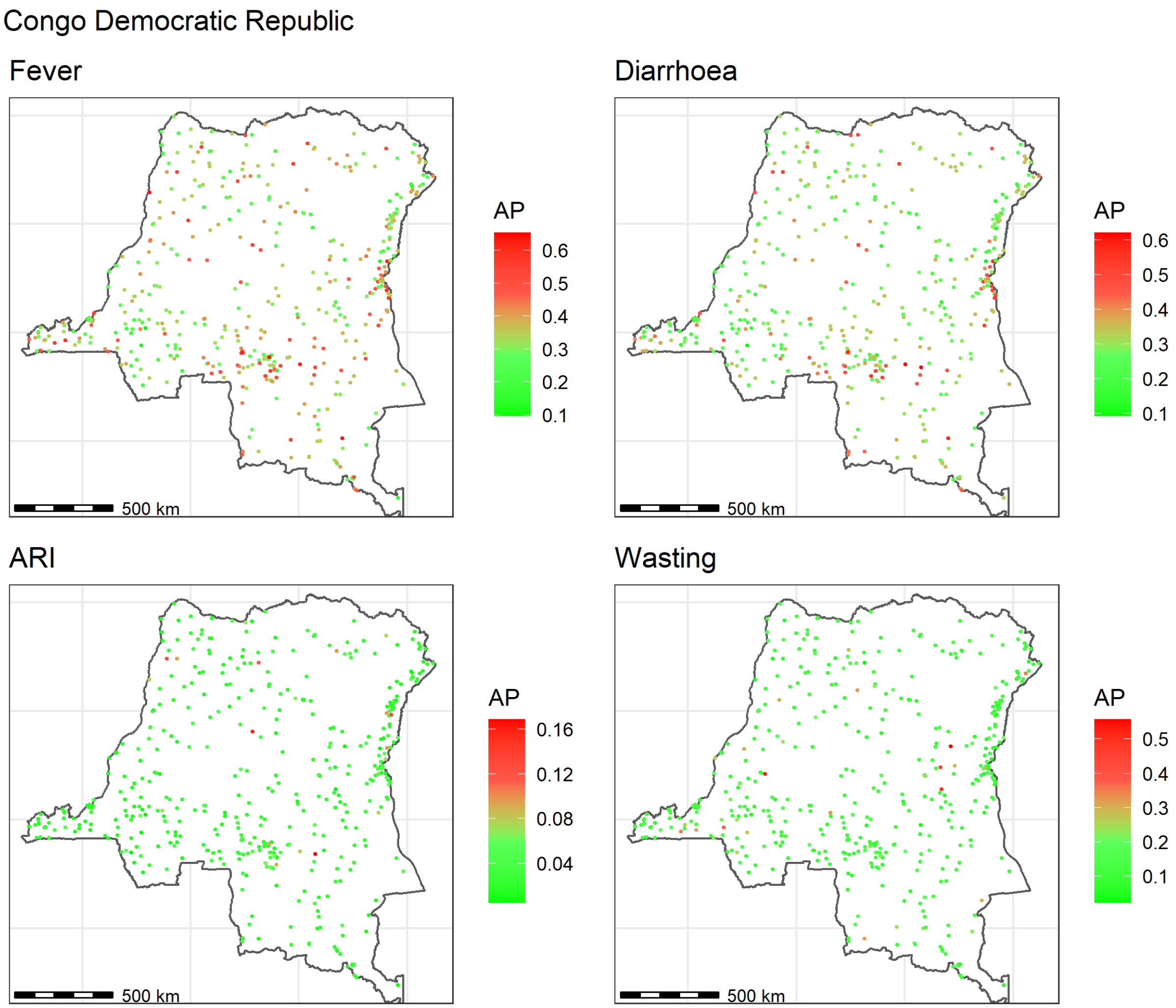
Congo Democratic Republic
Fever
AP
0.6
0.5
0.4
0.3
0.2
0.1
500 km
Diarrhoea
AP
0.6
0.5
0.4
0.3
0.2
0.1
500 km
ARI
AP
0.16
0.12
0.08
0.04
500 km
Wasting
AP
0.5
0.4
0.3
0.2
0.1
500 km

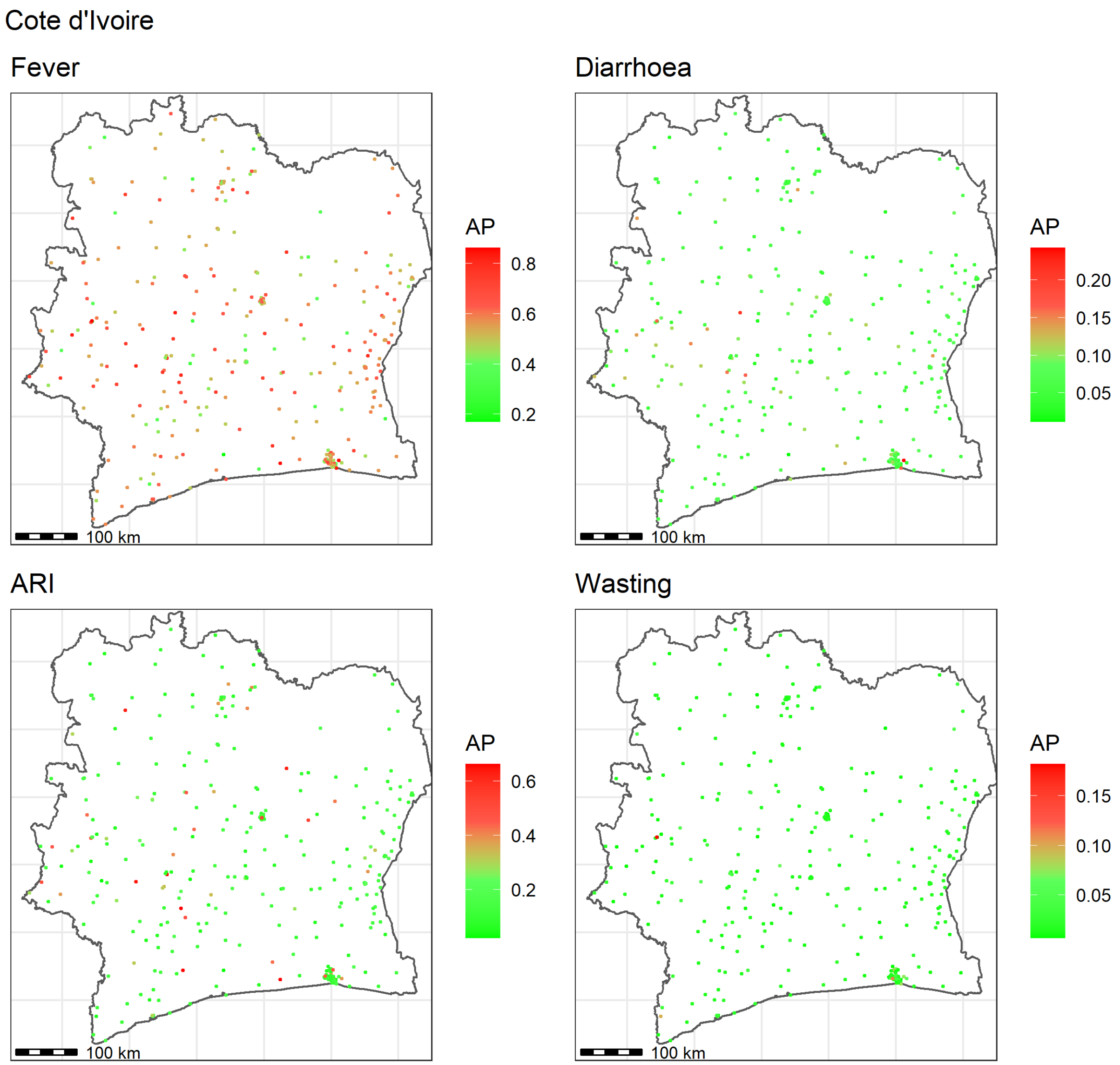
Cote d'Ivoire
Fever
AP
0.8
0.6
0.4
0.2
100 km
Diarrhoea
AP
0.20
0.15
0.10
0.05
100 km
ARI
AP
0.6
0.4
0.2
100 km
Wasting
AP
0.15
0.10
0.05
100 km

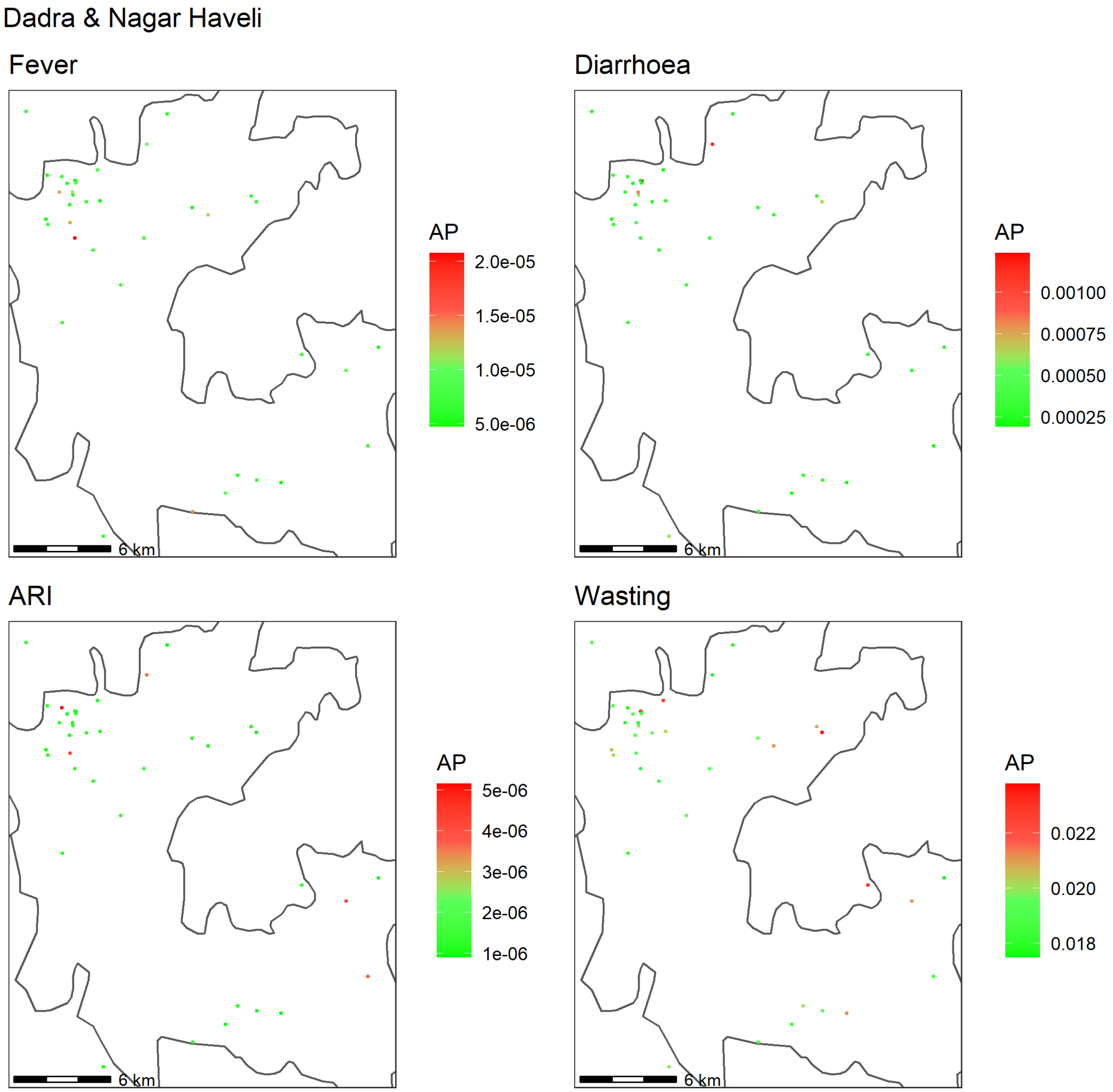
Dadra & Nagar Haveli
Fever
AP
2.0e-05
1.5e-05
1.0e-05
5.0e-06
6 km
Diarrhoea
AP
0.00100
0.00075
0.00050
0.00025
6 km
ARI
AP
5e-06
4e-06
3e-06
2e-06
1e-06
6 km
Wasting
AP
0.022
0.020
0.018
6 km

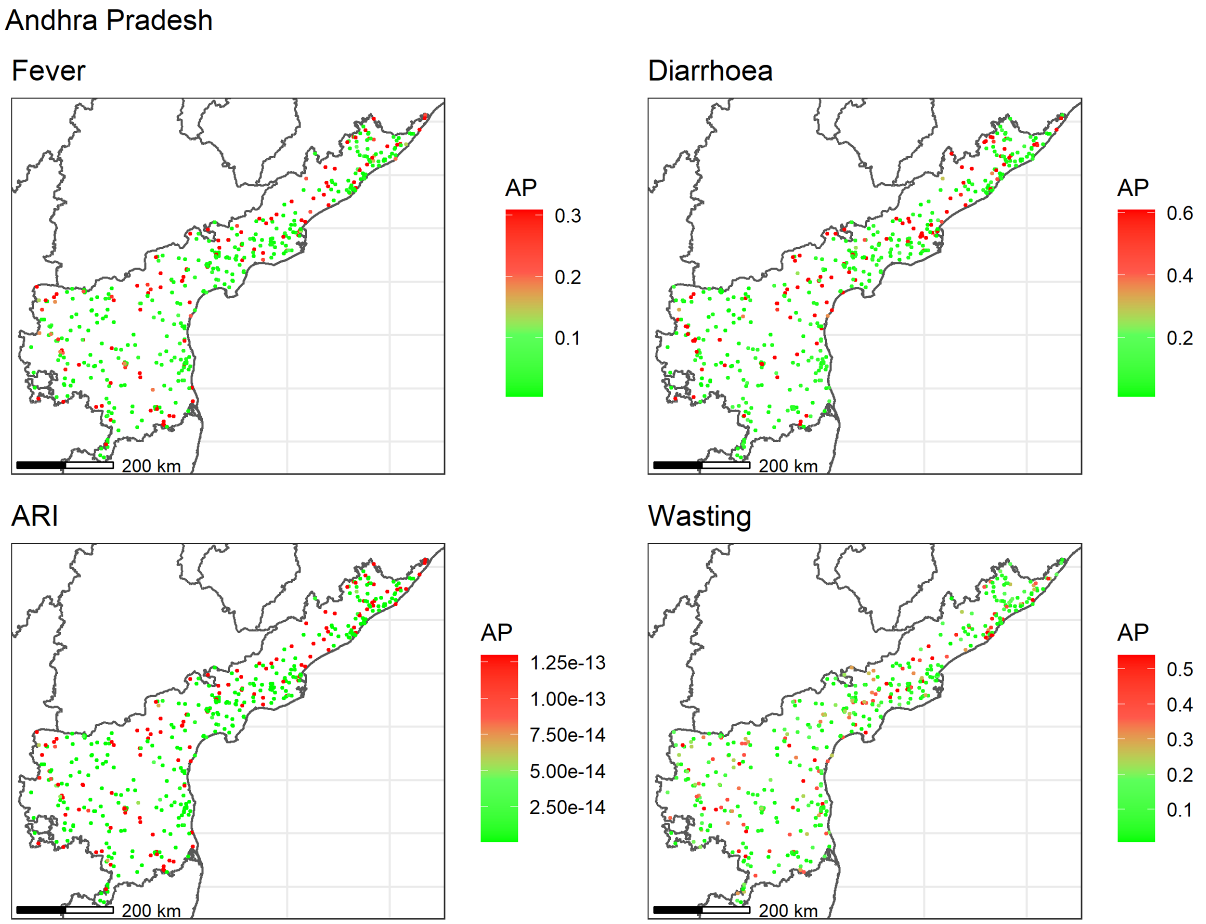
Andhra Pradesh
Fever
AP
0.3
0.2
0.1
200 km
Diarrhoea
AP
0.6
0.4
0.2
200 km
ARI
AP
1.25e-13
1.00e-13
7.50e-14
5.00e-14
2.50e-14
200 km
Wasting
AP
0.5
0.4
0.3
0.2
0.1
200 km

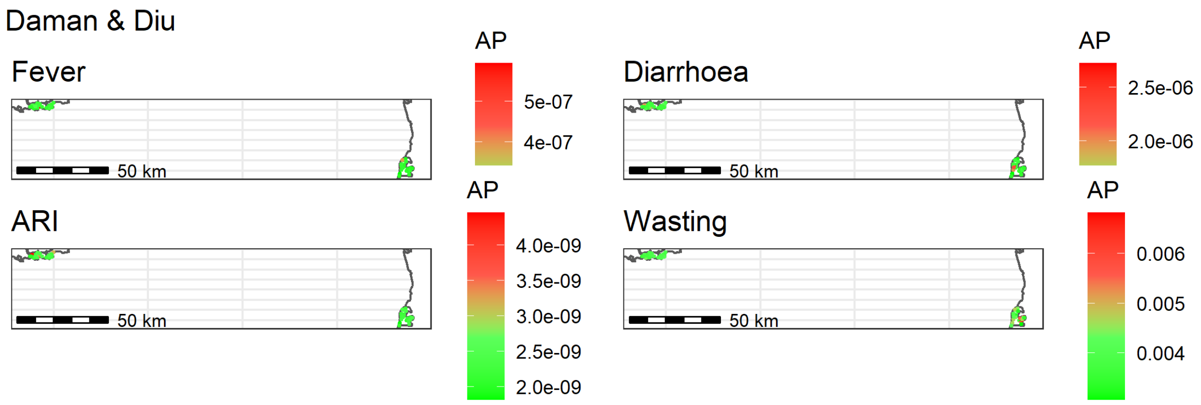

Daman & Diu
Fever
AP
5e-07
4e-07
50 km
Diarrhoea
AP
2.5e-06
2.0e-06
50 km
ARI
AP
4.0e-09
3.5e-09
3.0e-09
2.5e-09
2.0e-09
50 km
Wasting
AP
0.006
0.005
0.004
50 km

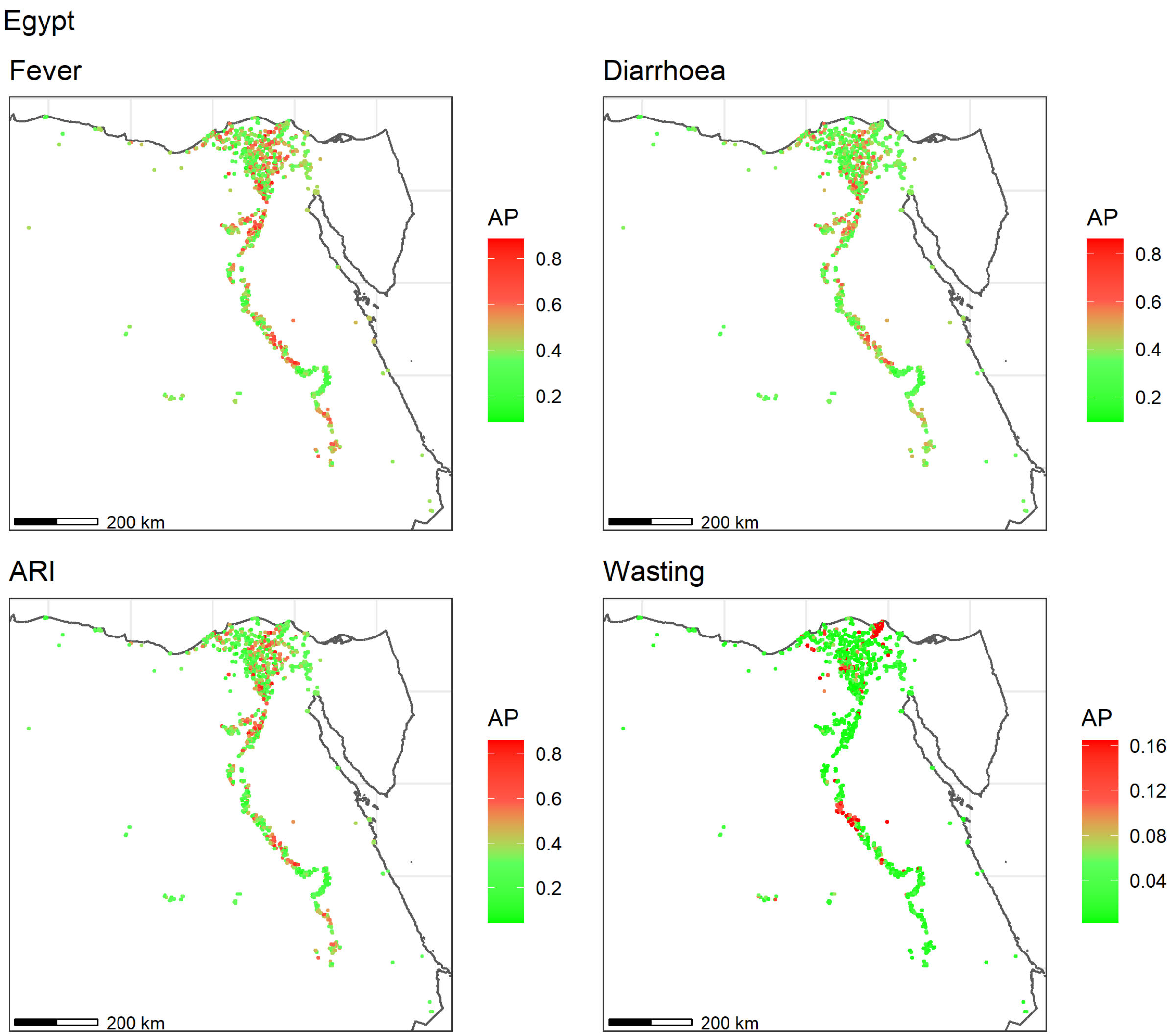
Egypt
Fever
Diarrhoea
ARI
Wasting
AP
0.8
0.6
0.4
0.2
0.16
0.12
0.08
0.04
200 km

Ethiopia

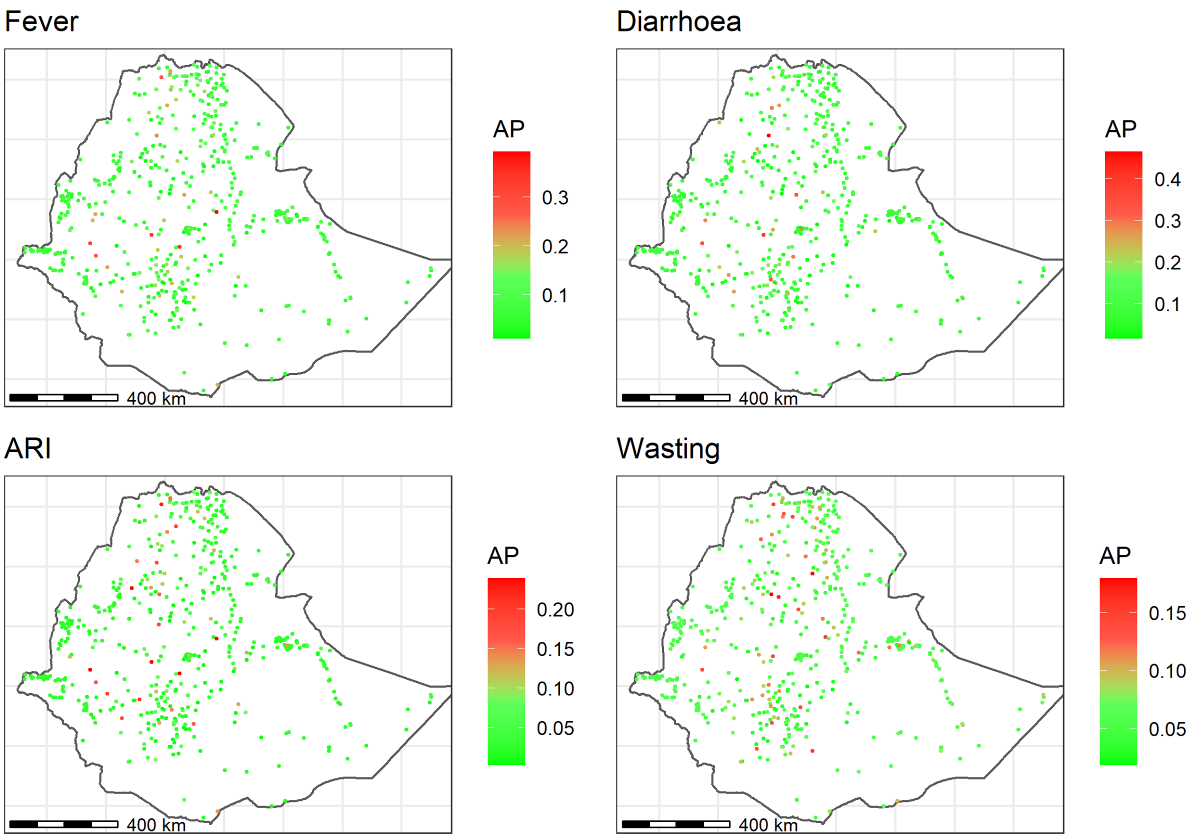

Fever
AP
0.3
0.2
0.1
400 km
Diarrhoea
AP
0.4
0.3
0.2
0.1
400 km
ARI
AP
0.20
0.15
0.10
0.05
400 km
Wasting
AP
0.15
0.10
0.05
400 km

Gabon

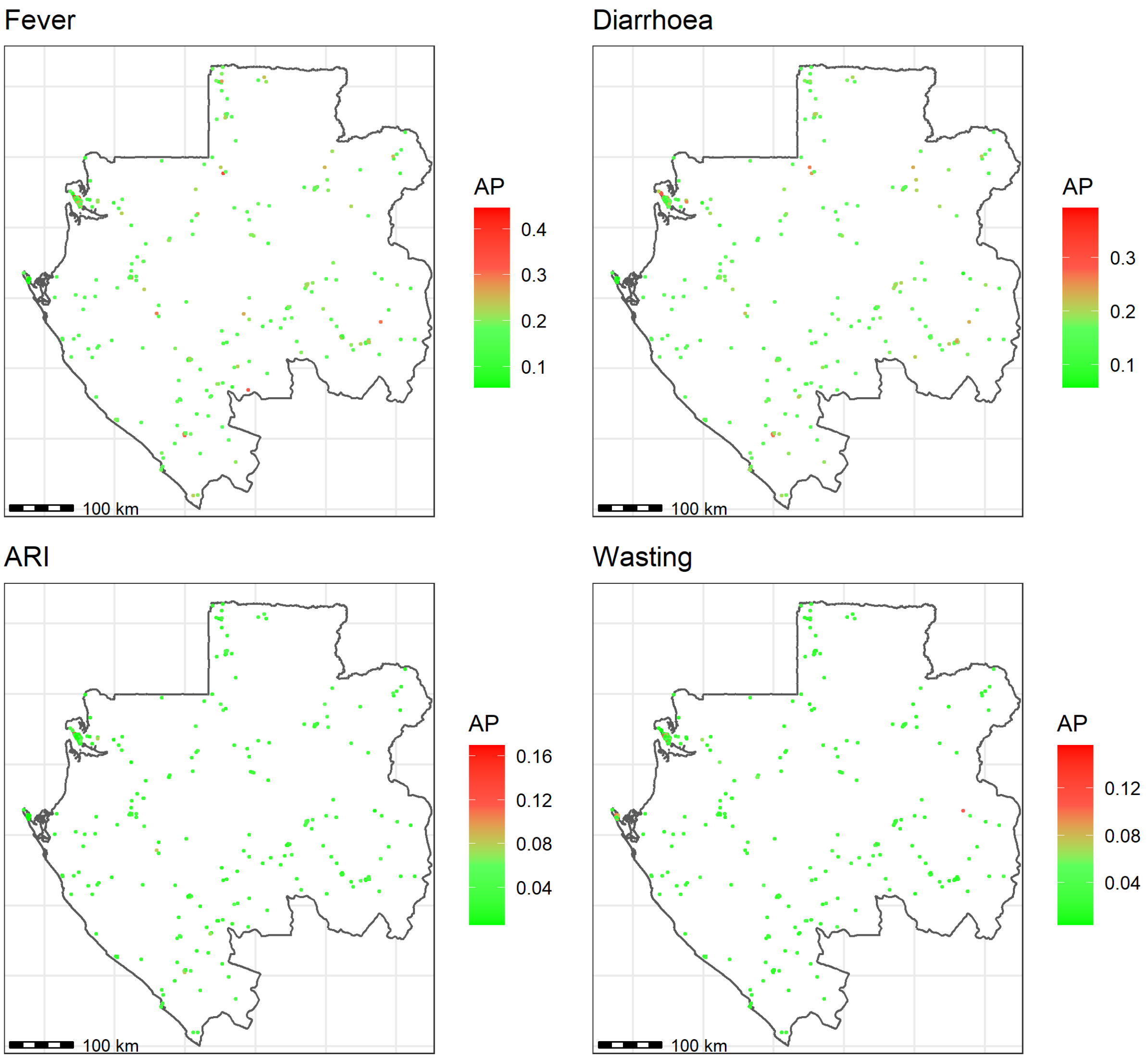
Fever
AP
0.4
0.3
0.2
0.1
100 km
Diarrhoea
AP
0.3
0.2
0.1
100 km
ARI
AP
0.16
0.12
0.08
0.04
100 km
Wasting
AP
0.12
0.08
0.04
100 km

Ghana

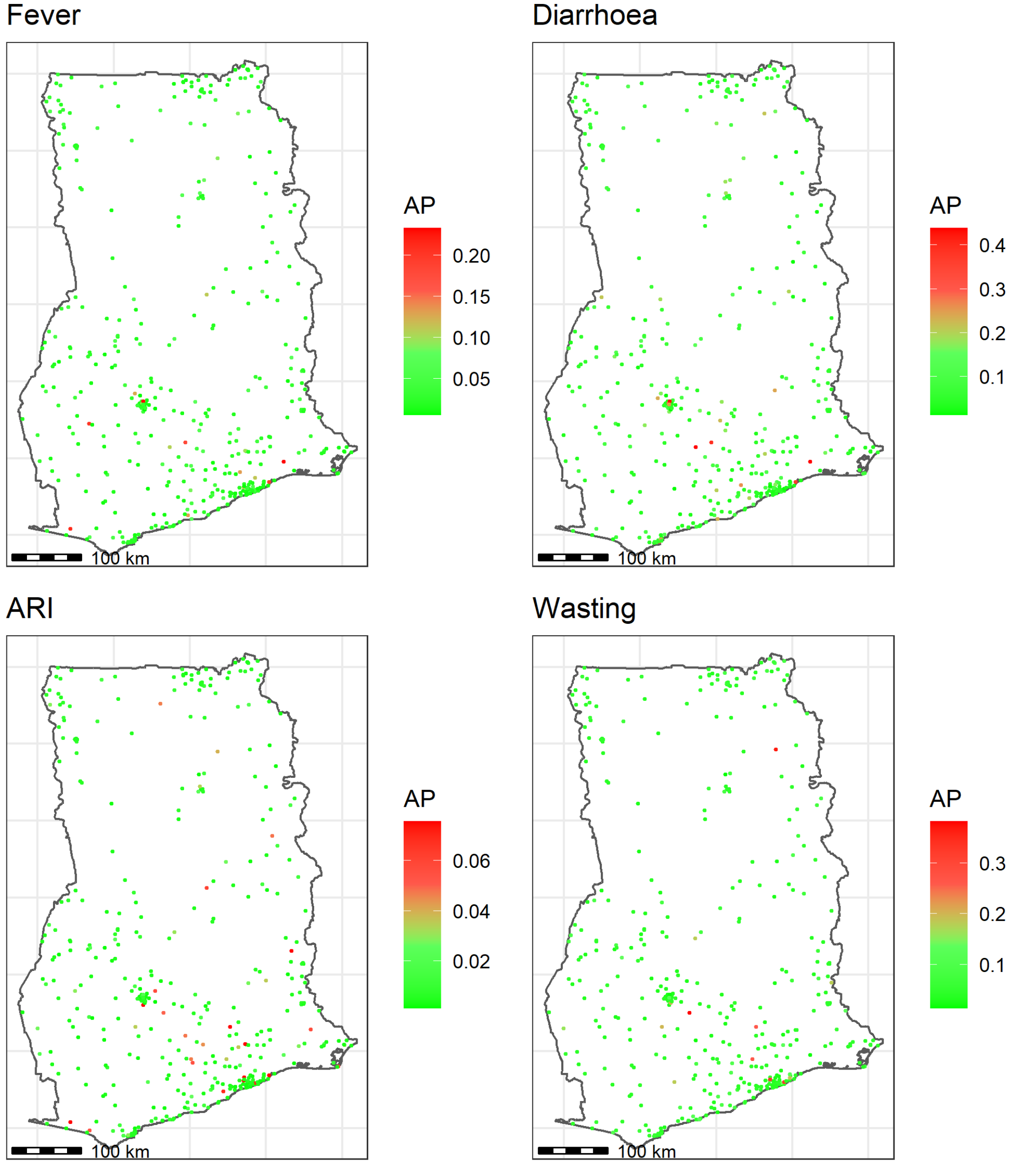
Fever
100 km
AP
0.20
0.15
0.10
0.05
Diarrhoea
100 km
AP
0.4
0.3
0.2
0.1
ARI
100 km
AP
0.06
0.04
0.02
Wasting
100 km
AP
0.3
0.2
0.1

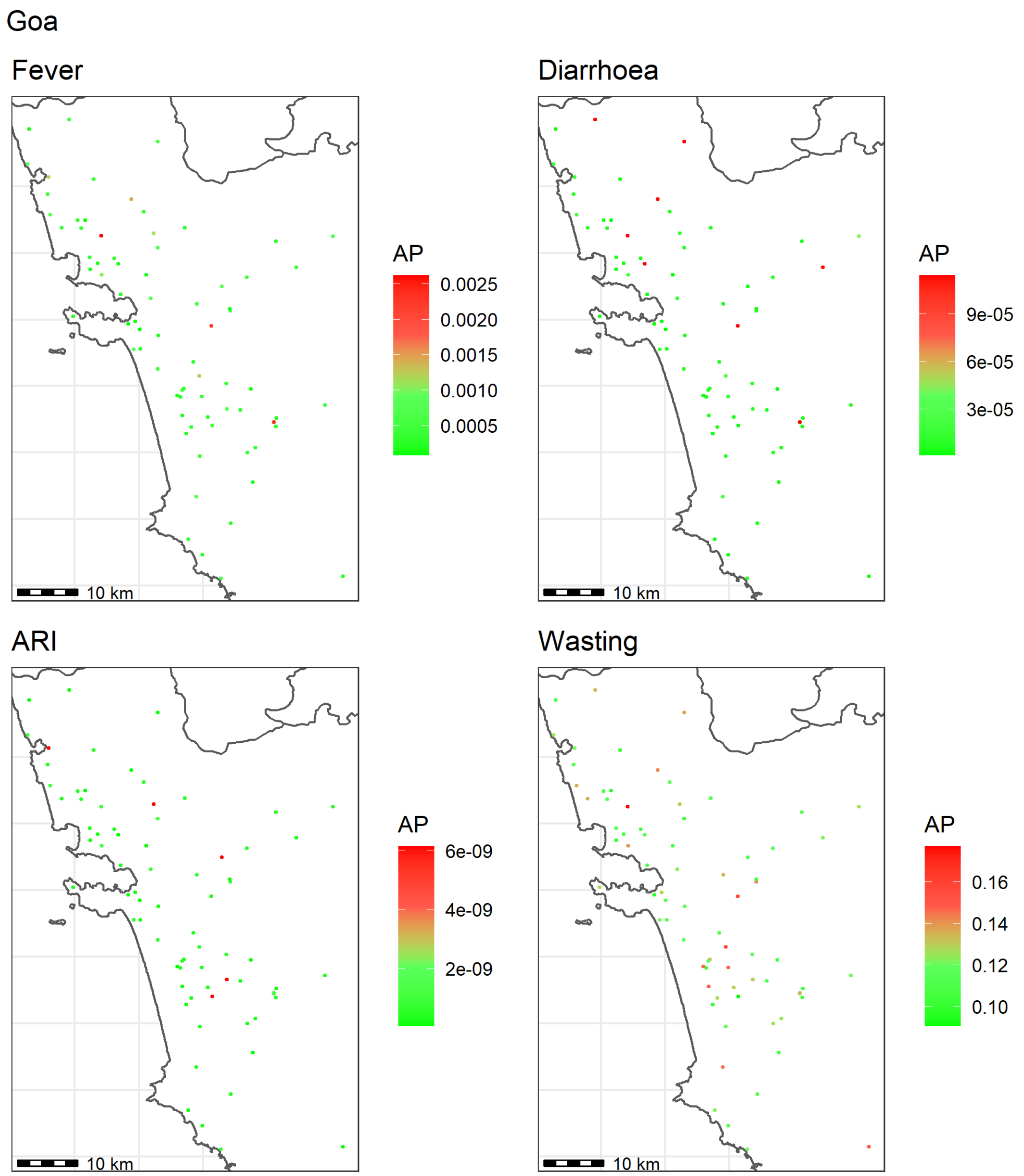
Goa
Fever
AP
0.0025
0.0020
0.0015
0.0010
0.0005
10 km
Diarrhoea
AP
9e-05
6e-05
3e-05
10 km
ARI
AP
6e-09
4e-09
2e-09
10 km
Wasting
AP
0.16
0.14
0.12
0.10
10 km

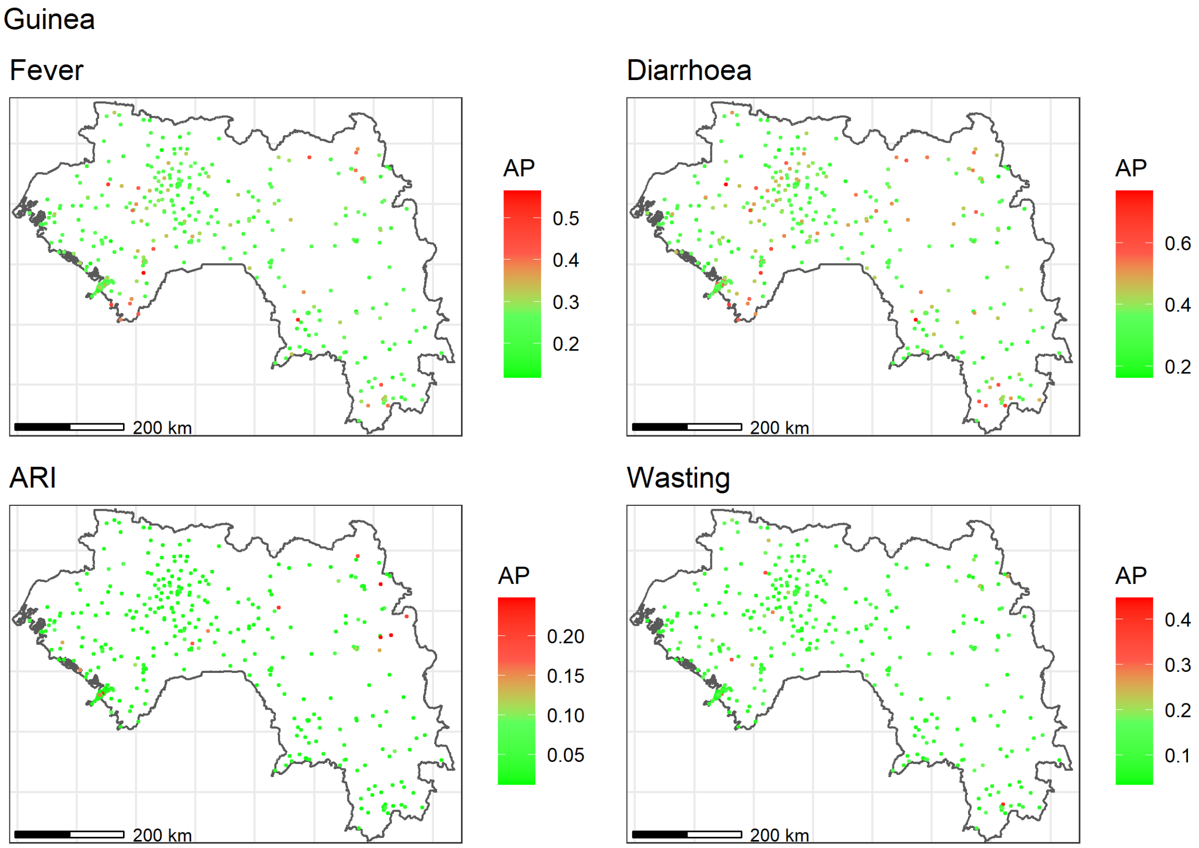
Guinea
Fever
AP
0.5
0.4
0.3
0.2
200 km
Diarrhoea
AP
0.6
0.4
0.2
200 km
ARI
AP
0.20
0.15
0.10
0.05
200 km
Wasting
AP
0.4
0.3
0.2
0.1
200 km

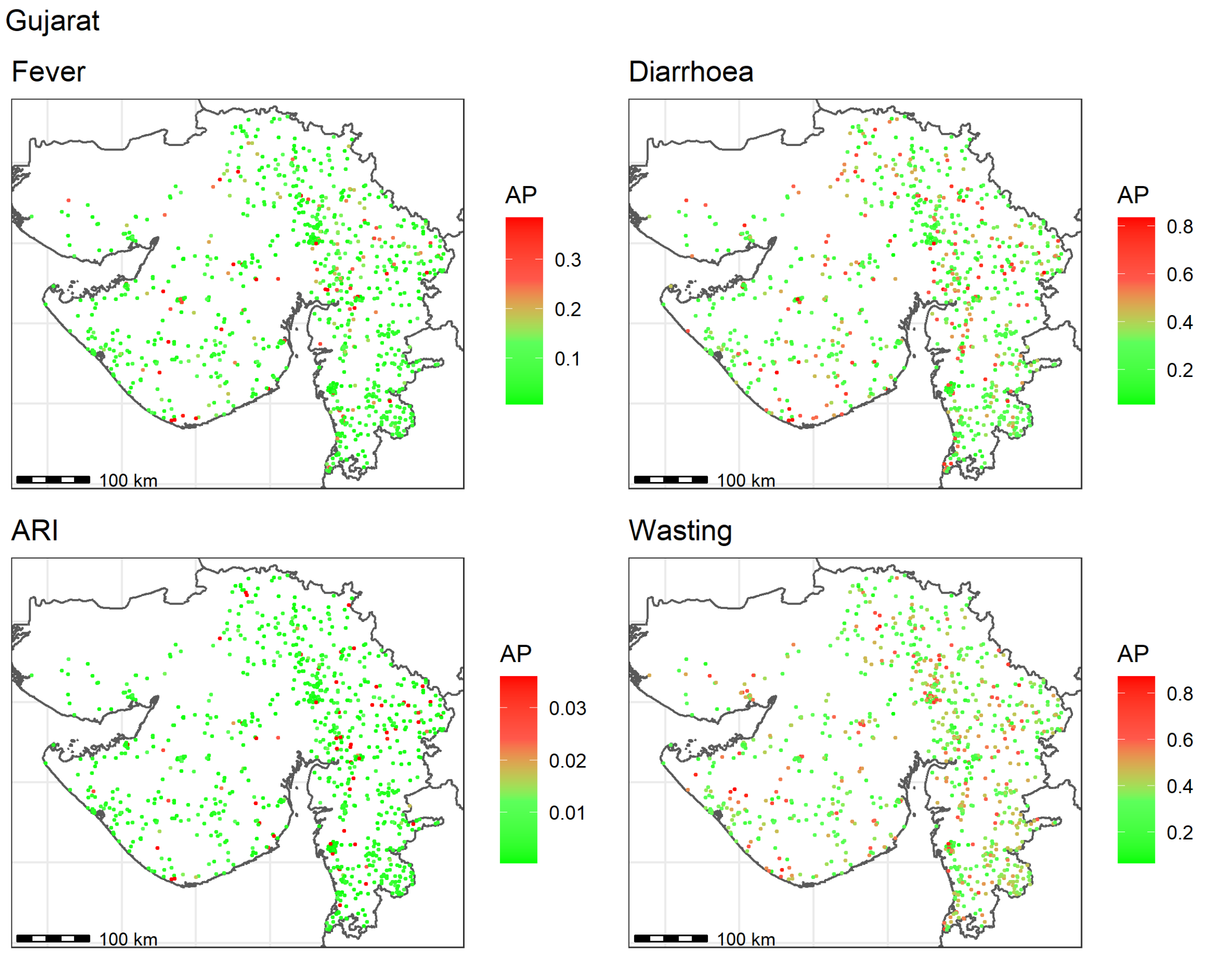
Gujarat
Fever
AP
0.3
0.2
0.1
100 km
Diarrhoea
AP
0.8
0.6
0.4
0.2
100 km
ARI
AP
0.03
0.02
0.01
100 km
Wasting
AP
0.8
0.6
0.4
0.2
100 km

Haiti

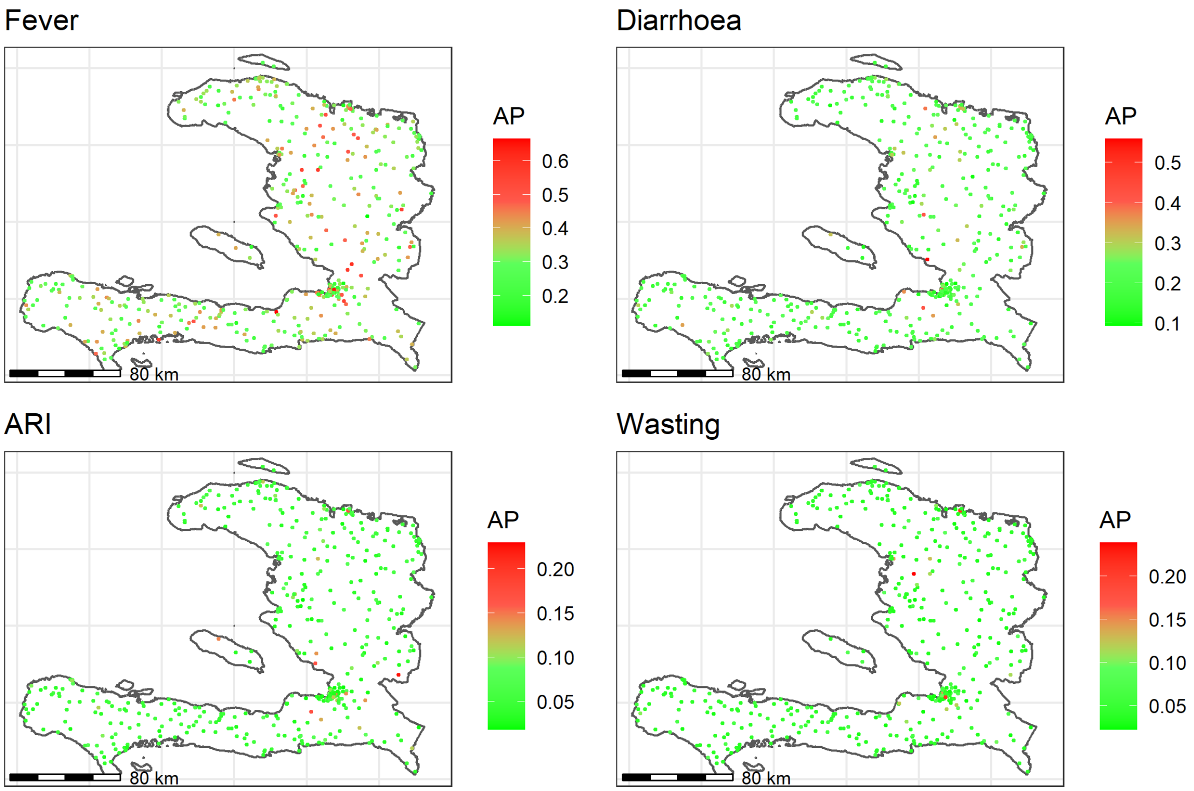
Fever
AP
0.6
0.5
0.4
0.3
0.2
80 km
Diarrhoea
AP
0.5
0.4
0.3
0.2
0.1
80 km
ARI
AP
0.20
0.15
0.10
0.05
80 km
Wasting
AP
0.20
0.15
0.10
0.05
80 km

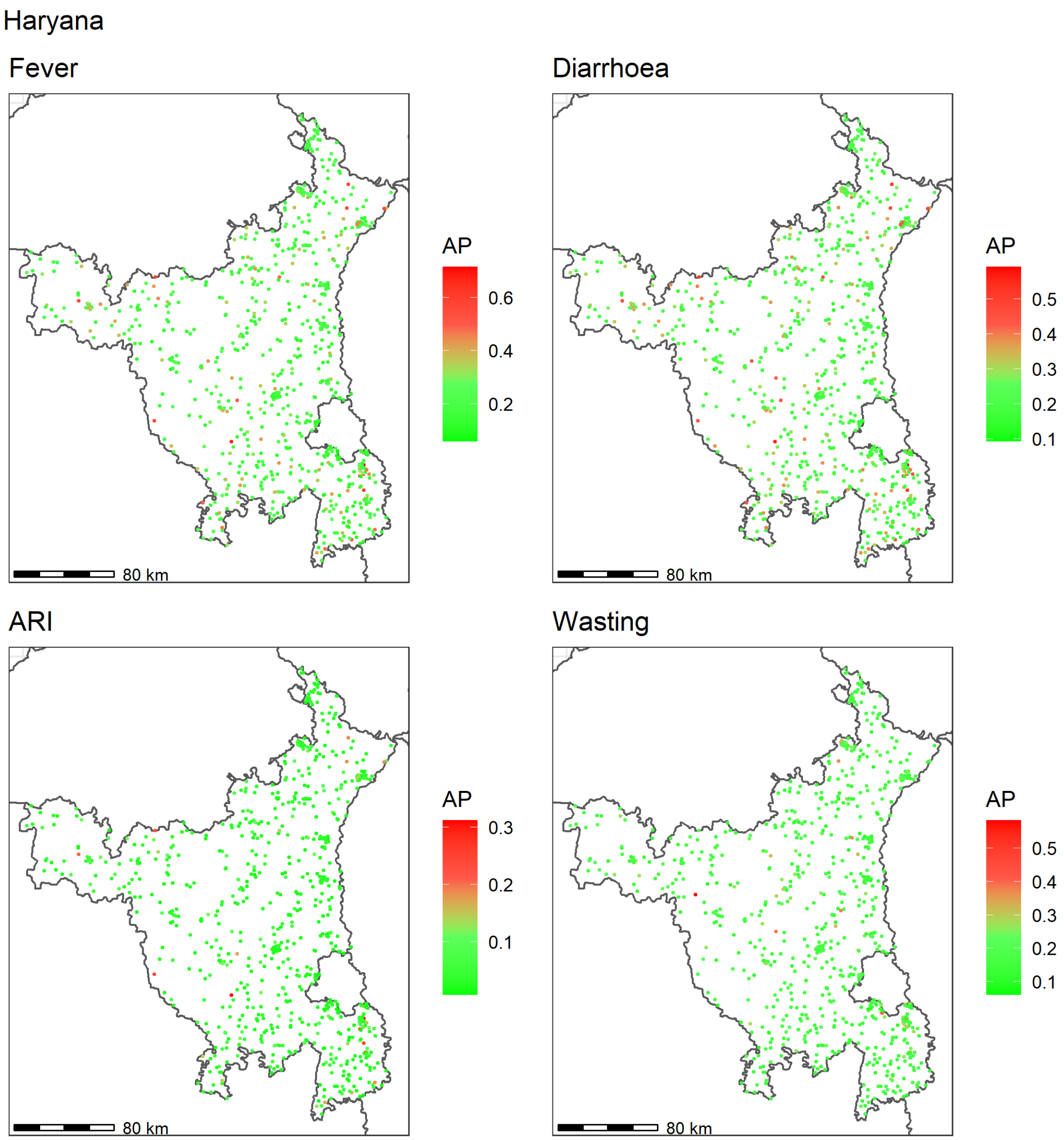
Haryana
Fever
AP
0.6
0.4
0.2
80 km
Diarrhoea
AP
0.5
0.4
0.3
0.2
0.1
80 km
ARI
AP
0.3
0.2
0.1
80 km
Wasting
AP
0.5
0.4
0.3
0.2
0.1
80 km

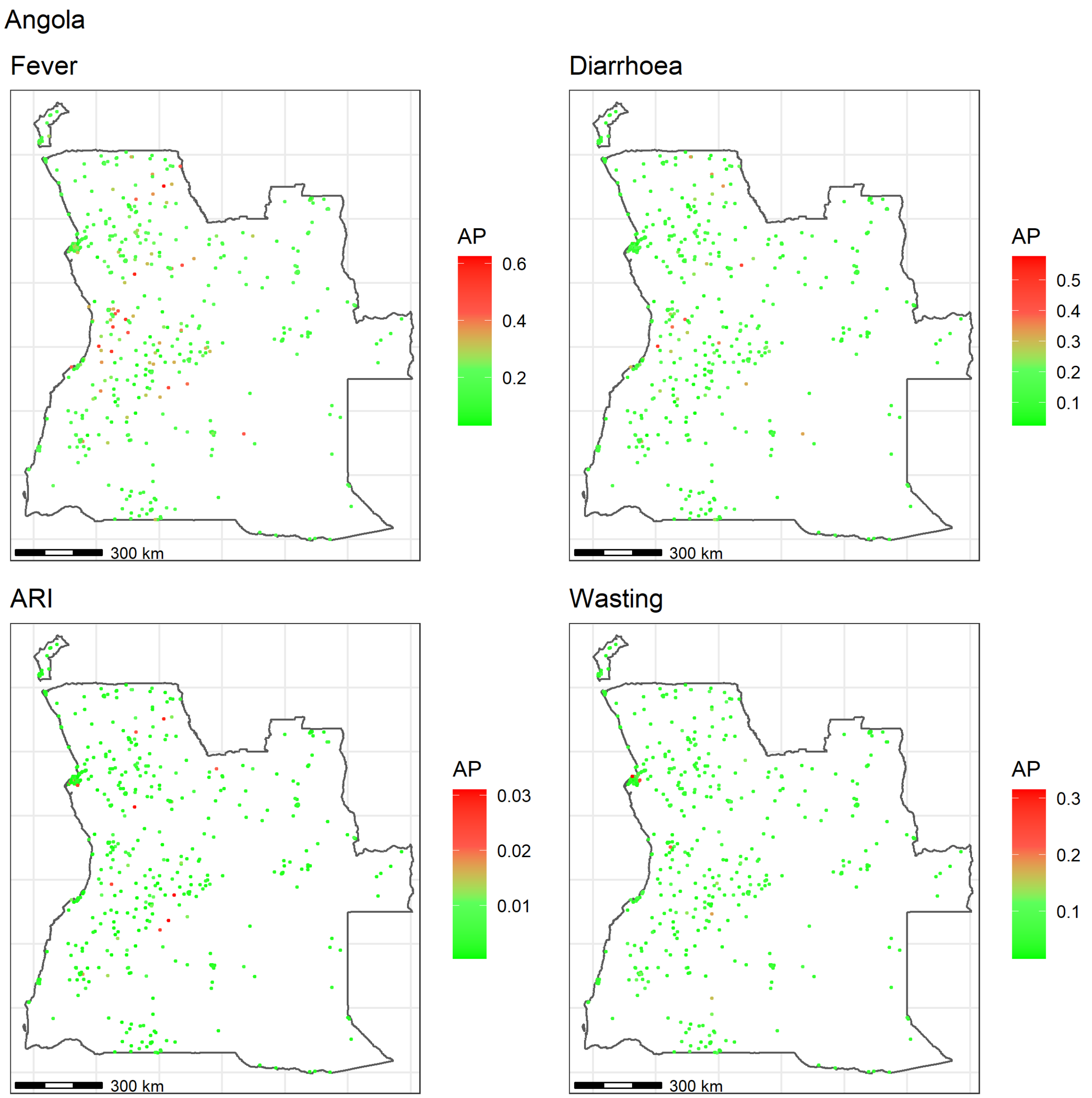
Angola
Fever
AP
0.6
0.4
0.2
300 km
Diarrhoea
AP
0.5
0.4
0.3
0.2
0.1
300 km
ARI
AP
0.03
0.02
0.01
300 km
Wasting
AP
0.3
0.2
0.1
300 km

Himachal Pradesh

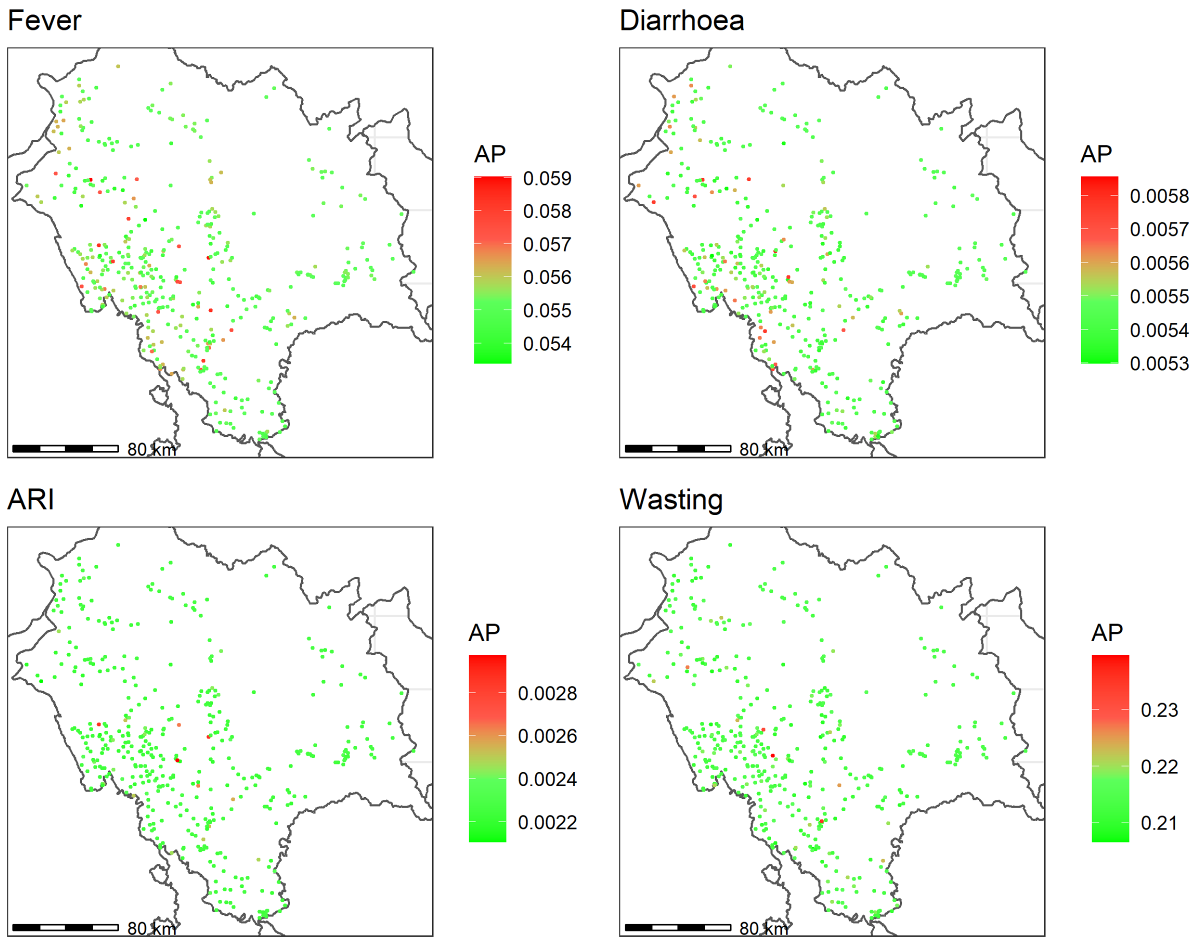

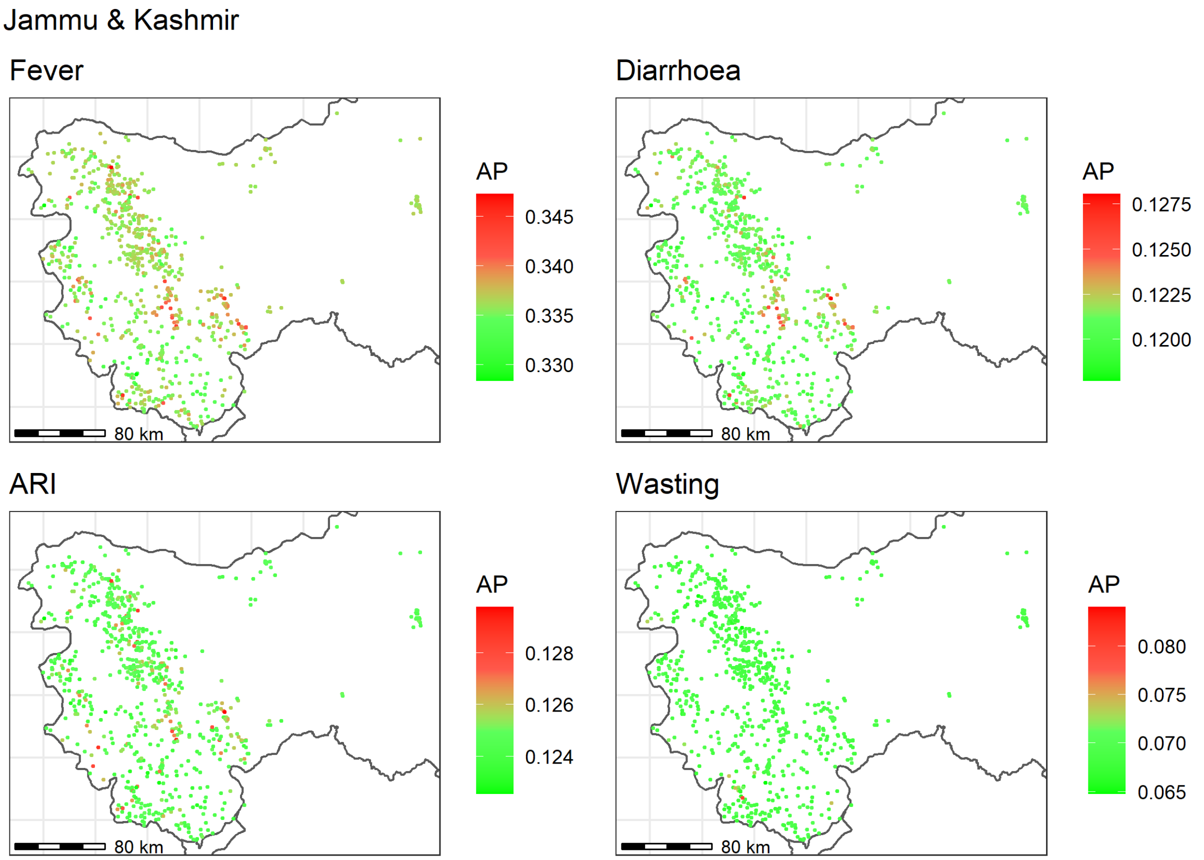
Jammu & Kashmir
Fever
AP
0.345
0.340
0.335
0.330
80 km
Diarrhoea
AP
0.1275
0.1250
0.1225
0.1200
80 km
ARI
AP
0.128
0.126
0.124
80 km
Wasting
AP
0.080
0.075
0.070
0.065
80 km

Jharkhand

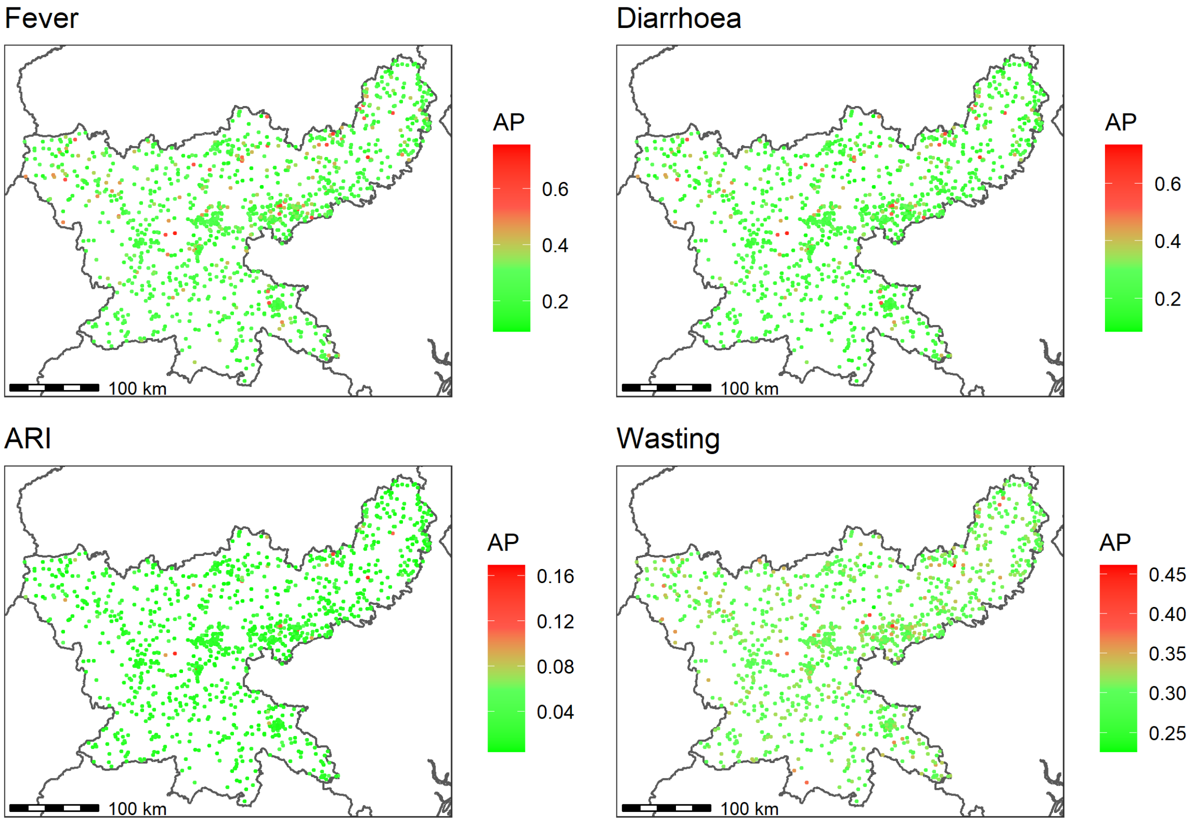
Fever
AP
0.6
0.4
0.2
100 km
Diarrhoea
AP
0.6
0.4
0.2
100 km
ARI
AP
0.16
0.12
0.08
0.04
100 km
Wasting
AP
0.45
0.40
0.35
0.30
0.25
100 km

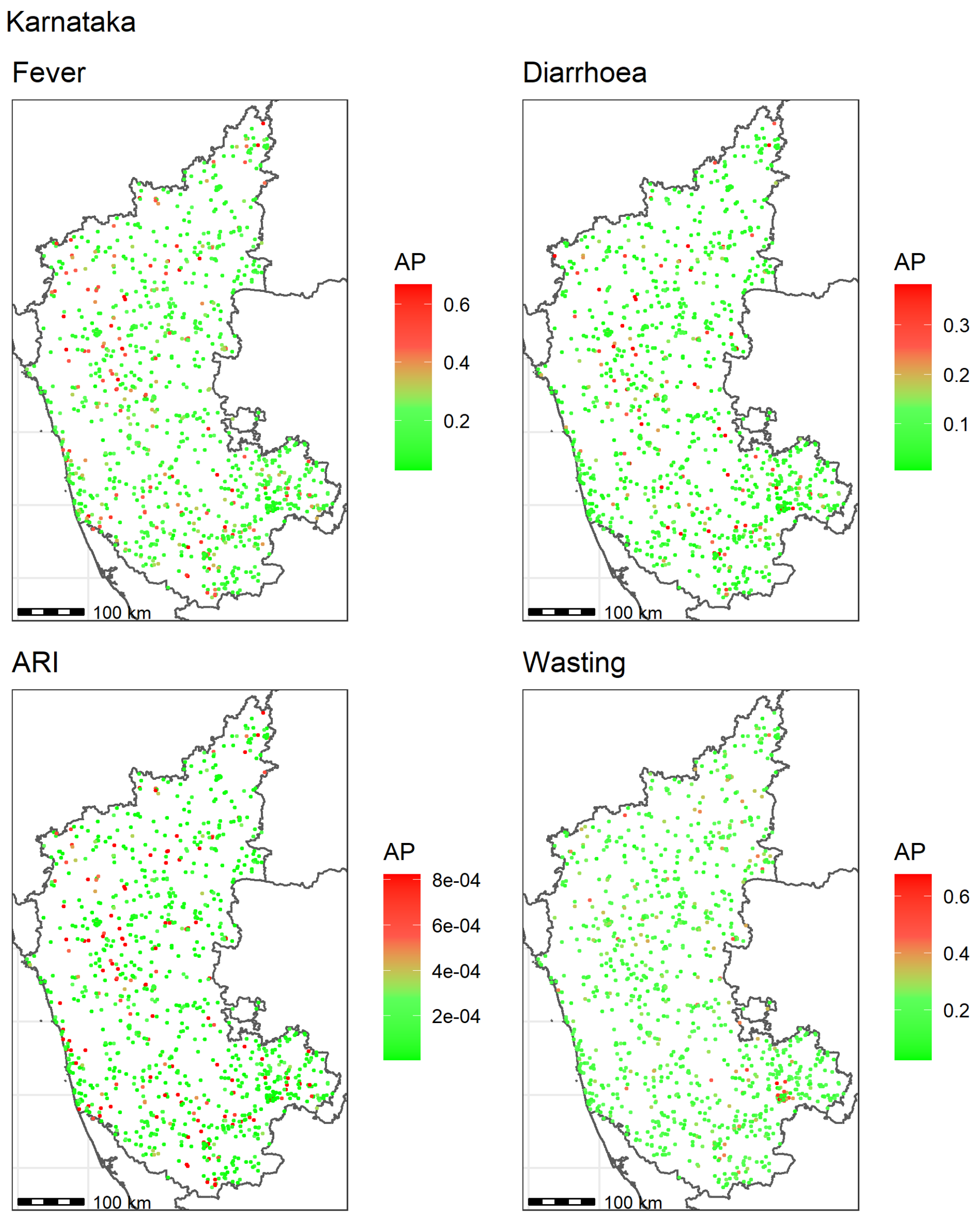
Karnataka
Fever
AP
0.6
0.4
0.2
100 km
Diarrhoea
AP
0.3
0.2
0.1
100 km
ARI
AP
8e-04
6e-04
4e-04
2e-04
100 km
Wasting
AP
0.6
0.4
0.2
100 km

Kenya

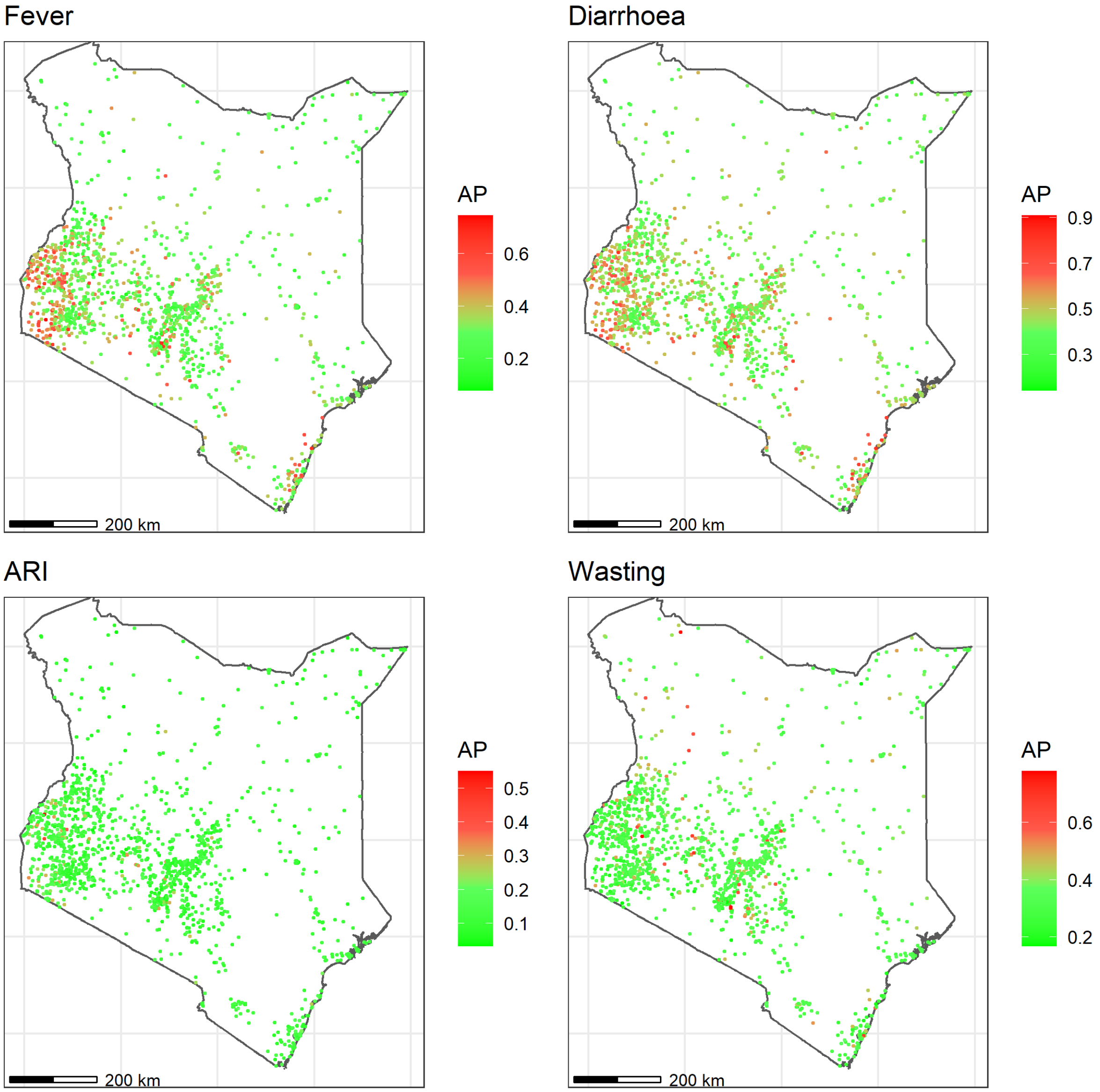

Fever
AP
0.6
0.4
0.2
200 km
Diarrhoea
AP
0.9
0.7
0.5
0.3
200 km
ARI
AP
0.5
0.4
0.3
0.2
0.1
200 km
Wasting
AP
0.6
0.4
0.2
200 km

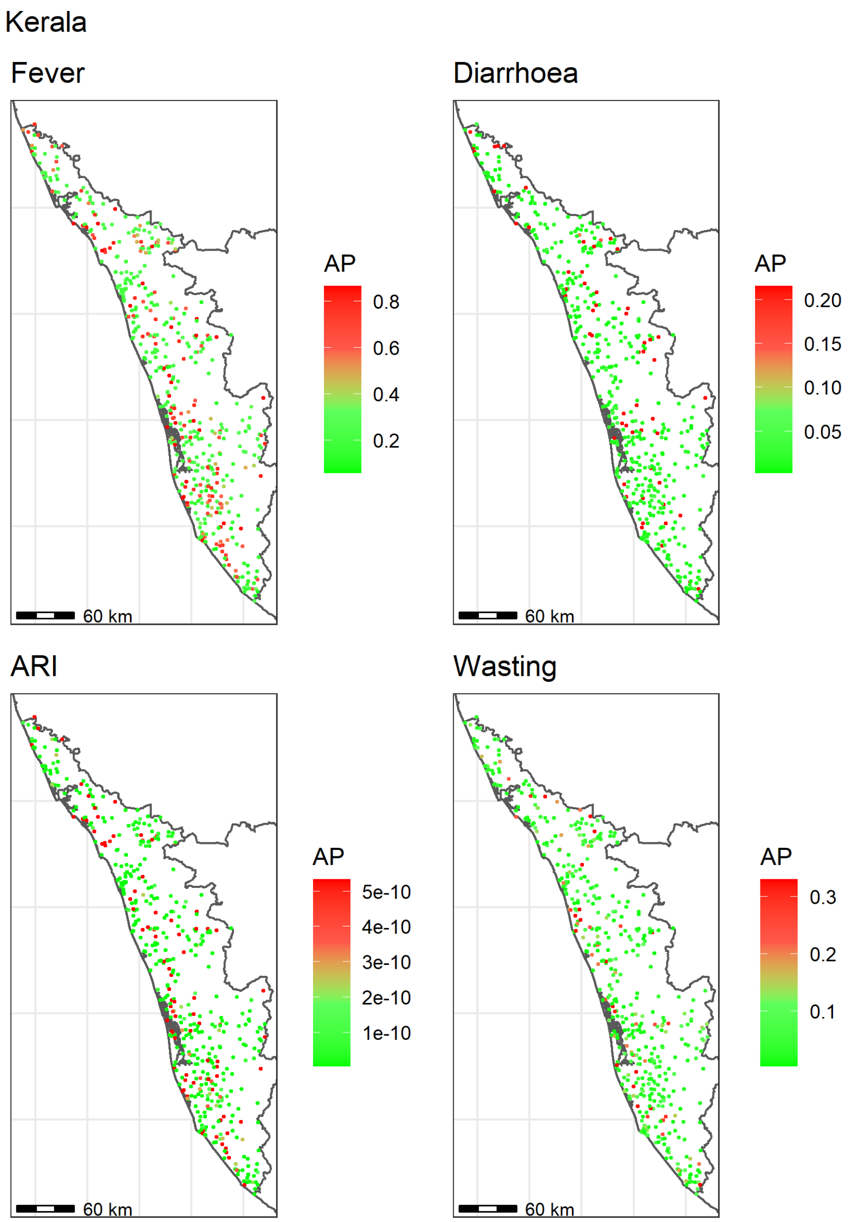

Kerala
Fever
AP
0.8
0.6
0.4
0.2
60 km
Diarrhoea
AP
0.20
0.15
0.10
0.05
60 km
ARI
AP
5e-10
4e-10
3e-10
2e-10
1e-10
60 km
Wasting
AP
0.3
0.2
0.1
60 km

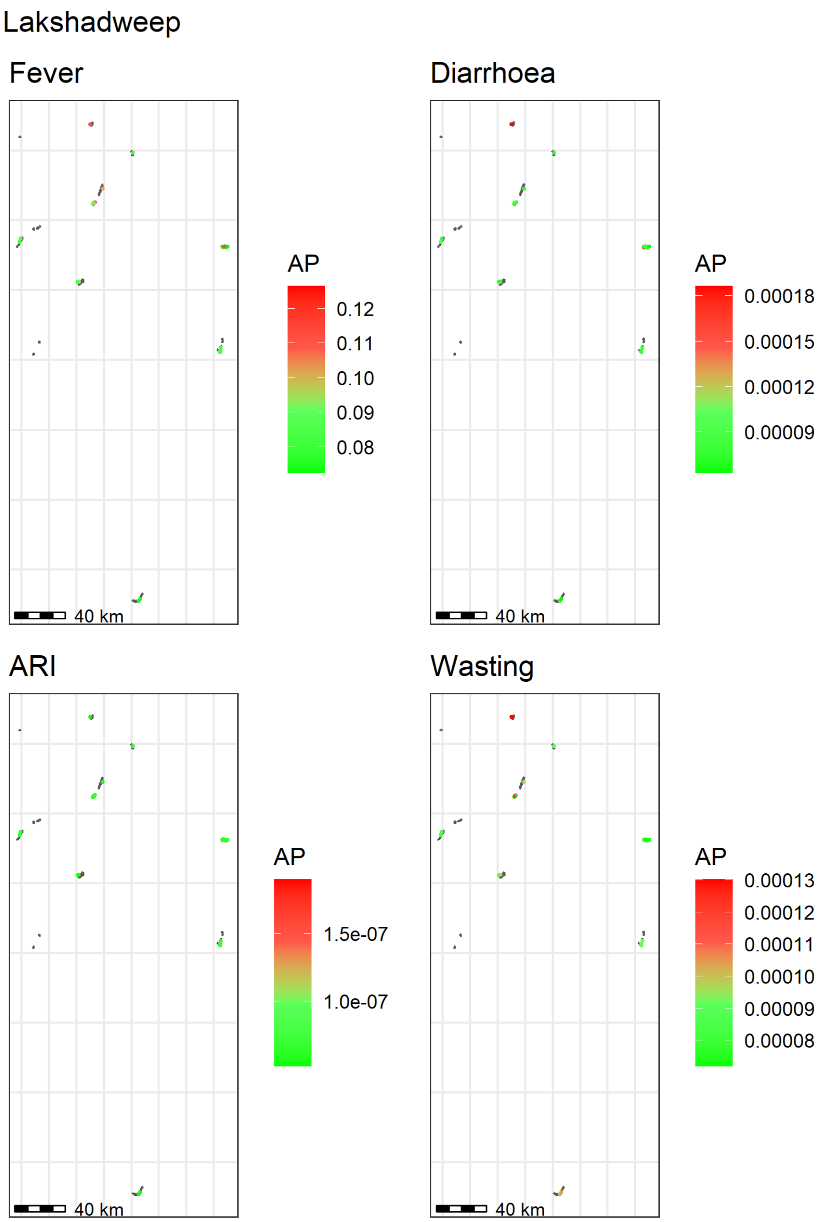

Lakshadweep
Fever
40 km
AP
0.12
0.11
0.10
0.09
0.08
Diarrhoea
40 km
AP
0.00018
0.00015
0.00012
0.00009
ARI
40 km
AP
1.5e-07
1.0e-07
Wasting
40 km
AP
0.00013
0.00012
0.00011
0.00010
0.00009
0.00008

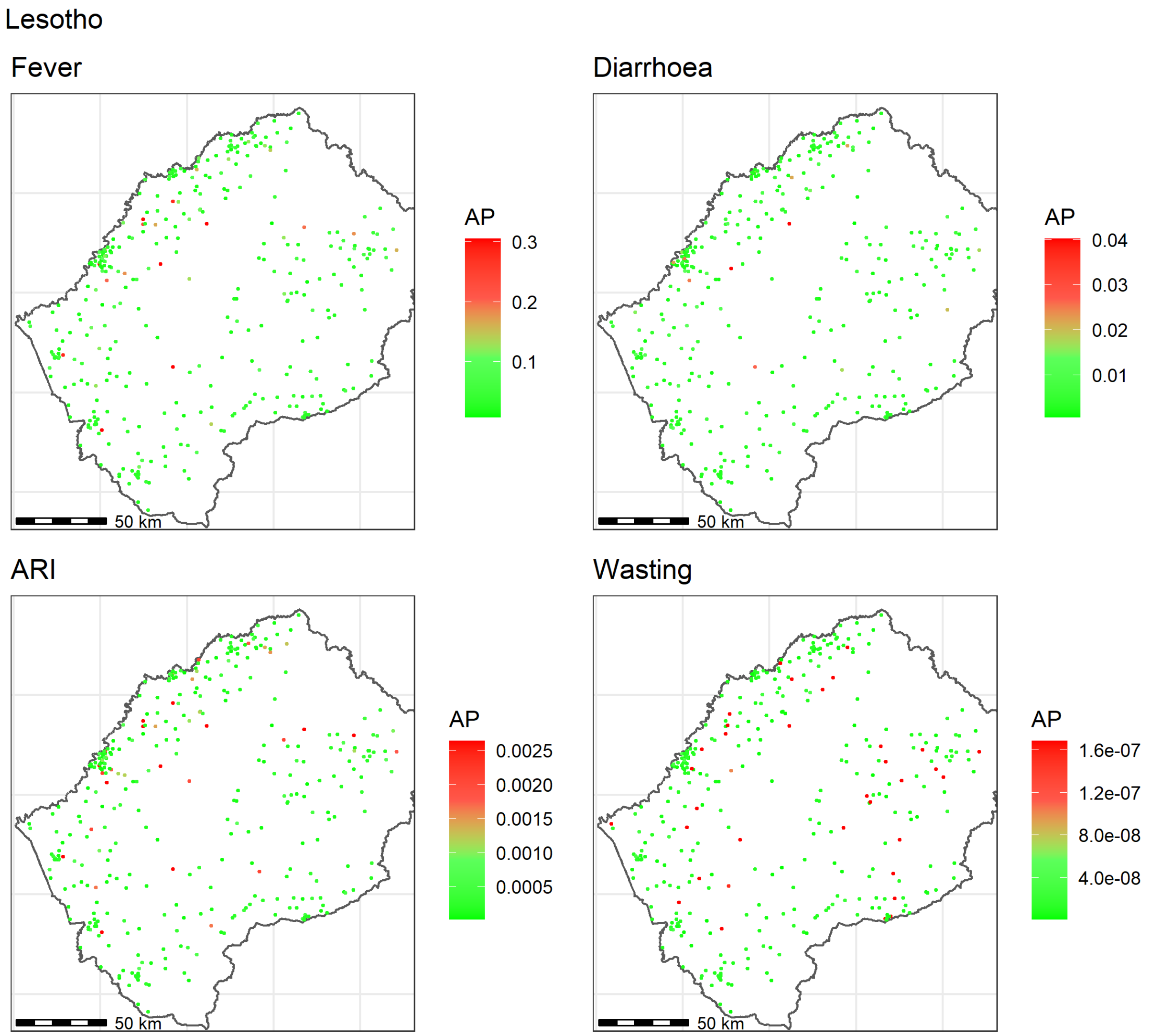
Lesotho
Fever
AP
0.3
0.2
0.1
50 km
Diarrhoea
AP
0.04
0.03
0.02
0.01
50 km
ARI
AP
0.0025
0.0020
0.0015
0.0010
0.0005
50 km
Wasting
AP
1.6e-07
1.2e-07
8.0e-08
4.0e-08
50 km

Liberia

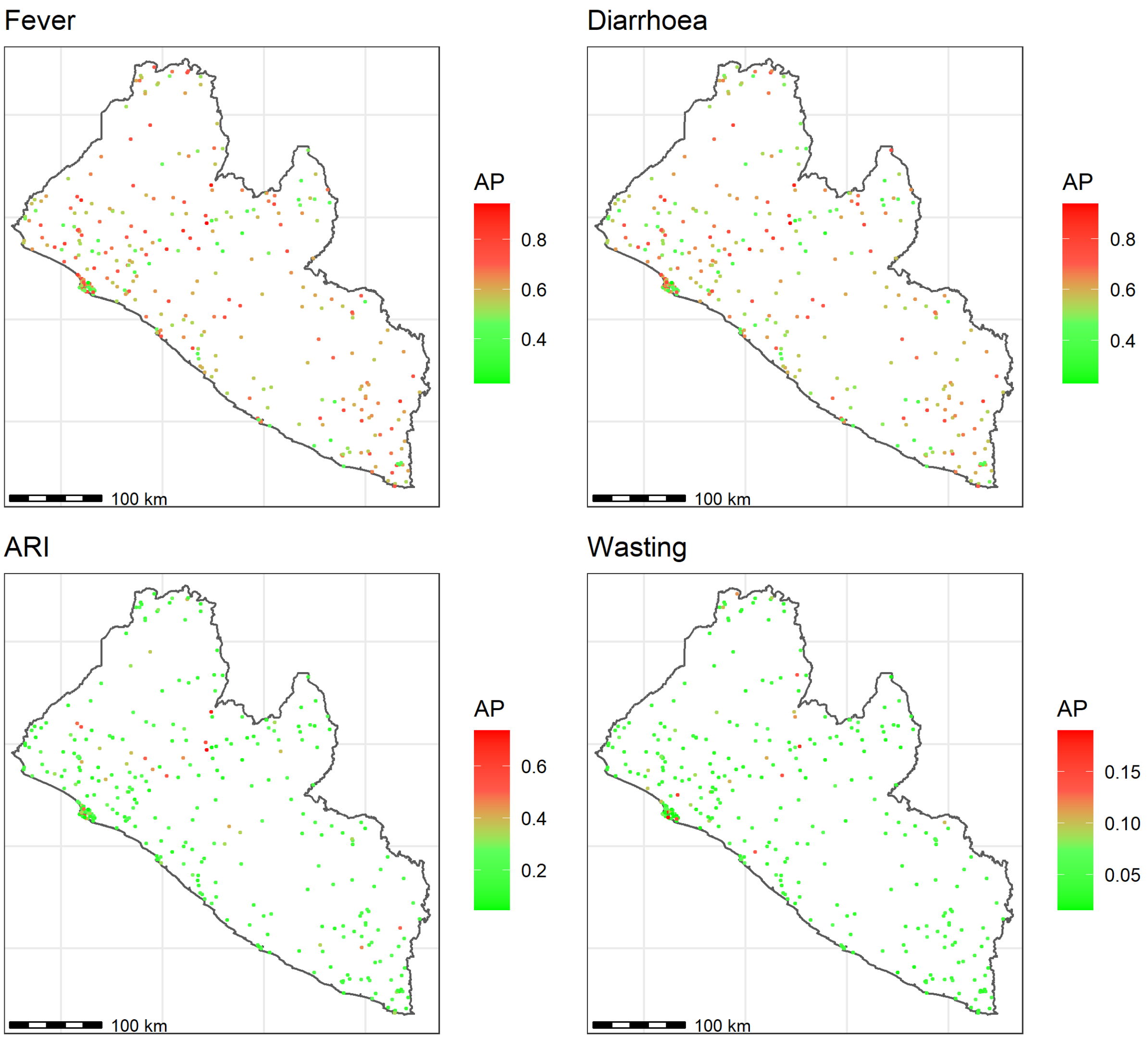

Fever
AP
0.8
0.6
0.4
100 km
Diarrhoea
AP
0.8
0.6
0.4
100 km
ARI
AP
0.6
0.4
0.2
100 km
Wasting
AP
0.15
0.10
0.05
100 km

Madhya Pradesh

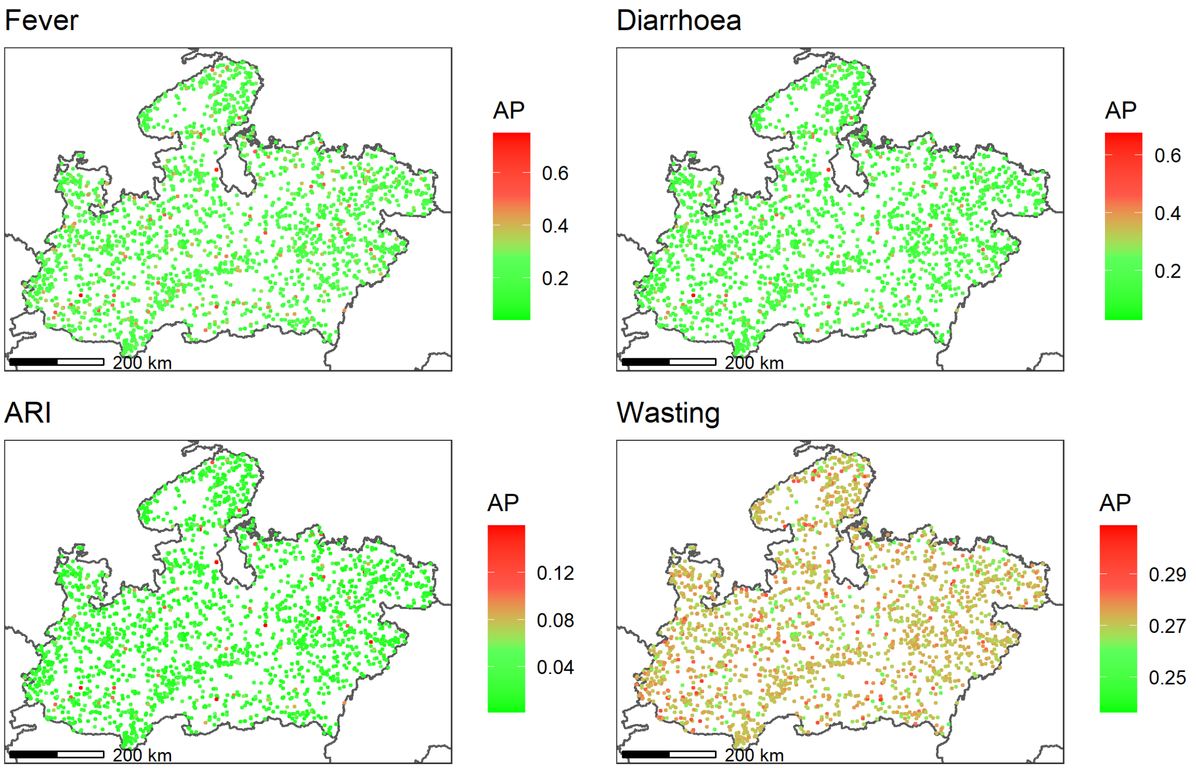

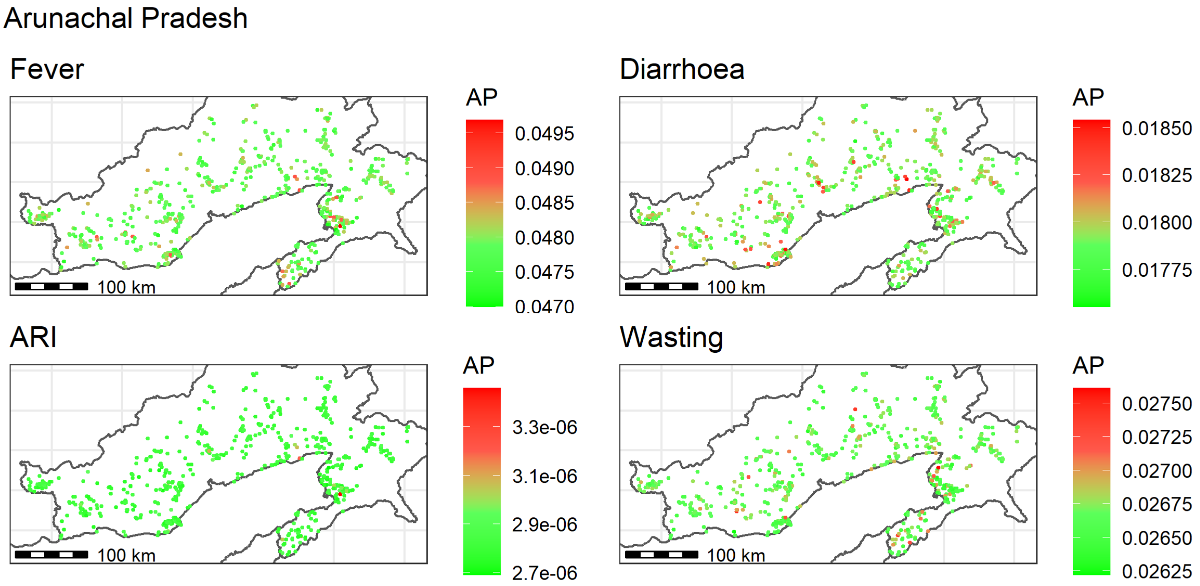
Arunachal Pradesh
Fever
100 km
AP
0.0495
0.0490
0.0485
0.0480
0.0475
0.0470
Diarrhoea
100 km
AP
0.01850
0.01825
0.01800
0.01775
ARI
100 km
AP
3.3e-06
3.1e-06
2.9e-06
2.7e-06
Wasting
100 km
AP
0.02750
0.02725
0.02700
0.02675
0.02650
0.02625

Maharashtra

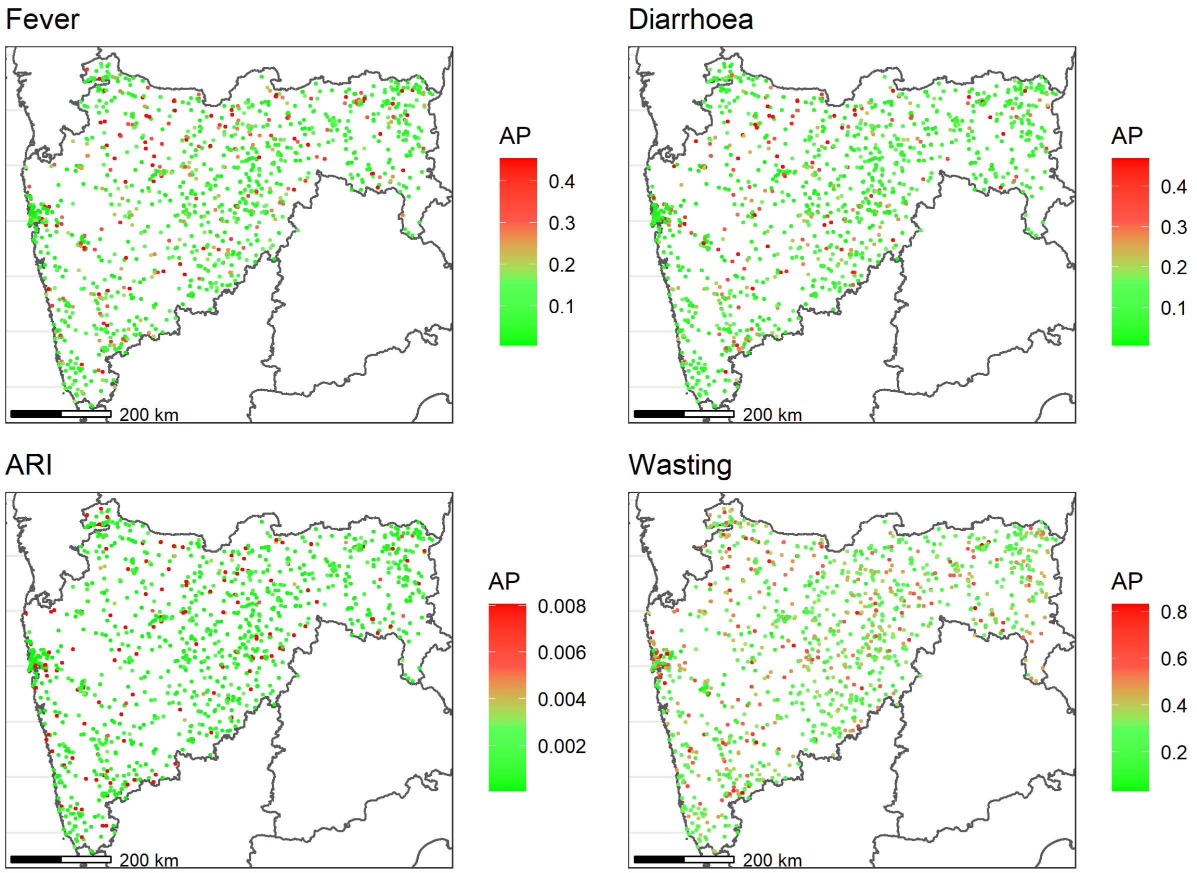

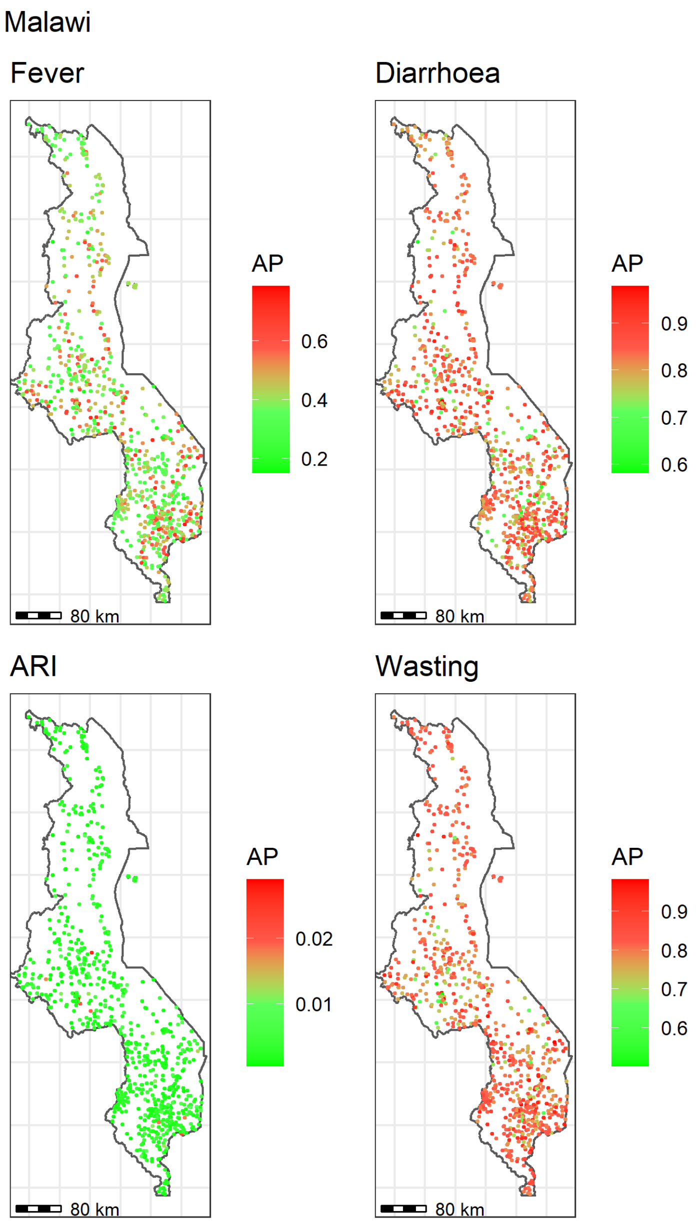
Malawi
Fever
AP
0.6
0.4
0.2
80 km
Diarrhoea
AP
0.9
0.8
0.7
0.6
80 km
ARI
AP
0.02
0.01
80 km
Wasting
AP
0.9
0.8
0.7
0.6
80 km

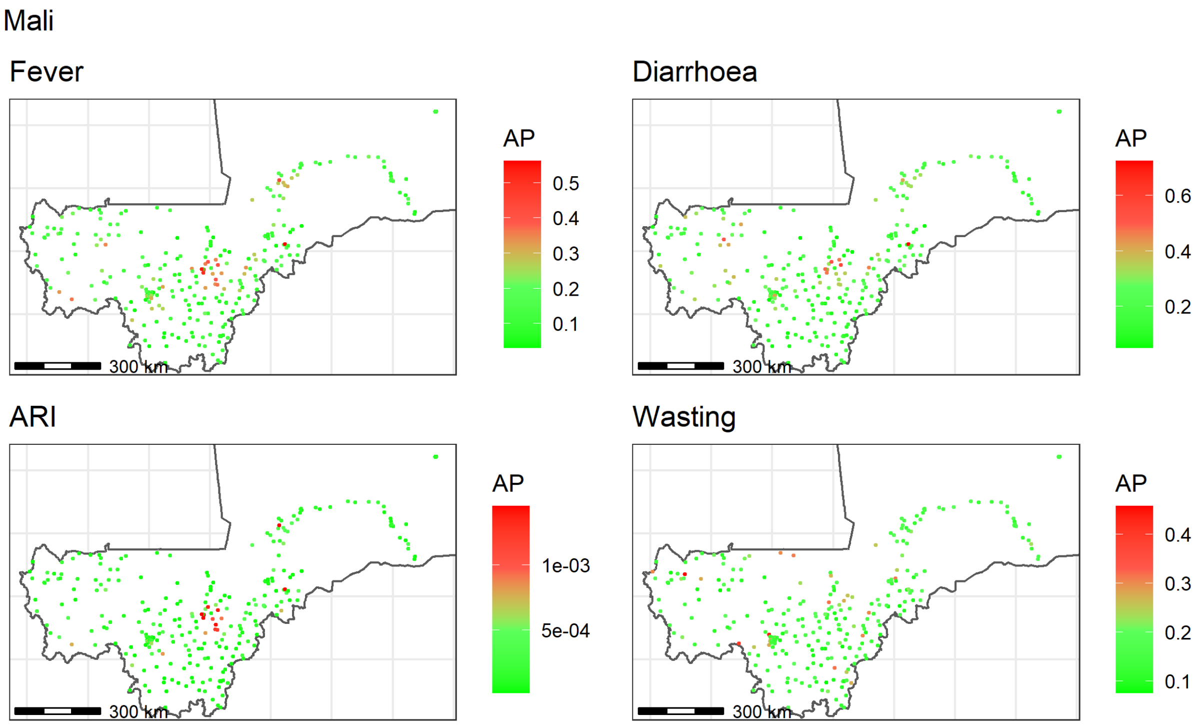
Mali
Fever
AP
0.5
0.4
0.3
0.2
0.1
300 km
Diarrhoea
AP
0.6
0.4
0.2
300 km
ARI
AP
1e-03
5e-04
300 km
Wasting
AP
0.4
0.3
0.2
0.1
300 km

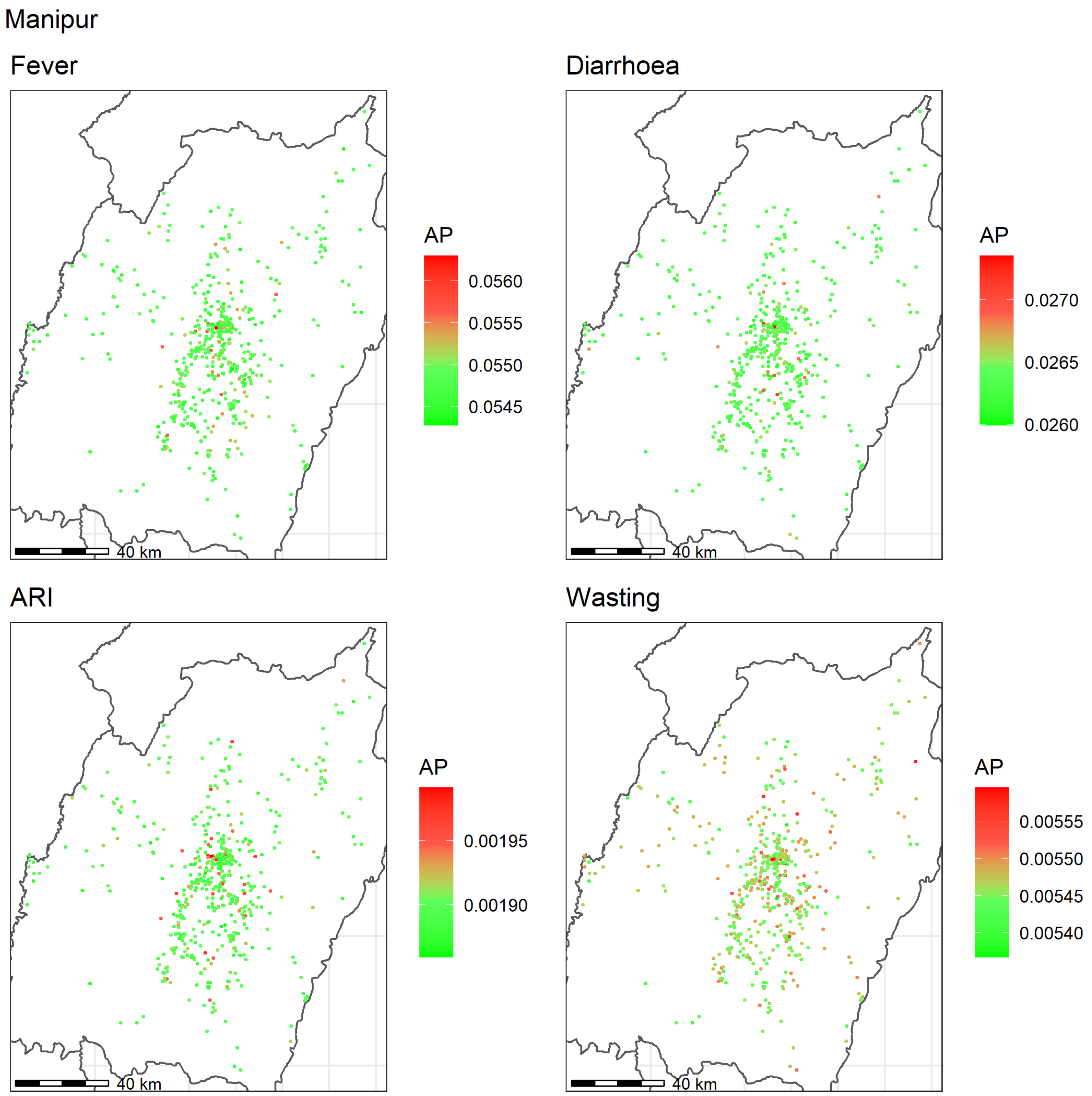
Manipur
Fever
AP
0.0560
0.0555
0.0550
0.0545
40 km
Diarrhoea
AP
0.0270
0.0265
0.0260
40 km
ARI
AP
0.00195
0.00190
40 km
Wasting
AP
0.00555
0.00550
0.00545
0.00540
40 km

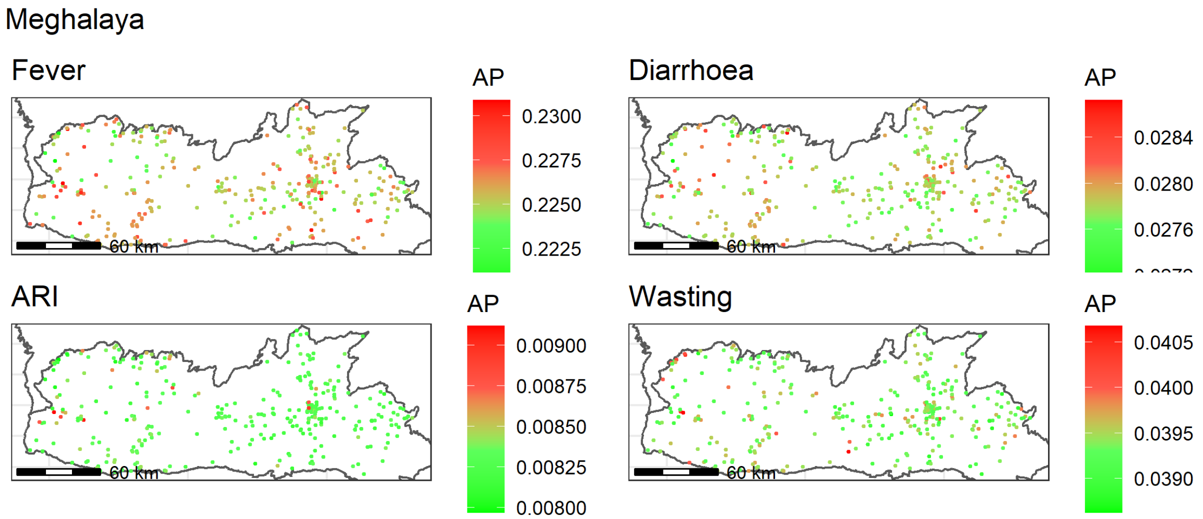
Meghalaya
Fever
AP
0.2300
0.2275
0.2250
0.2225
60 km
Diarrhoea
AP
0.0284
0.0280
0.0276
60 km
ARI
AP
0.00900
0.00875
0.00850
0.00825
0.00800
60 km
Wasting
AP
0.0405
0.0400
0.0395
0.0390
60 km

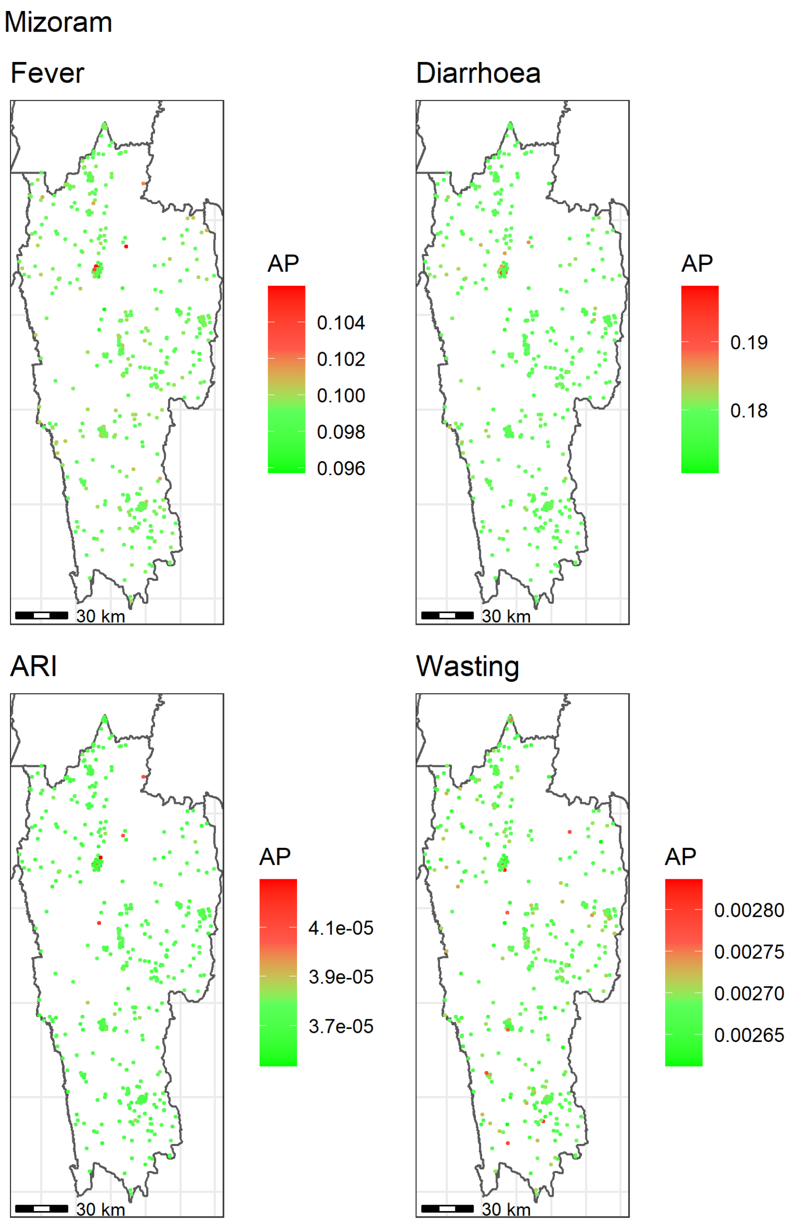
Mizoram
Fever
AP
0.104
0.102
0.100
0.098
0.096
30 km
Diarrhoea
AP
0.19
0.18
30 km
ARI
AP
4.1e-05
3.9e-05
3.7e-05
30 km
Wasting
AP
0.00280
0.00275
0.00270
0.00265
30 km

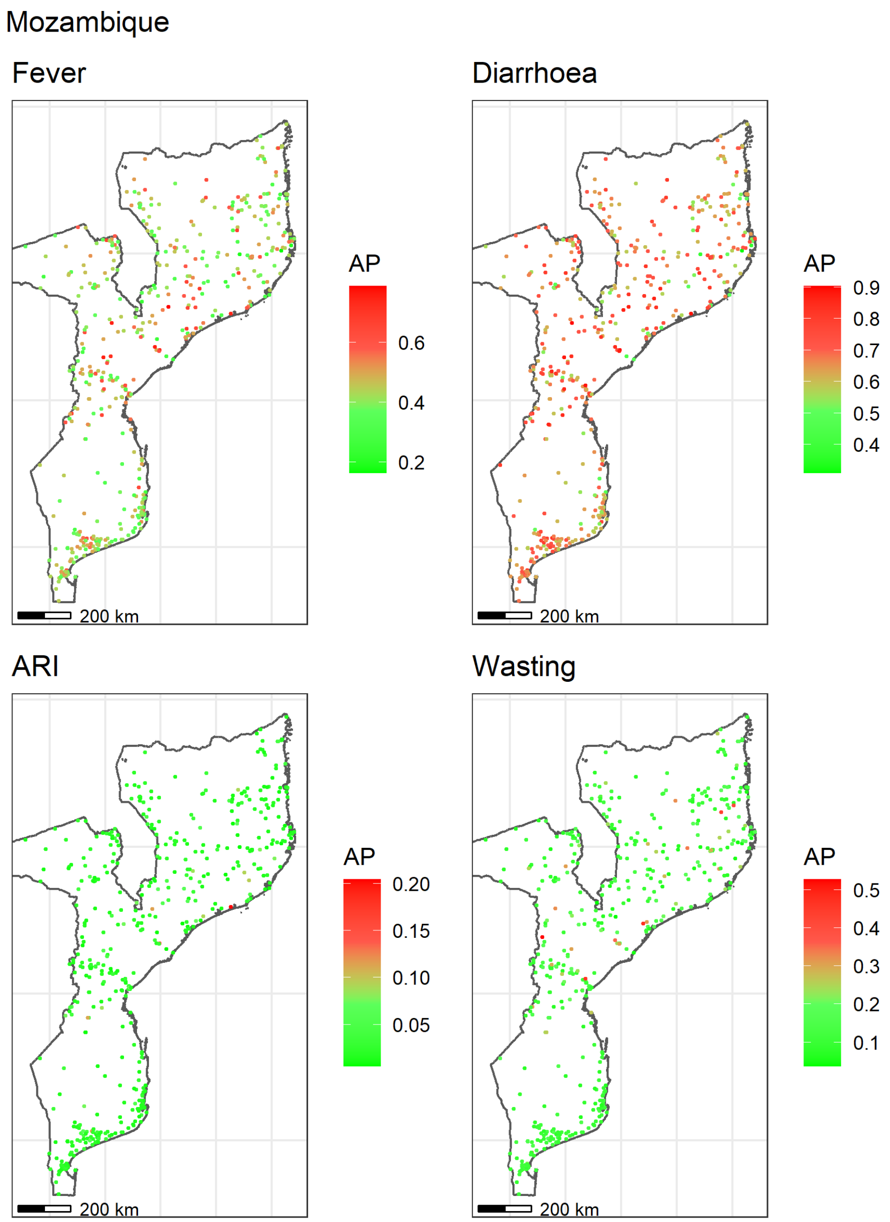
Mozambique
Fever
AP
0.6
0.4
0.2
200 km
Diarrhoea
AP
0.9
0.8
0.7
0.6
0.5
0.4
200 km
ARI
AP
0.20
0.15
0.10
0.05
200 km
Wasting
AP
0.5
0.4
0.3
0.2
0.1
200 km

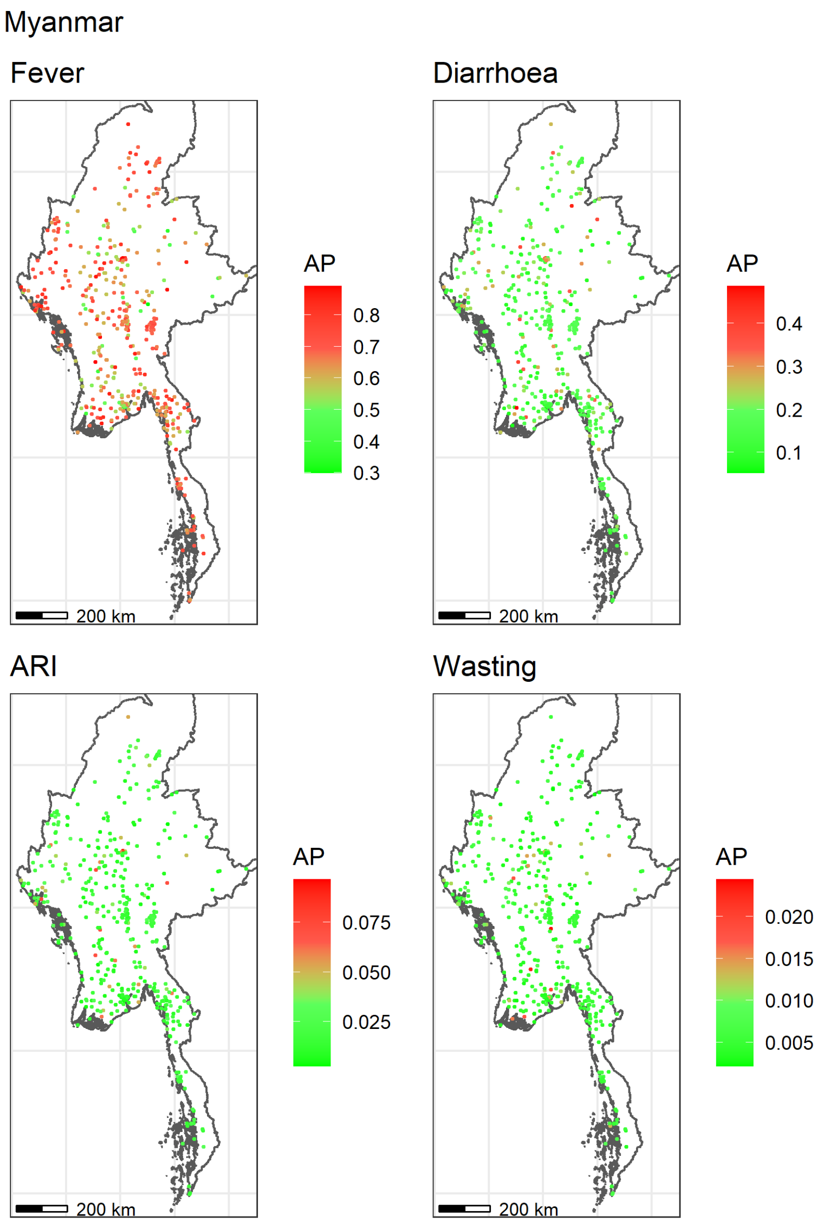
Myanmar
Fever
Diarrhoea
ARI
Wasting
AP
0.8
0.7
0.6
0.5
0.4
0.3
AP
0.4
0.3
0.2
0.1
AP
0.075
0.050
0.025
AP
0.020
0.015
0.010
0.005
200 km

Nagaland

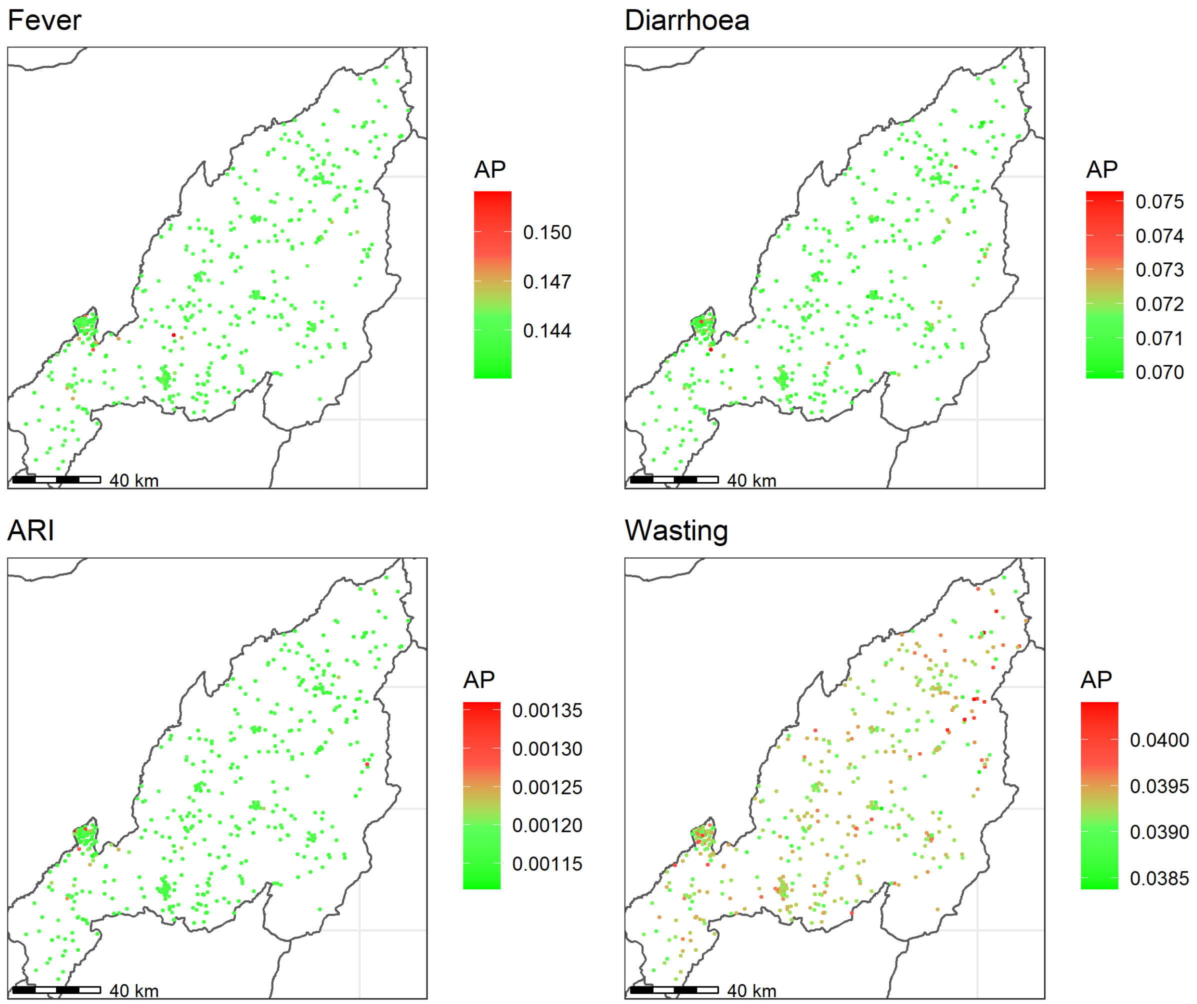

Fever
AP
0.150
0.147
0.144
40 km
Diarrhoea
AP
0.075
0.074
0.073
0.072
0.071
0.070
40 km
ARI
AP
0.00135
0.00130
0.00125
0.00120
0.00115
40 km
Wasting
AP
0.0400
0.0395
0.0390
0.0385
40 km

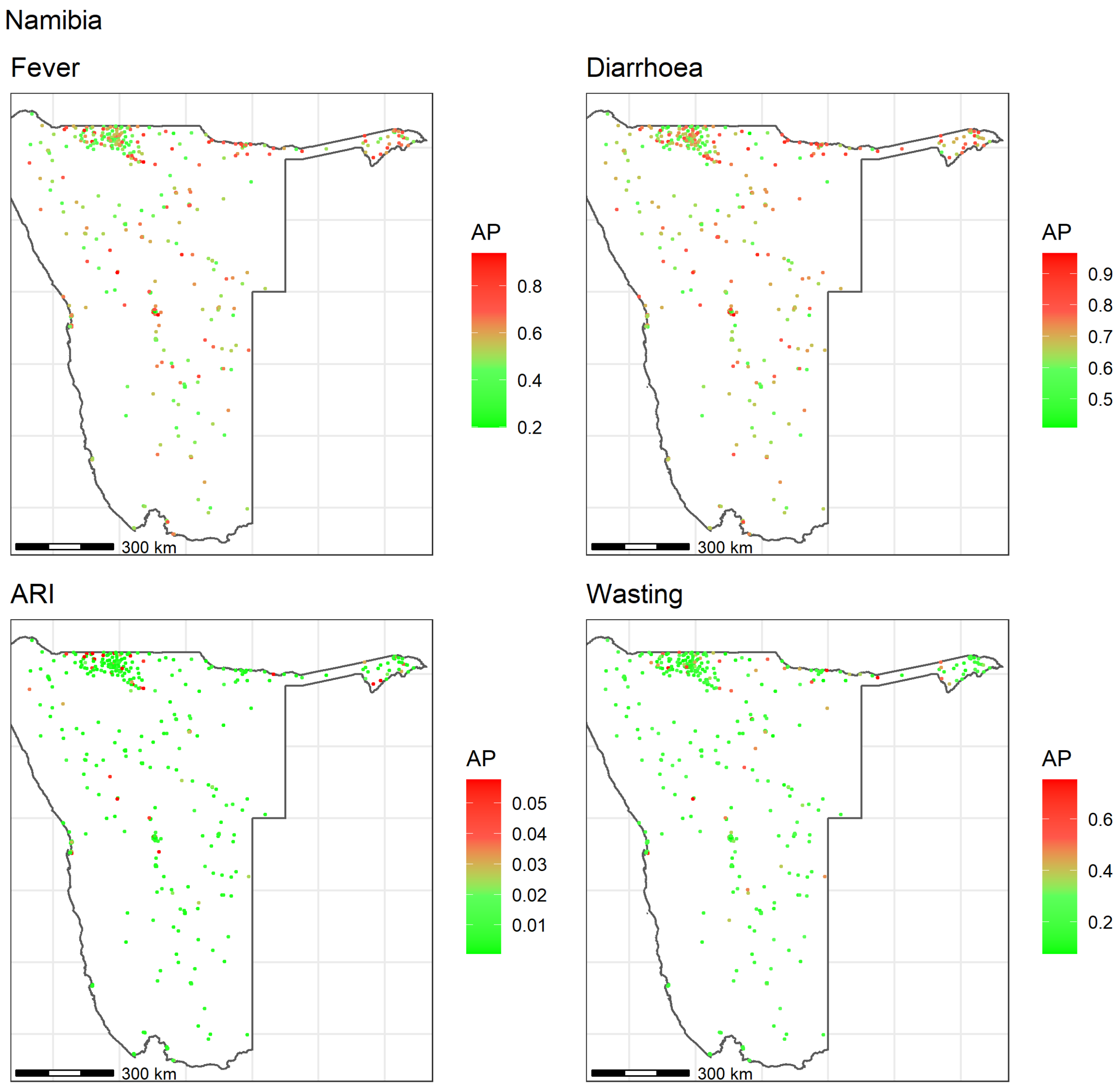
Namibia
Fever
AP
0.8
0.6
0.4
0.2
300 km
Diarrhoea
AP
0.9
0.8
0.7
0.6
0.5
300 km
ARI
AP
0.05
0.04
0.03
0.02
0.01
300 km
Wasting
AP
0.6
0.4
0.2
300 km

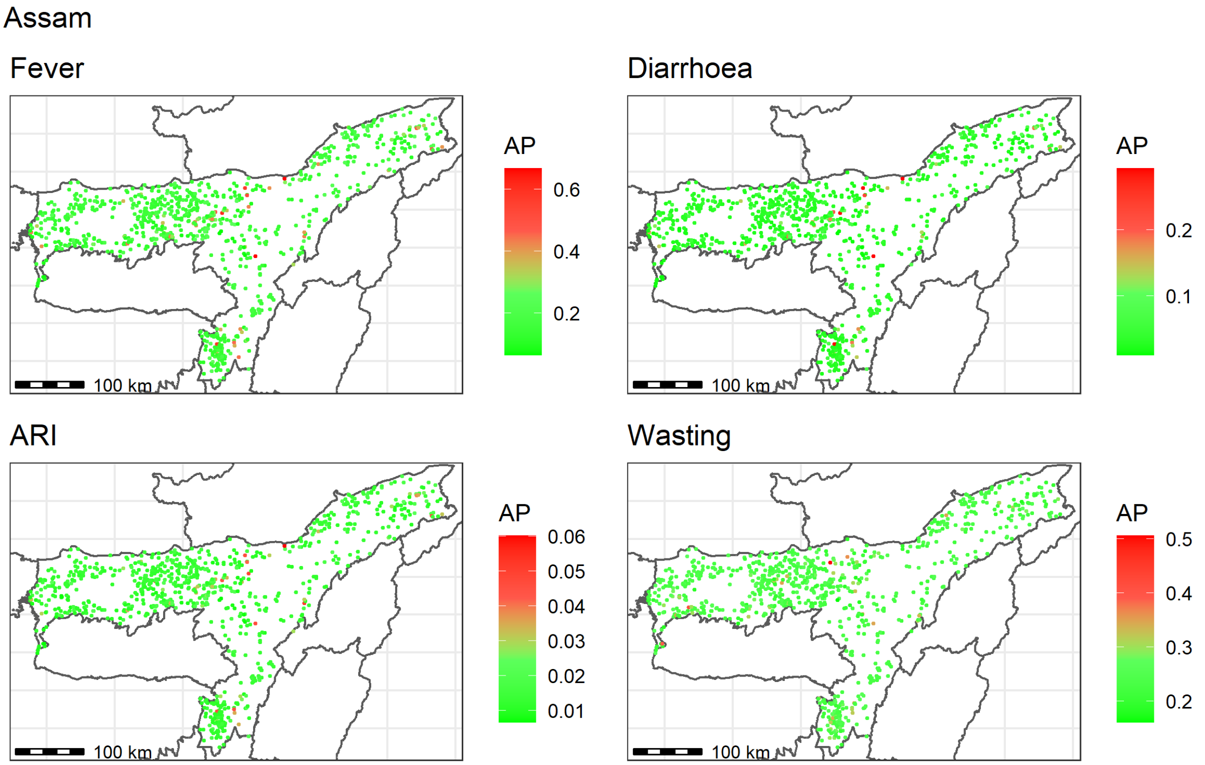
Assam
Fever
AP
0.6
0.4
0.2
100 km
Diarrhoea
AP
0.2
0.1
100 km
ARI
AP
0.06
0.05
0.04
0.03
0.02
0.01
100 km
Wasting
AP
0.5
0.4
0.3
0.2
100 km

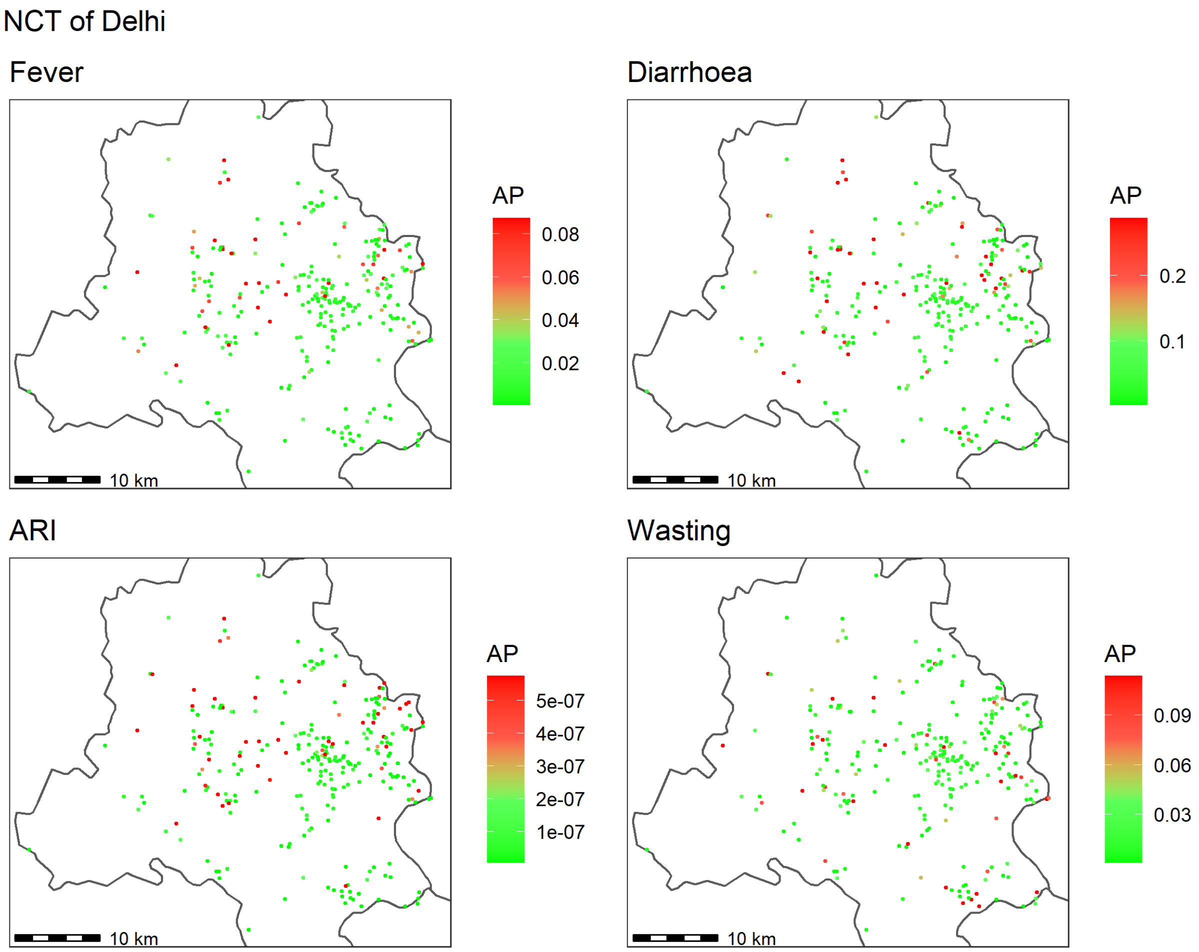
NCT of Delhi
Fever
AP
0.08
0.06
0.04
0.02
10 km
Diarrhoea
AP
0.2
0.1
10 km
ARI
AP
5e-07
4e-07
3e-07
2e-07
1e-07
10 km
Wasting
AP
0.09
0.06
0.03
10 km

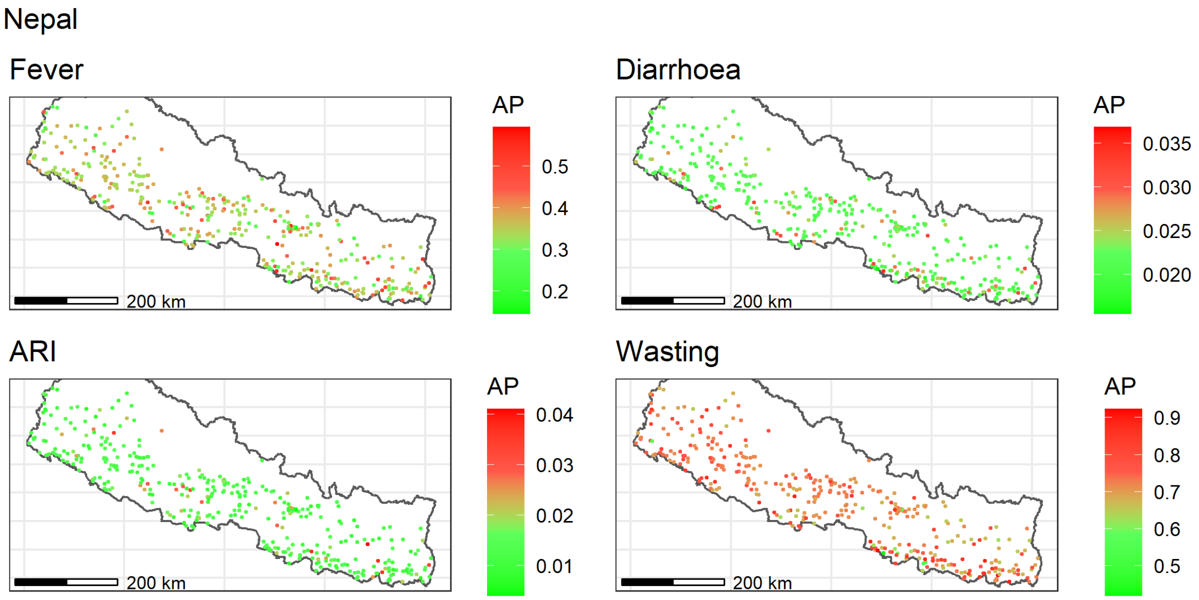

Nepal
Fever
AP
0.5
0.4
0.3
0.2
200 km
Diarrhoea
AP
0.035
0.030
0.025
0.020
200 km
ARI
AP
0.04
0.03
0.02
0.01
200 km
Wasting
AP
0.9
0.8
0.7
0.6
0.5
200 km

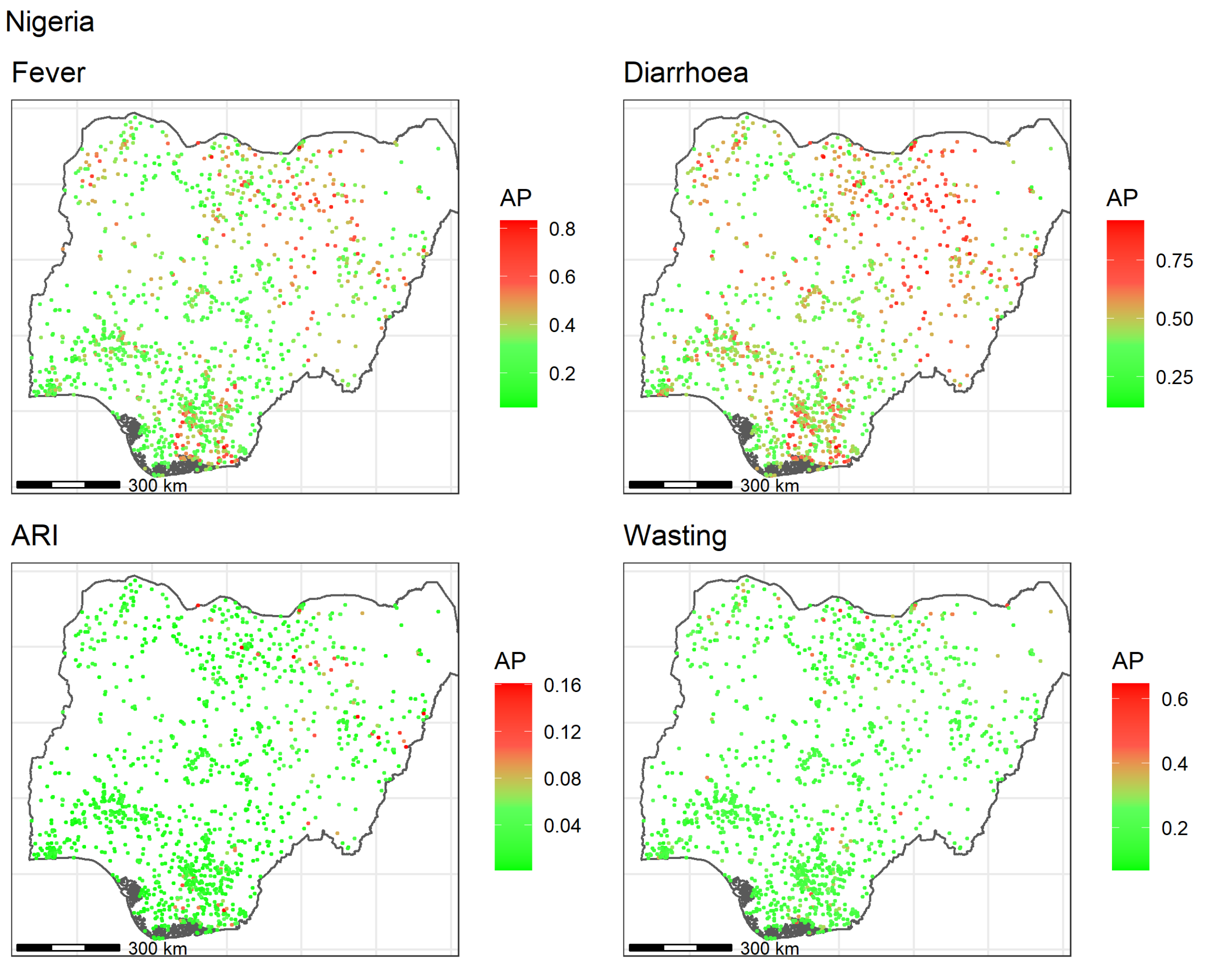
Nigeria
Fever
AP
0.8
0.6
0.4
0.2
300 km
Diarrhoea
AP
0.75
0.50
0.25
300 km
ARI
AP
0.16
0.12
0.08
0.04
300 km
Wasting
AP
0.6
0.4
0.2
300 km

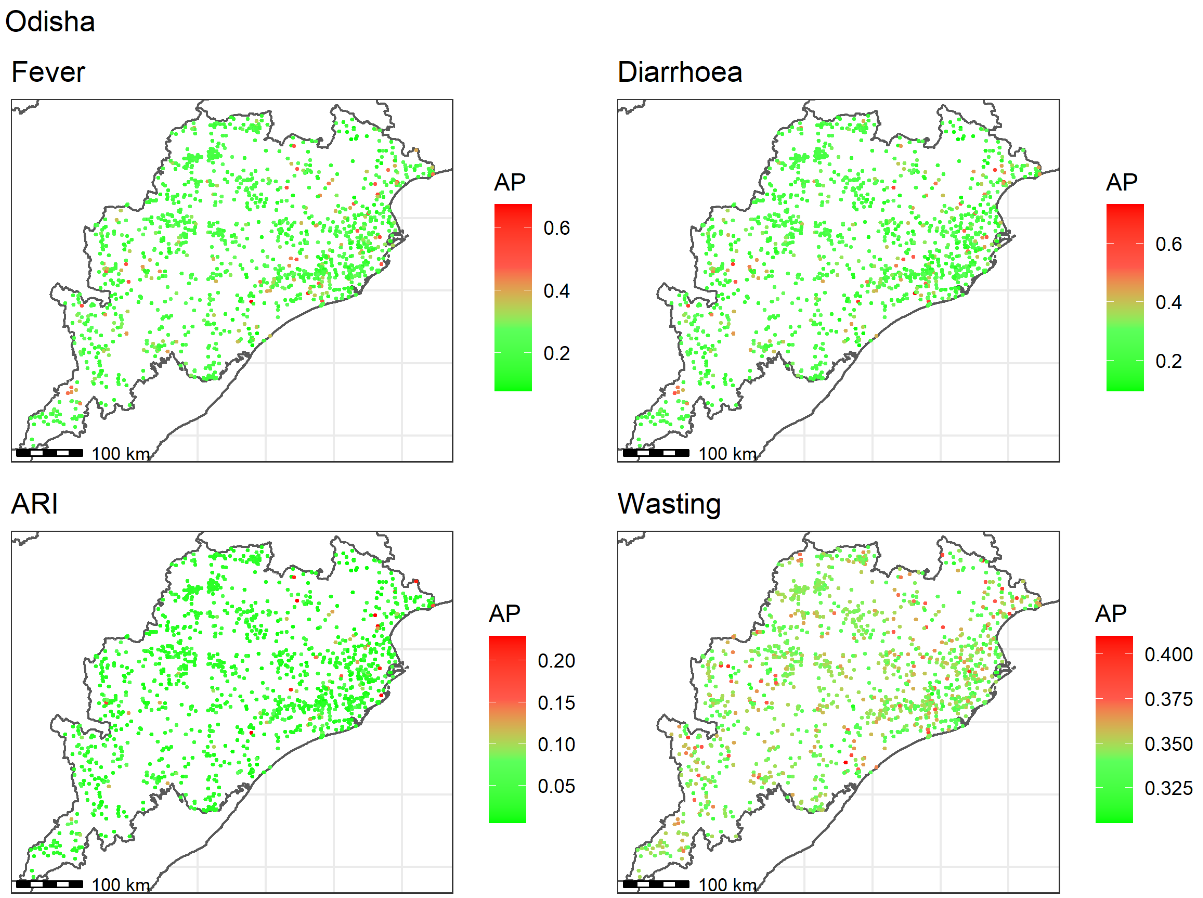
Odisha
Fever
AP
0.6
0.4
0.2
100 km
Diarrhoea
AP
0.6
0.4
0.2
100 km
ARI
AP
0.20
0.15
0.10
0.05
100 km
Wasting
AP
0.400
0.375
0.350
0.325
100 km

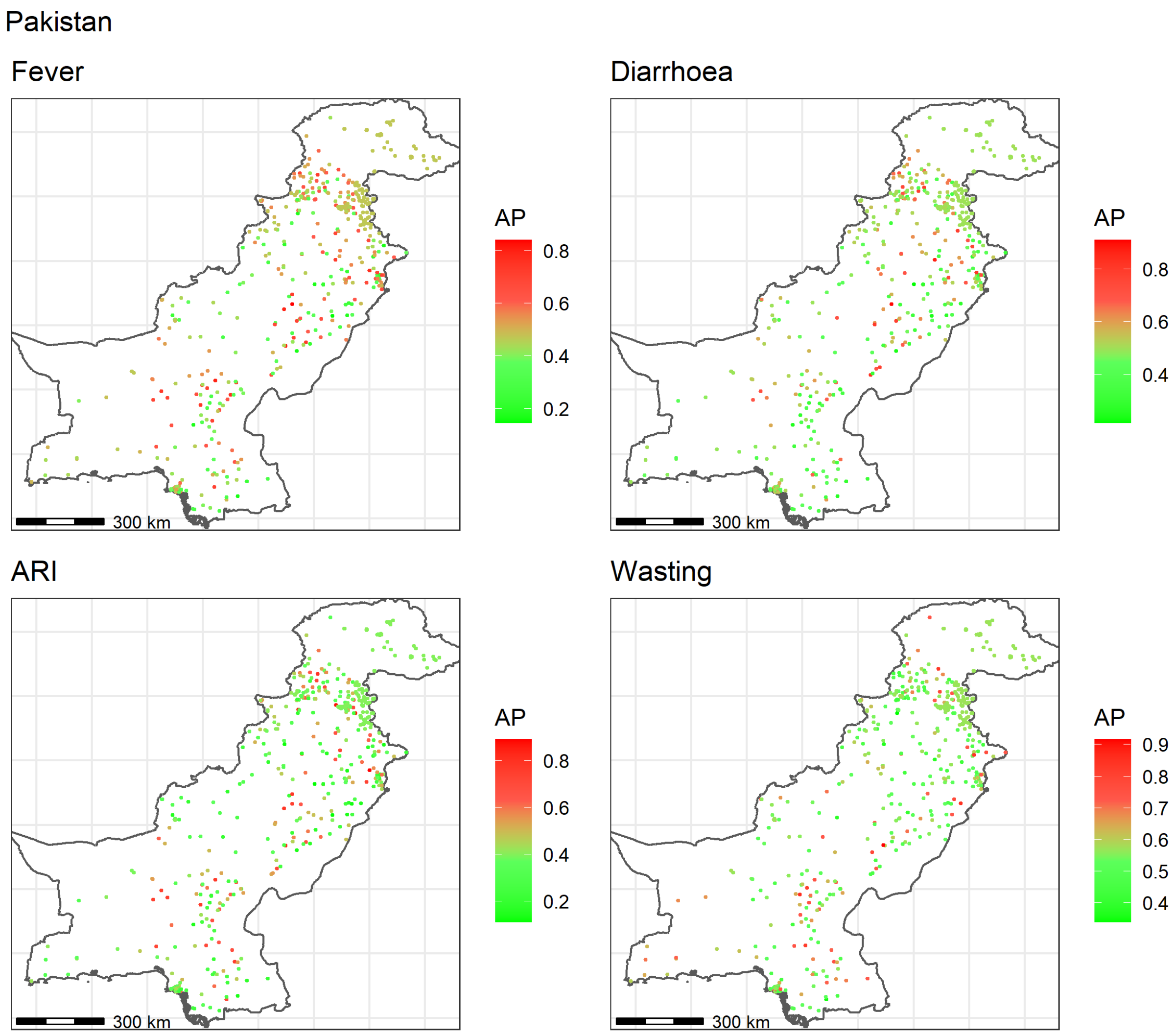
Pakistan
Fever
AP
0.8
0.6
0.4
0.2
300 km
Diarrhoea
AP
0.8
0.6
0.4
300 km
ARI
AP
0.8
0.6
0.4
0.2
300 km
Wasting
AP
0.9
0.8
0.7
0.6
0.5
0.4
300 km

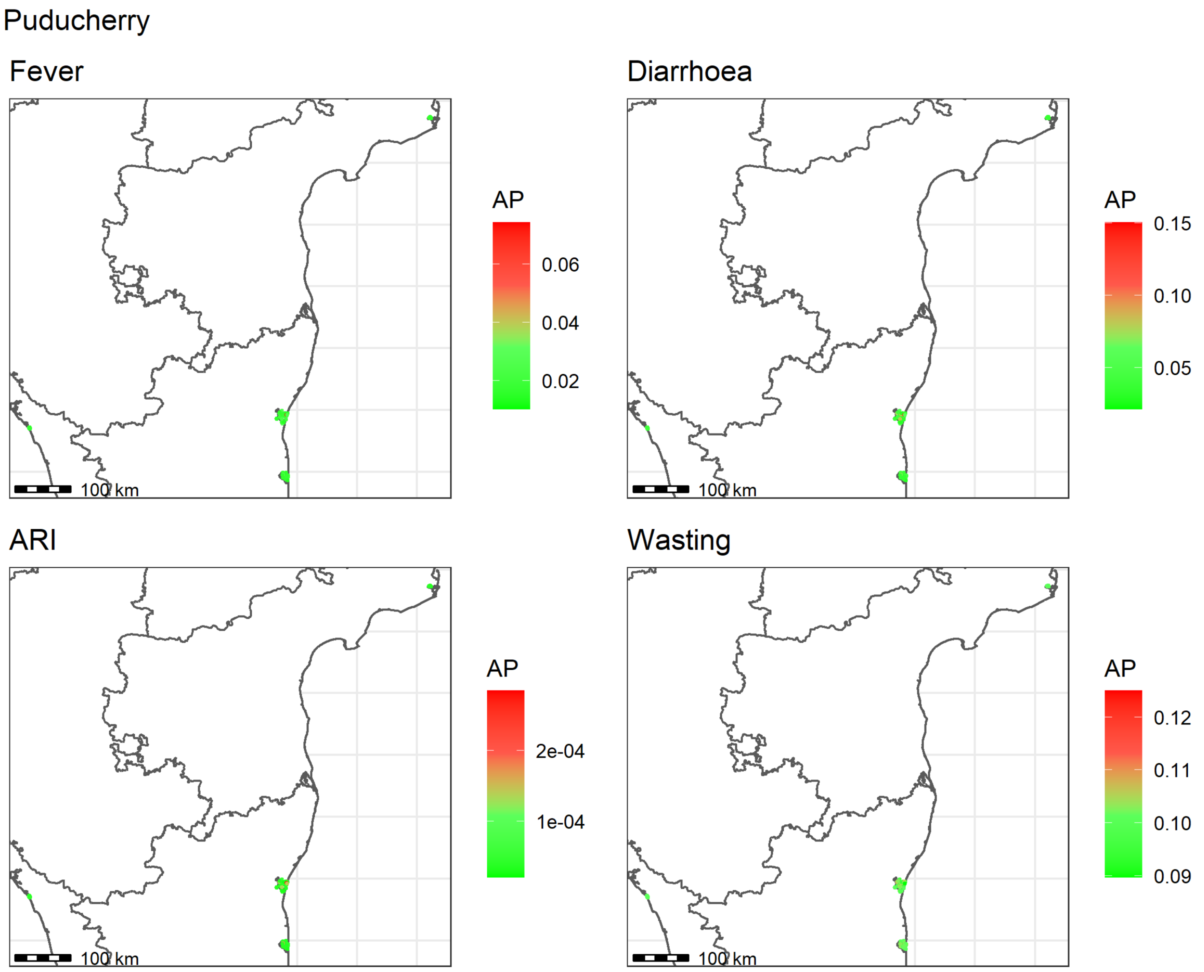
Puducherry
Fever
AP
0.06
0.04
0.02
100 km
Diarrhoea
AP
0.15
0.10
0.05
100 km
ARI
AP
2e-04
1e-04
100 km
Wasting
AP
0.12
0.11
0.10
0.09
100 km

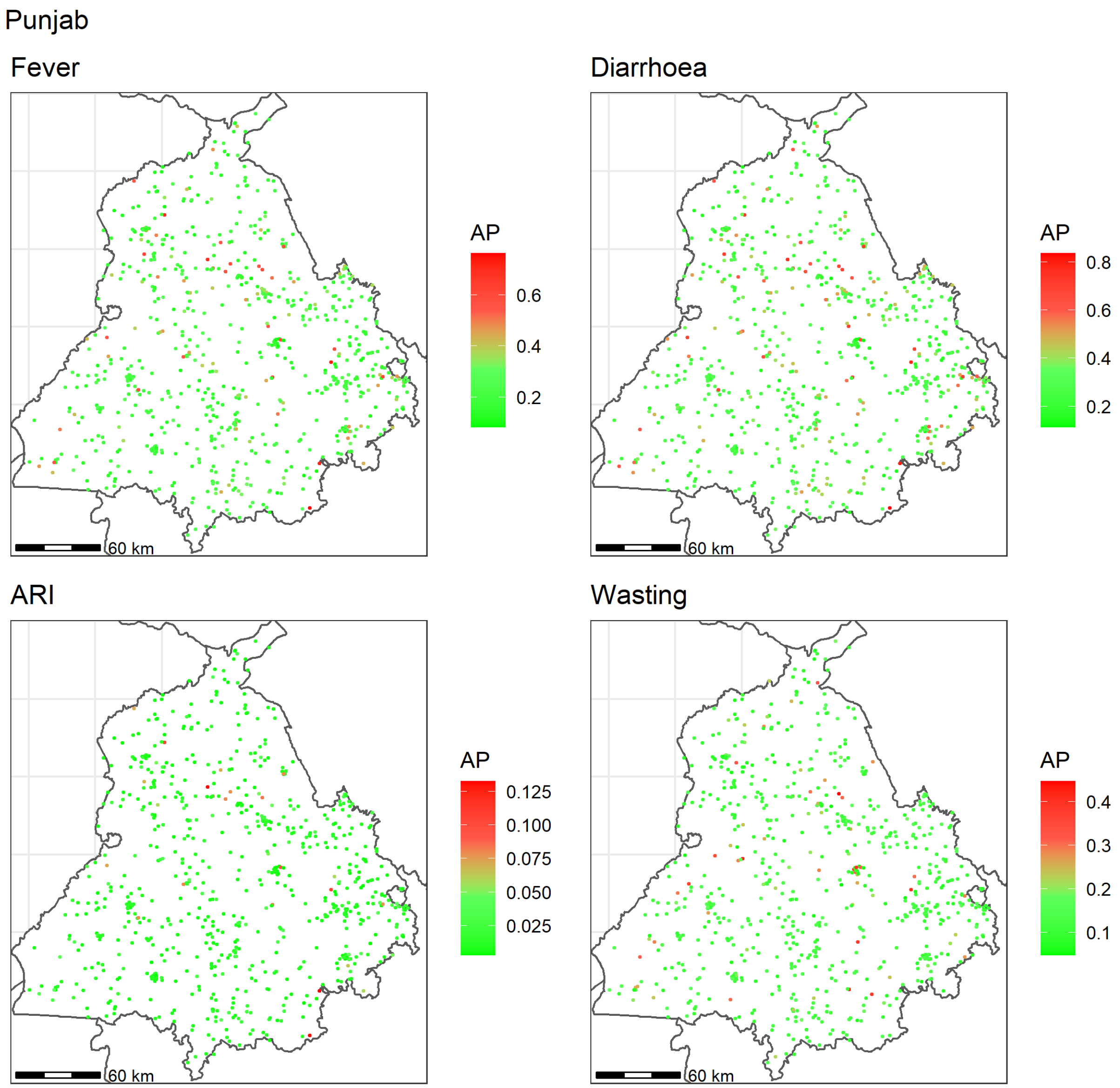
Punjab
Fever
AP
0.6
0.4
0.2
60 km
Diarrhoea
AP
0.8
0.6
0.4
0.2
60 km
ARI
AP
0.125
0.100
0.075
0.050
0.025
60 km
Wasting
AP
0.4
0.3
0.2
0.1
60 km

Rajasthan

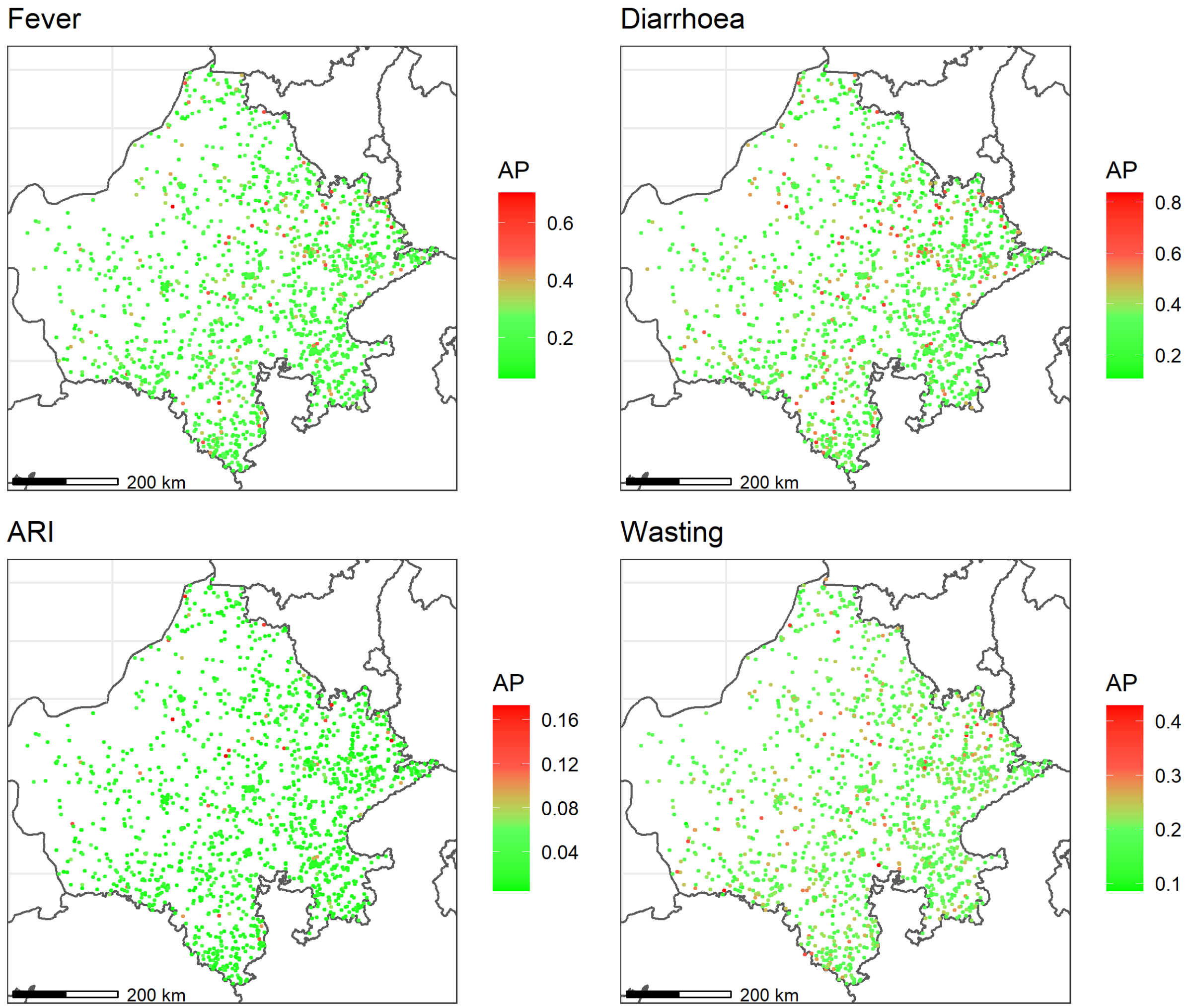
Fever
AP
0.6
0.4
0.2
200 km
Diarrhoea
AP
0.8
0.6
0.4
0.2
200 km
ARI
AP
0.16
0.12
0.08
0.04
200 km
Wasting
AP
0.4
0.3
0.2
0.1
200 km

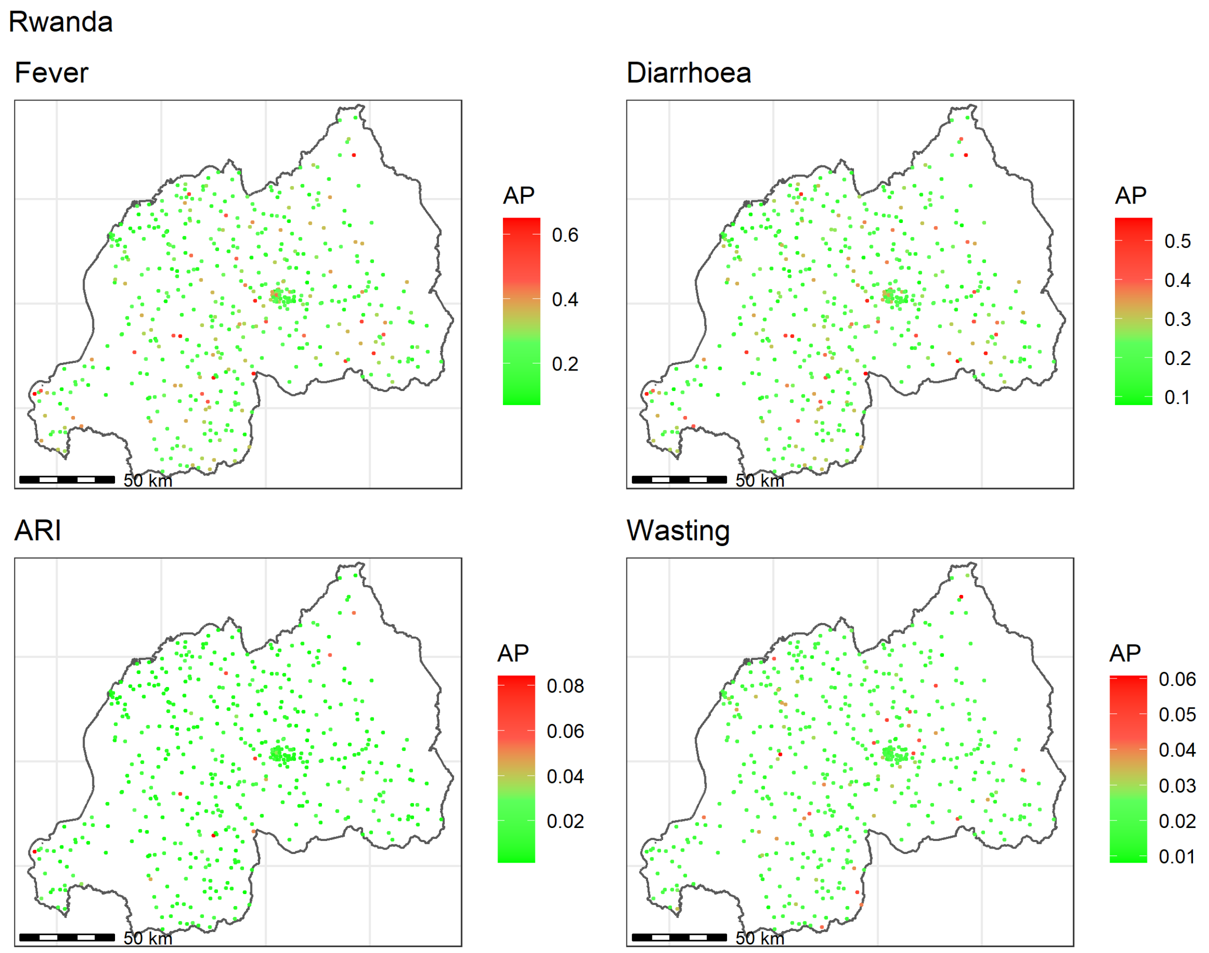

Rwanda
Fever
AP
0.6
0.4
0.2
50 km
Diarrhoea
AP
0.5
0.4
0.3
0.2
0.1
50 km
ARI
AP
0.08
0.06
0.04
0.02
50 km
Wasting
AP
0.06
0.05
0.04
0.03
0.02
0.01
50 km

Senegal

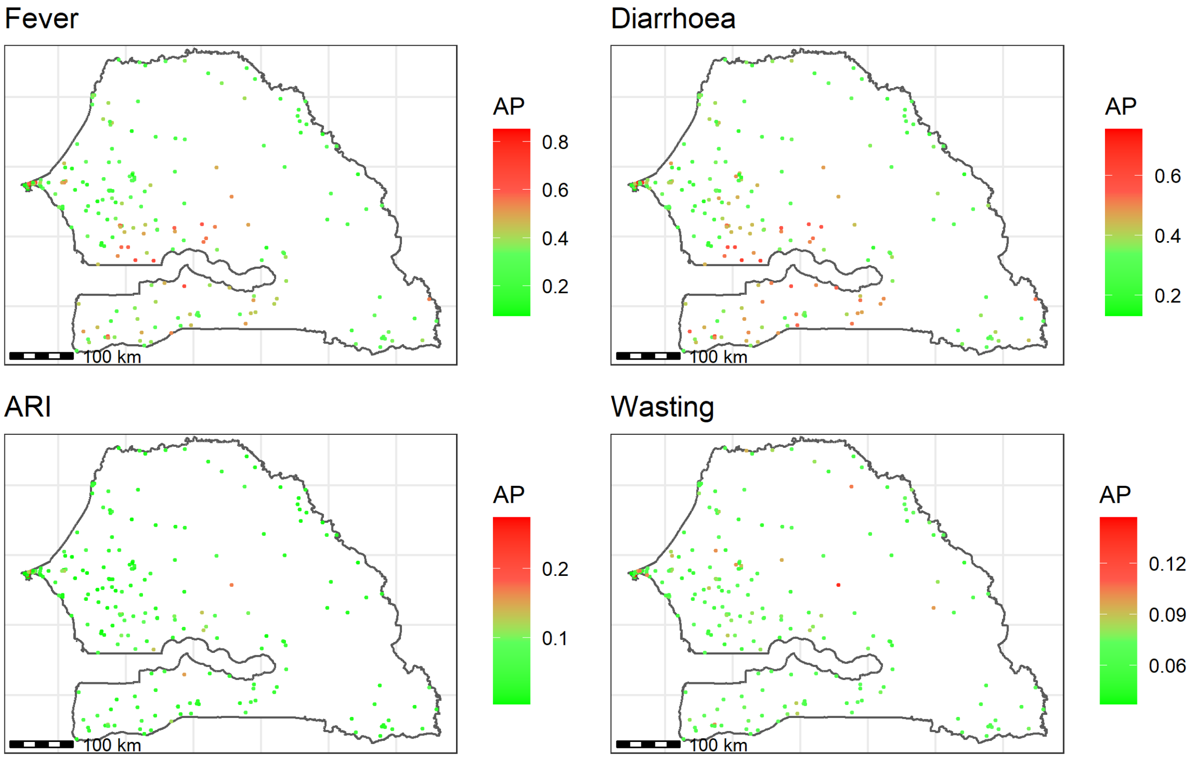

Fever
AP
0.8
0.6
0.4
0.2
100 km
Diarrhoea
AP
0.6
0.4
0.2
100 km
ARI
AP
0.2
0.1
100 km
Wasting
AP
0.12
0.09
0.06
100 km

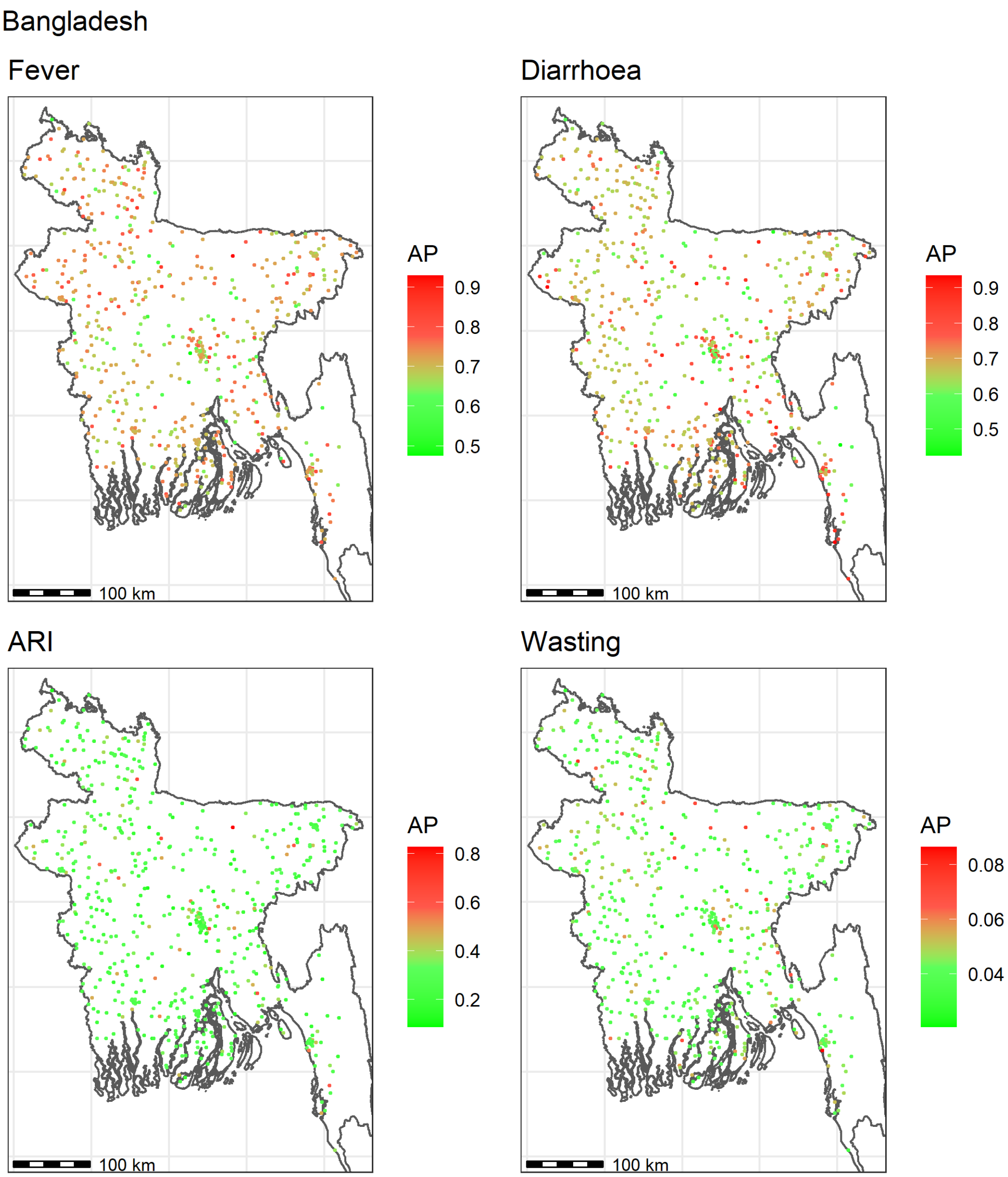
Bangladesh
Fever
Diarrhoea
ARI
Wasting
AP
0.9
0.8
0.7
0.6
0.5
AP
0.9
0.8
0.7
0.6
0.5
AP
0.8
0.6
0.4
0.2
AP
0.08
0.06
0.04
100 km
100 km
100 km
100 km

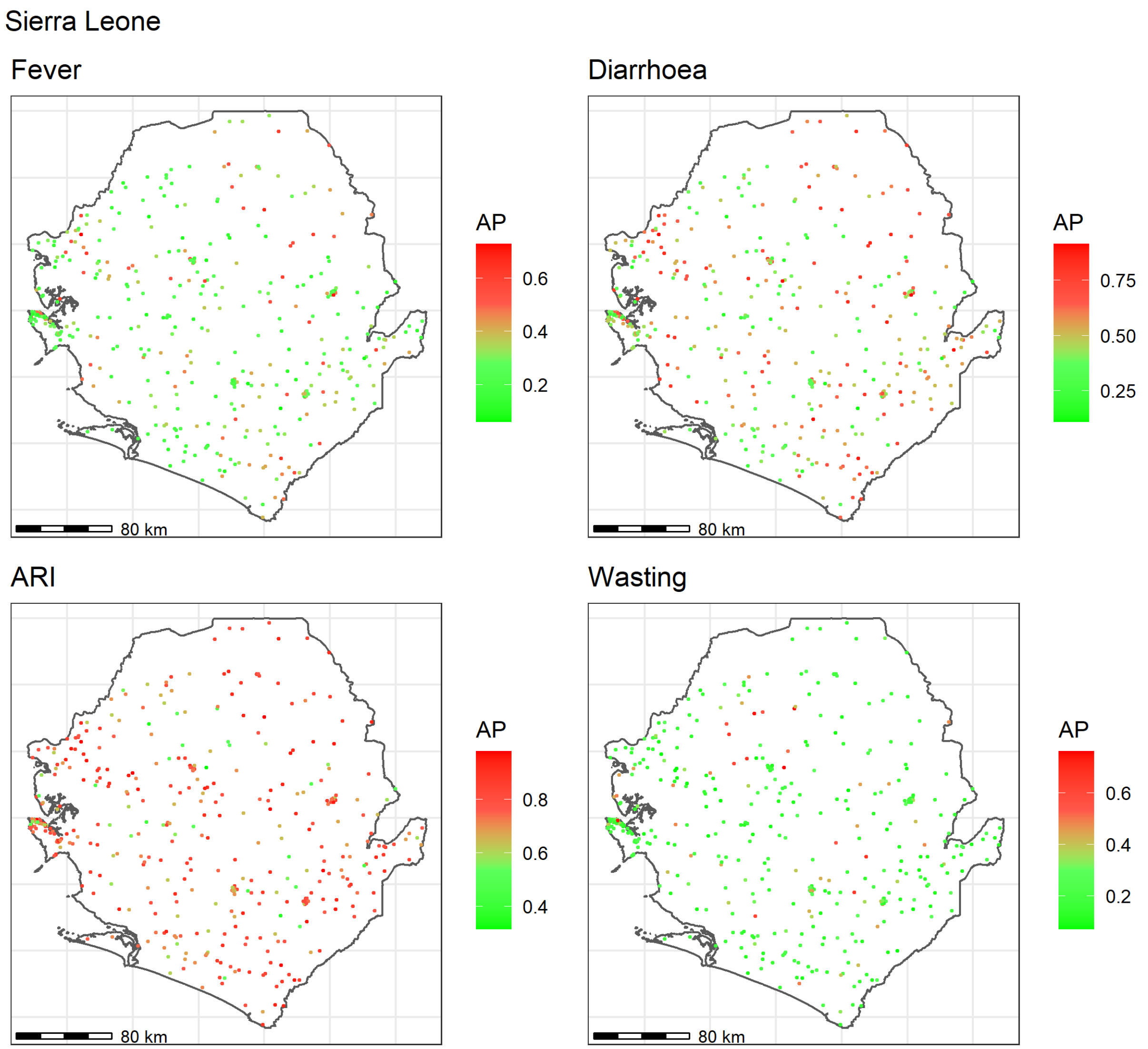
Sierra Leone
Fever
AP
0.6
0.4
0.2
80 km
Diarrhoea
AP
0.75
0.50
0.25
80 km
ARI
AP
0.8
0.6
0.4
80 km
Wasting
AP
0.6
0.4
0.2
80 km

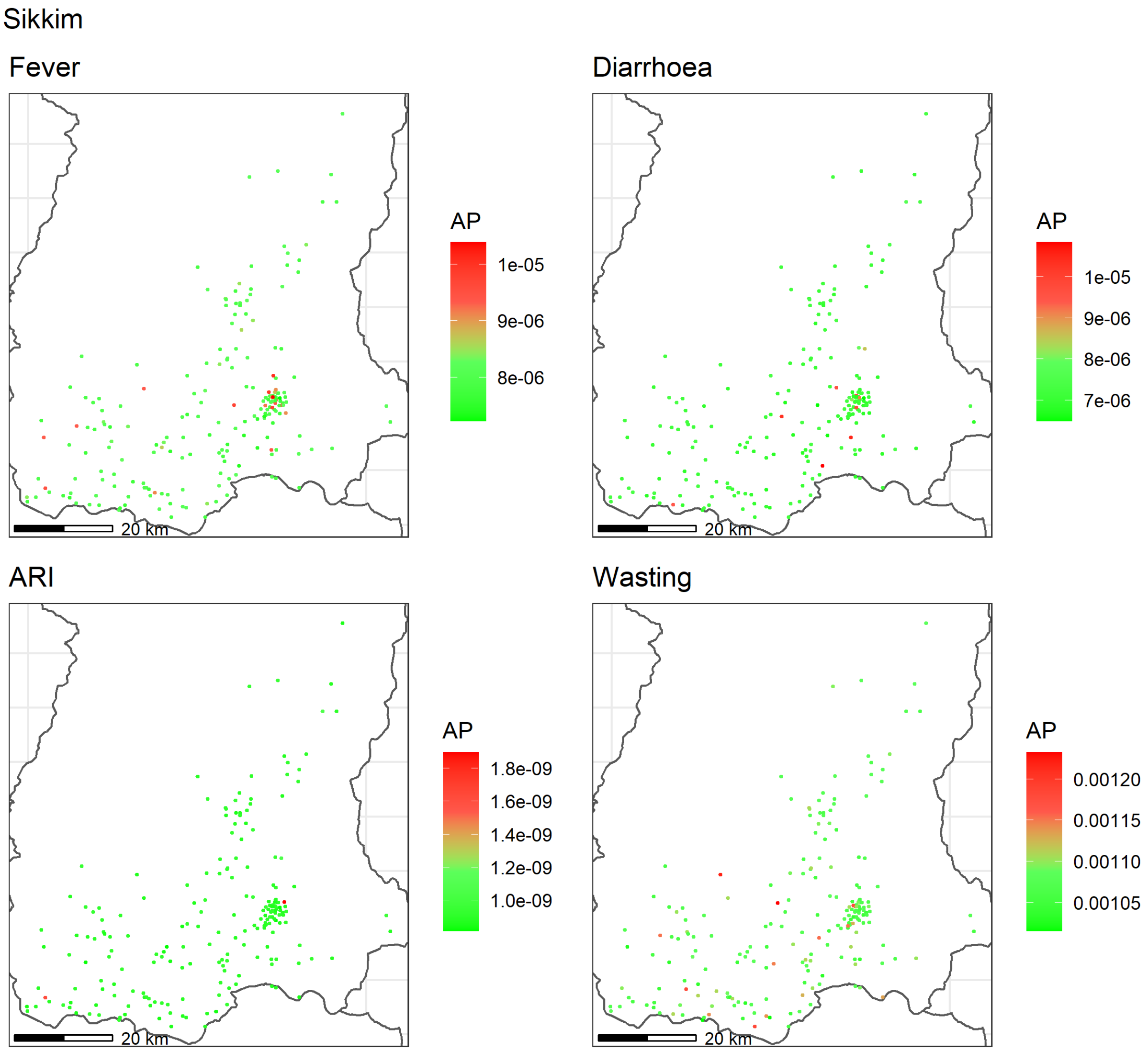

Sikkim
Fever
AP
1e-05
9e-06
8e-06
20 km
Diarrhoea
AP
1e-05
9e-06
8e-06
7e-06
20 km
ARI
AP
1.8e-09
1.6e-09
1.4e-09
1.2e-09
1.0e-09
20 km
Wasting
AP
0.00120
0.00115
0.00110
0.00105
20 km

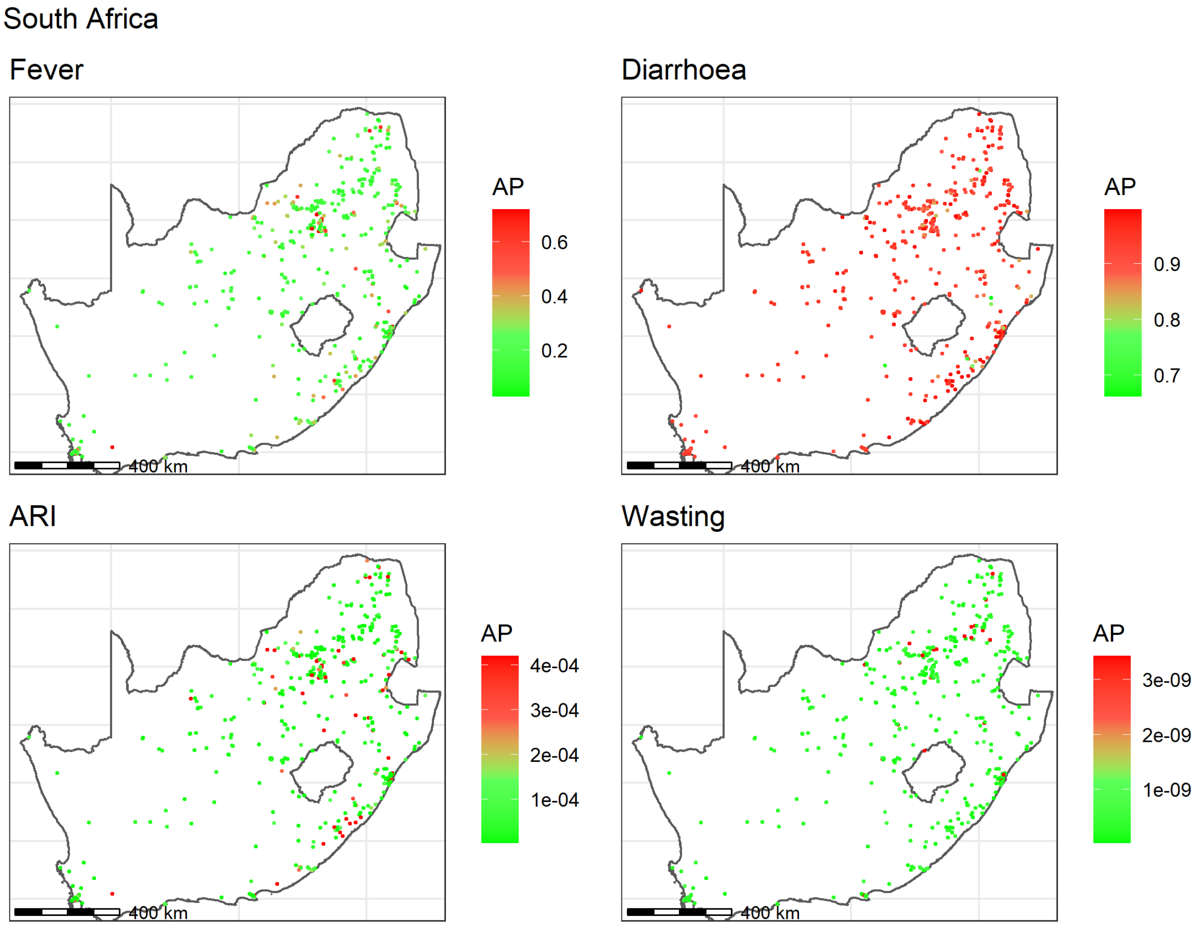
South Africa
Fever
AP
0.6
0.4
0.2
400 km
Diarrhoea
AP
0.9
0.8
0.7
400 km
ARI
AP
4e-04
3e-04
2e-04
1e-04
400 km
Wasting
AP
3e-09
2e-09
1e-09
400 km

Tamil Nadu

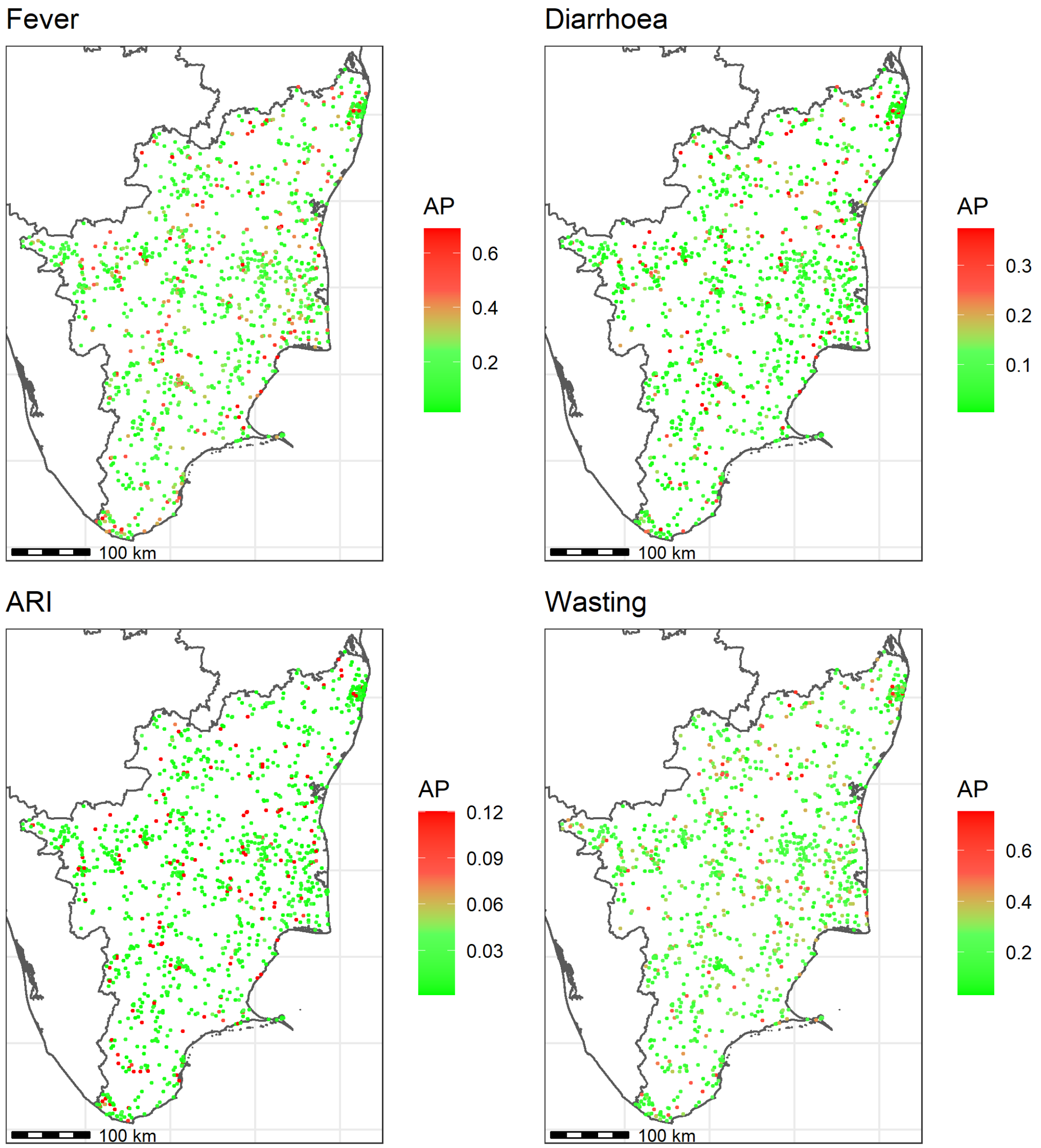

Fever
AP
0.6
0.4
0.2
100 km
Diarrhoea
AP
0.3
0.2
0.1
100 km
ARI
AP
0.12
0.09
0.06
0.03
100 km
Wasting
AP
0.6
0.4
0.2
100 km

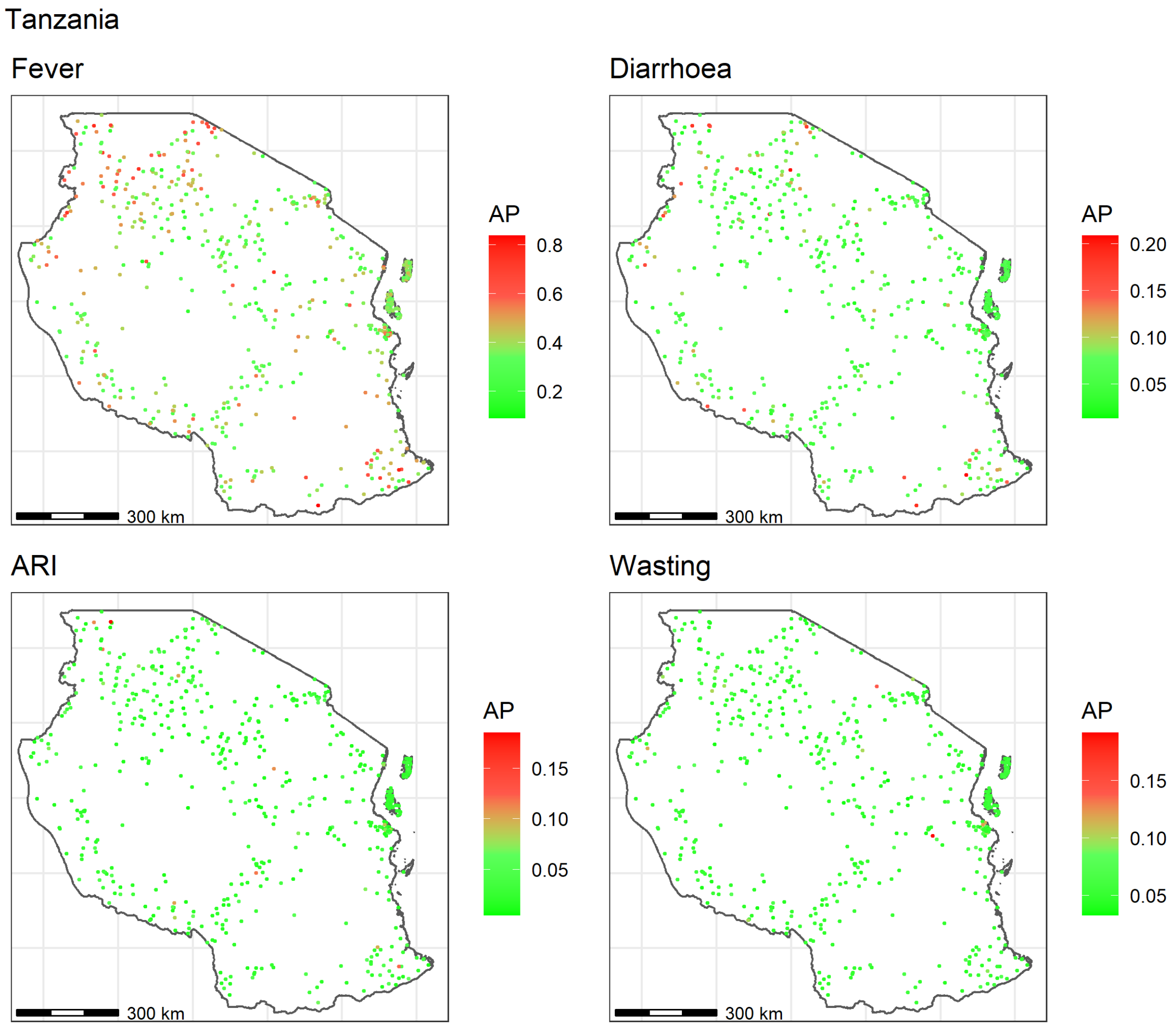

Tanzania
Fever
AP
0.8
0.6
0.4
0.2
300 km
Diarrhoea
AP
0.20
0.15
0.10
0.05
300 km
ARI
AP
0.15
0.10
0.05
300 km
Wasting
AP
0.15
0.10
0.05
300 km

Telangana

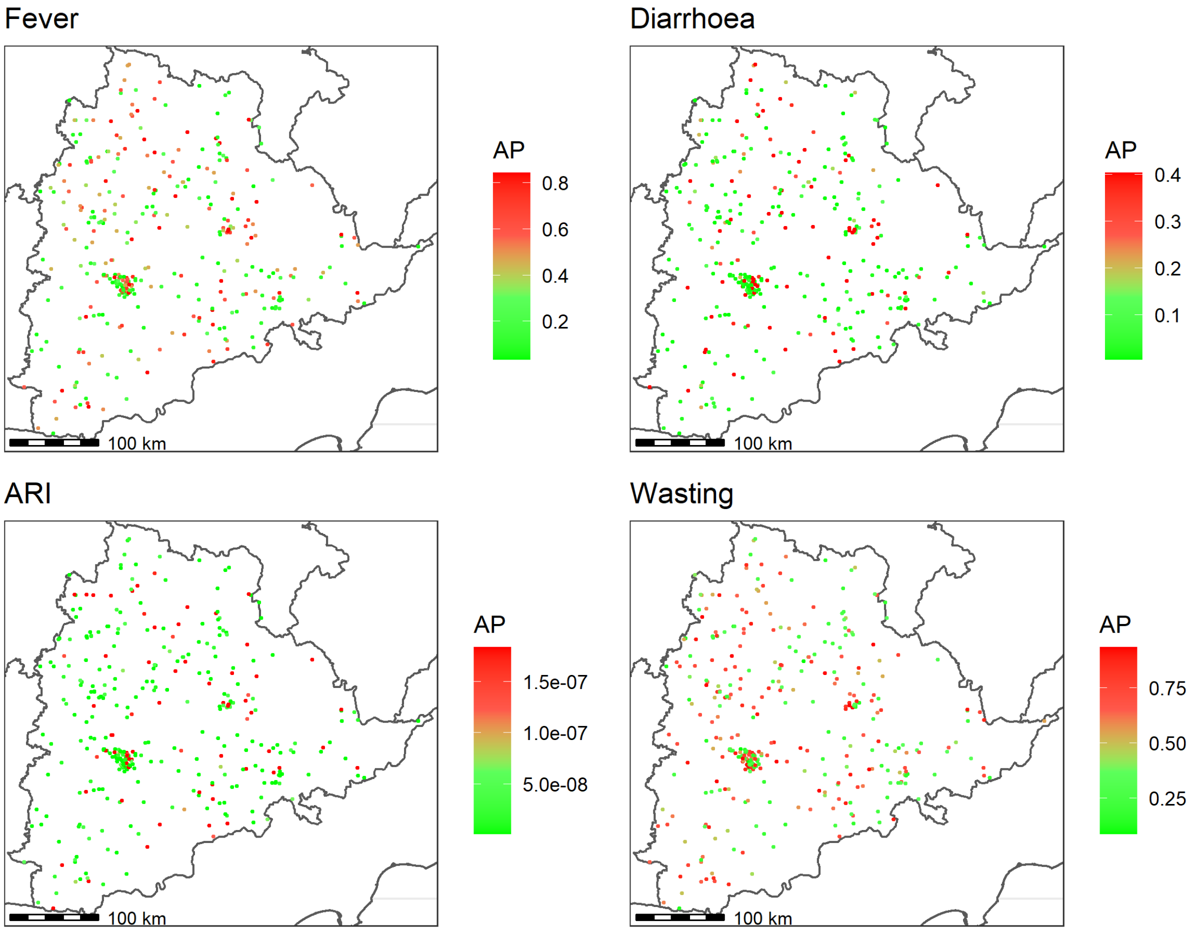
Fever
AP
0.8
0.6
0.4
0.2
100 km
Diarrhoea
AP
0.4
0.3
0.2
0.1
100 km
ARI
AP
1.5e-07
1.0e-07
5.0e-08
100 km
Wasting
AP
0.75
0.50
0.25
100 km

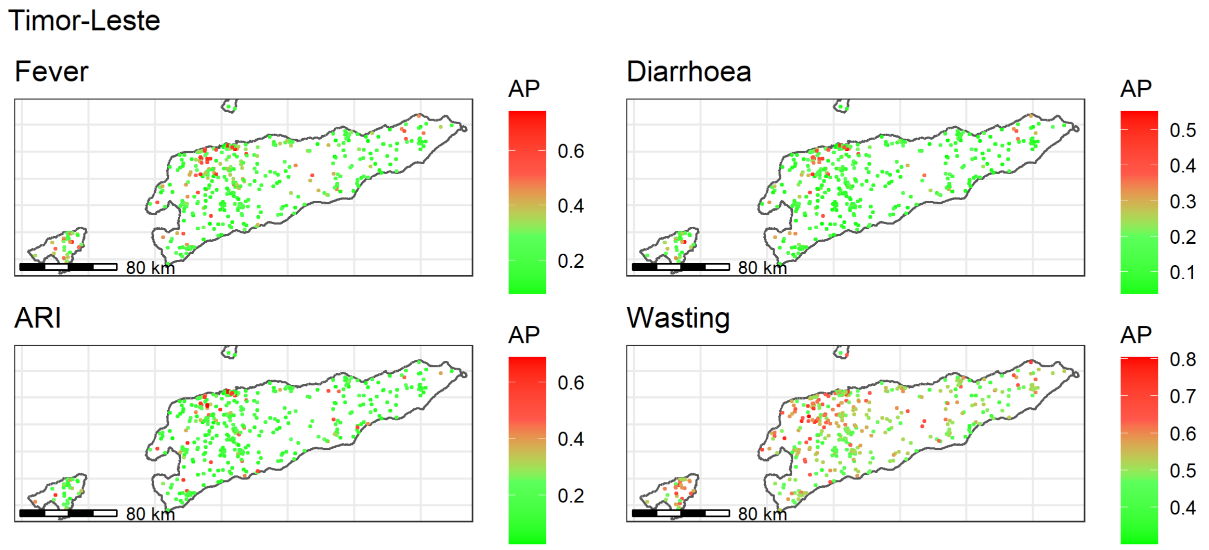
Timor-Leste
Fever
AP
0.6
0.4
0.2
80 km
Diarrhoea
AP
0.5
0.4
0.3
0.2
0.1
80 km
ARI
AP
0.6
0.4
0.2
80 km
Wasting
AP
0.8
0.7
0.6
0.5
0.4
80 km

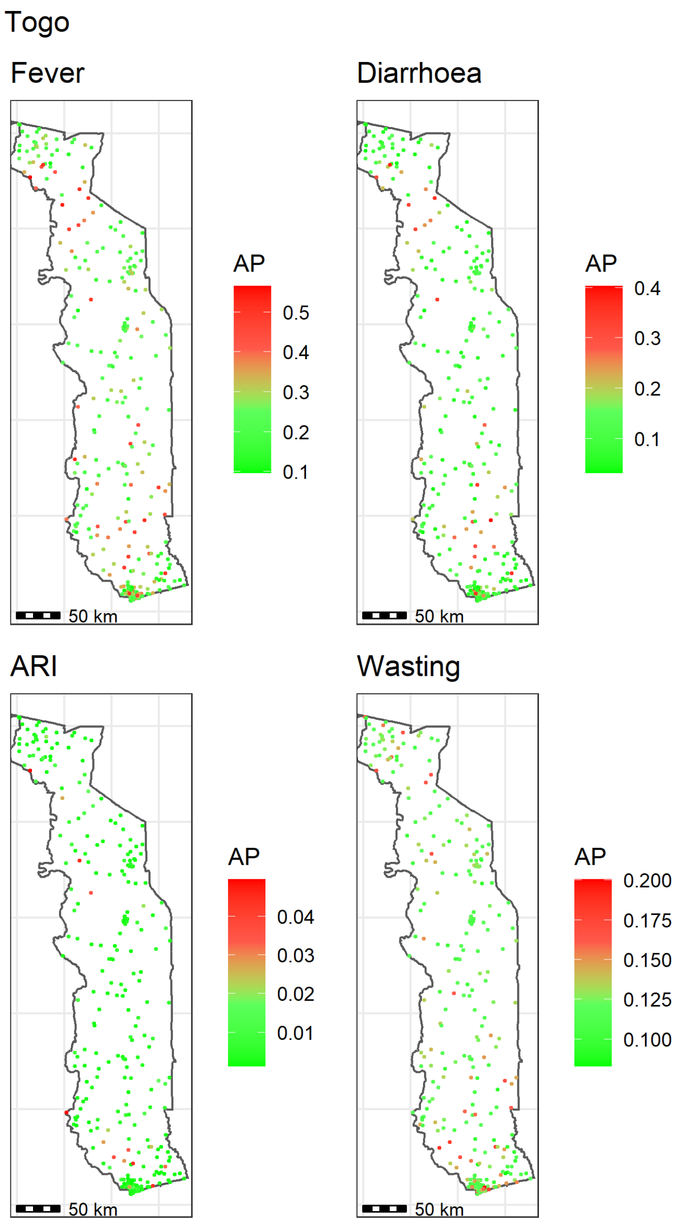
Togo
Fever
AP
0.5
0.4
0.3
0.2
0.1
50 km
Diarrhoea
AP
0.4
0.3
0.2
0.1
50 km
ARI
AP
0.04
0.03
0.02
0.01
50 km
Wasting
AP
0.200
0.175
0.150
0.125
0.100
50 km

Tripura

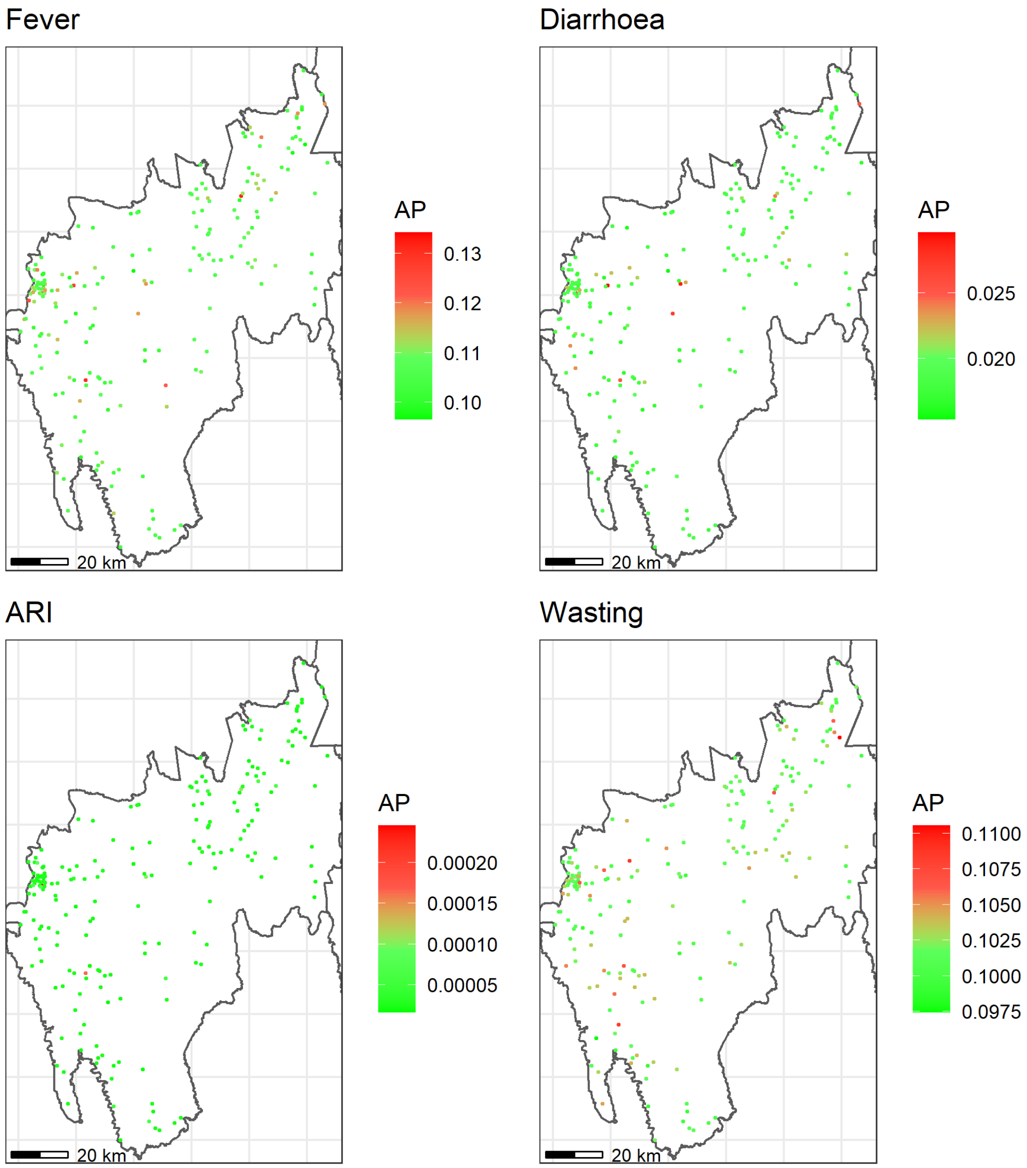
Fever
AP
0.13
0.12
0.11
0.10
20 km
Diarrhoea
AP
0.025
0.020
20 km
ARI
AP
0.00020
0.00015
0.00010
0.00005
20 km
Wasting
AP
0.1100
0.1075
0.1050
0.1025
0.1000
0.0975
20 km

Uganda

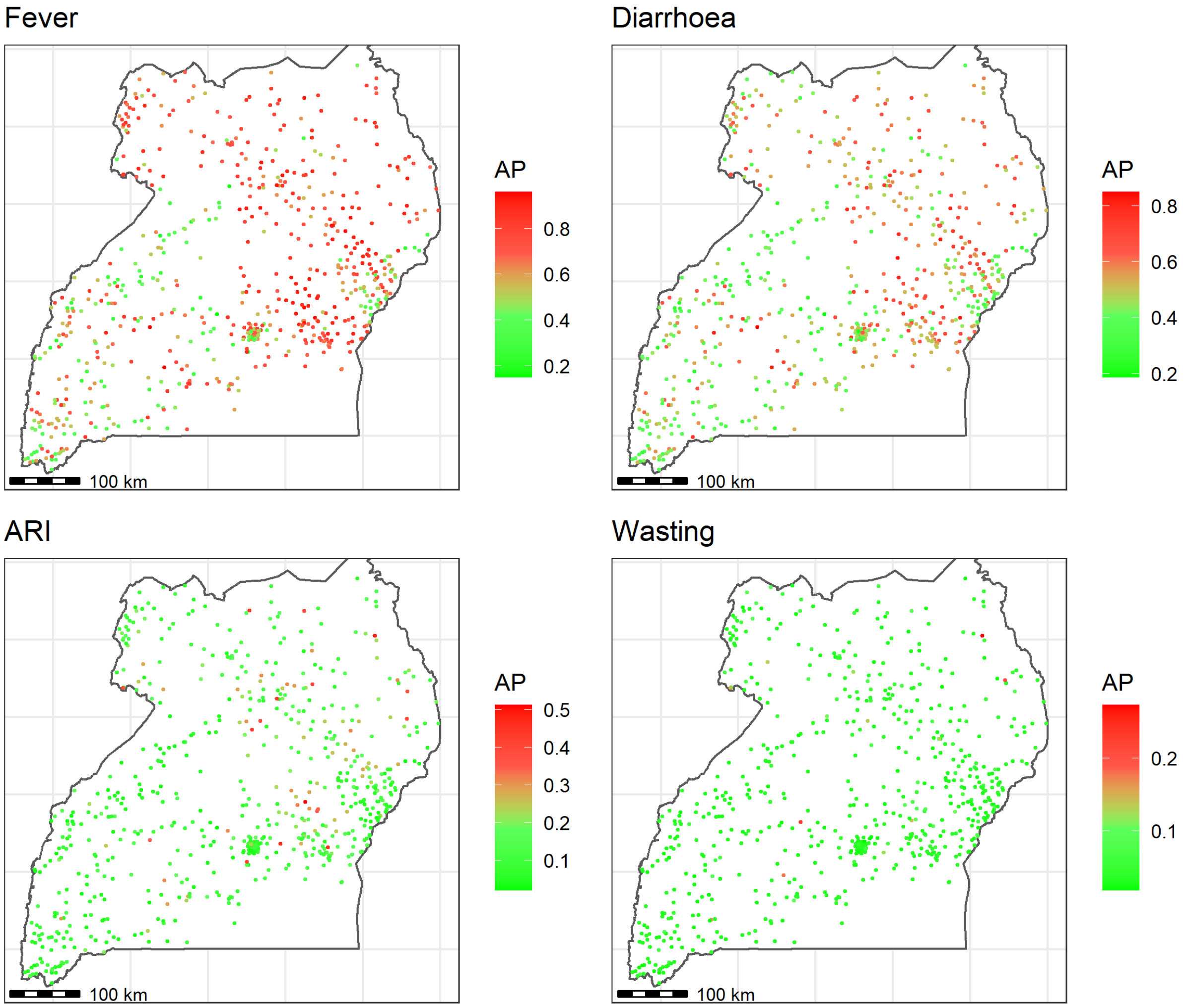
Fever
AP
0.8
0.6
0.4
0.2
100 km
Diarrhoea
AP
0.8
0.6
0.4
0.2
100 km
ARI
AP
0.5
0.4
0.3
0.2
0.1
100 km
Wasting
AP
0.2
0.1
100 km

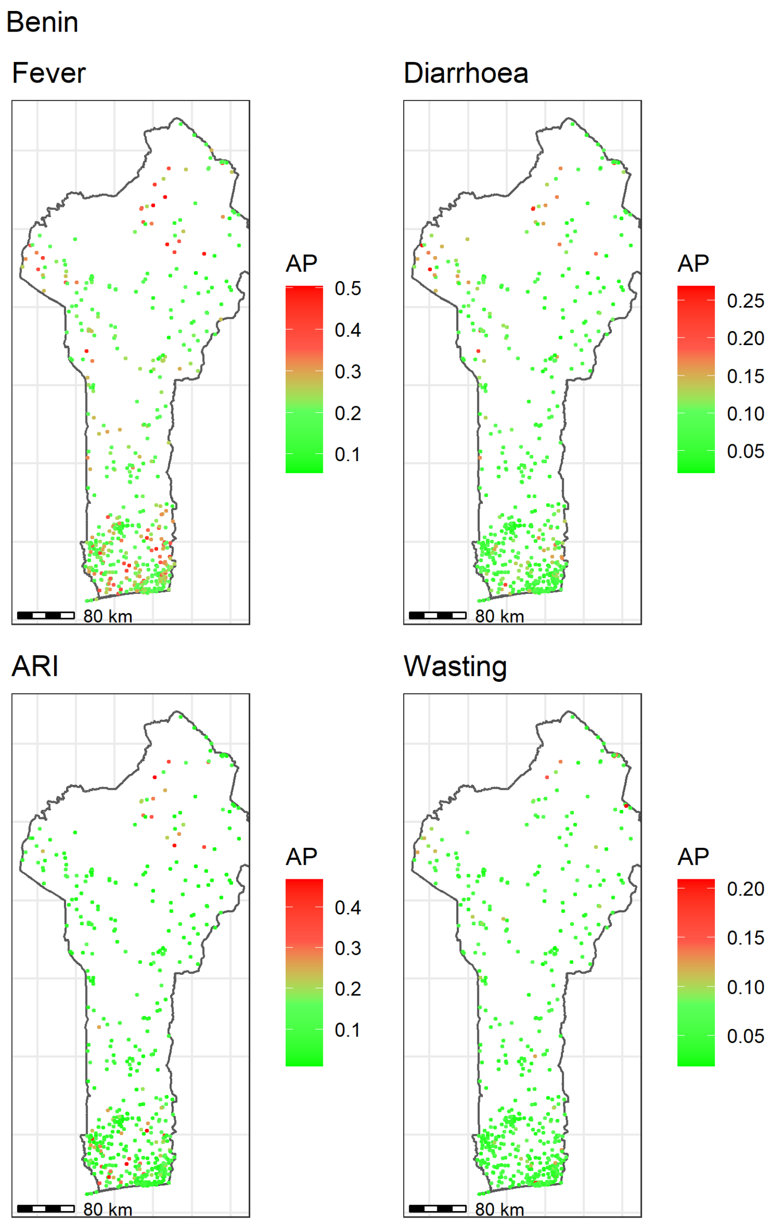
Benin
Fever
AP
0.5
0.4
0.3
0.2
0.1
80 km
Diarrhoea
AP
0.25
0.20
0.15
0.10
0.05
80 km
ARI
AP
0.4
0.3
0.2
0.1
80 km
Wasting
AP
0.20
0.15
0.10
0.05
80 km

Uttar Pradesh

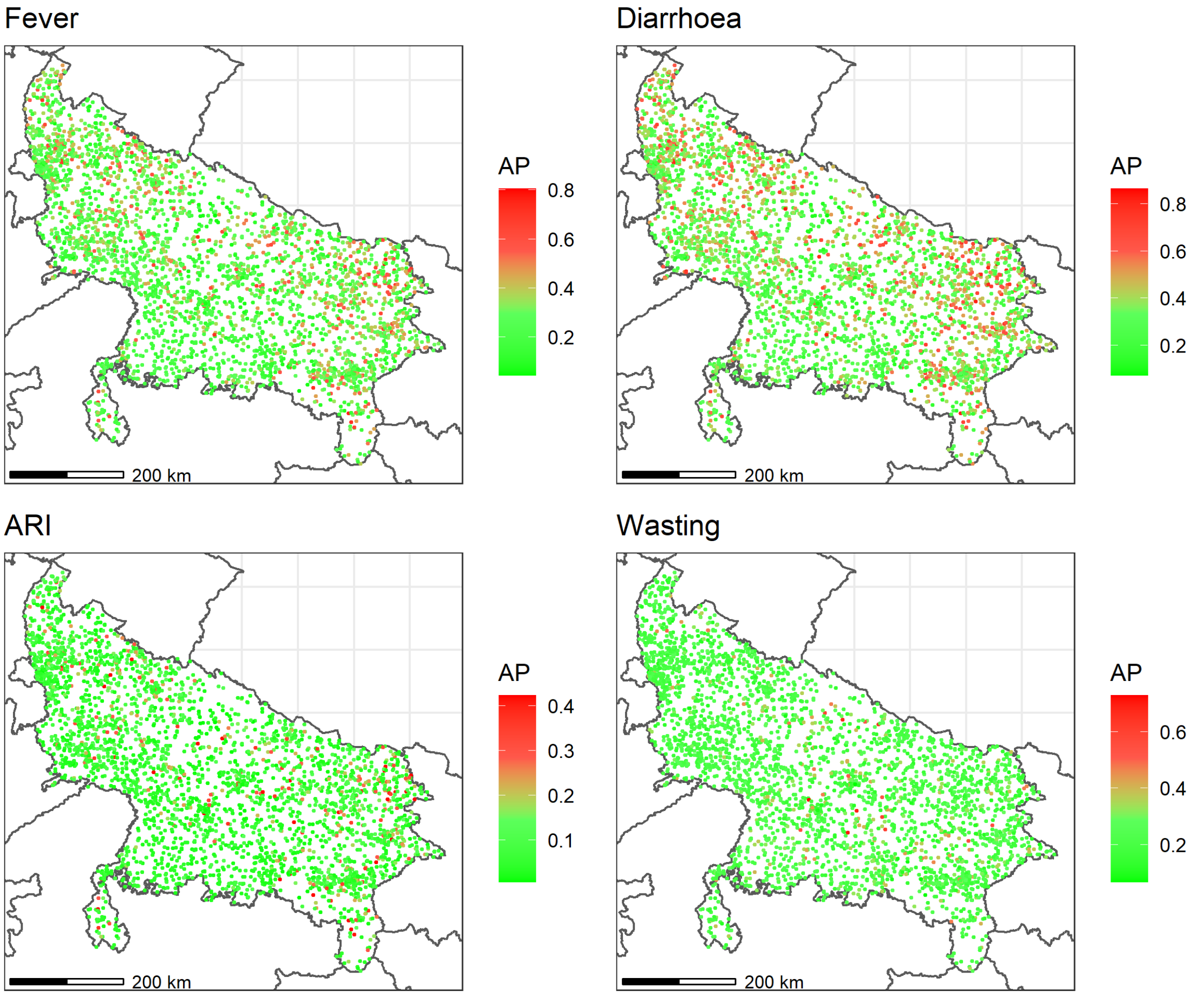

Uttarakhand

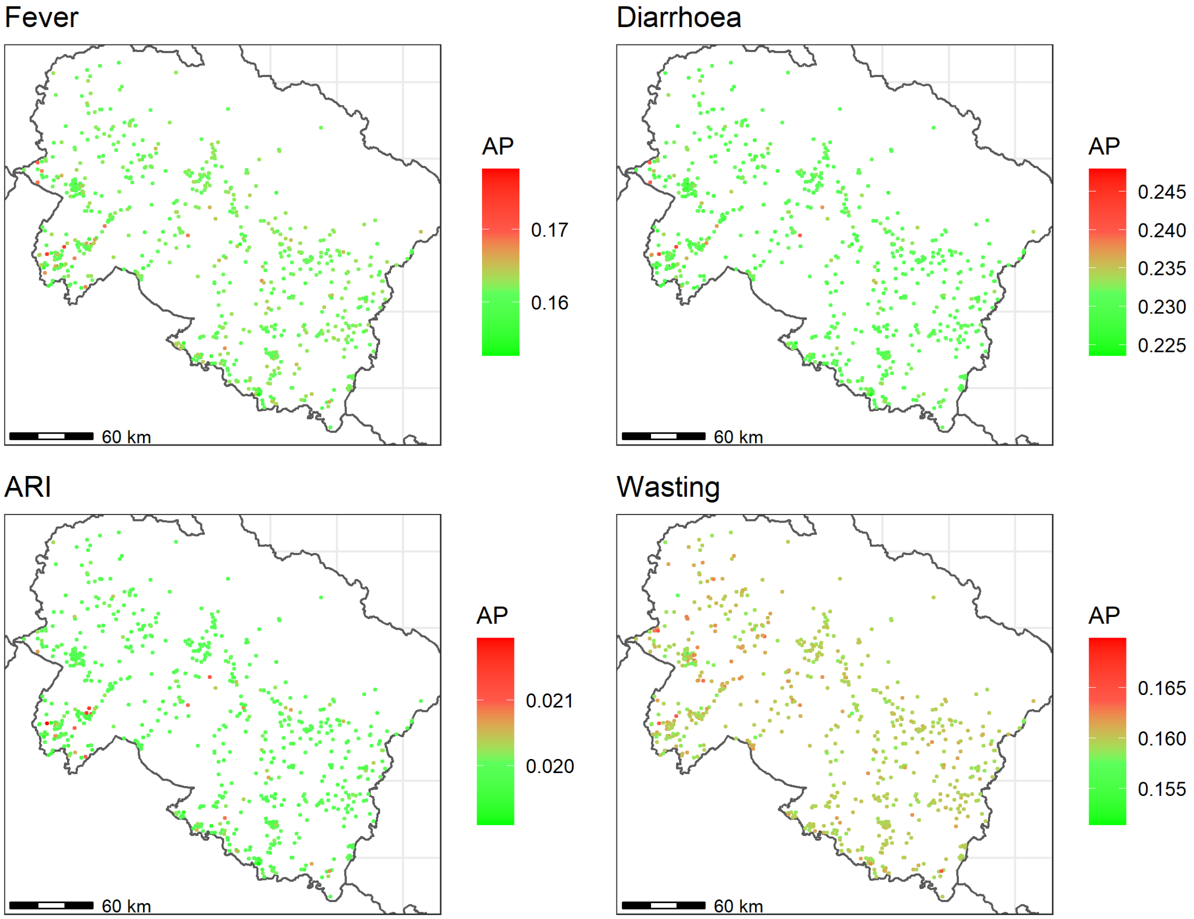

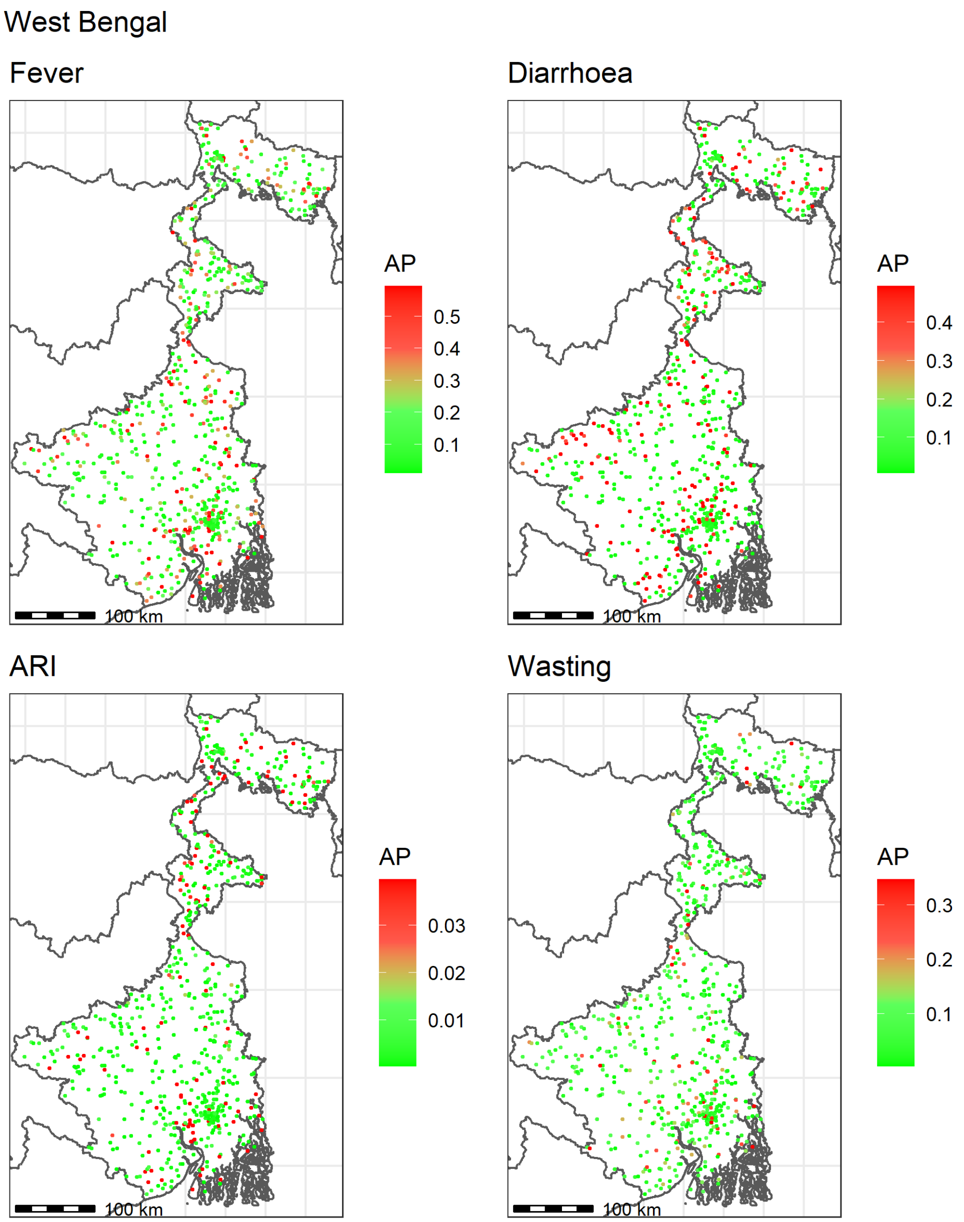
West Bengal
Fever
AP
0.5
0.4
0.3
0.2
0.1
100 km
Diarrhoea
AP
0.4
0.3
0.2
0.1
100 km
ARI
AP
0.03
0.02
0.01
100 km
Wasting
AP
0.3
0.2
0.1
100 km

Zambia

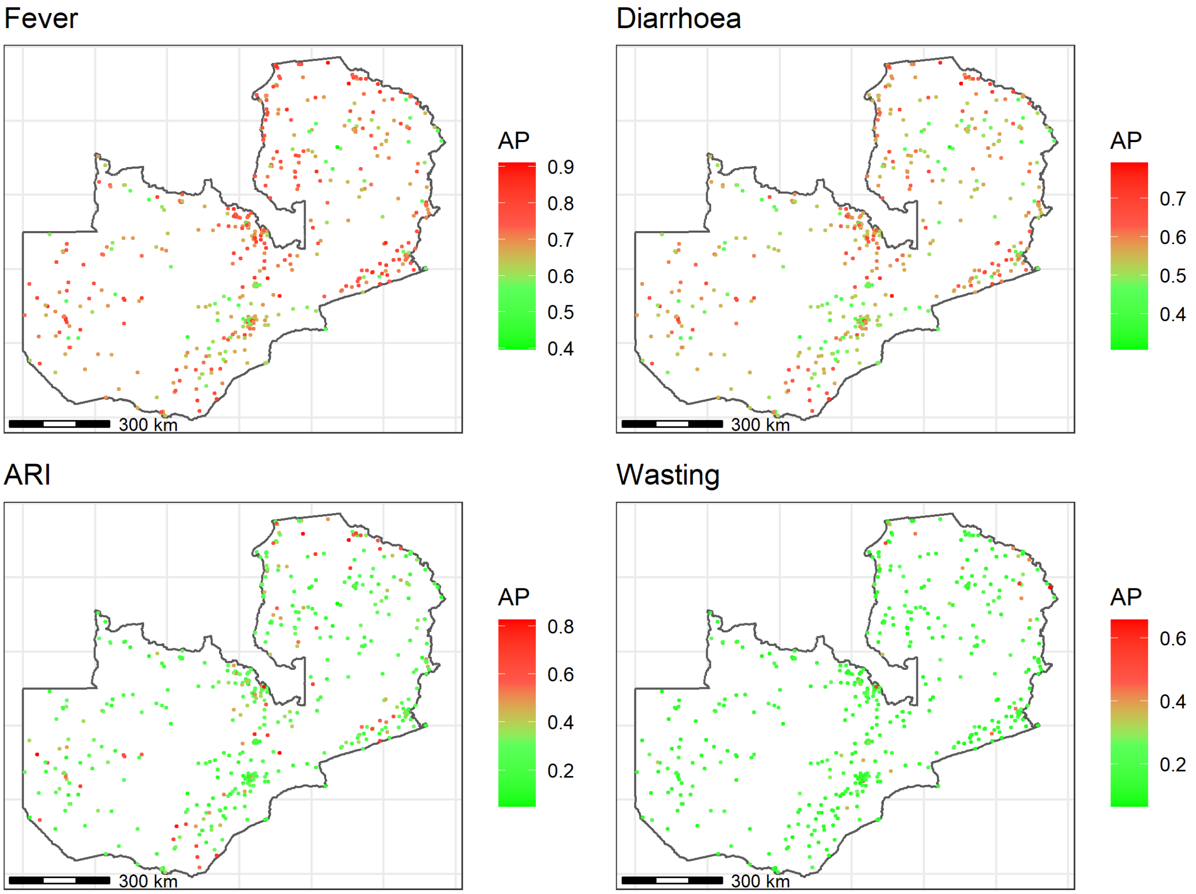

Fever
AP
0.9
0.8
0.7
0.6
0.5
0.4
300 km
Diarrhoea
AP
0.7
0.6
0.5
0.4
300 km
ARI
AP
0.8
0.6
0.4
0.2
300 km
Wasting
AP
0.6
0.4
0.2
300 km

Zimbabwe

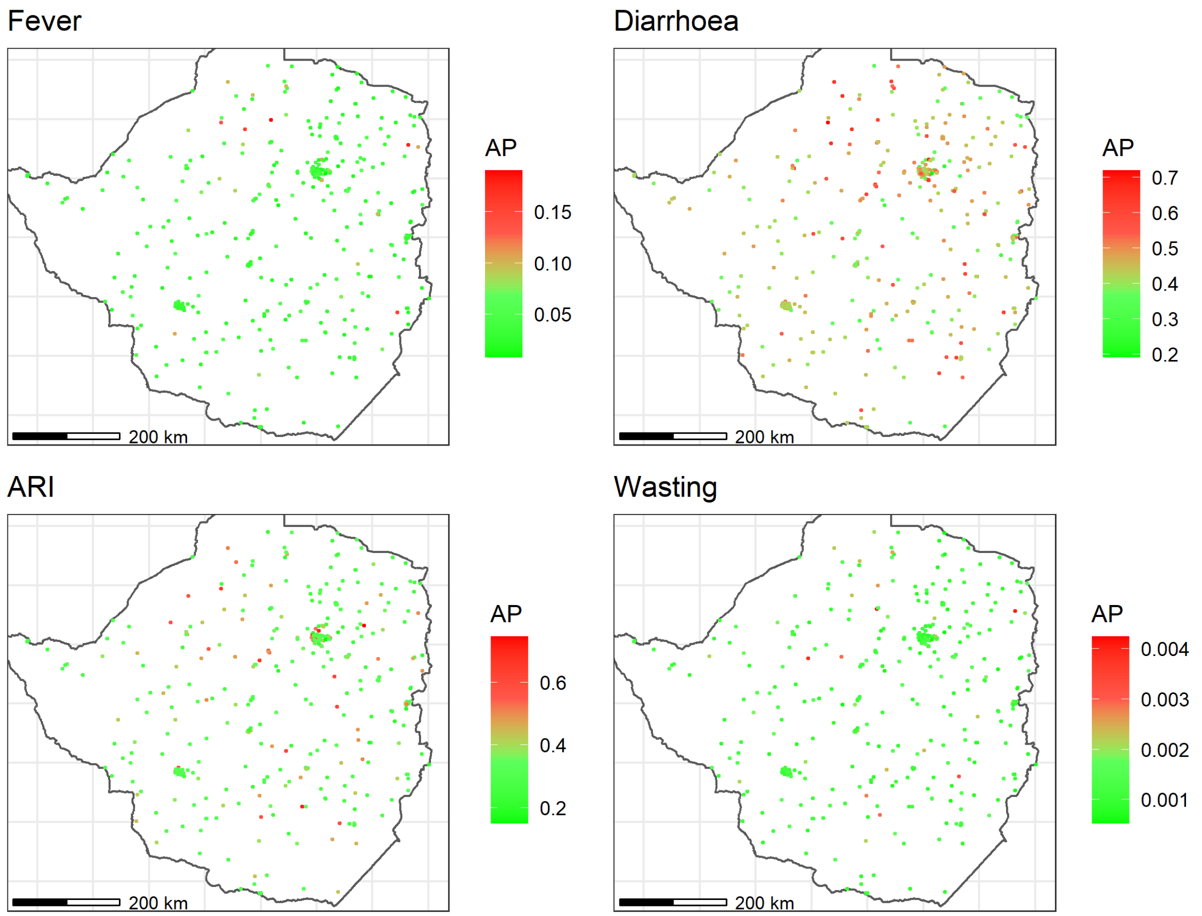
Fever
AP
0.15
0.10
0.05
200 km
Diarrhoea
AP
0.7
0.6
0.5
0.4
0.3
0.2
200 km
ARI
AP
0.6
0.4
0.2
200 km
Wasting
AP
0.004
0.003
0.002
0.001
200 km

Bihar

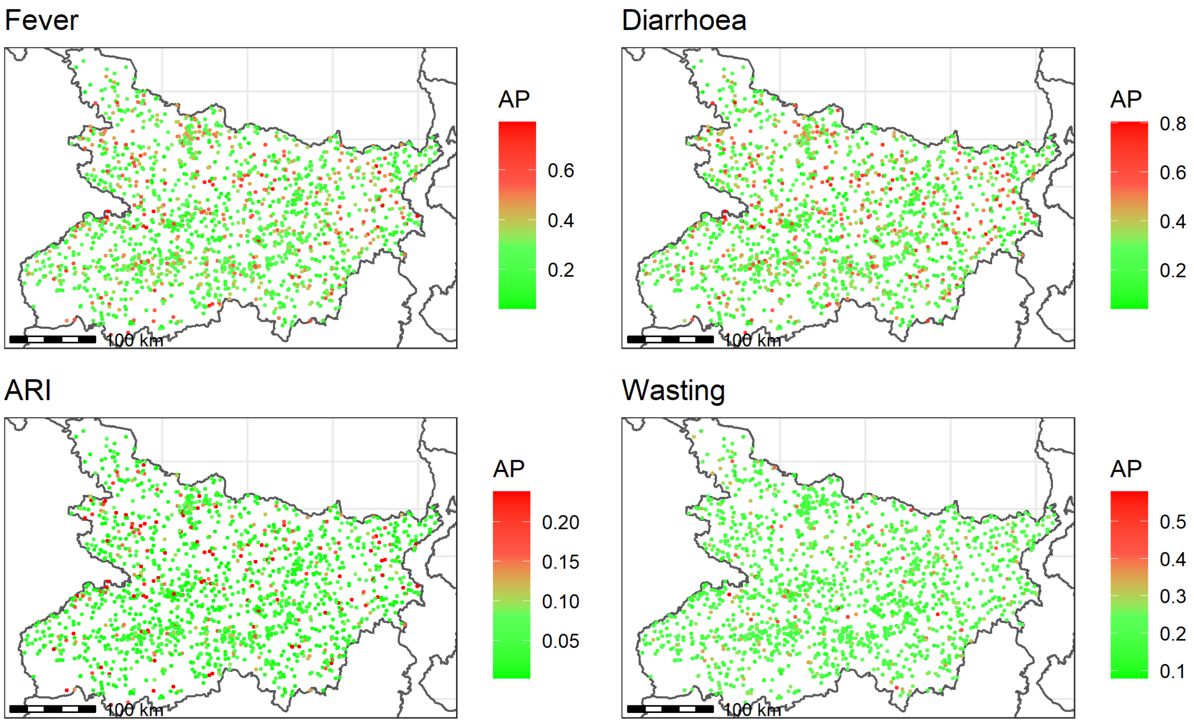

Fever
AP
0.6
0.4
0.2
100 km
Diarrhoea
AP
0.8
0.6
0.4
0.2
100 km
ARI
AP
0.20
0.15
0.10
0.05
100 km
Wasting
AP
0.5
0.4
0.3
0.2
0.1
100 km

Burkina Faso

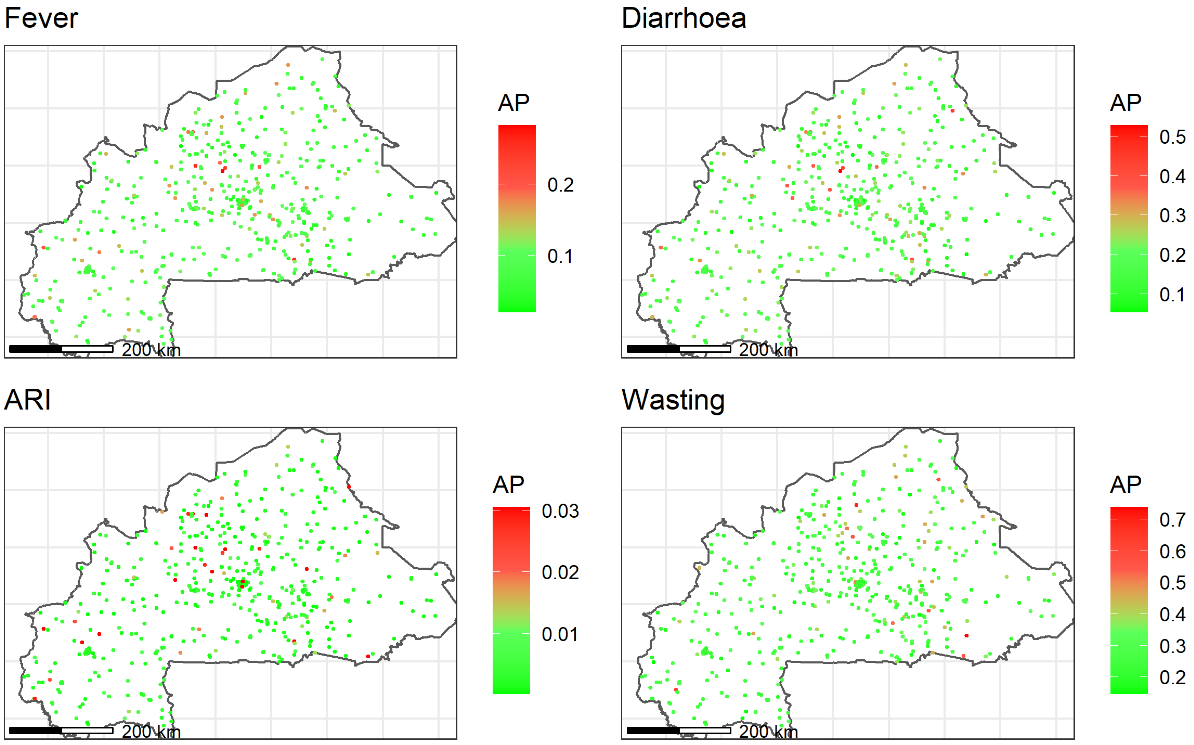

Supplement: Supplementary file 5 — Additional file 5. Spatial distribution of adjusted prevalence (AP). [file 12916_2021_2018_MOESM5_ESM.pdf]
