# Supplementary material for: Health inequities and clustering of fever, acute respiratory infection, diarrhoea and wasting in children under five in low- and middle-income countries: a Demographic and Health Surveys analysis
Source: BMC Med. 2021 Jun 24;19:144. doi: 10.1186/s12916-021-02018-0 (PMC8223394; doi:10.1186/s12916-021-02018-0)

Health inequities and clustering of fever, acute respiratory infection, diarrhoea and wasting in children under five in low- and middle-income countries: A Demographic and Health Surveys analysis.

Supplementary Information: Model fit

Posterior predictive checks

		Grouped			
	All	Wealth	Access	Urban/Rural	Cluster
**% of checks < 0.05 or > 0.95**	3.4	0.8	2	1.9	13.9

AUC

Country/Region	ARI	Diarrhoea	Fever	Wasted
Andaman & Nicobar Islands	0.67	0.61	0.70	0.74
Andhra Pradesh	1.00	0.95	0.93	0.85
Angola	0.86	0.80	0.80	0.79
Arunachal Pradesh	0.70	0.65	0.61	0.63
Assam	0.76	0.88	0.81	0.71
Bangladesh	0.76	0.78	0.68	0.65
Benin	0.87	0.79	0.73	0.78
Bihar	0.92	0.86	0.83	0.71
Burkina Faso	0.92	0.78	0.76	0.81
Burundi	0.81	0.75	0.73	0.80
Cambodia	0.86	0.80	0.75	0.74
Cameroon	0.93	0.80	0.74	0.86
Chad	0.84	0.77	0.77	0.73
Chandigarh	0.94	NA	0.95	0.96
Chhattisgarh	0.77	0.75	0.76	0.67
Comoros	0.94	0.83	0.78	0.70
Congo Democratic Republic	0.75	0.77	0.76	0.77
Cote d'Ivoire	0.89	0.75	0.70	0.82
Dadra & Nagar Haveli	0.85	0.97	0.84	0.67
Daman & Diu	0.88	0.79	0.81	0.72
Egypt	0.80	0.79	0.77	0.90
Ethiopia	0.81	0.73	0.72	0.74
Gabon	0.70	0.72	0.66	0.70
Ghana	0.88	0.83	0.84	0.87
Goa	0.91	0.93	0.87	0.72
Guinea	0.91	0.79	0.74	0.83
Gujarat	0.95	0.87	0.88	0.80
Haiti	0.78	0.76	0.72	0.78
Haryana	0.82	0.78	0.80	0.74
Himachal Pradesh	0.72	0.73	0.66	0.59
Jammu & Kashmir	0.65	0.65	0.62	0.61
Jharkhand	0.84	0.79	0.79	0.64
Karnataka	0.97	0.91	0.90	0.79
Kenya	0.73	0.76	0.74	0.79
Kerala	0.99	0.97	0.91	0.89
Lakshadweep	0.67	0.84	0.77	0.63
Lesotho	0.91	0.86	0.85	0.93

Country/Region	ARI	Diarrhoea	Fever	Wasted
Liberia	0.77	0.73	0.71	0.80
Madhya Pradesh	0.86	0.80	0.80	0.62
Maharashtra	0.96	0.87	0.86	0.79
Malawi	0.82	0.77	0.77	0.86
Mali	0.85	0.77	0.74	0.76
Manipur	0.68	0.65	0.66	0.61
Meghalaya	0.71	0.63	0.64	0.65
Mizoram	0.72	0.69	0.70	0.64
Mozambique	0.84	0.76	0.74	0.83
Myanmar	0.76	0.77	0.72	0.76
Nagaland	0.77	0.73	0.72	0.62
Namibia	0.92	0.83	0.81	0.90
NCT of Delhi	0.98	0.89	0.89	0.89
Nepal	0.82	0.75	0.75	0.79
Nigeria	0.87	0.81	0.77	0.80
Odisha	0.91	0.79	0.79	0.64
Pakistan	0.76	0.76	0.72	0.80
Puducherry	0.75	0.68	0.67	0.57
Punjab	0.85	0.83	0.78	0.80
Rajasthan	0.87	0.82	0.80	0.74
Rwanda	0.85	0.80	0.80	0.87
Senegal	0.80	0.75	0.75	0.68
Sierra Leone	0.87	0.83	0.77	0.80
Sikkim	0.82	0.86	0.81	0.66
South Africa	0.99	0.93	0.85	0.95
Tamil Nadu	0.95	0.90	0.87	0.81
Tanzania	0.80	0.79	0.75	0.70
Telangana	0.99	0.93	0.87	0.87
Timor-Leste	0.86	0.82	0.80	0.70
Togo	0.90	0.78	0.72	0.78
Tripura	0.90	0.81	0.74	0.70
Uganda	0.82	0.81	0.83	0.82
Uttar Pradesh	0.87	0.80	0.78	0.78
Uttarakhand	0.69	0.64	0.66	0.70
West Bengal	0.95	0.91	0.87	0.83
Zambia	0.85	0.74	0.72	0.79
Zimbabwe	0.83	0.74	0.76	0.83

Posterior predictive spatial checks

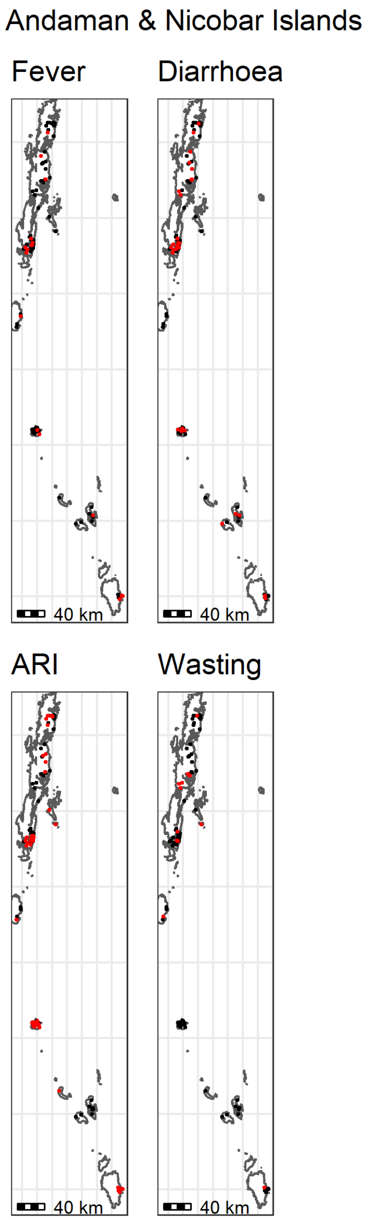

Burundi

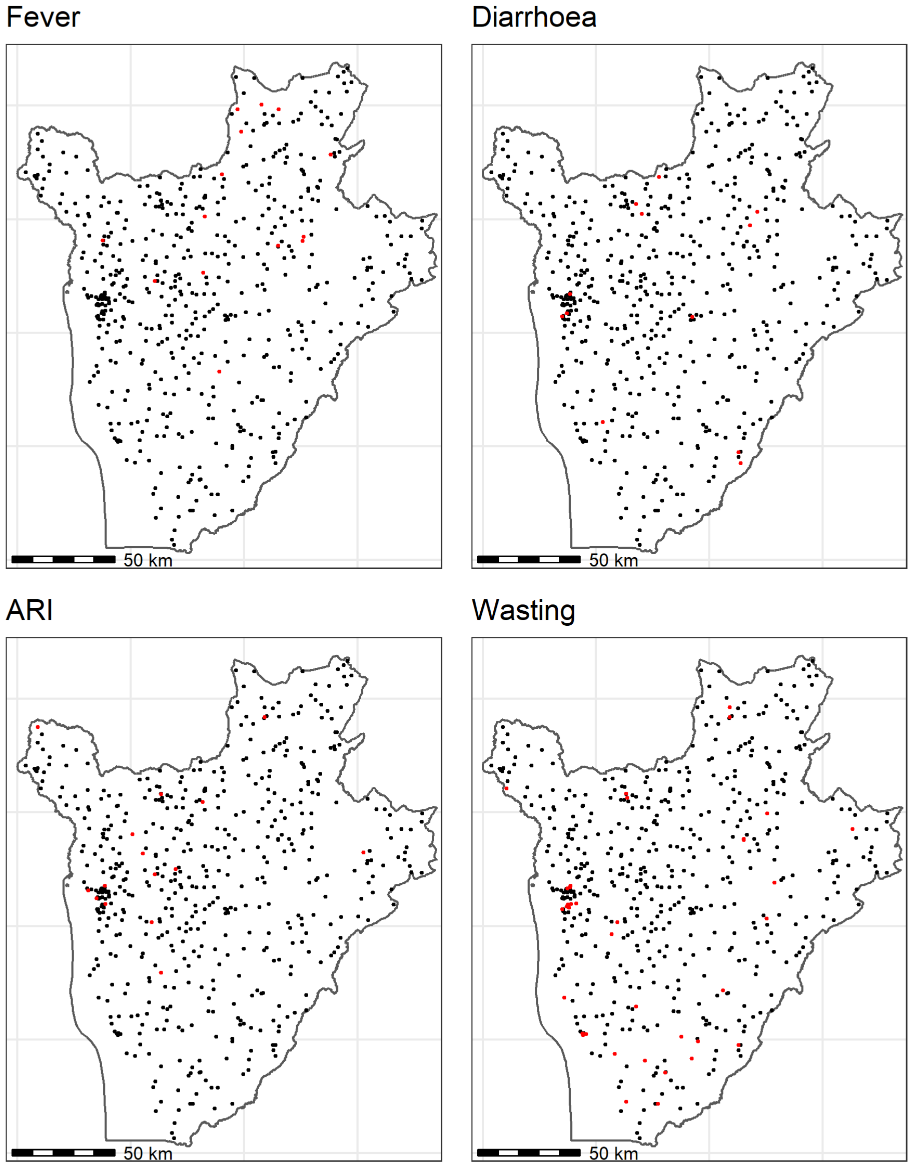

Cambodia

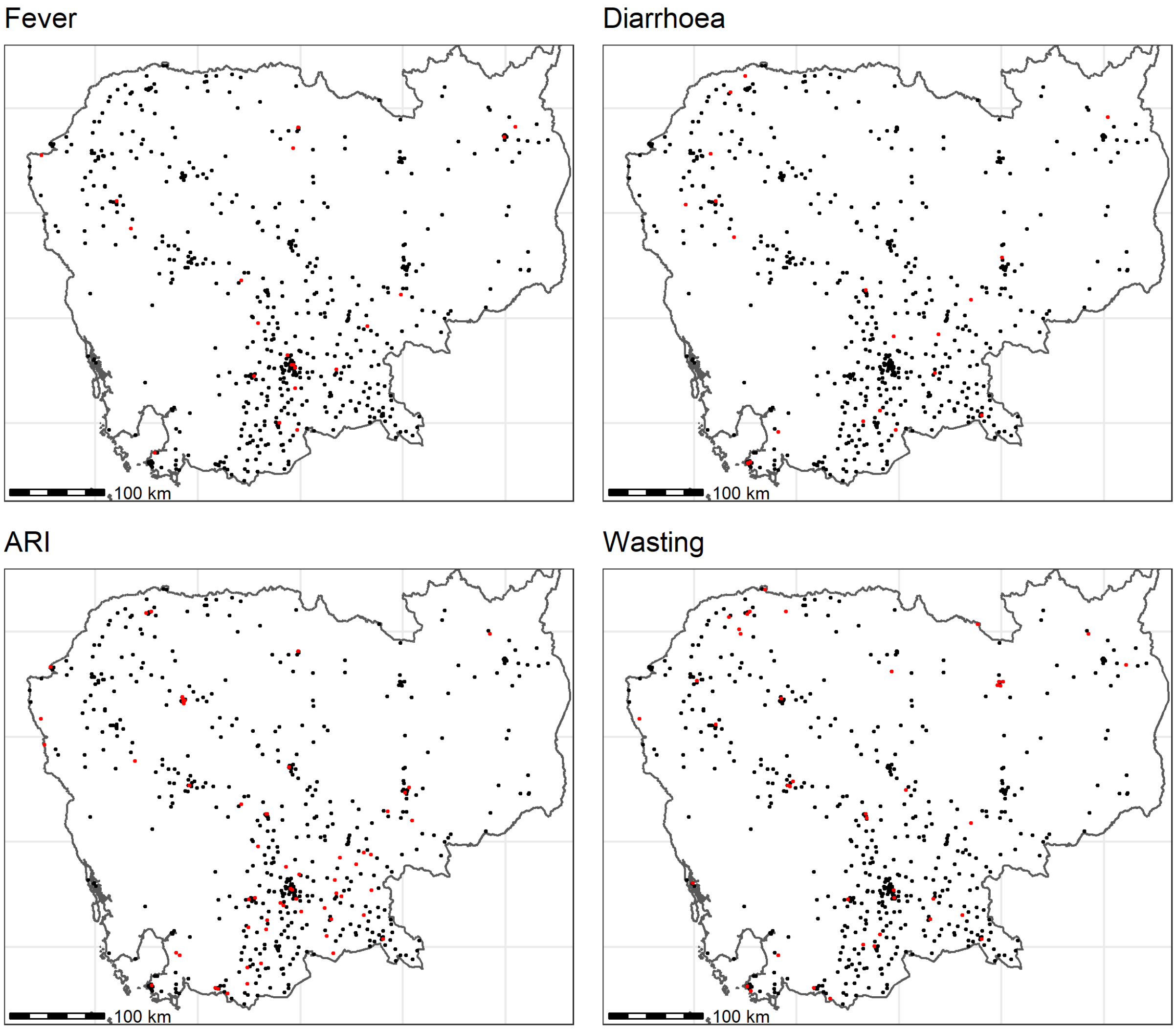

Cameroon

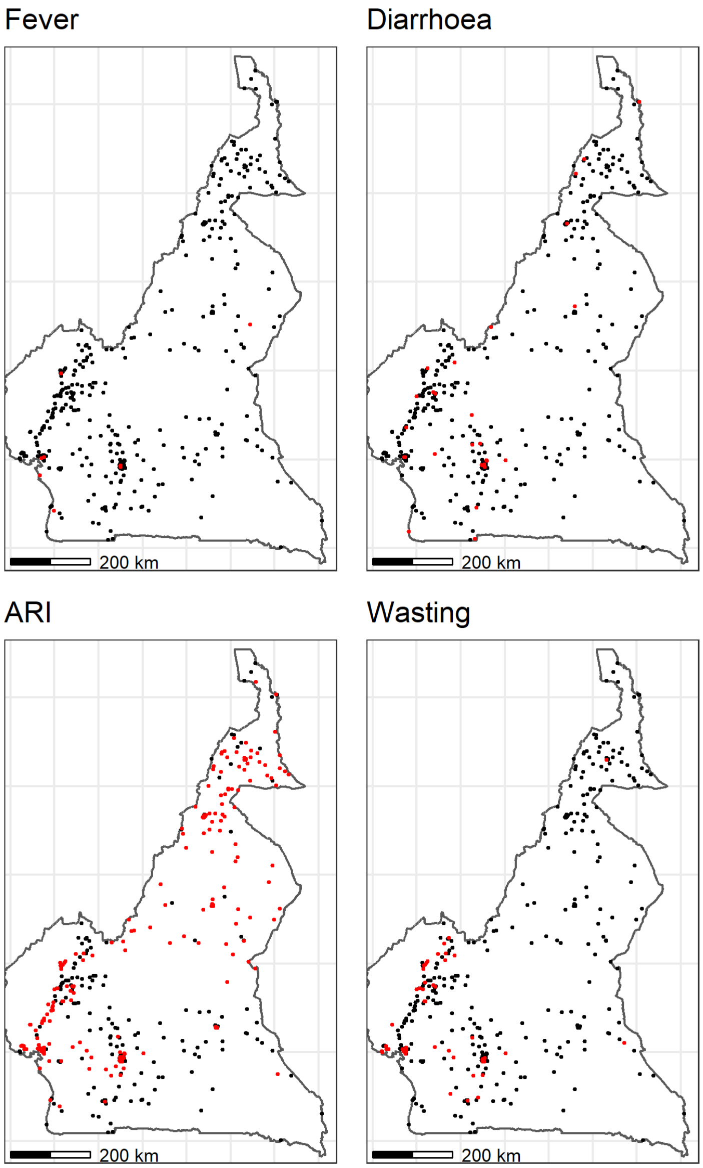

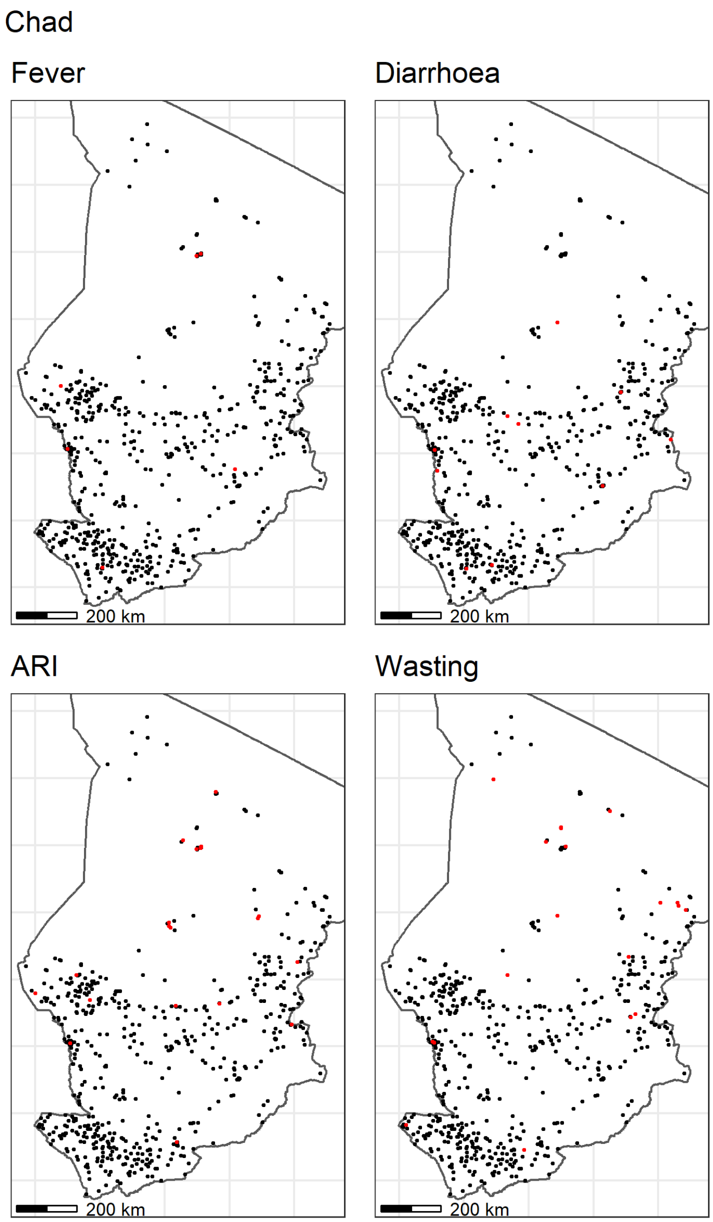
Chad
Fever
Diarrhoea
ARI
Wasting
200 km
200 km
200 km
200 km

Chandigarh

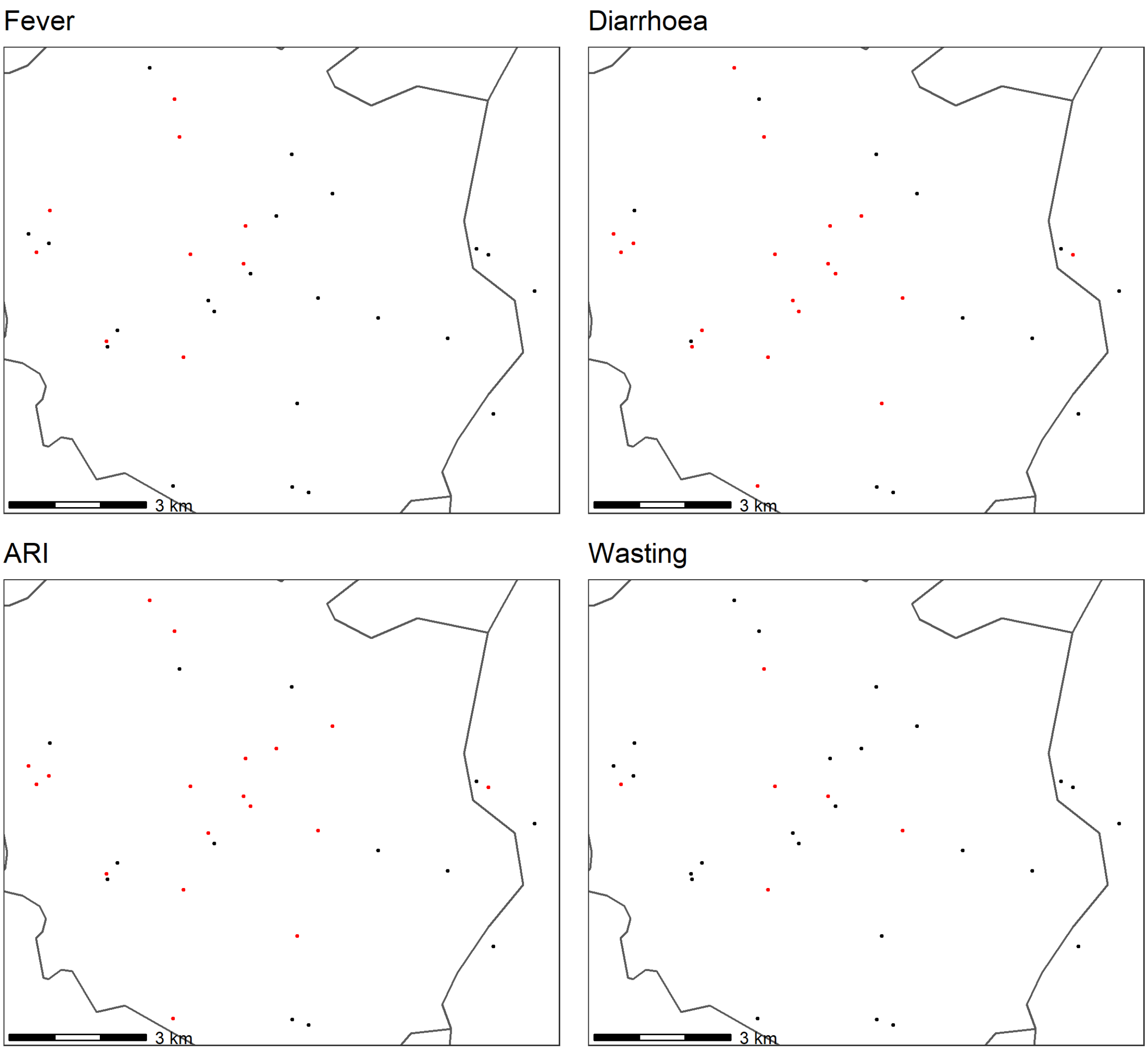

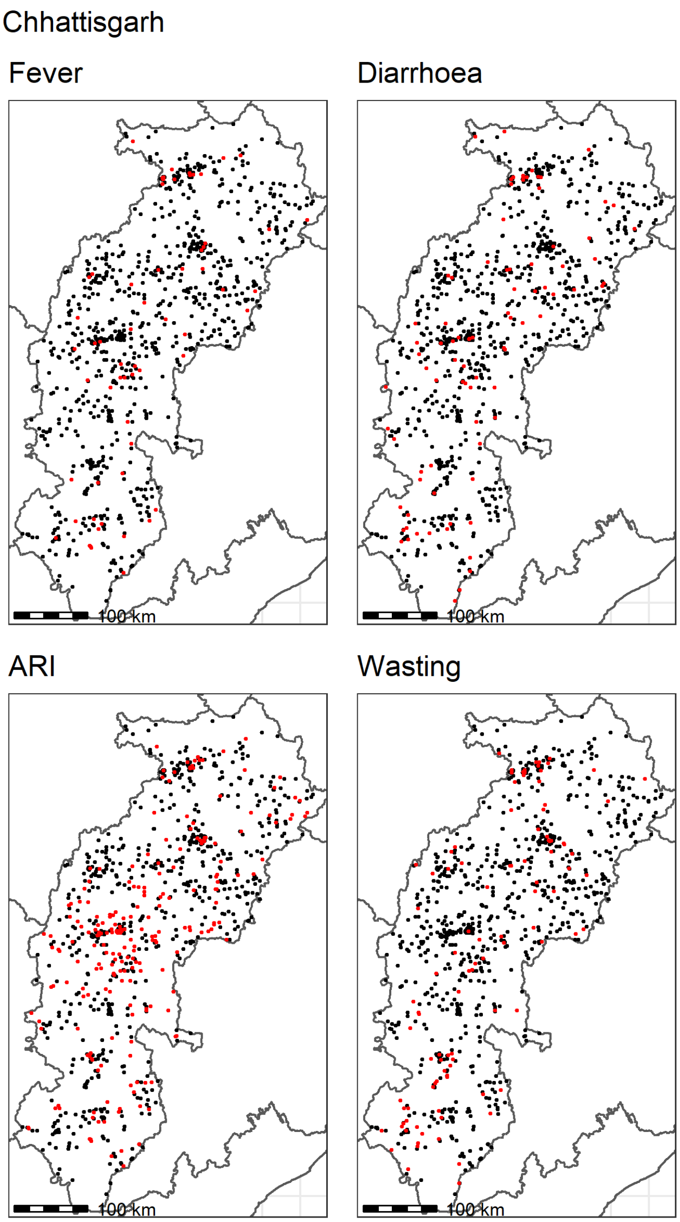
Chhattisgarh
Fever
Diarrhoea
ARI
Wasting
100 km
100 km
100 km
100 km

Comoros

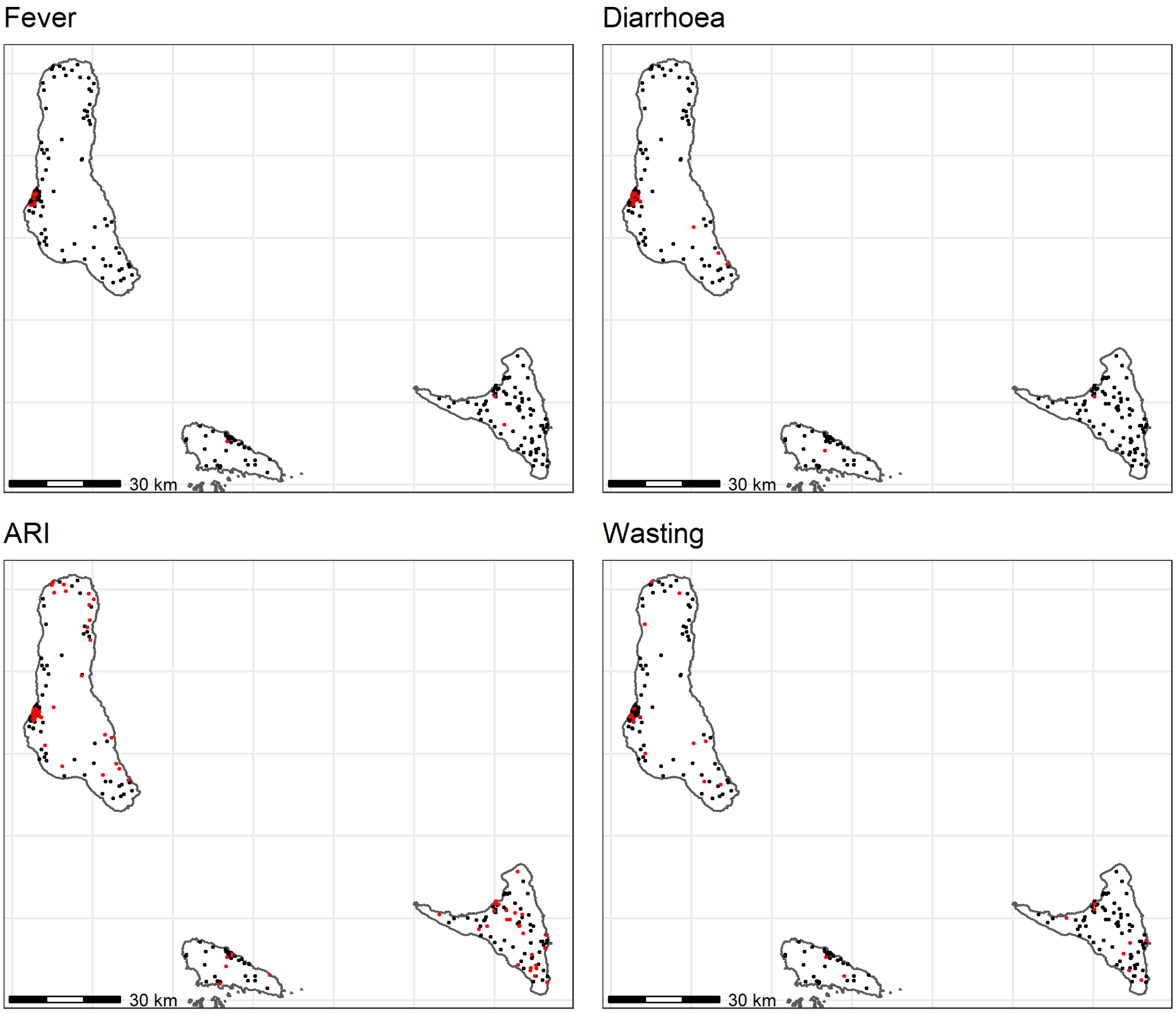

Congo Democratic Republic

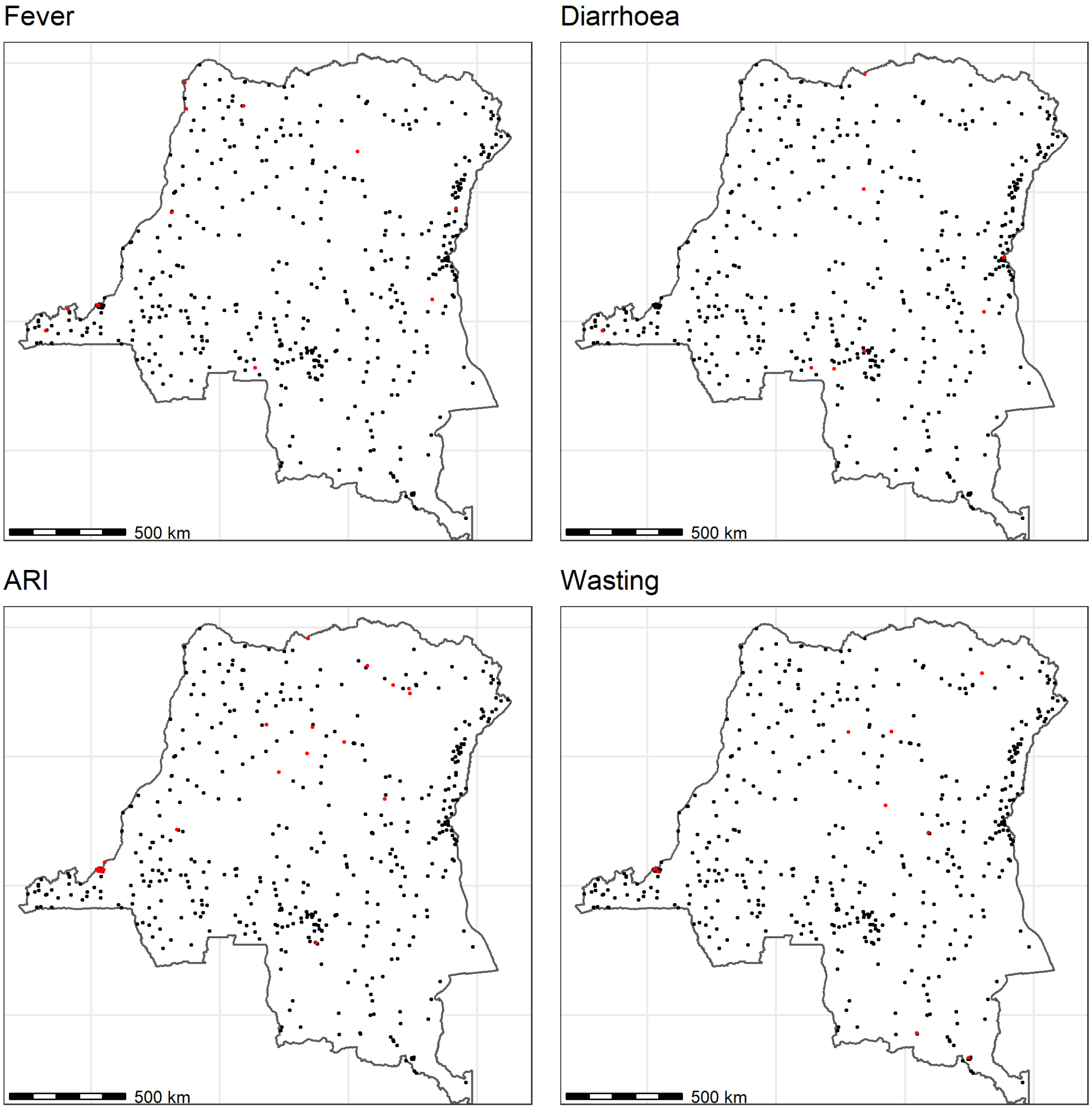

Cote d'Ivoire

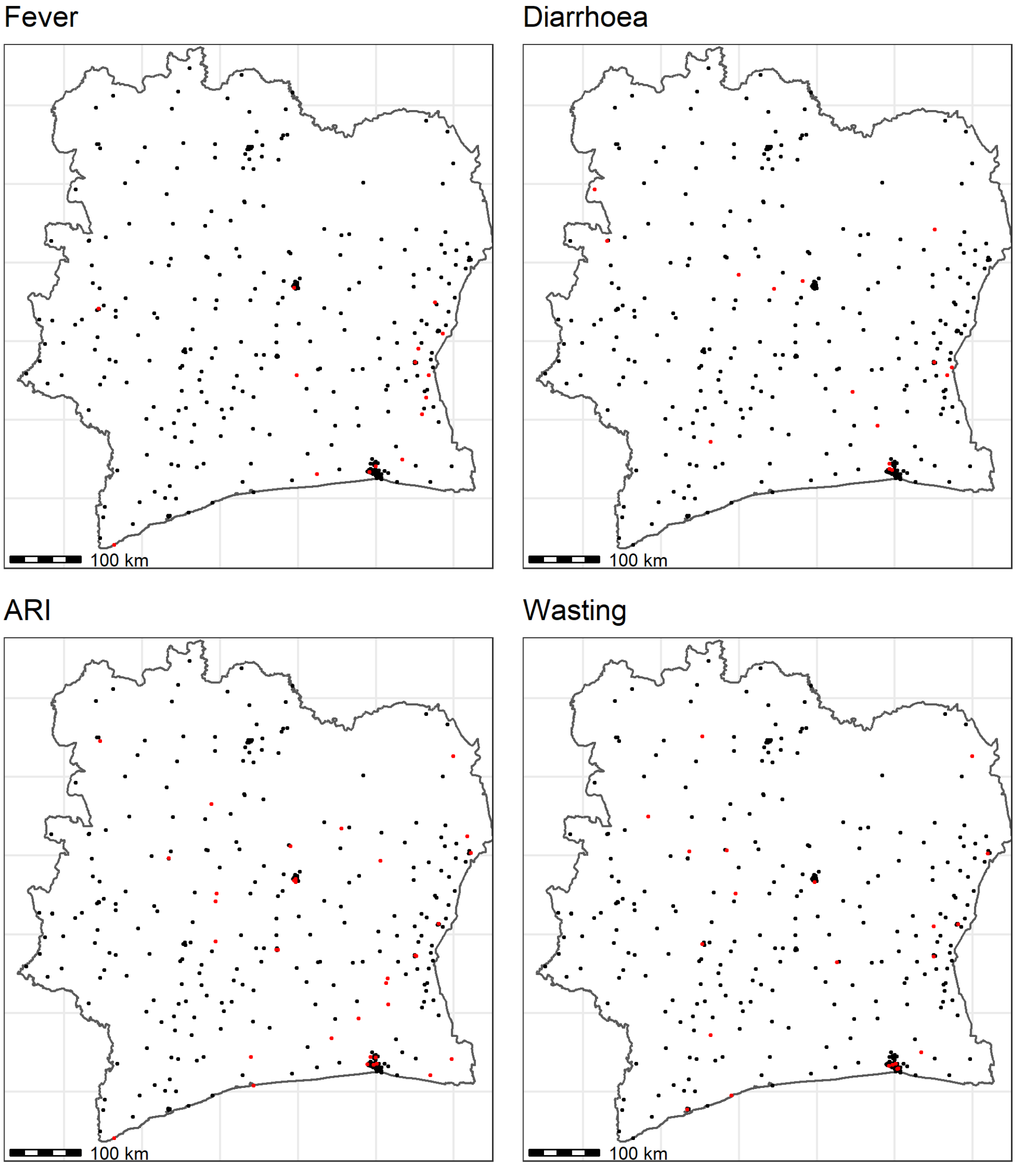

Dadra & Nagar Haveli

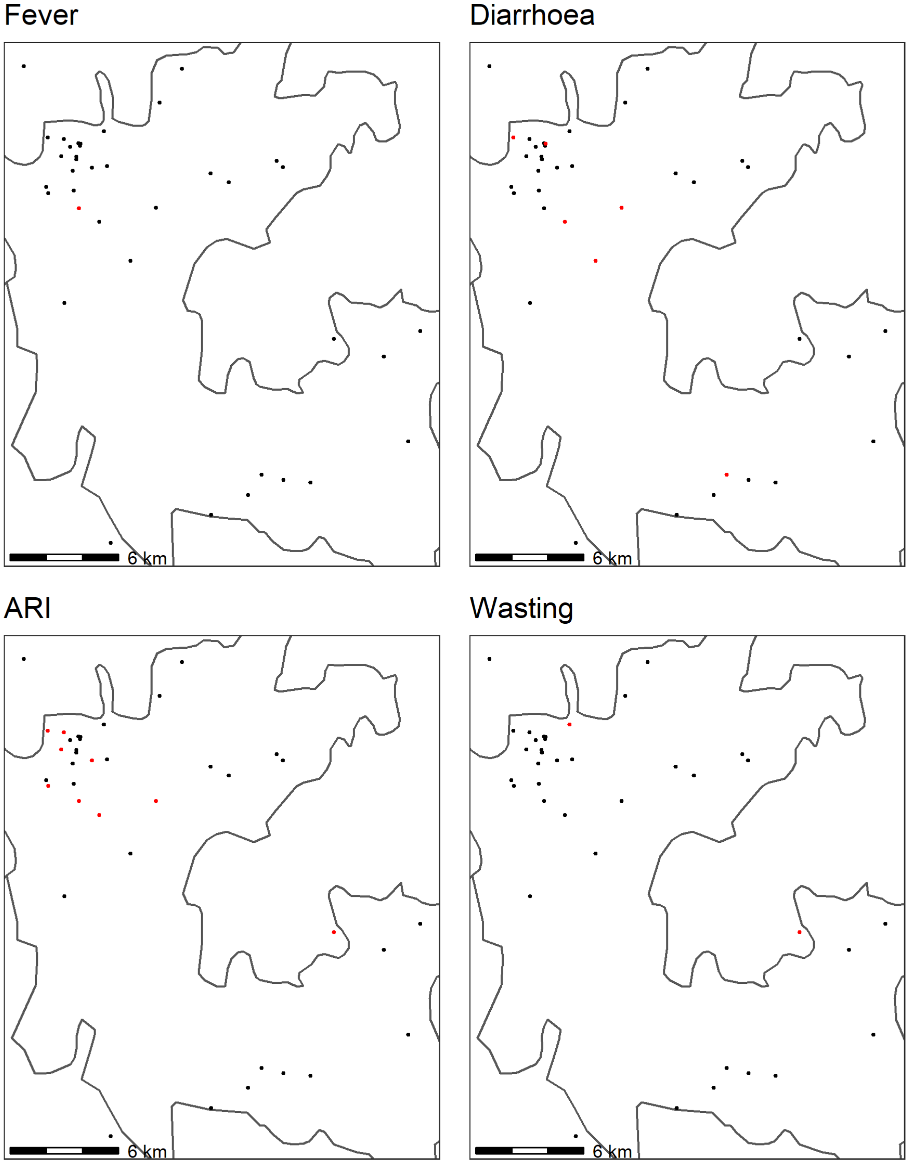

Andhra Pradesh

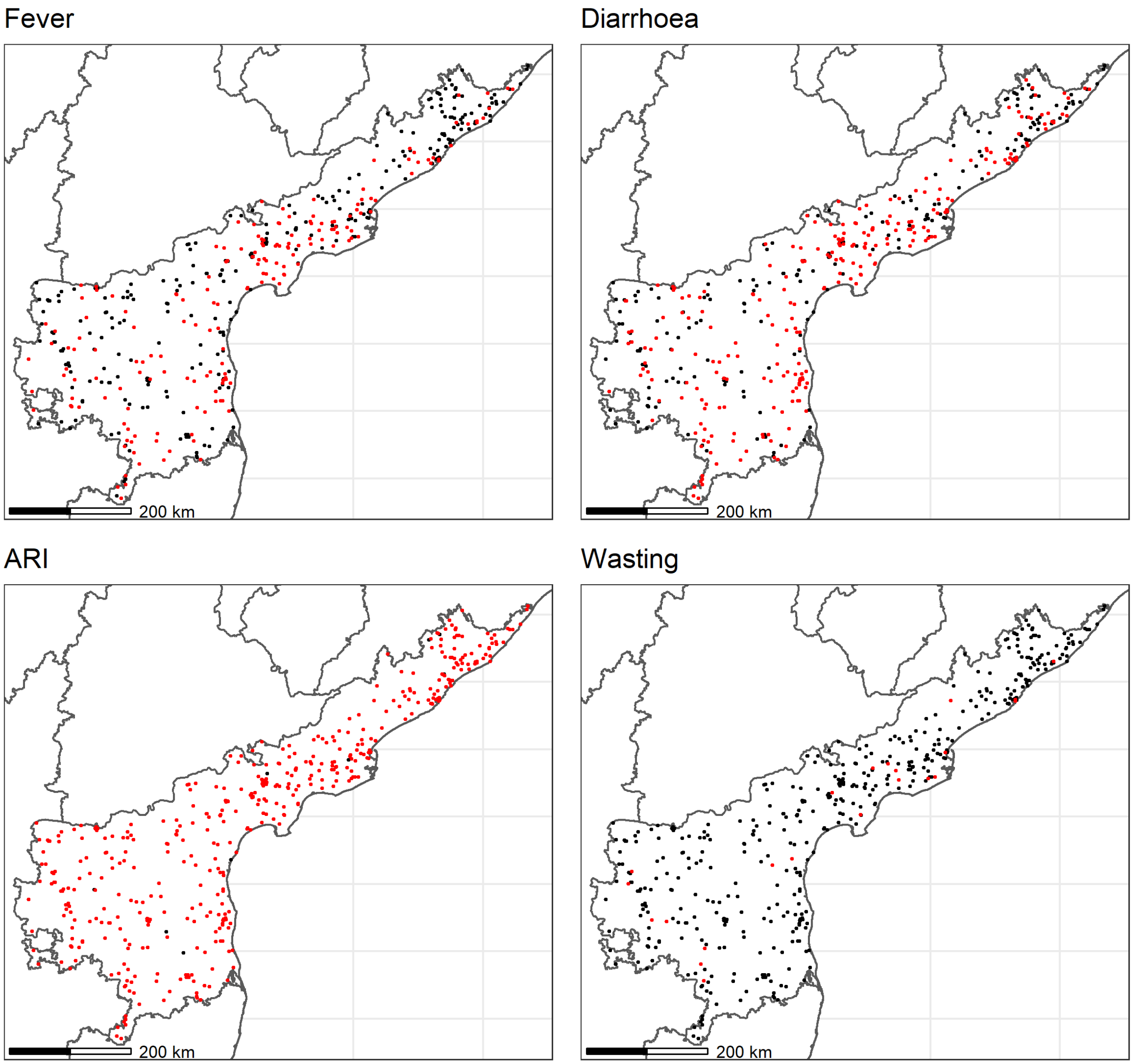

Daman & Diu

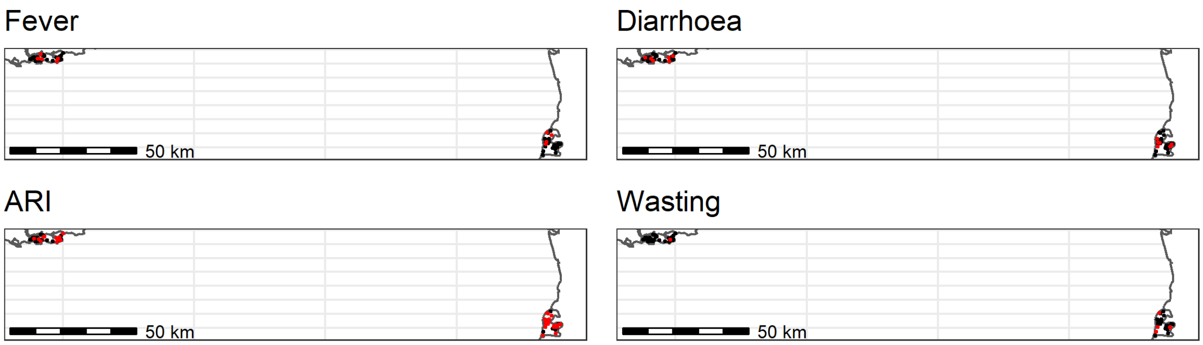

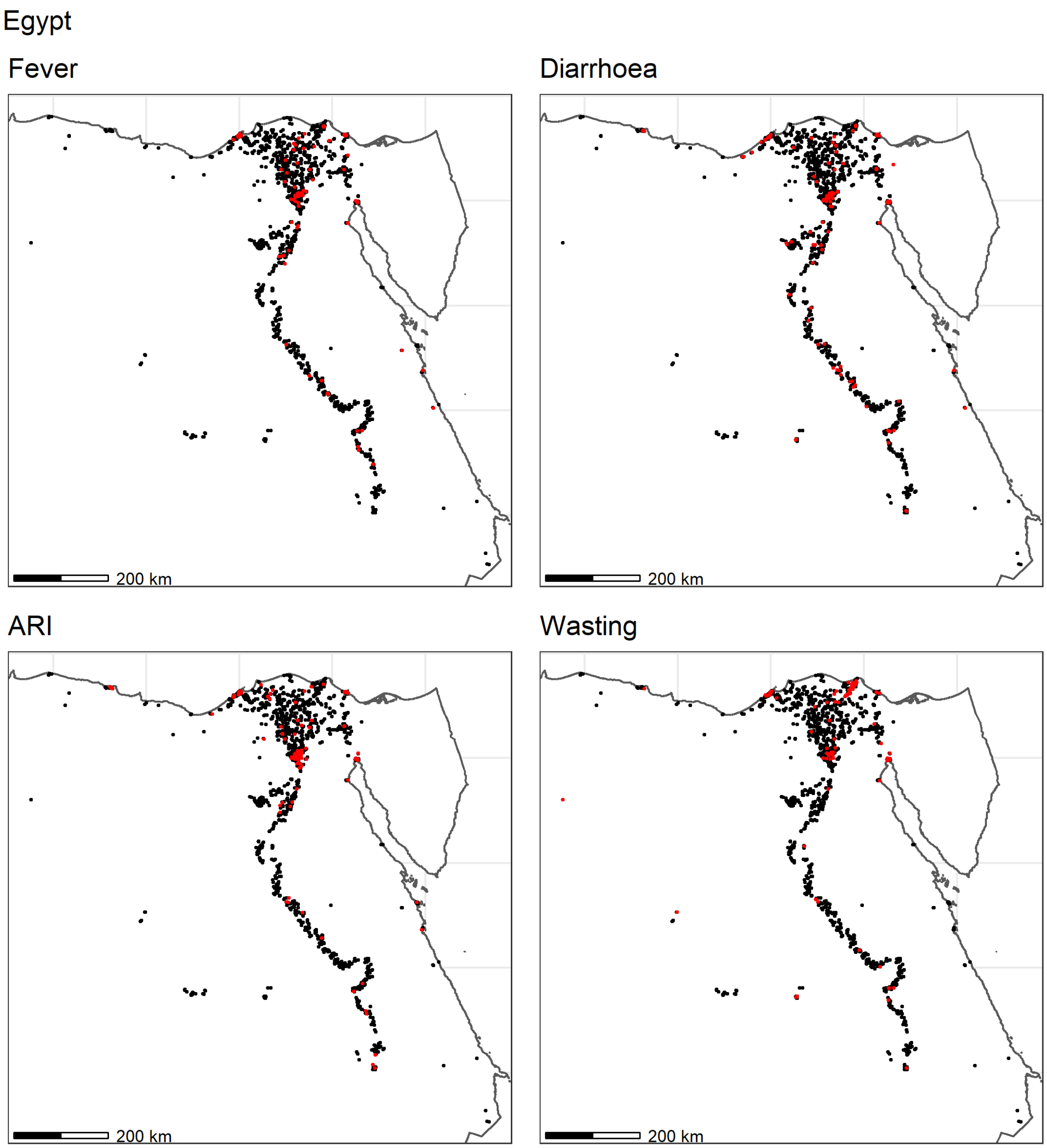
Egypt
Fever
200 km
Diarrhoea
200 km
ARI
200 km
Wasting
200 km

Ethiopia

Fever

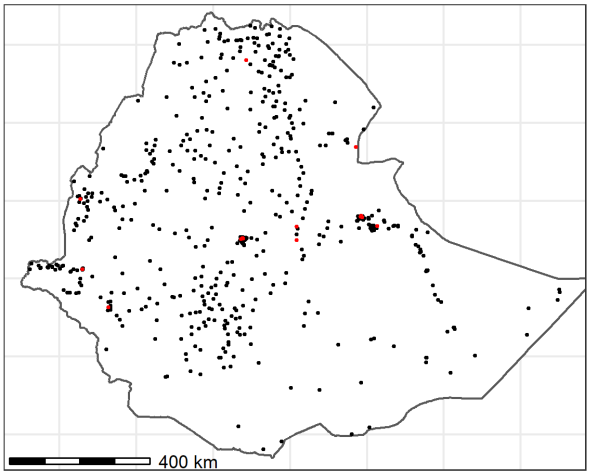

Diarrhoea

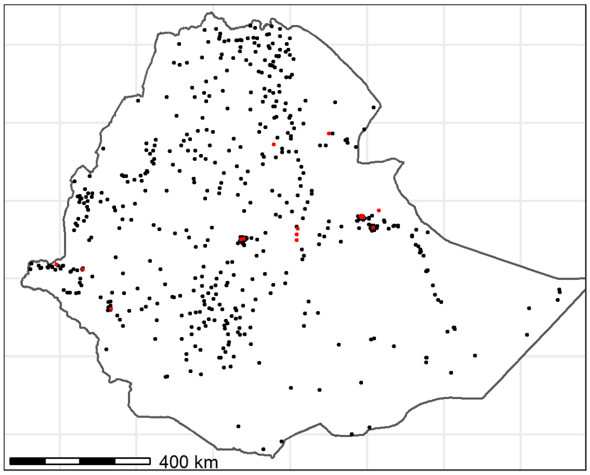

ARI

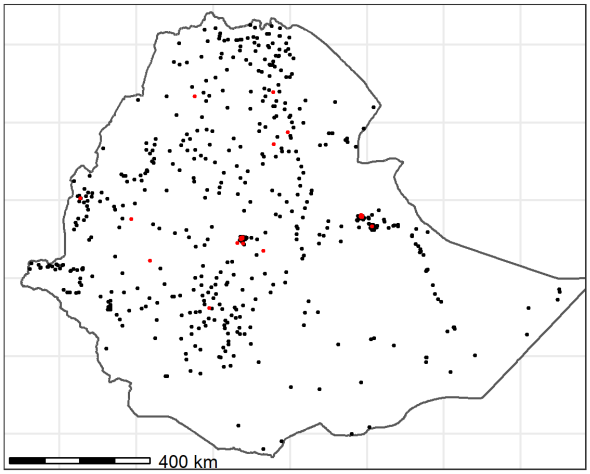

Wasting

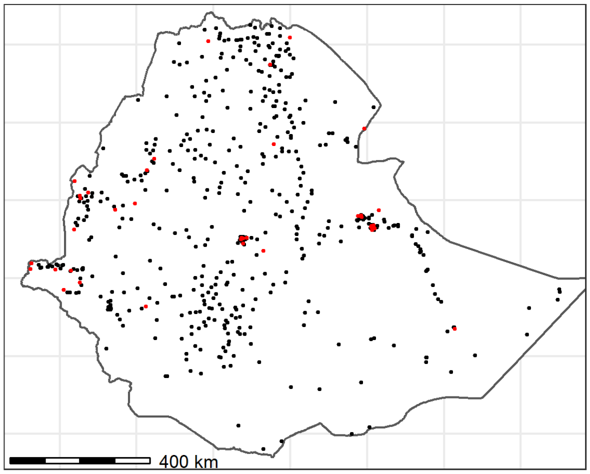

Gabon

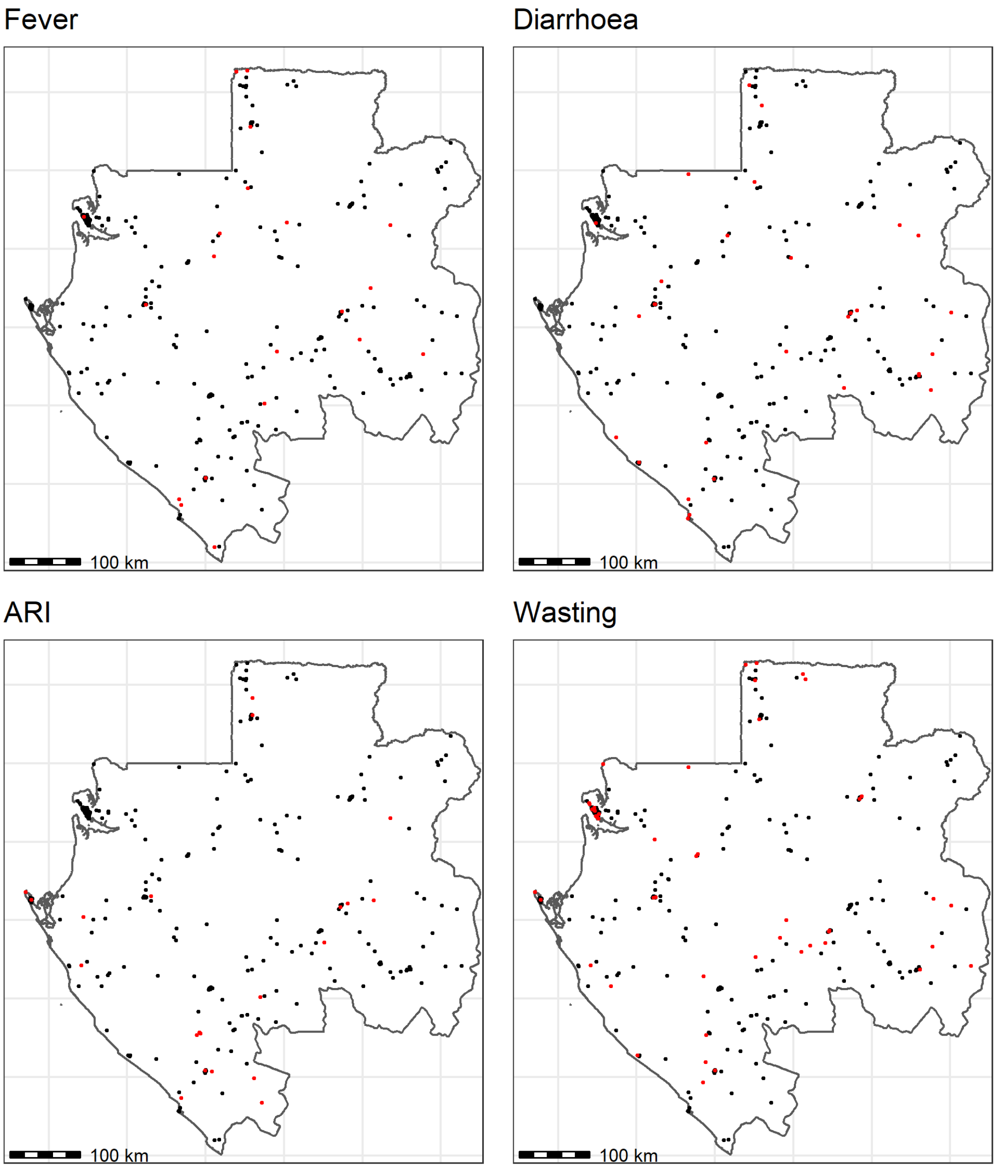

Ghana

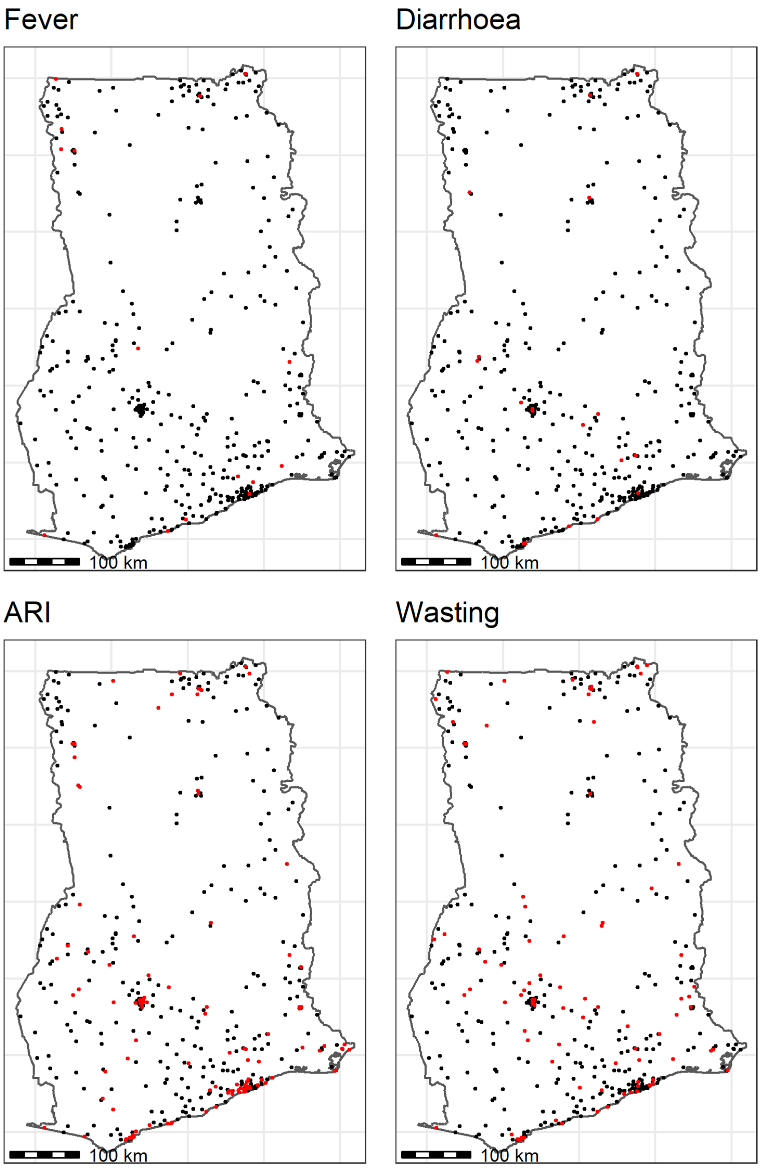

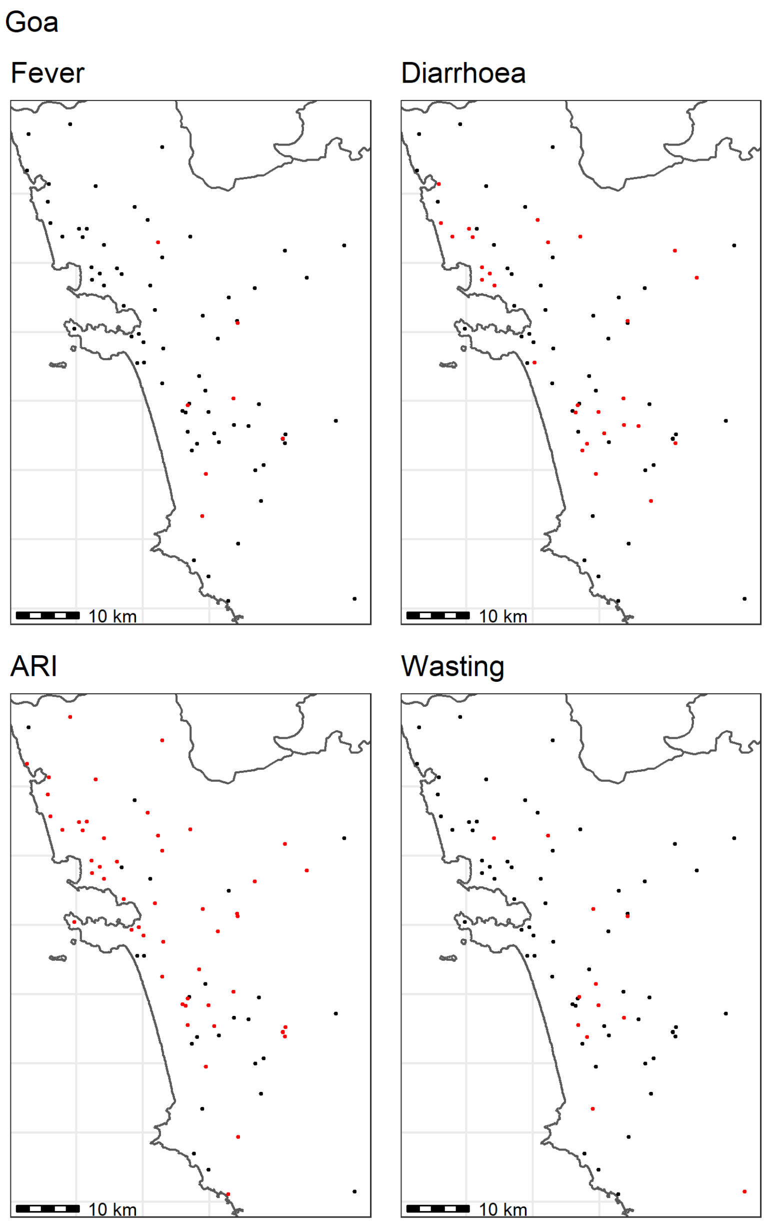
Goa
Fever
Diarrhoea
ARI
Wasting
10 km
10 km
10 km
10 km

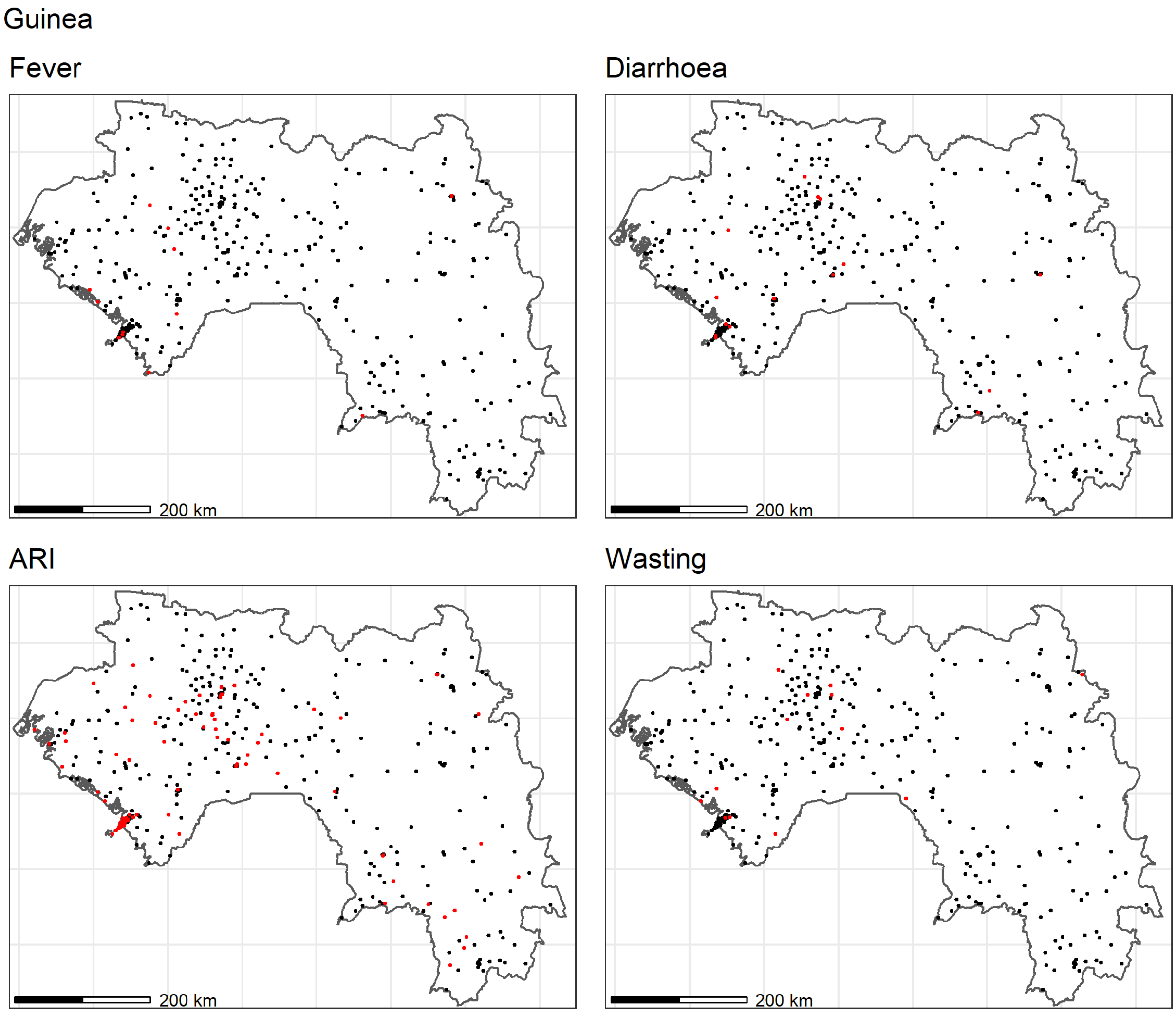
Guinea
Fever
Diarrhoea
ARI
Wasting
200 km
200 km
200 km
200 km

Gujarat

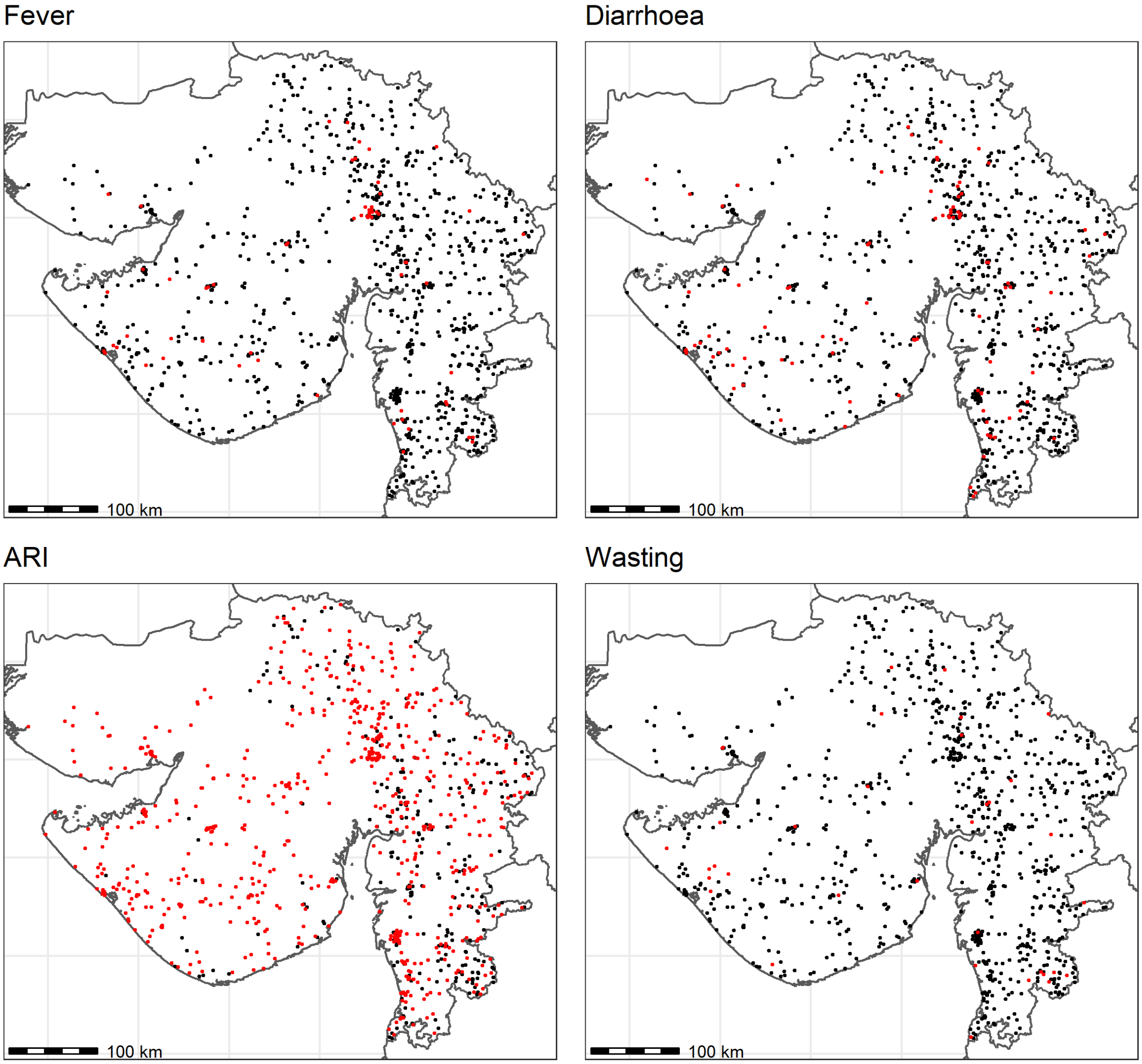

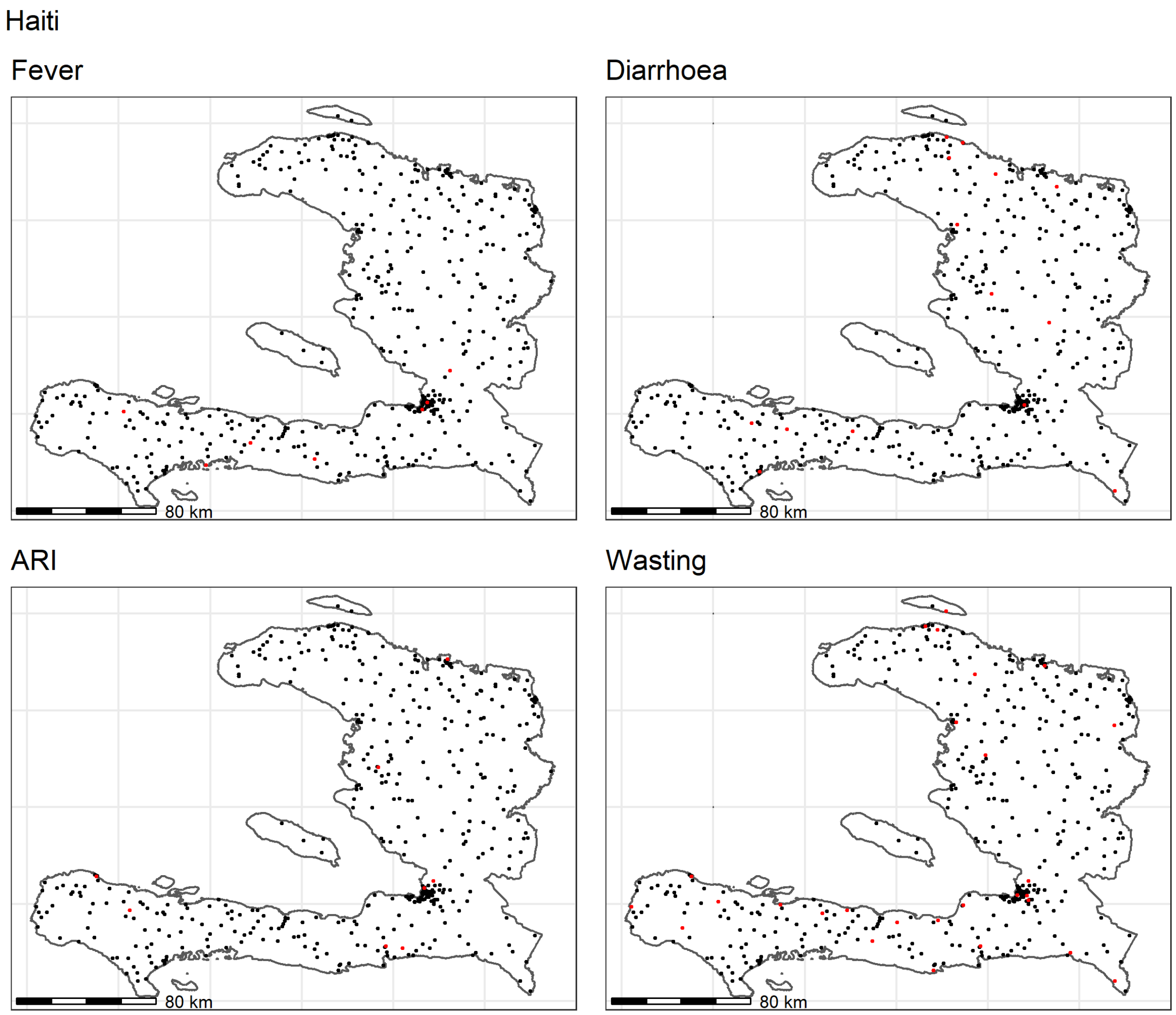
Haiti
Fever
Diarrhoea
ARI
Wasting
80 km
80 km
80 km
80 km

Haryana

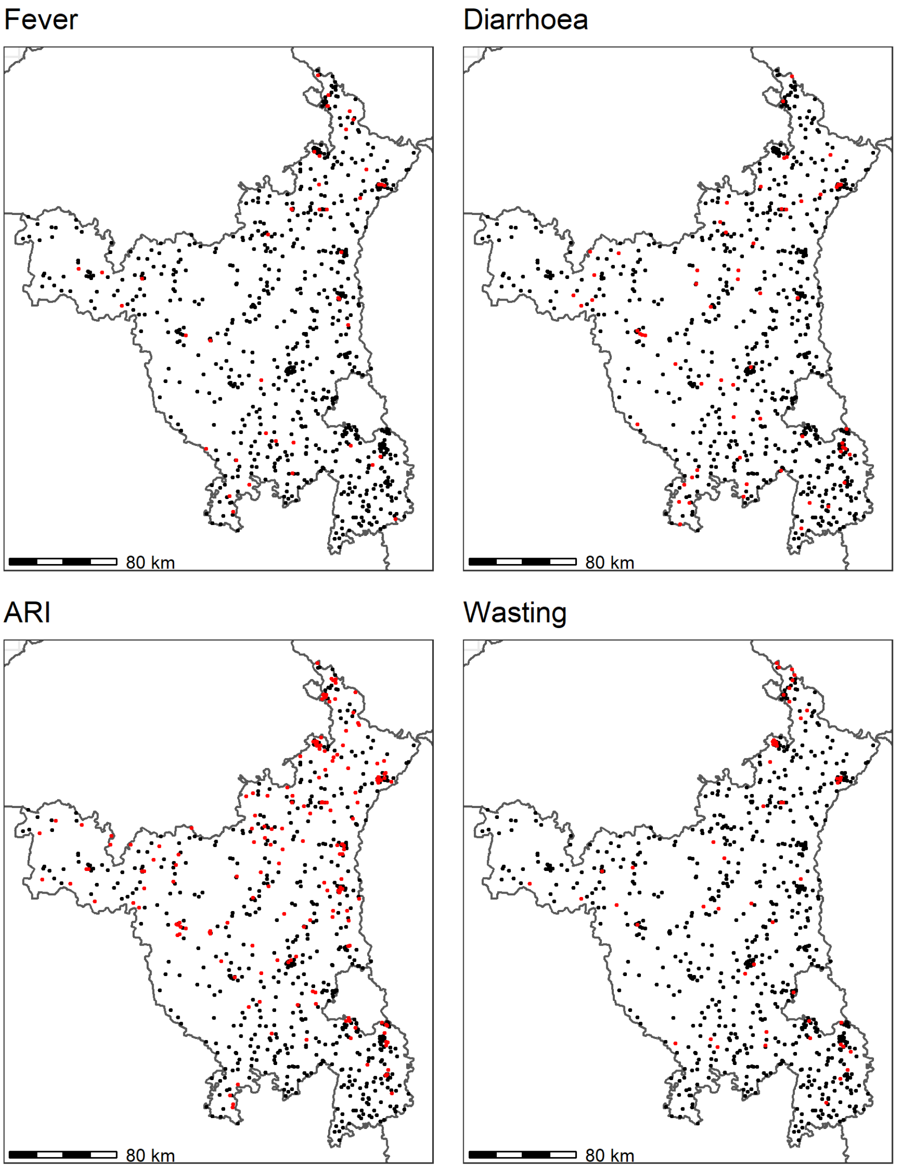

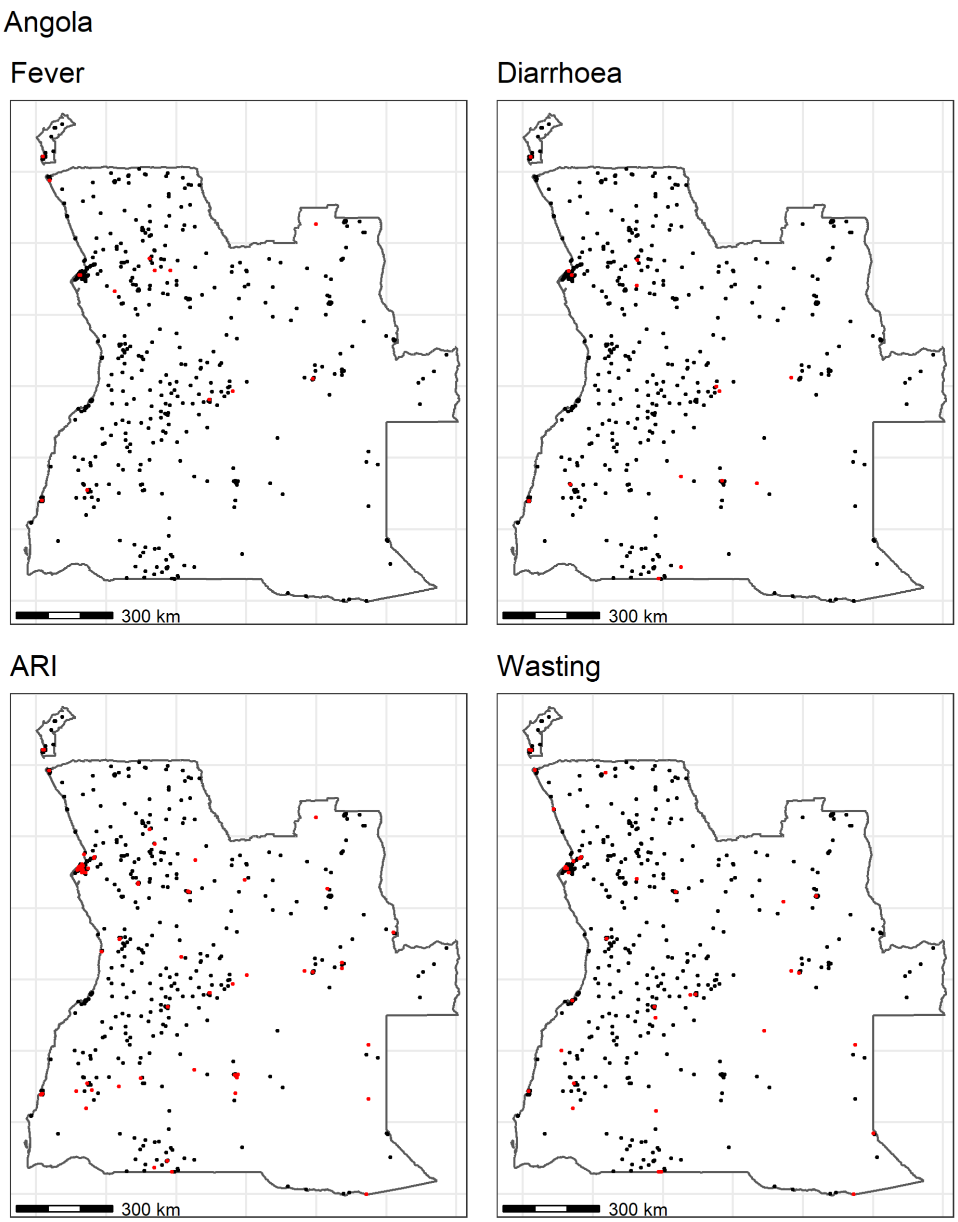
Angola
Fever
300 km
Diarrhoea
300 km
ARI
300 km
Wasting
300 km

Himachal Pradesh

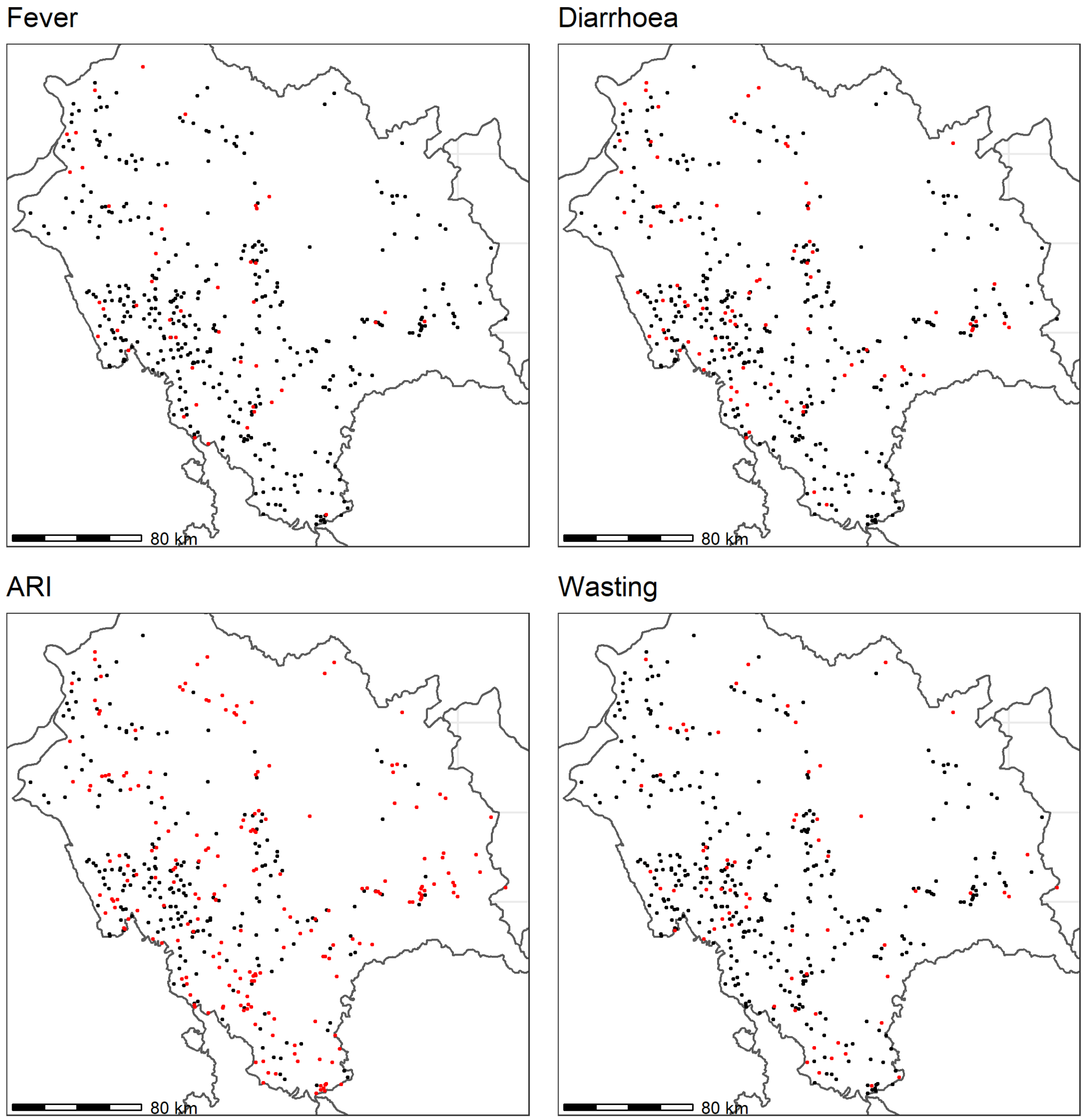

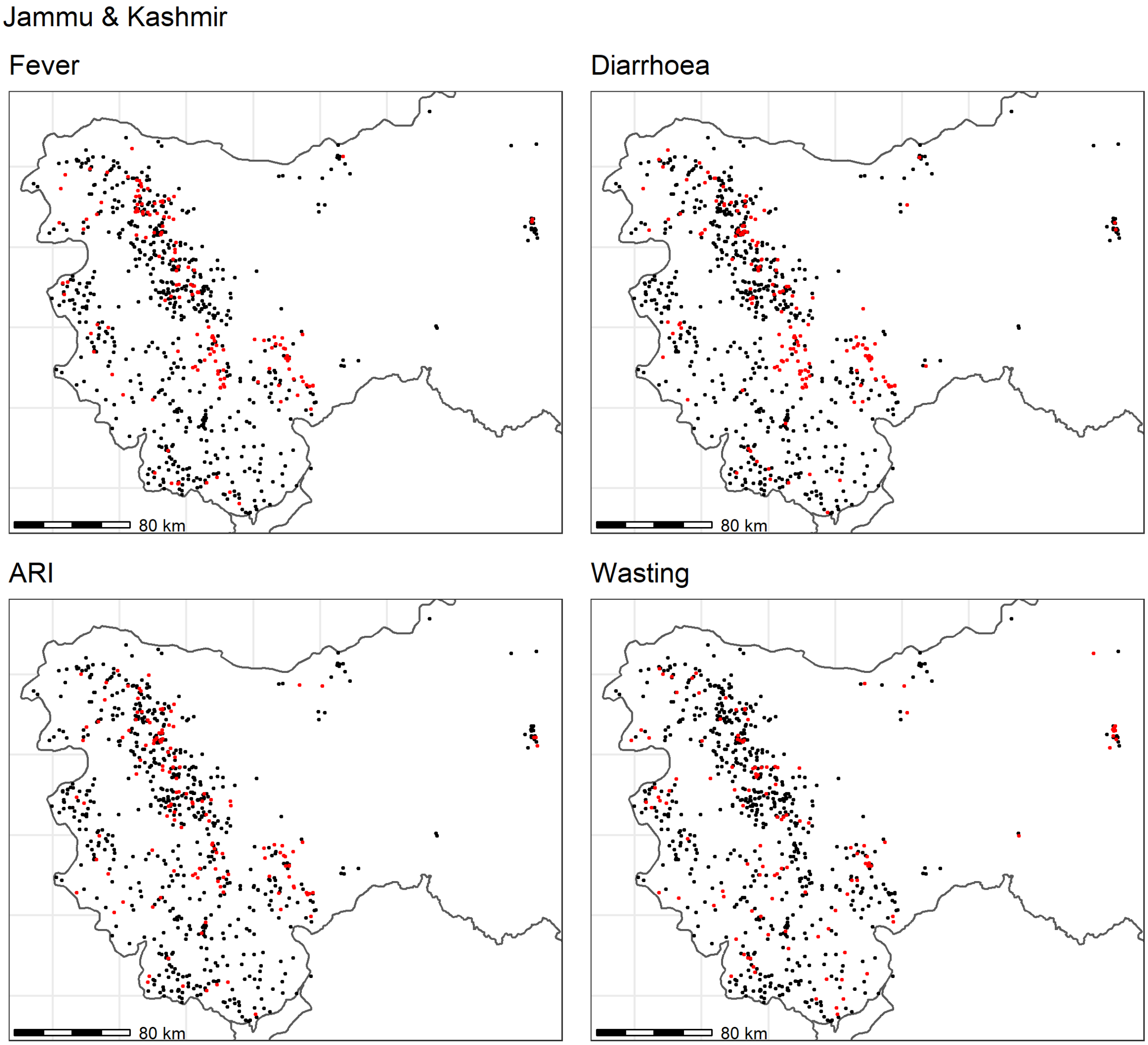
Jammu & Kashmir
Fever
80 km
Diarrhoea
80 km
ARI
80 km
Wasting
80 km

Jharkhand

Fever

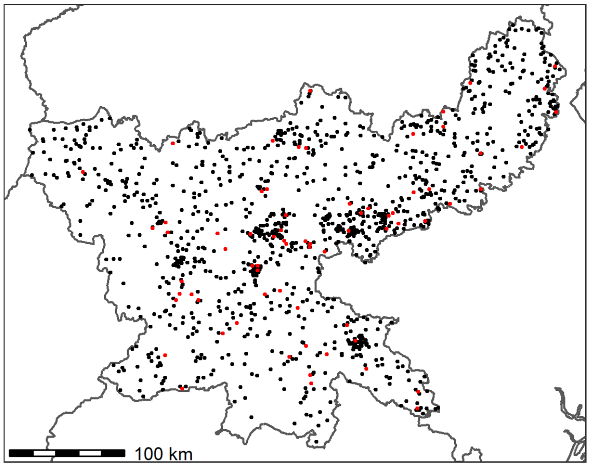

Diarrhoea

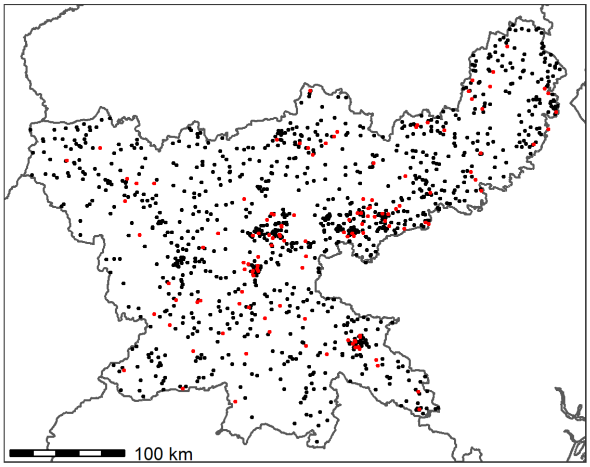

ARI

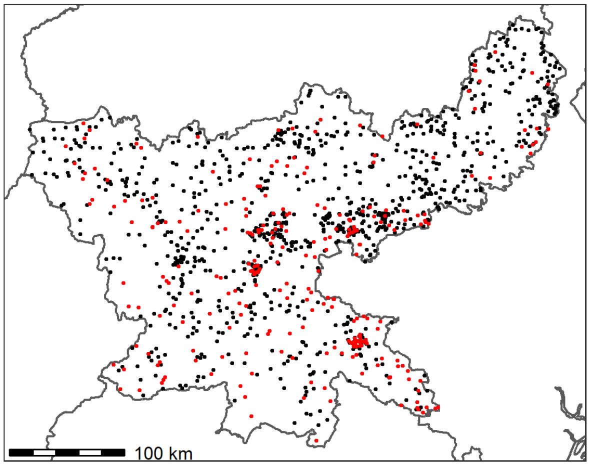

Wasting

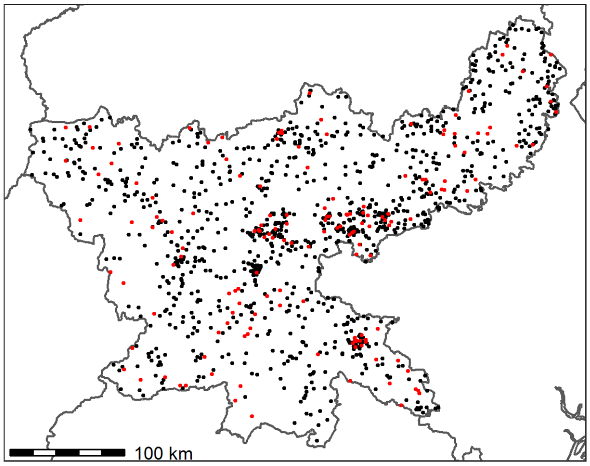

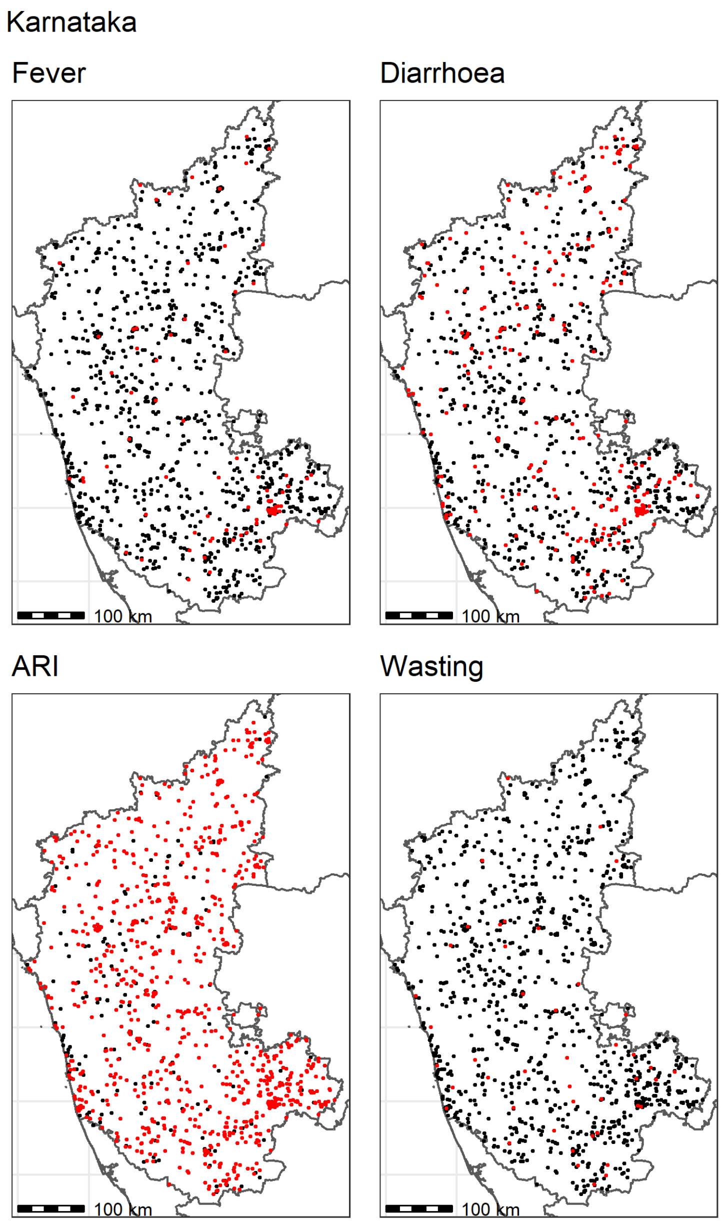
Karnataka
Fever
Diarrhoea
ARI
Wasting
100 km
100 km
100 km
100 km

Kenya

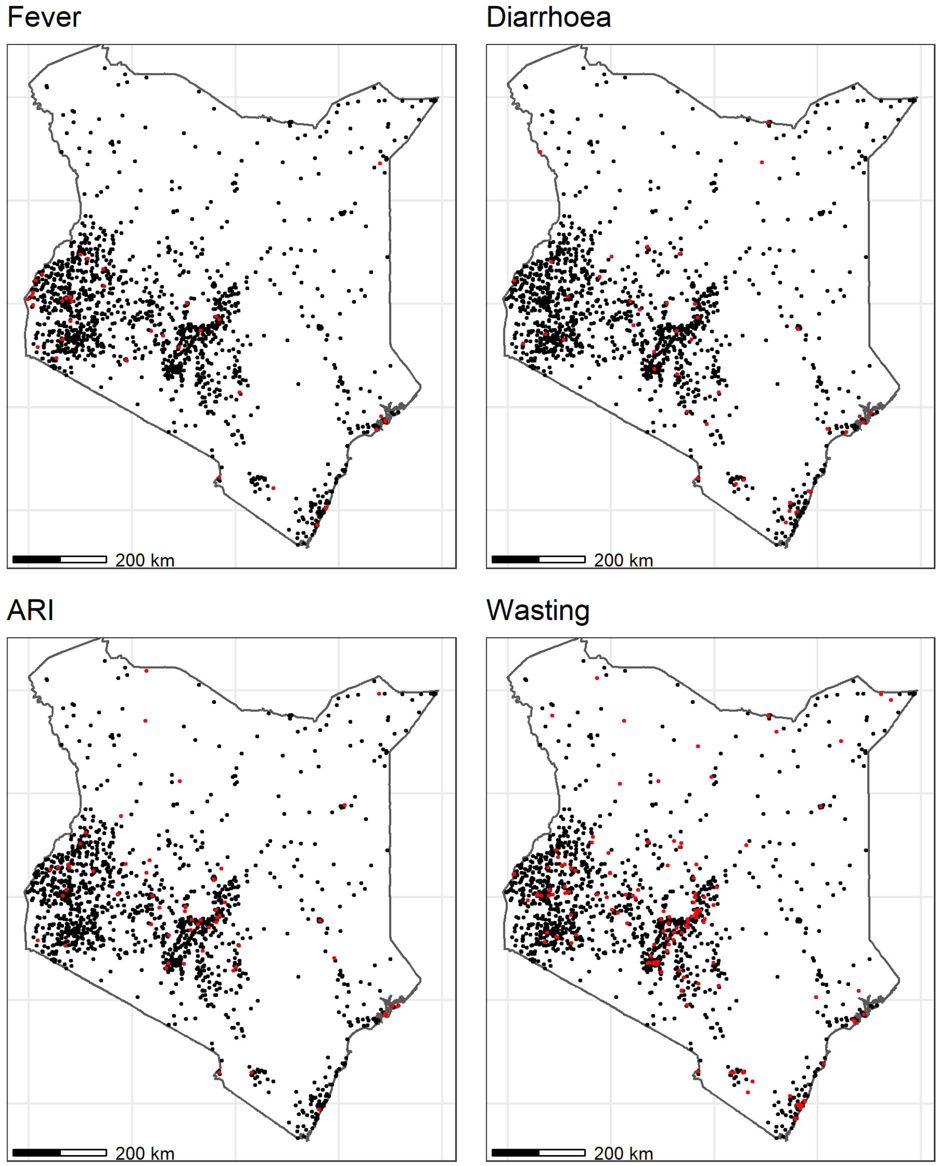

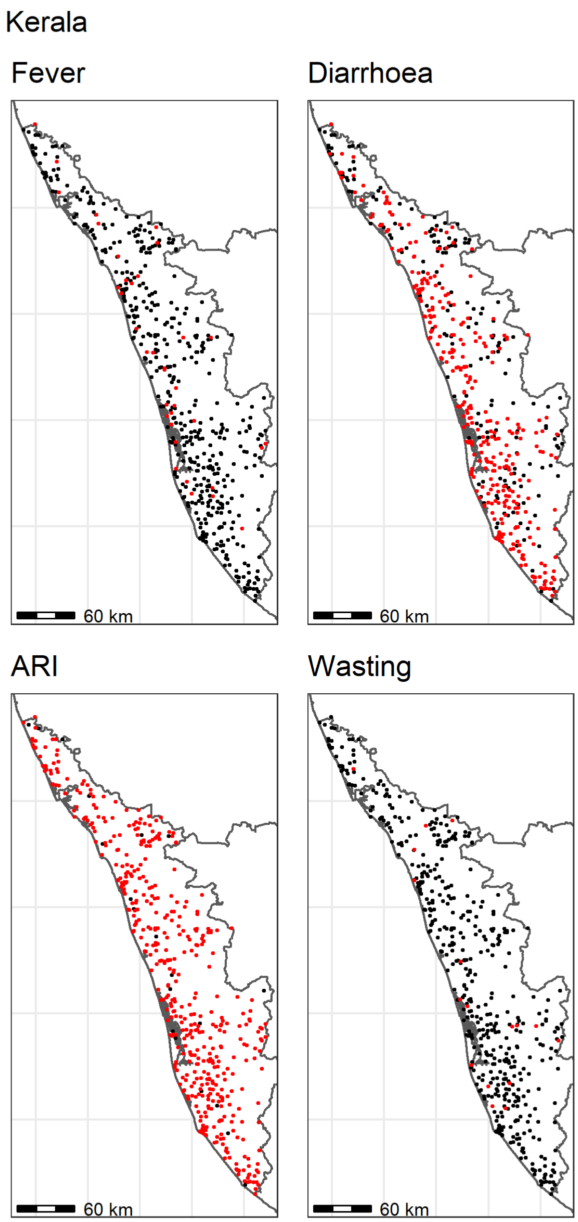
Kerala
Fever
Diarrhoea
60 km
60 km
ARI
Wasting
60 km
60 km

Lakshadweep

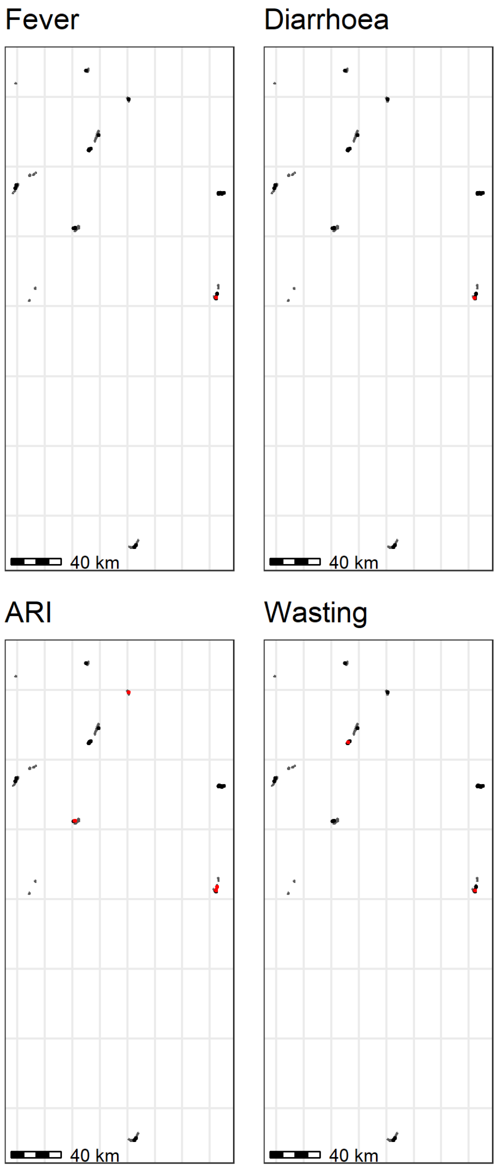

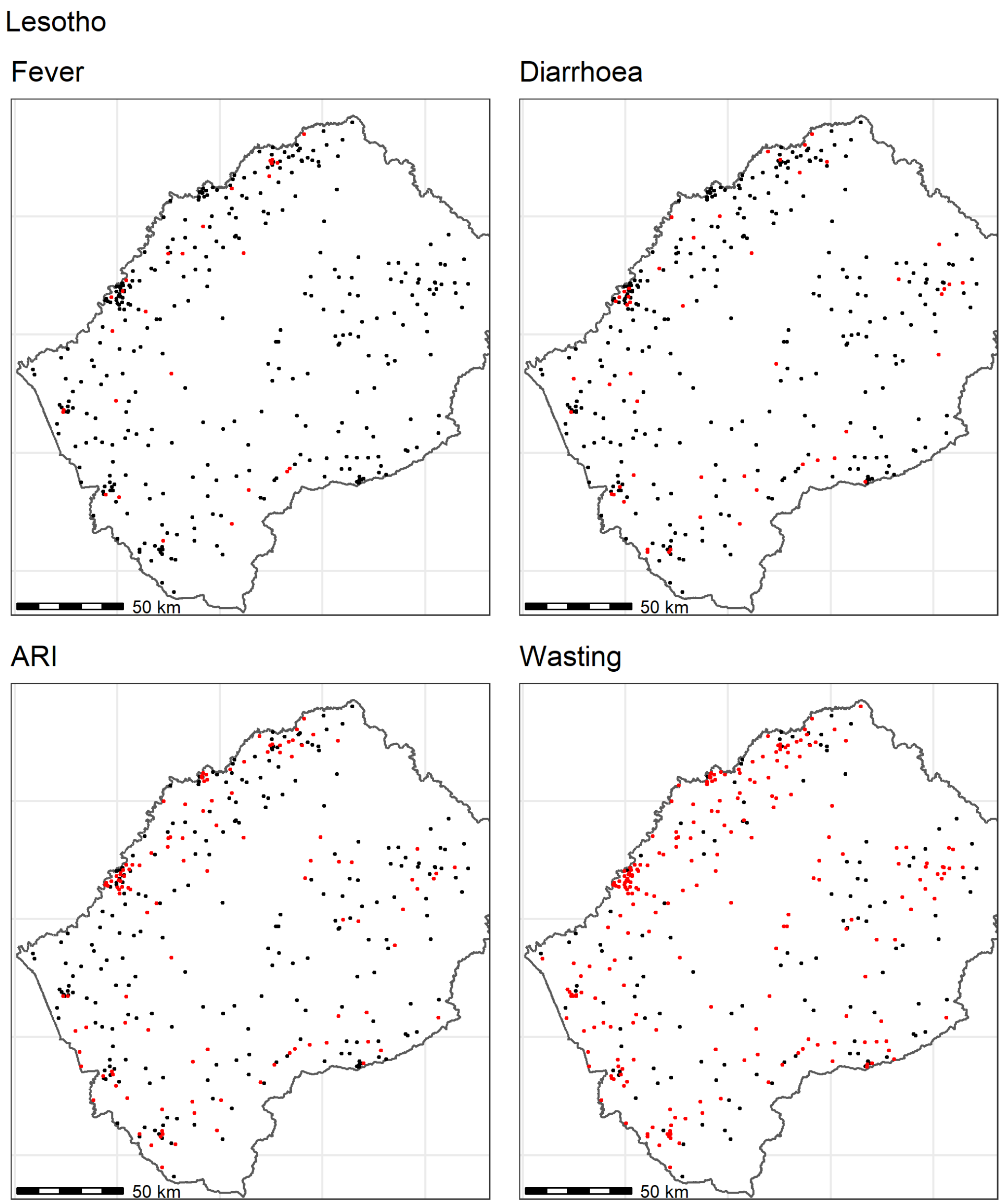

Lesotho
Fever
Diarrhoea
ARI
Wasting
50 km
50 km
50 km
50 km

Liberia

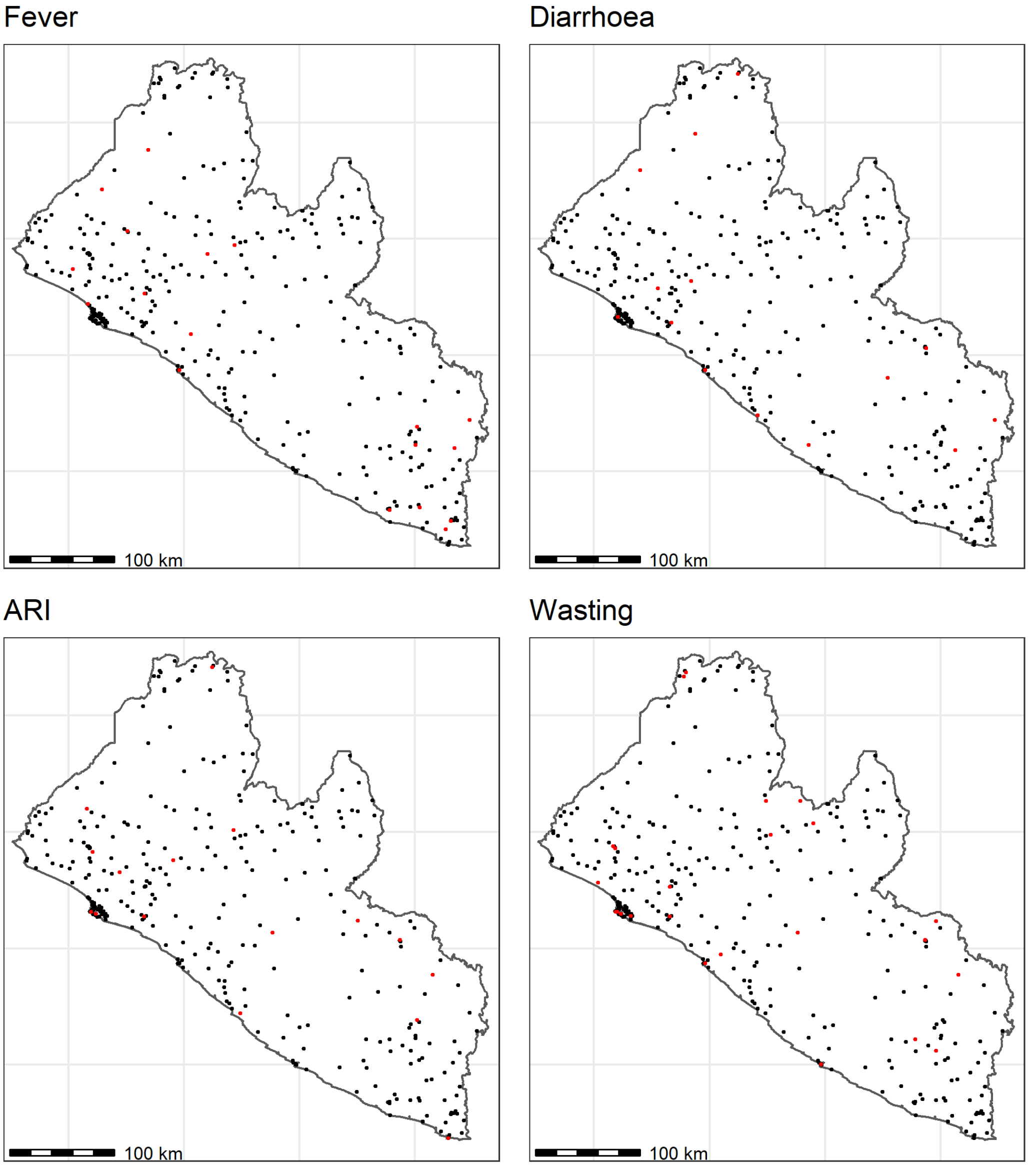

Madhya Pradesh

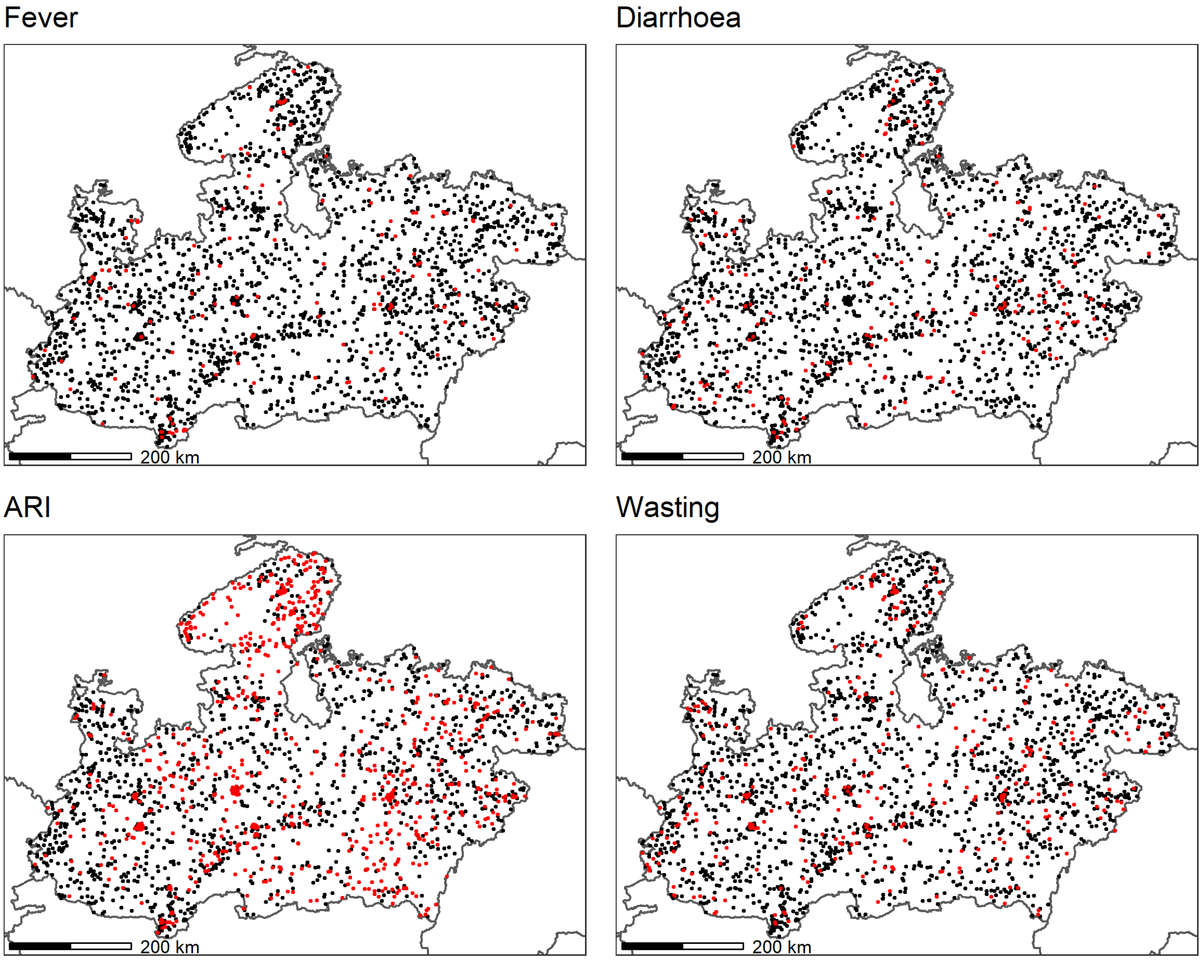

Arunachal Pradesh

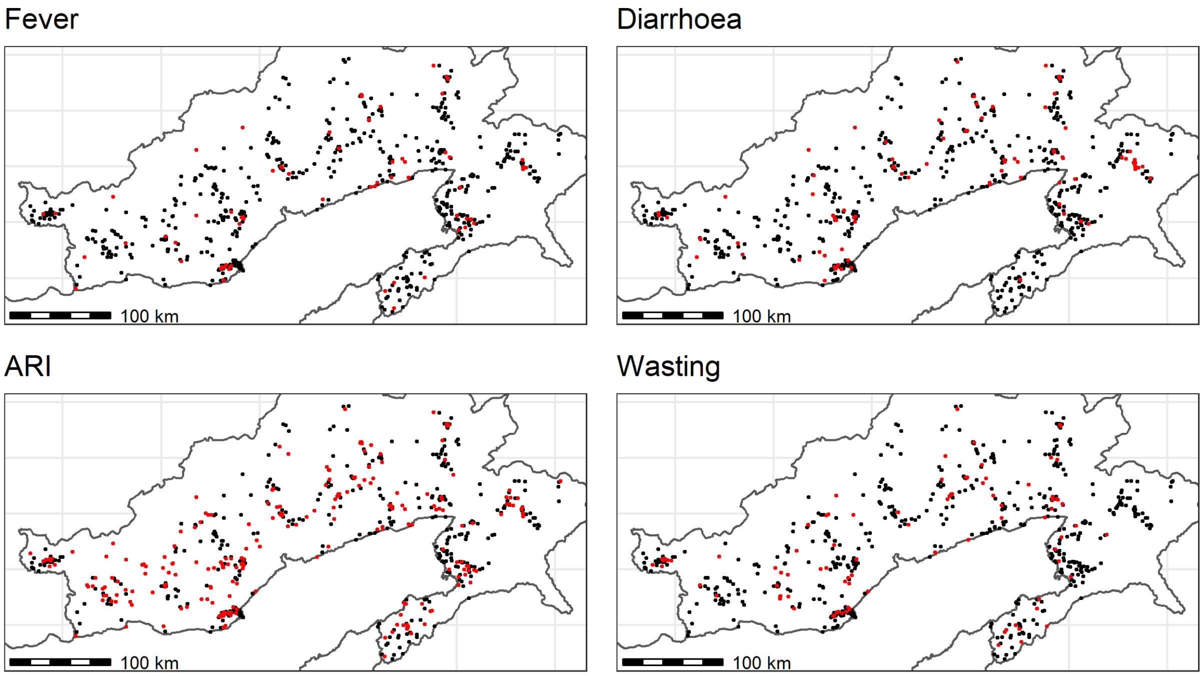

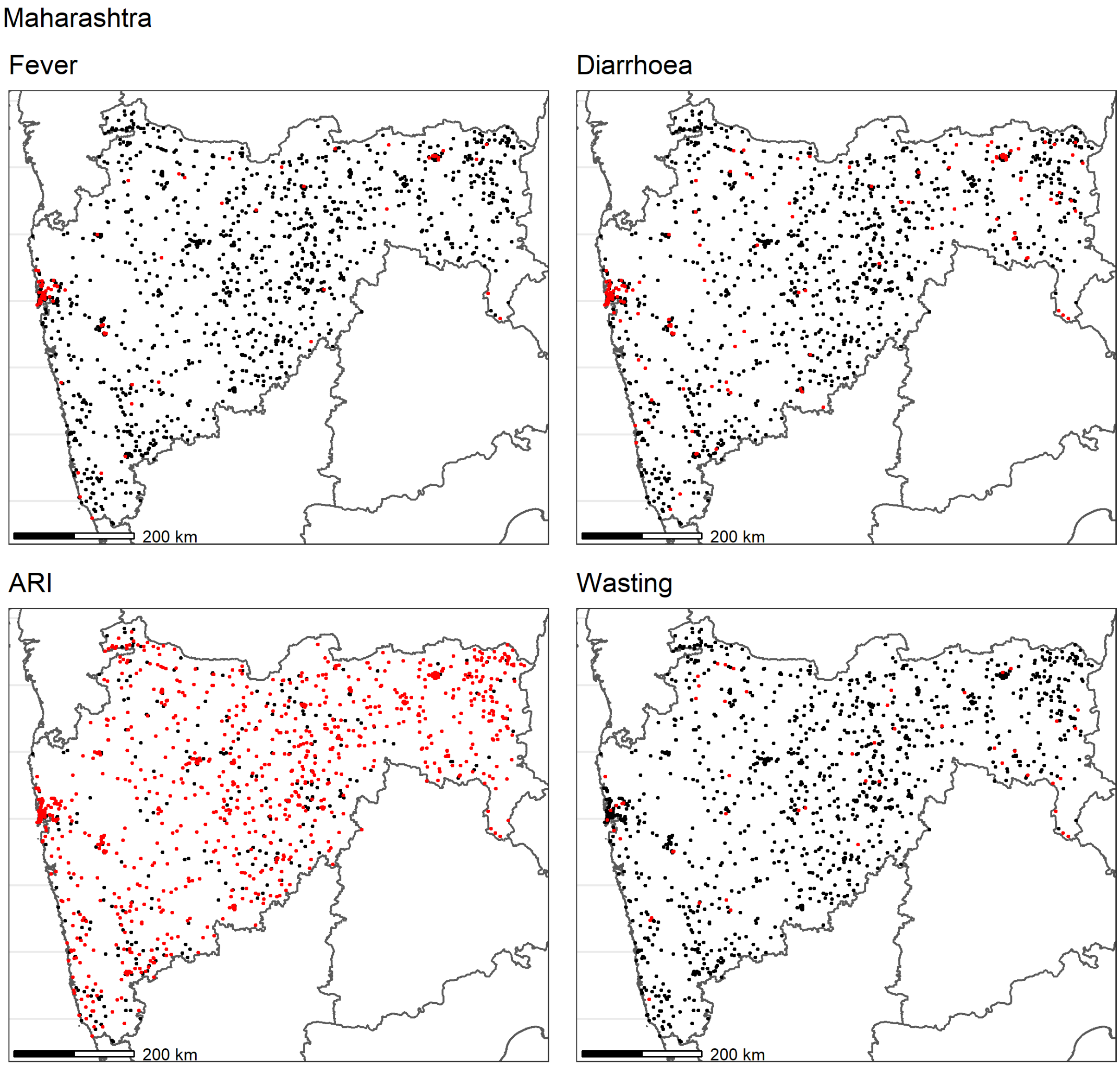
Maharashtra
Fever
Diarrhoea
ARI
Wasting
200 km
200 km
200 km
200 km

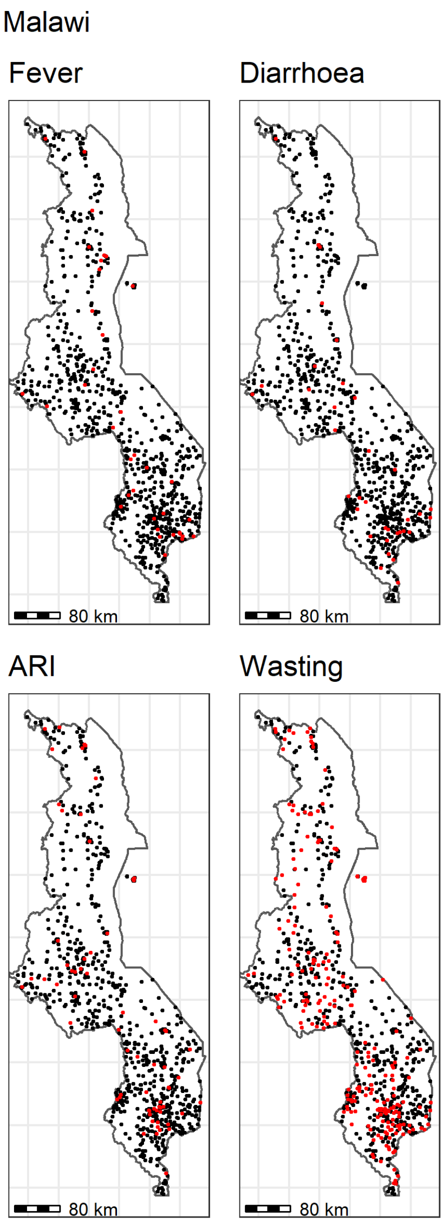
Malawi
Fever
Diarrhoea
ARI
Wasting
80 km
80 km
80 km
80 km

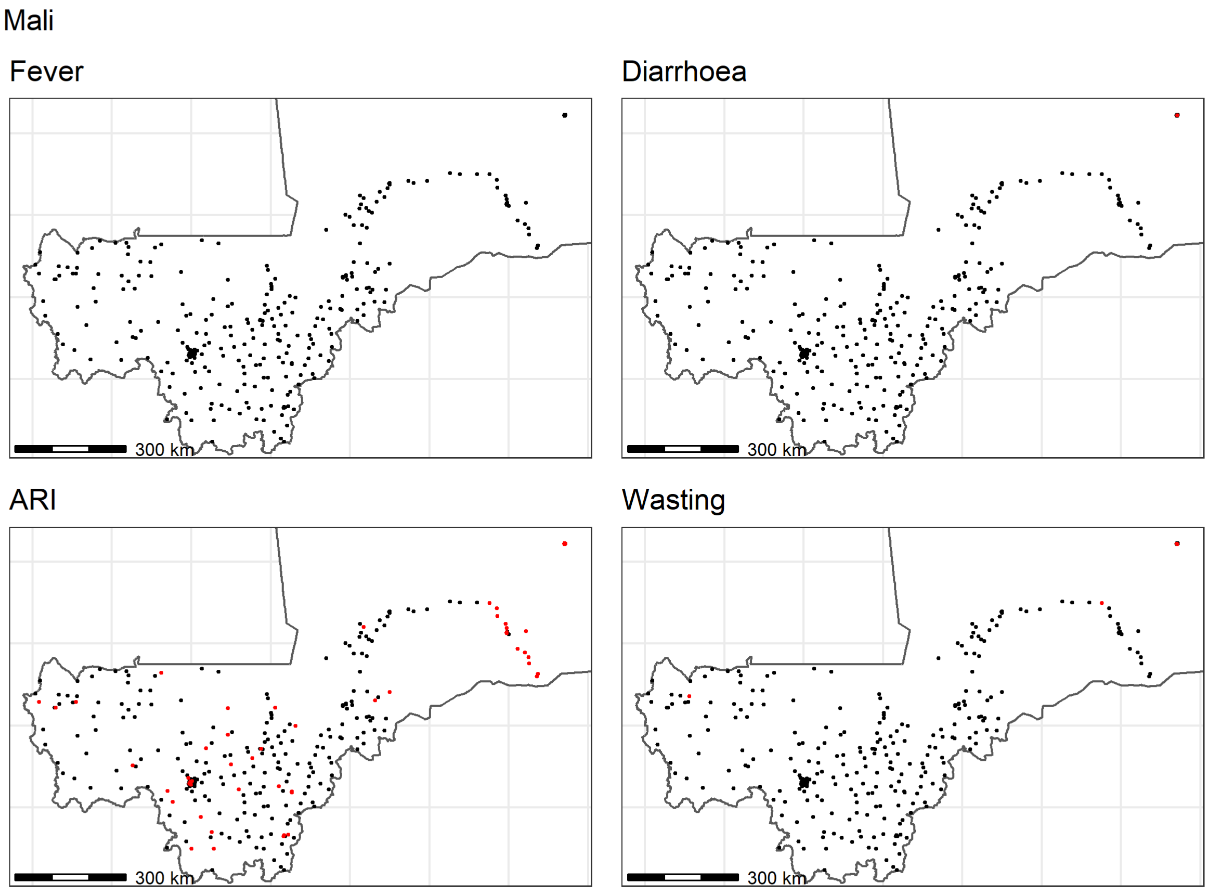
Mali
Fever
Diarrhoea
ARI
Wasting
300 km
300 km
300 km
300 km

Manipur

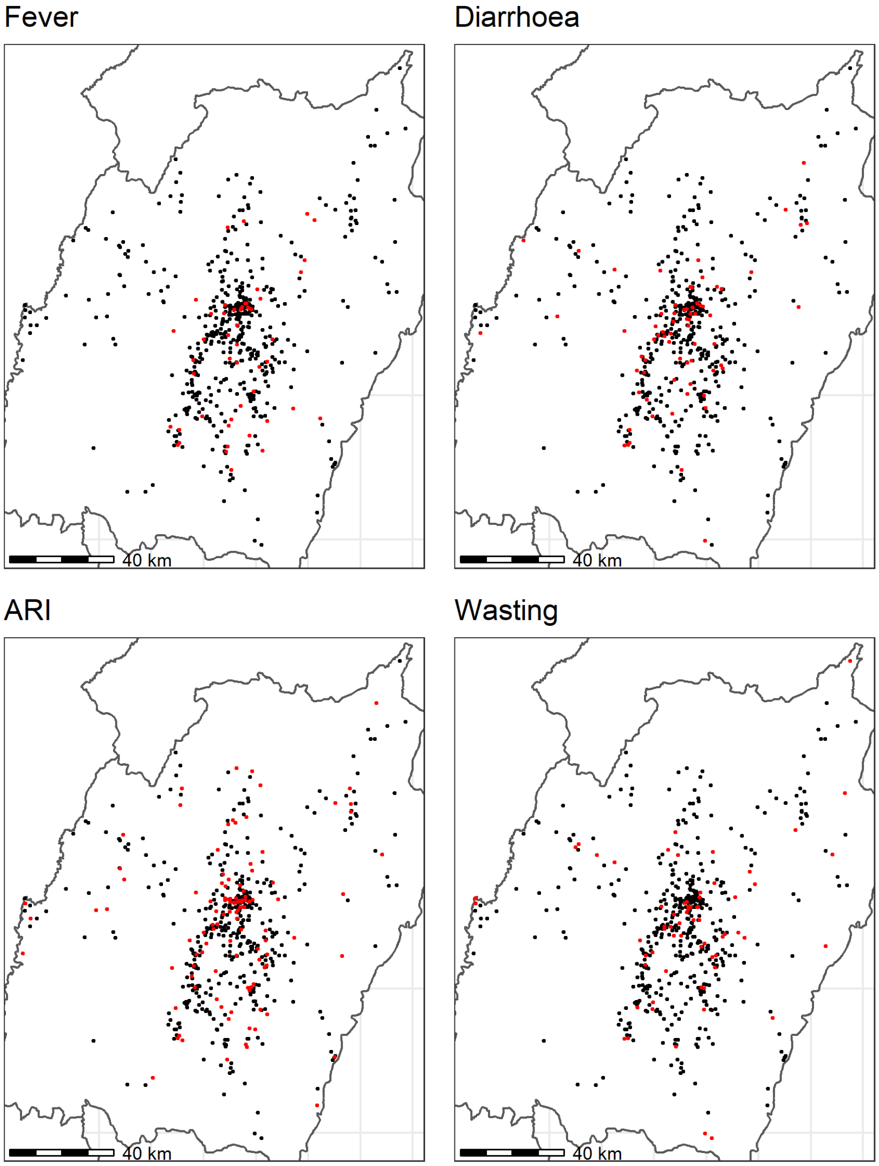

Meghalaya

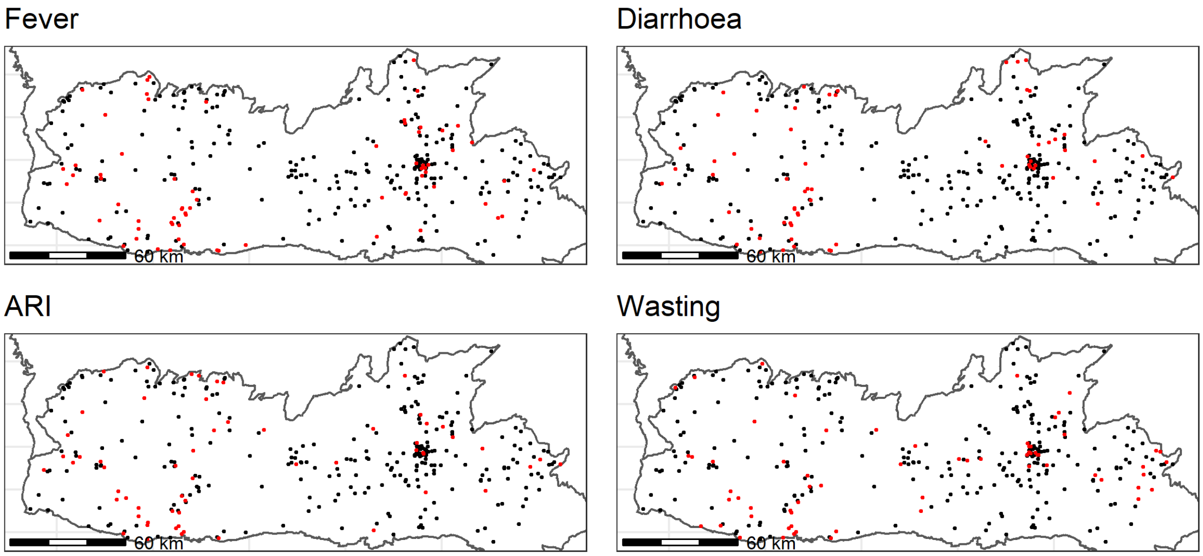

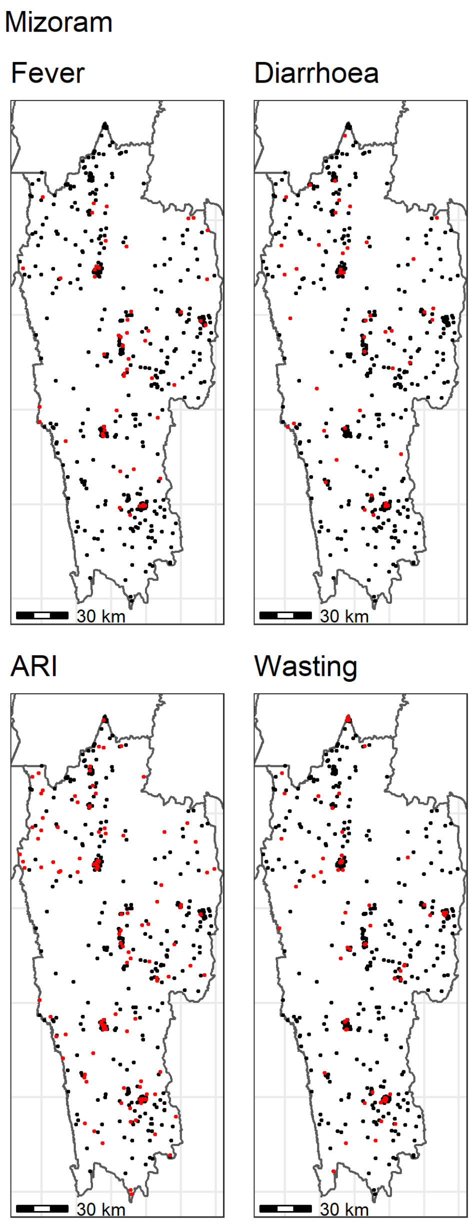
Mizoram
Fever
Diarrhoea
ARI
Wasting
30 km
30 km
30 km
30 km

Mozambique

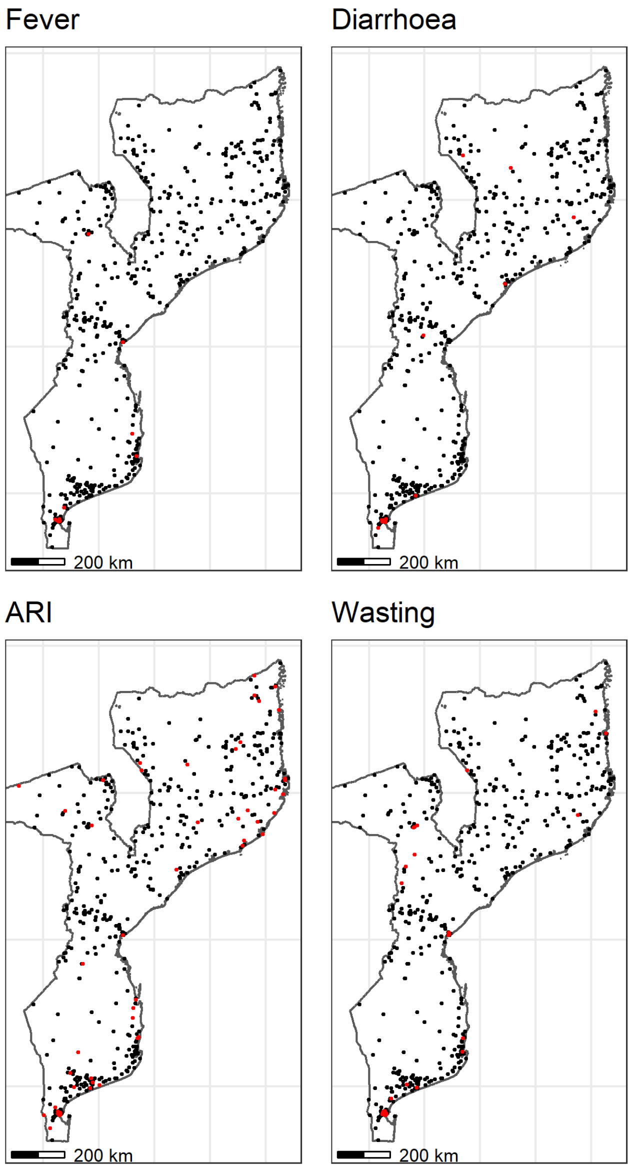

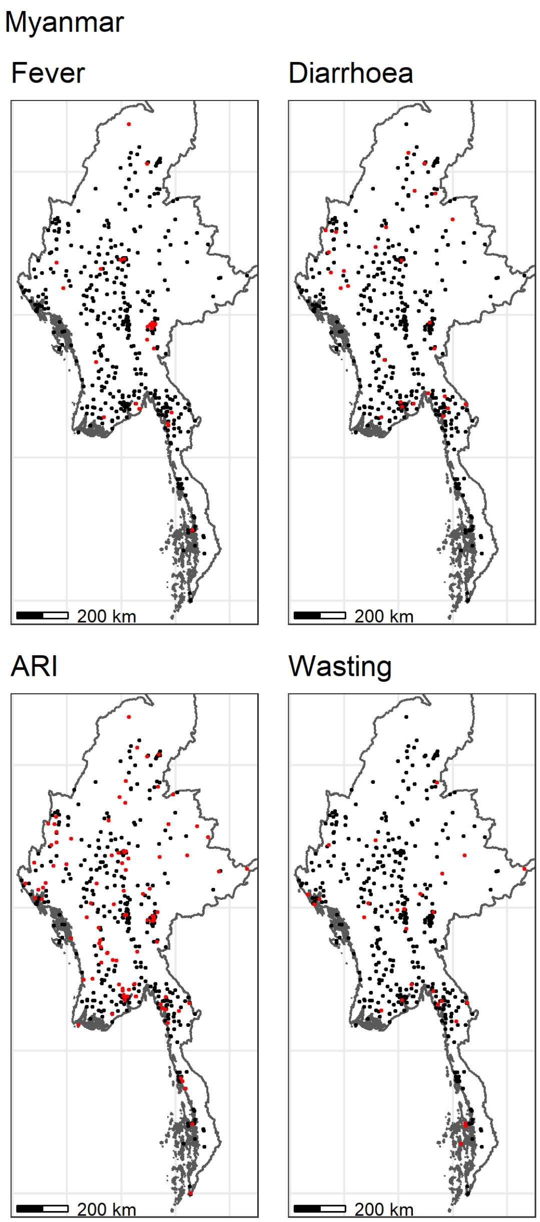
Myanmar
Fever
200 km
Diarrhoea
200 km
ARI
200 km
Wasting
200 km

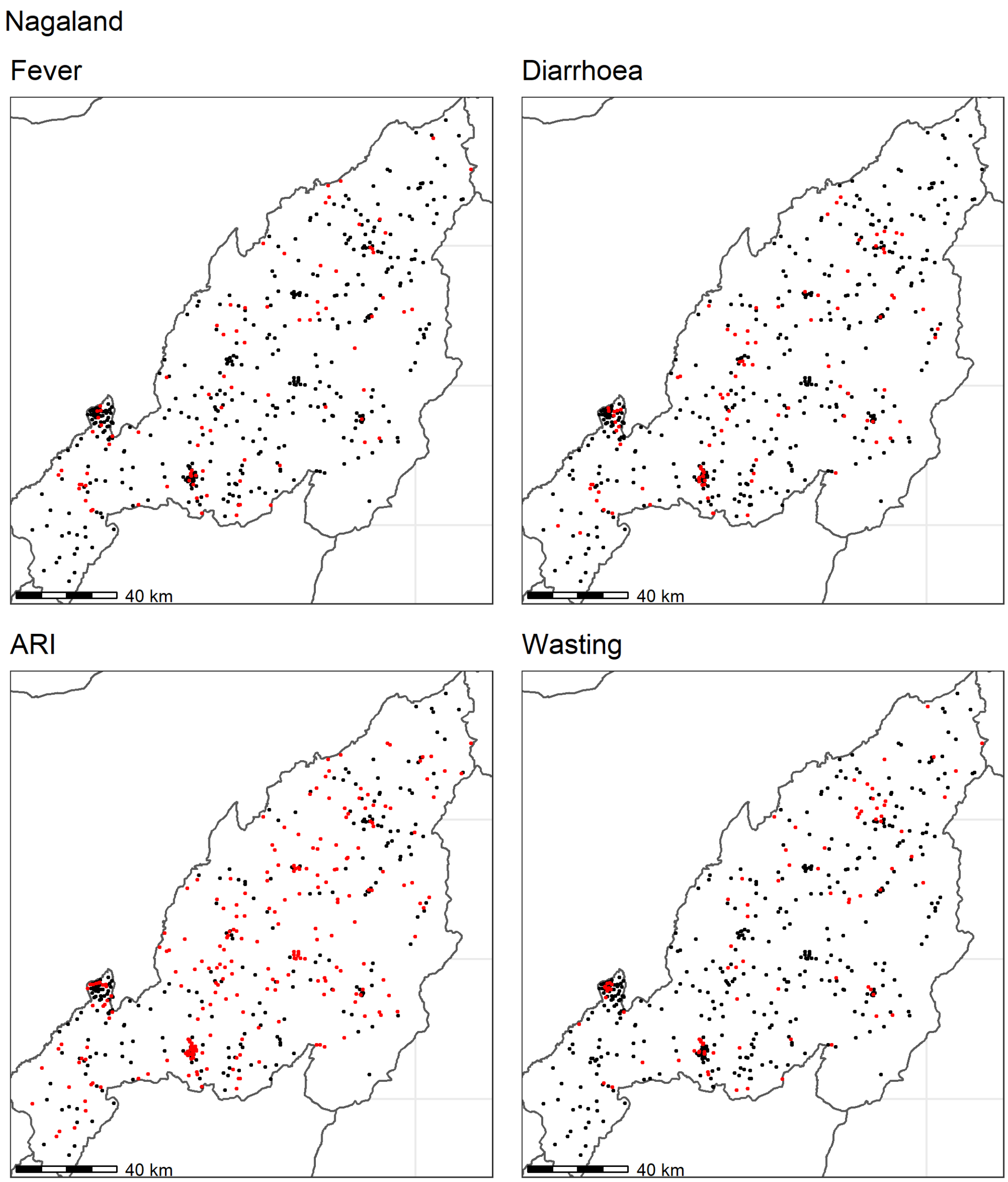
Nagaland
Fever
40 km
Diarrhoea
40 km
ARI
40 km
Wasting
40 km

Namibia

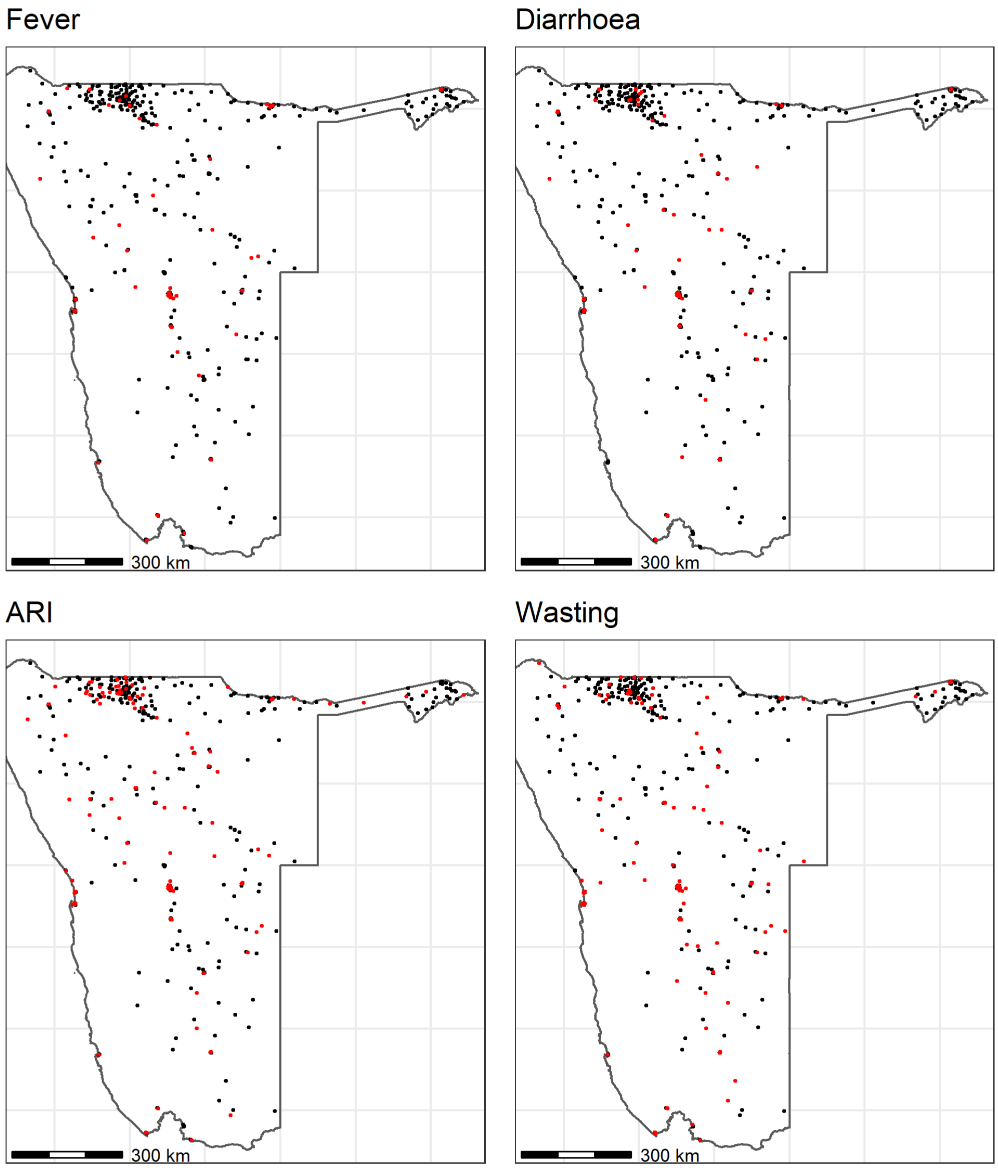

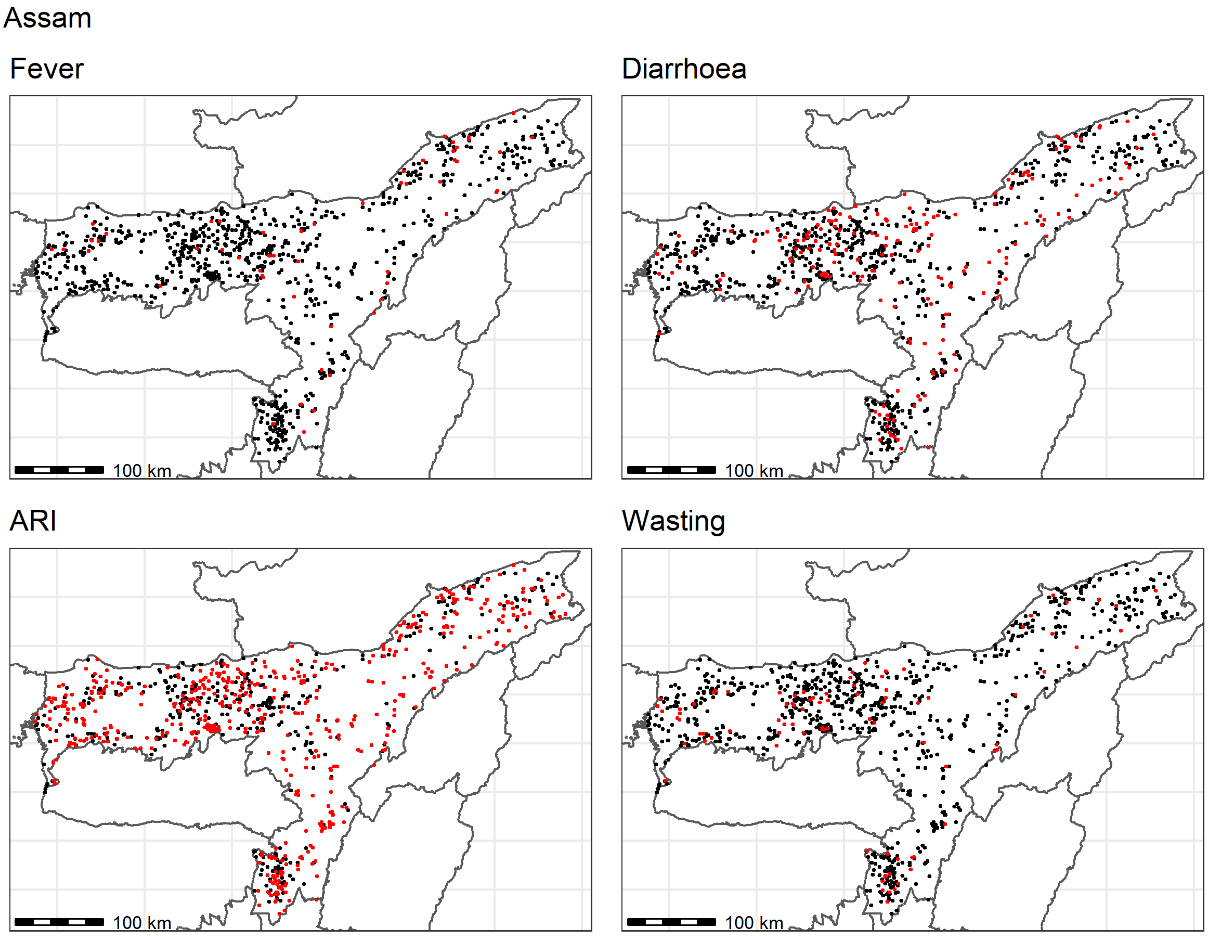

Assam
Fever
Diarrhoea
ARI
Wasting
100 km
100 km
100 km
100 km

NCT of Delhi

Fever

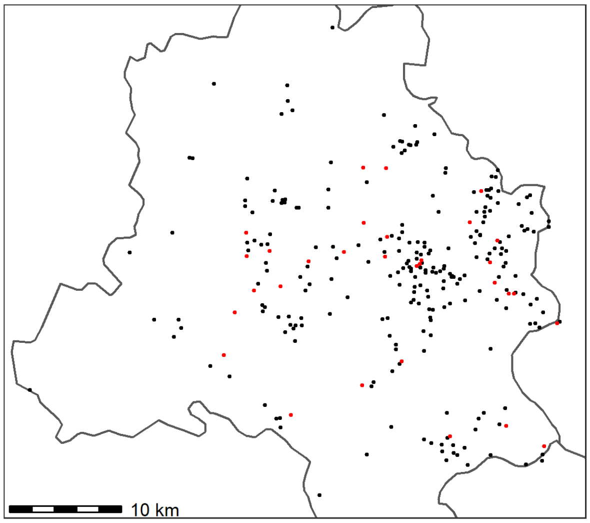

Diarrhoea

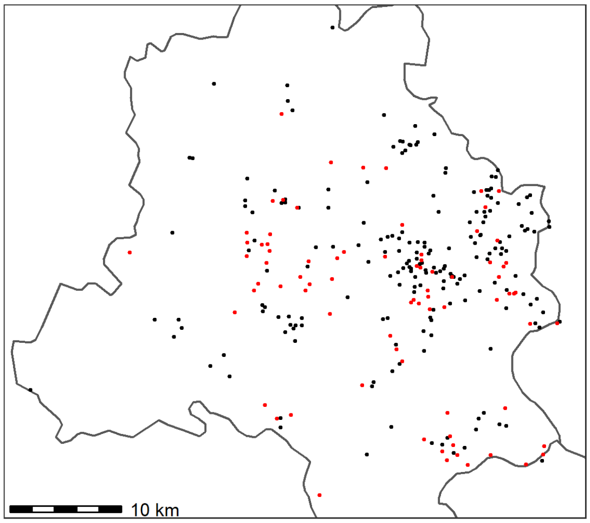

ARI

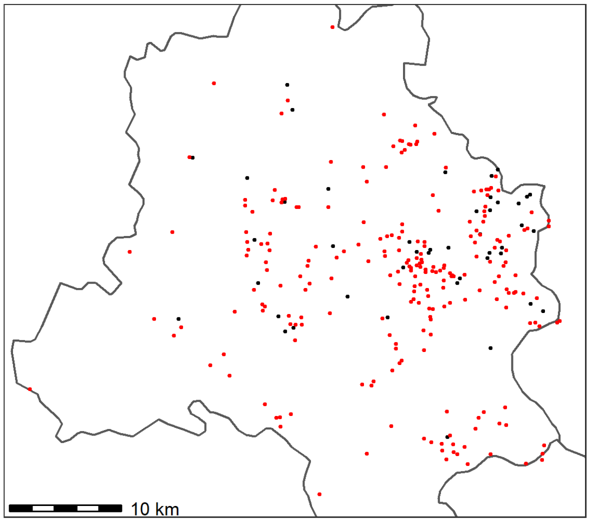

Wasting

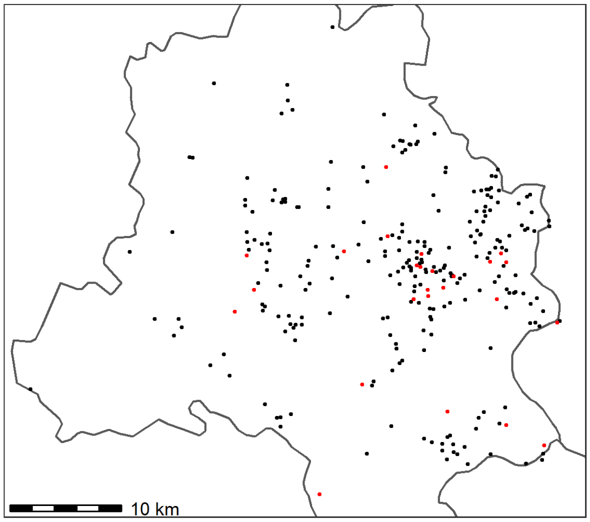

Nepal

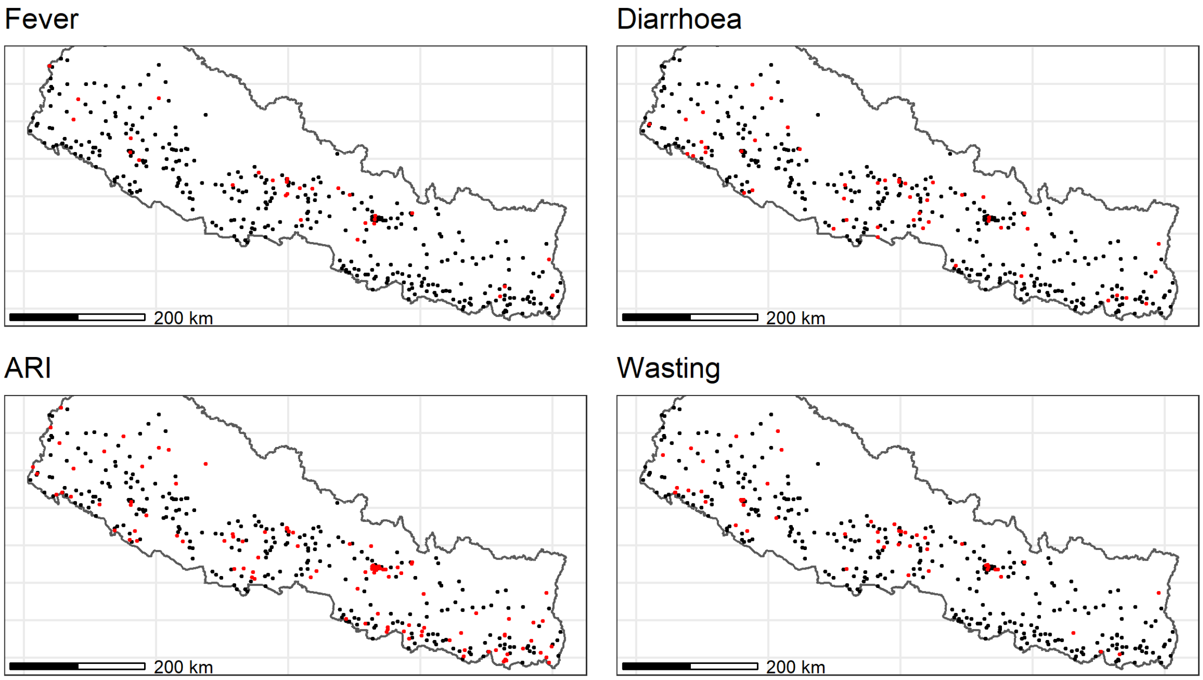

Nigeria

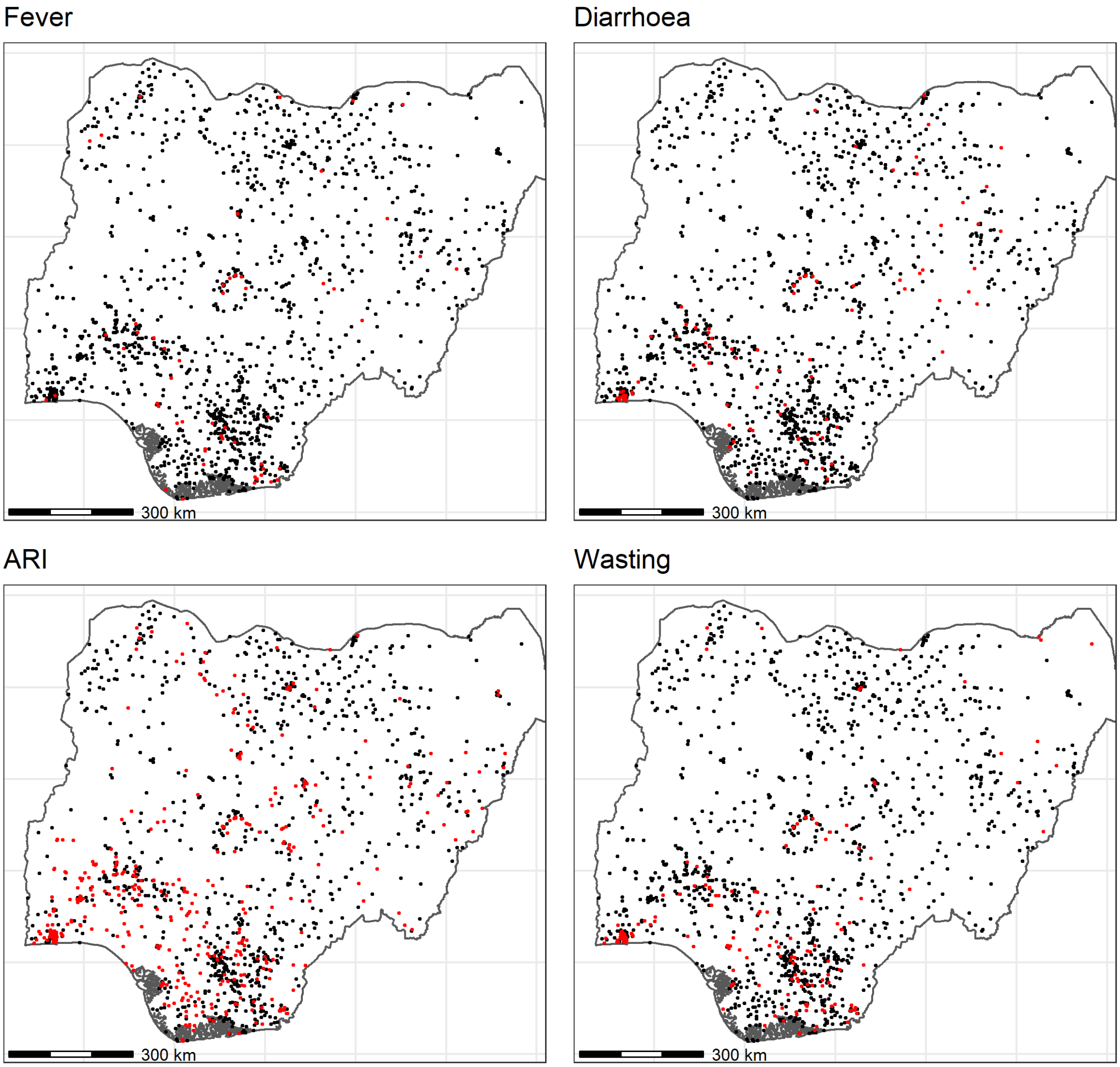

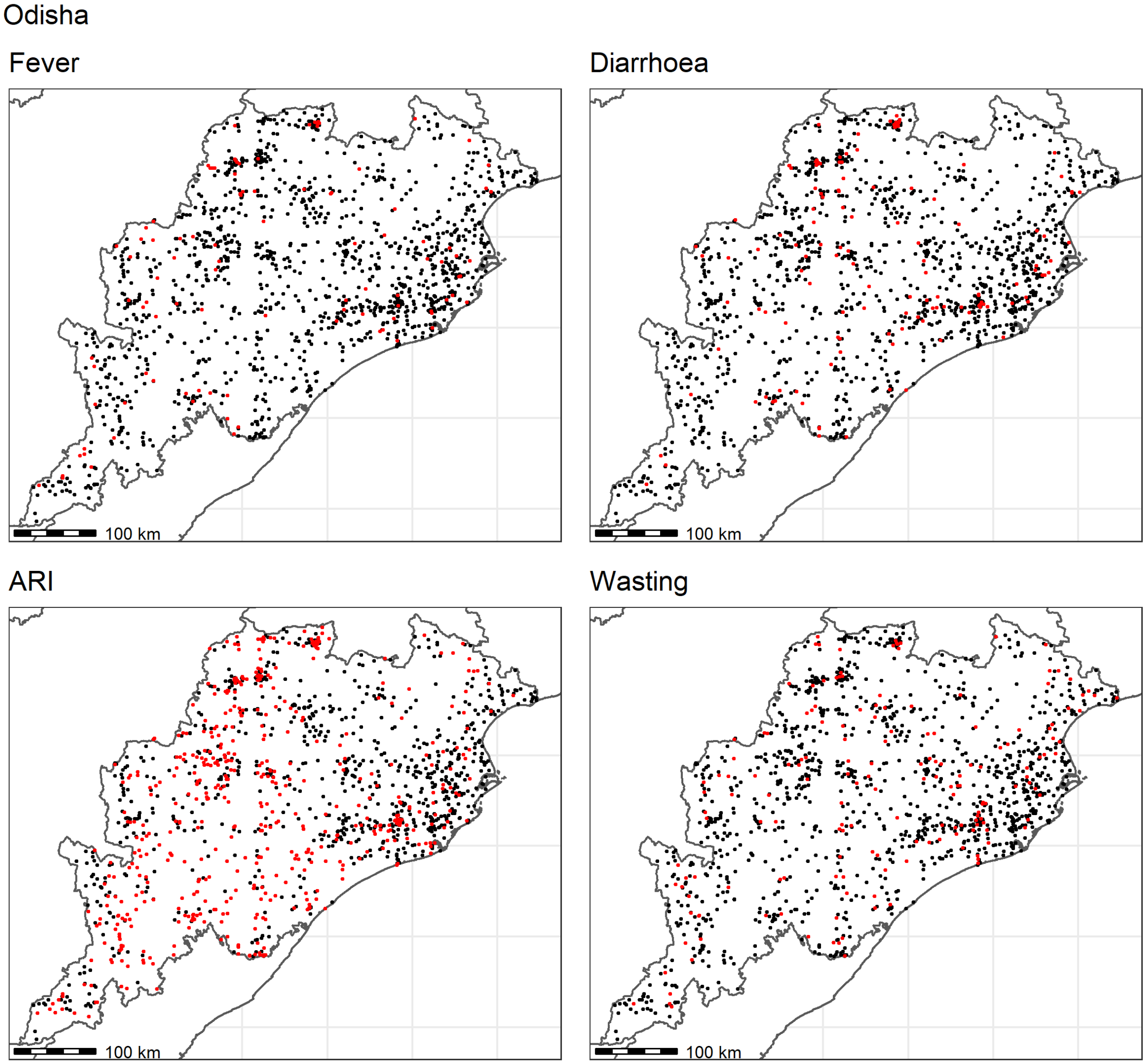
Odisha
Fever
Diarrhoea
ARI
Wasting
100 km
100 km
100 km
100 km

Pakistan

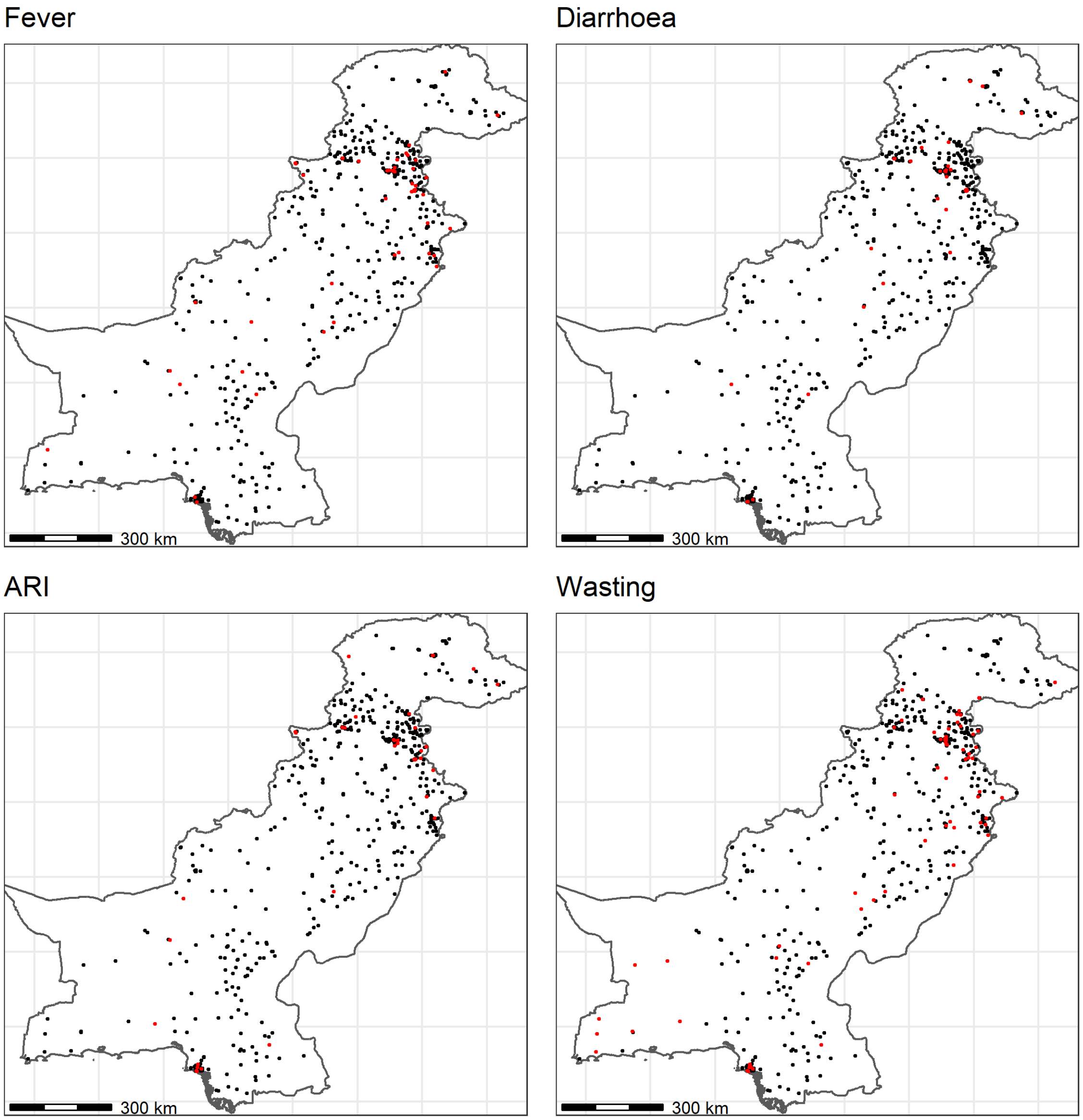

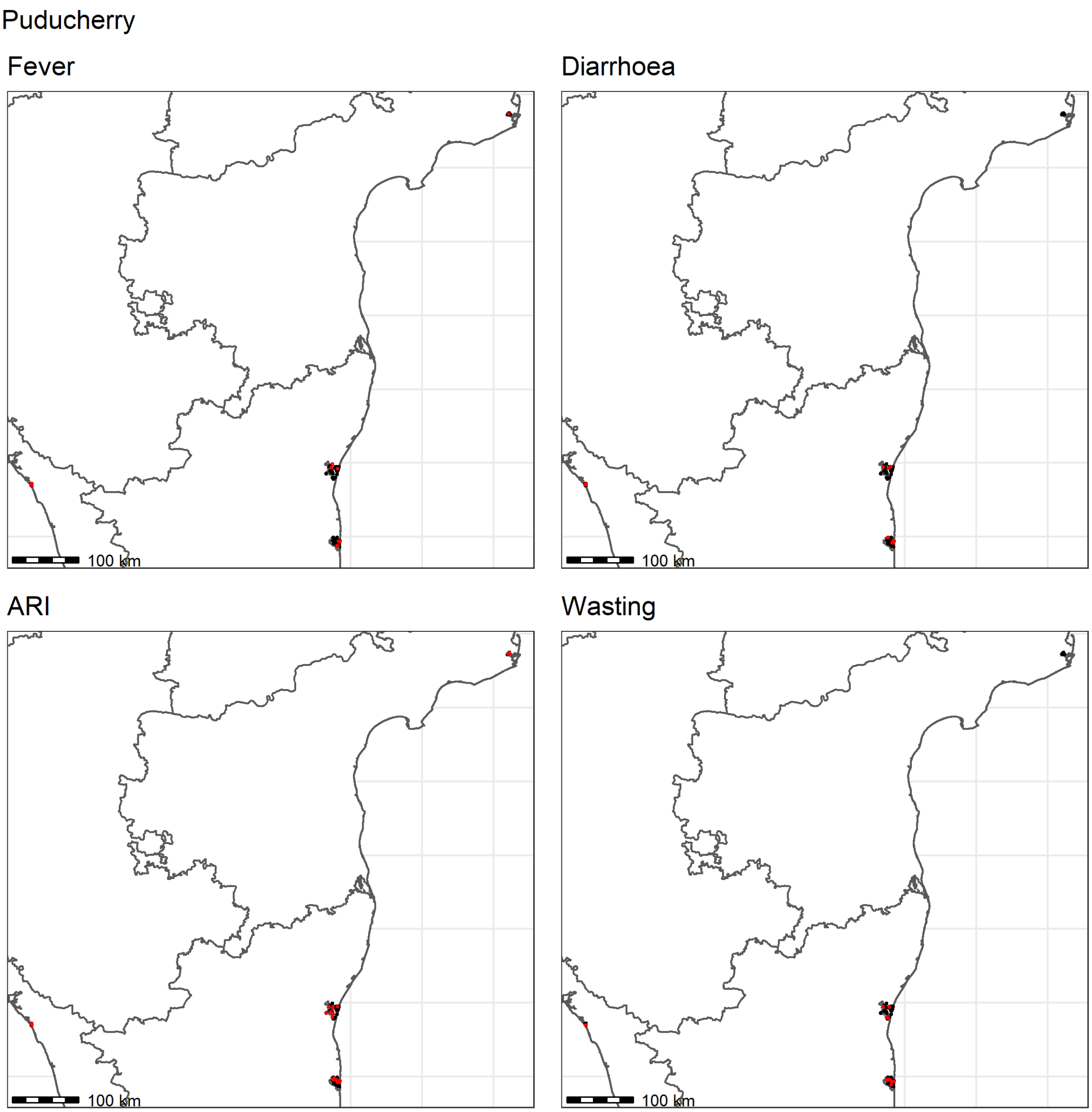
Puducherry
Fever
100 km
Diarrhoea
100 km
ARI
100 km
Wasting
100 km

Punjab

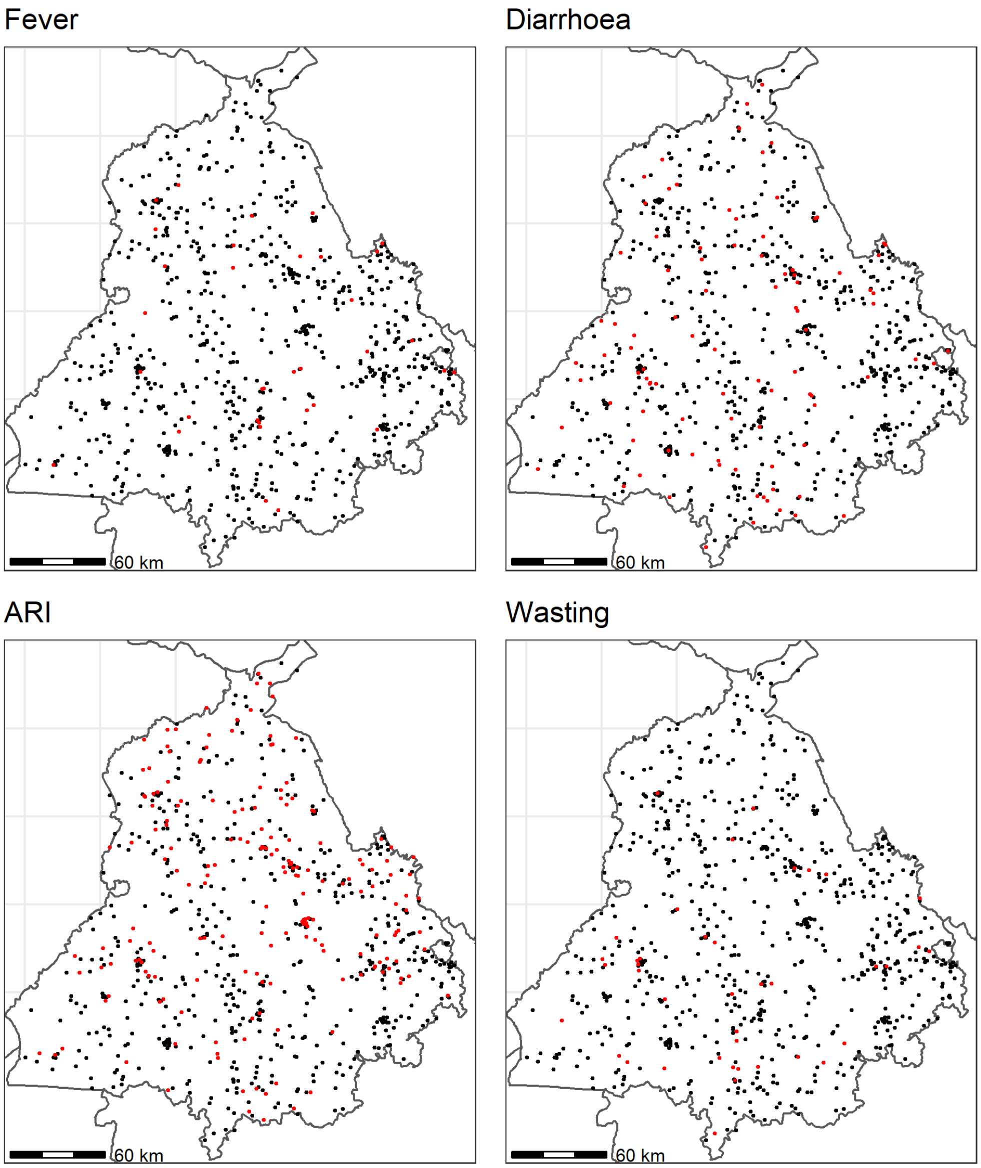

Rajasthan

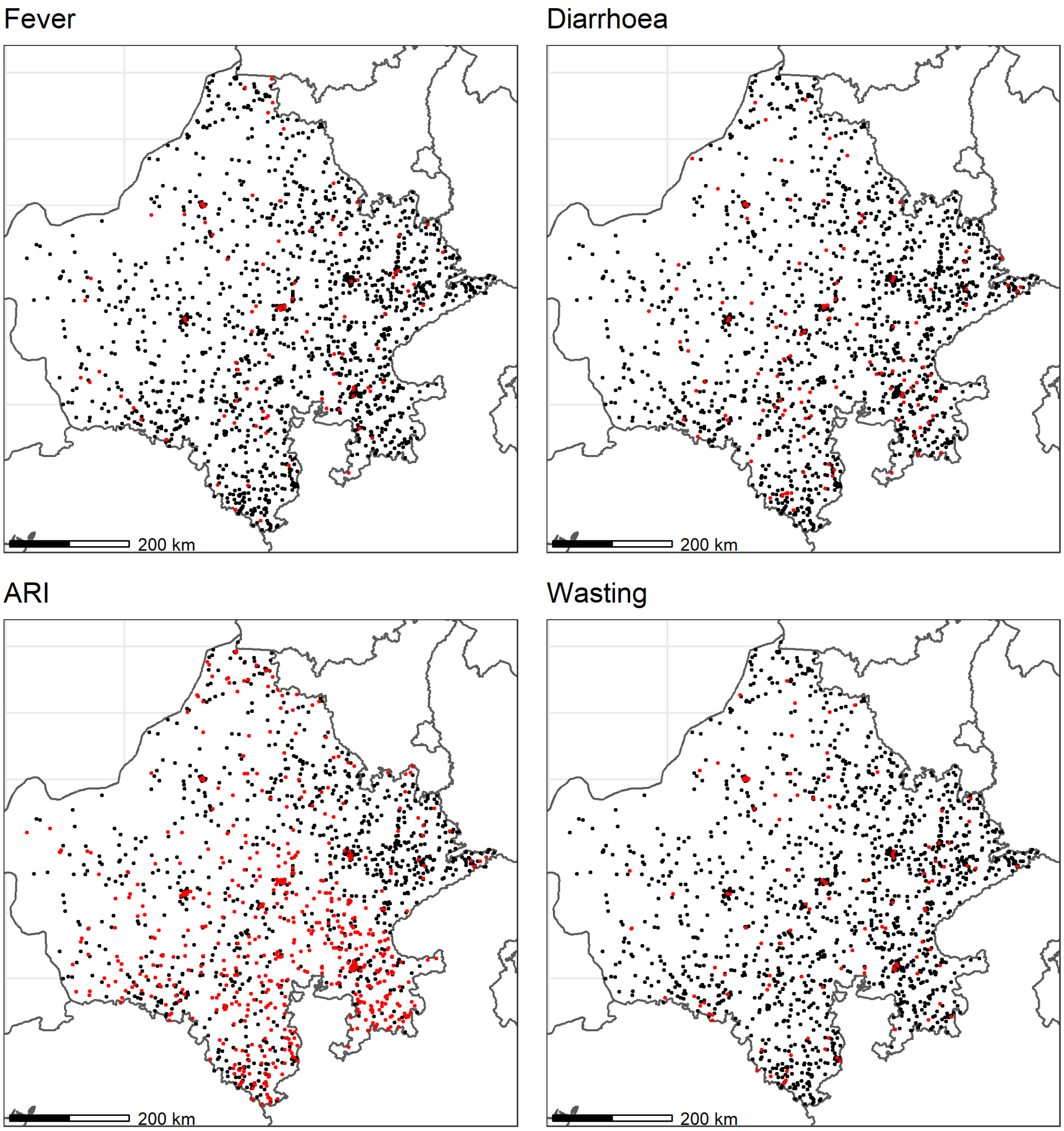

Rwanda

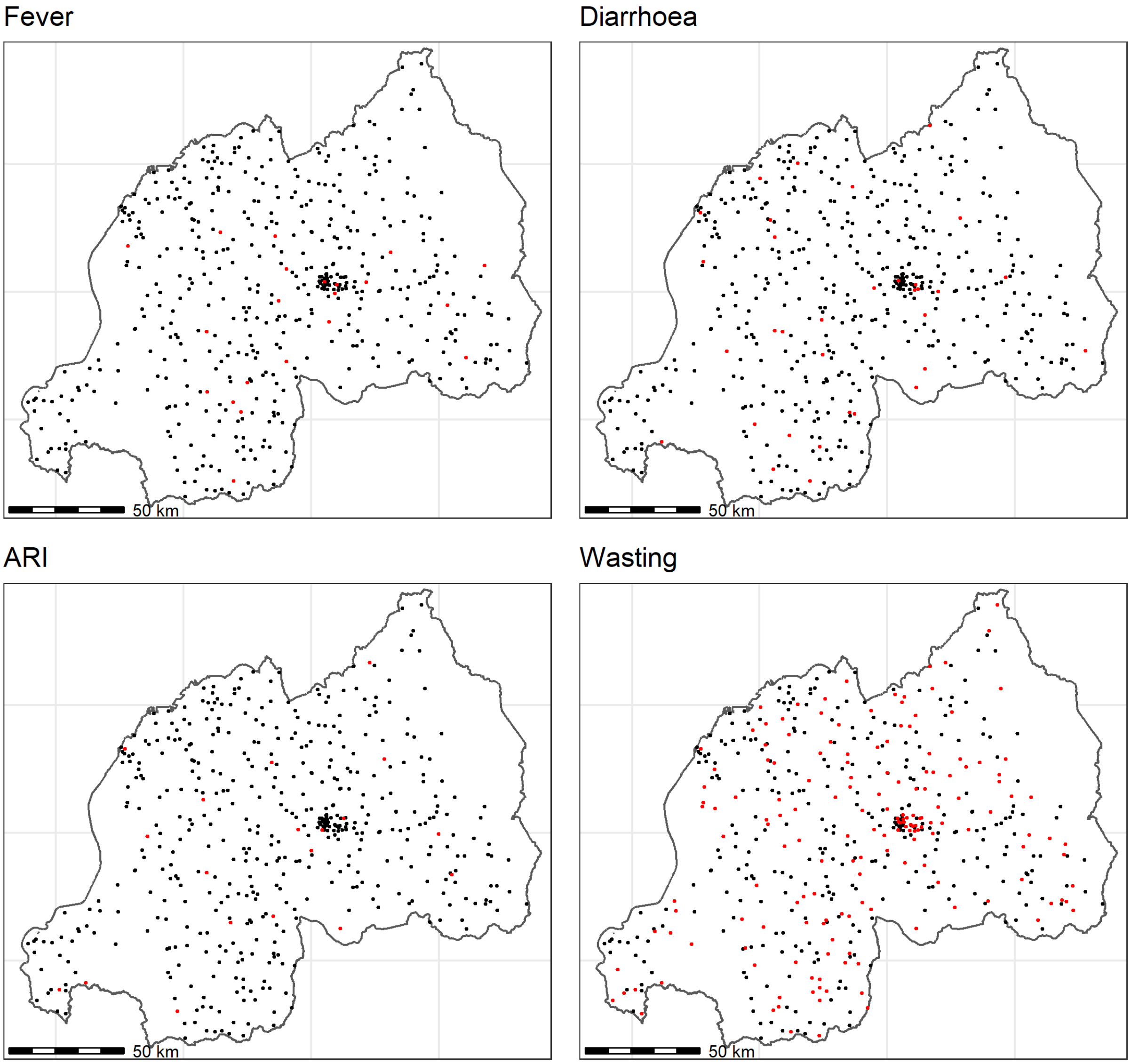

Senegal

Fever

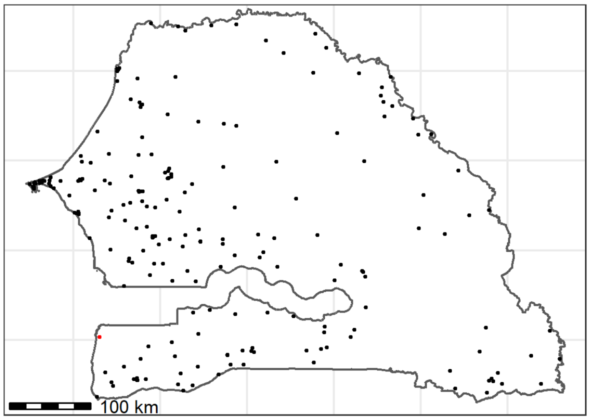

Diarrhoea

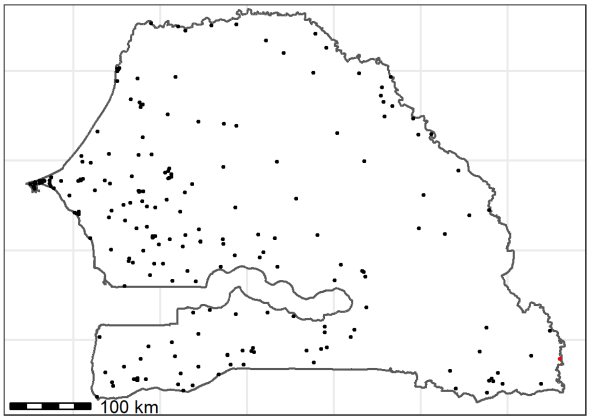

ARI

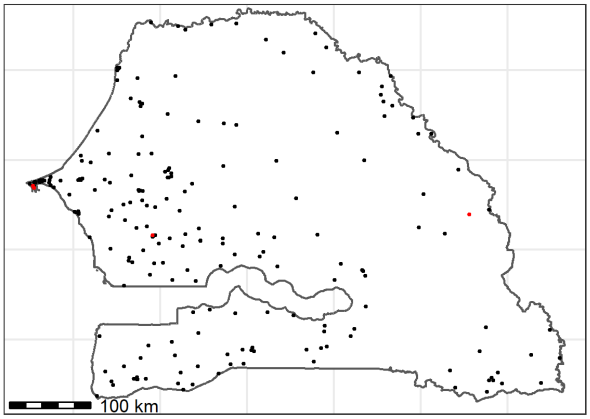

Wasting

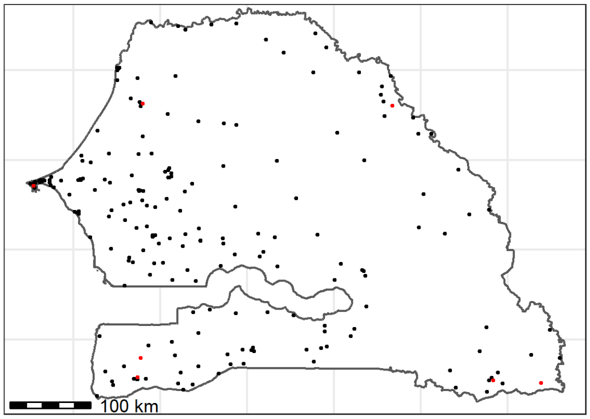

Bangladesh

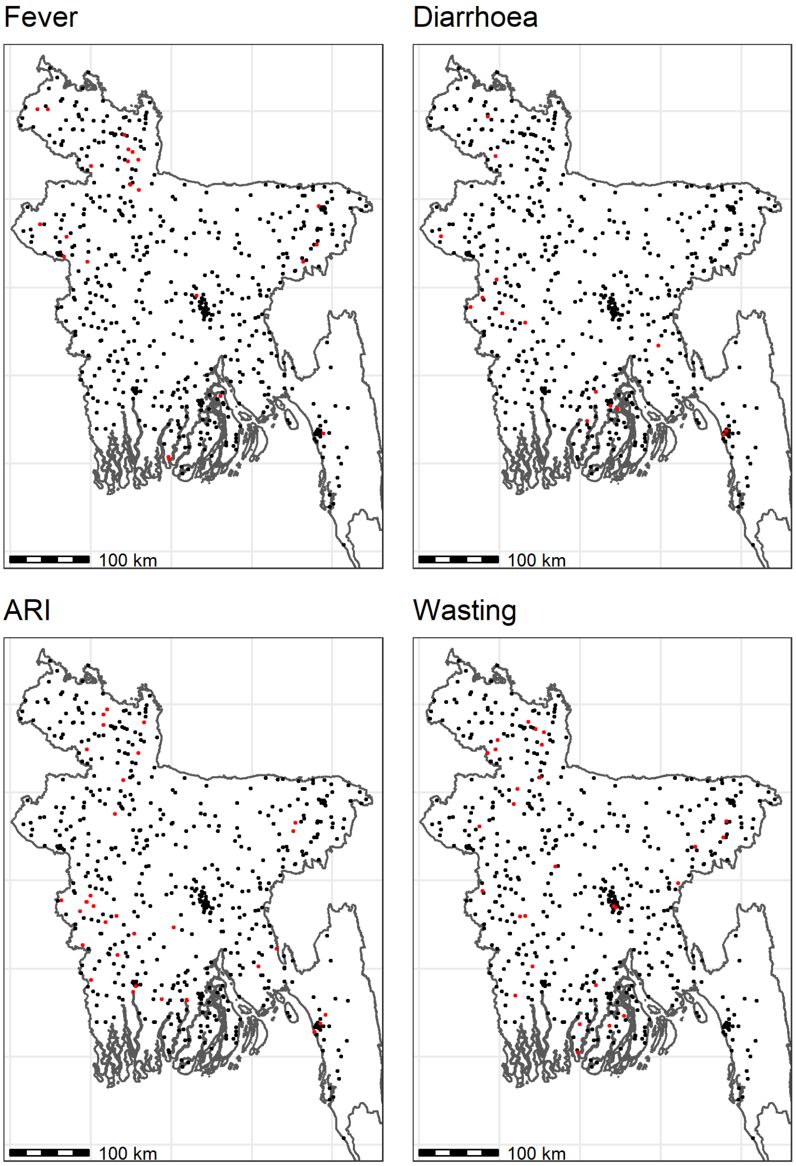

Sierra Leone

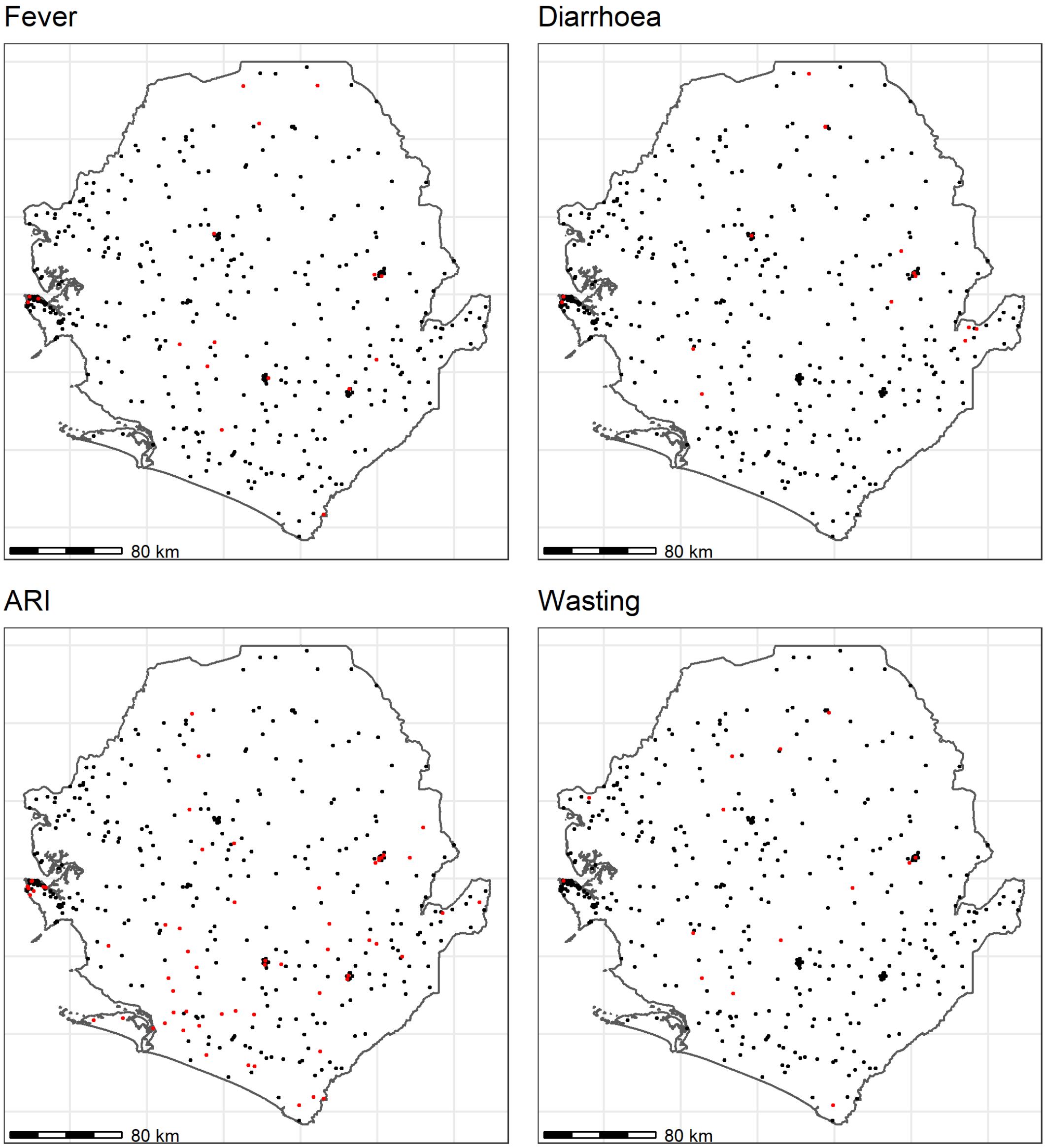

Sikkim

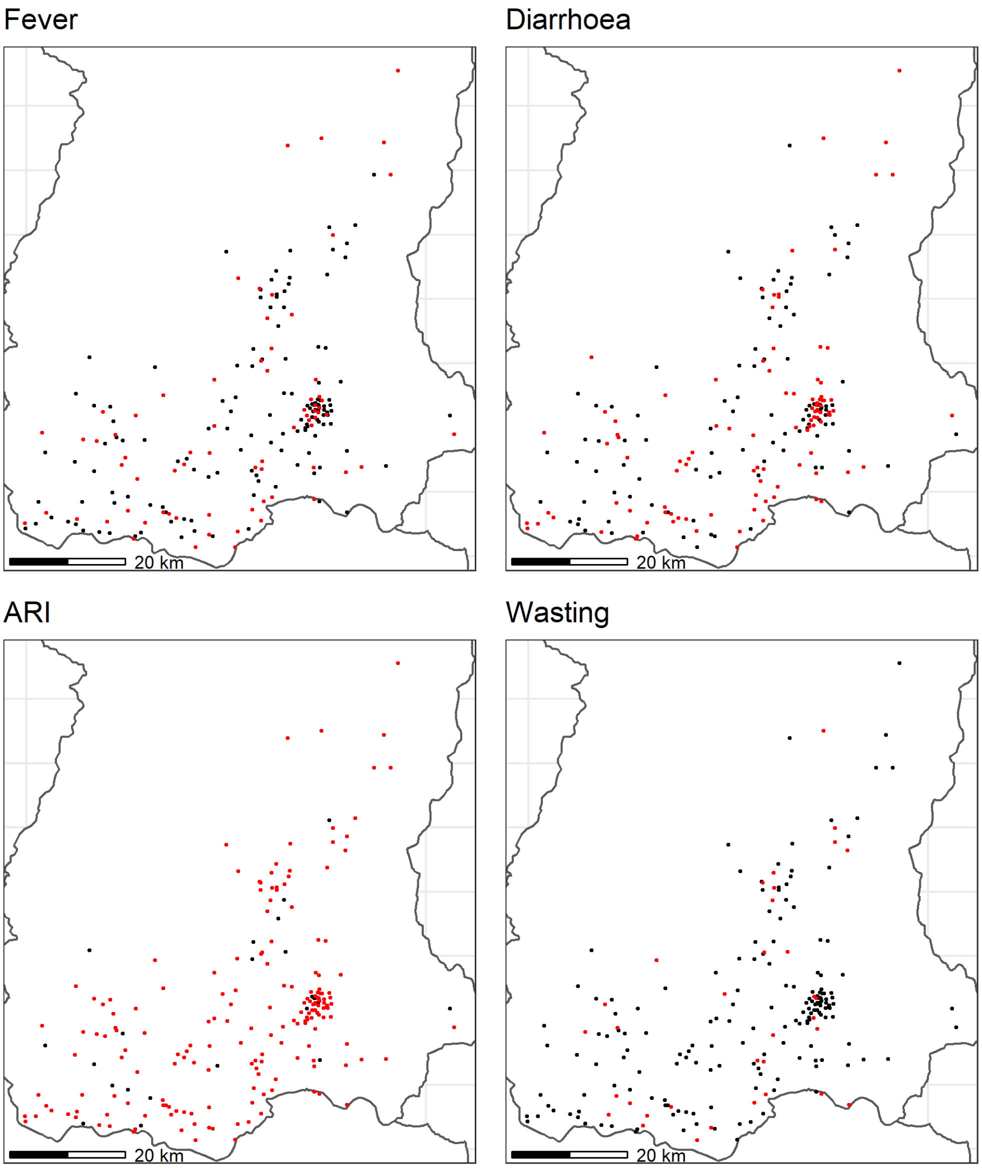

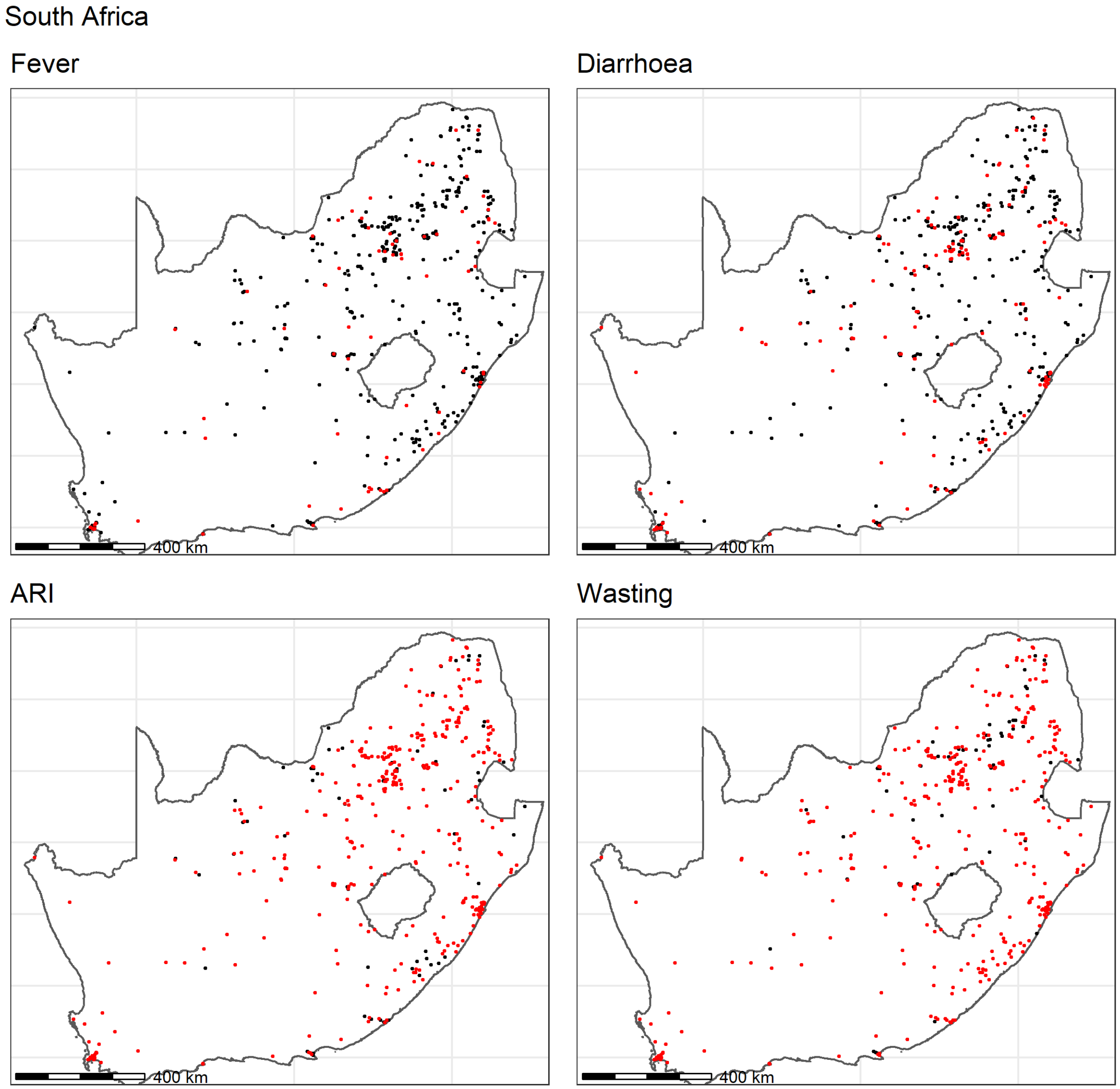
South Africa
Fever
Diarrhoea
ARI
Wasting
400 km
400 km
400 km
400 km

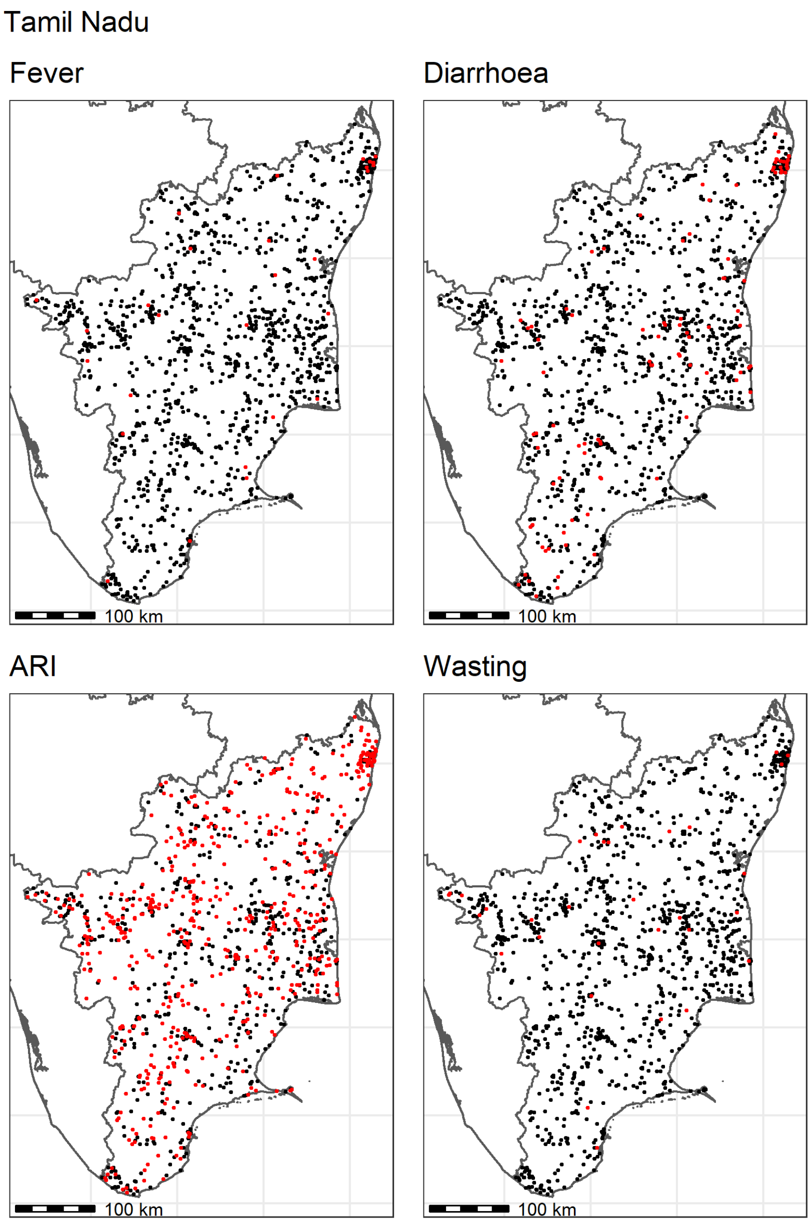
Tamil Nadu
Fever
Diarrhoea
ARI
Wasting
100 km
100 km
100 km
100 km

Tanzania

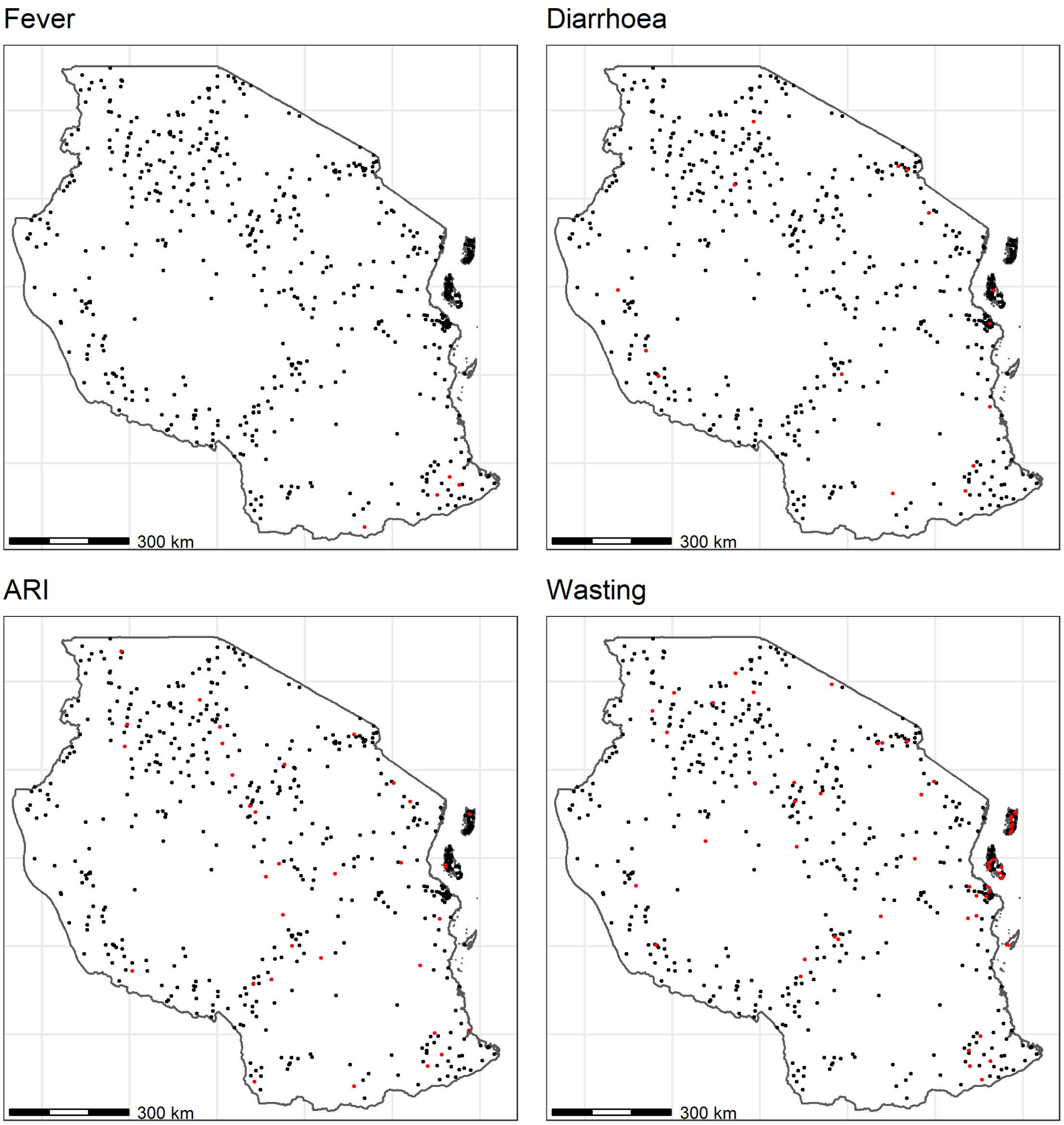

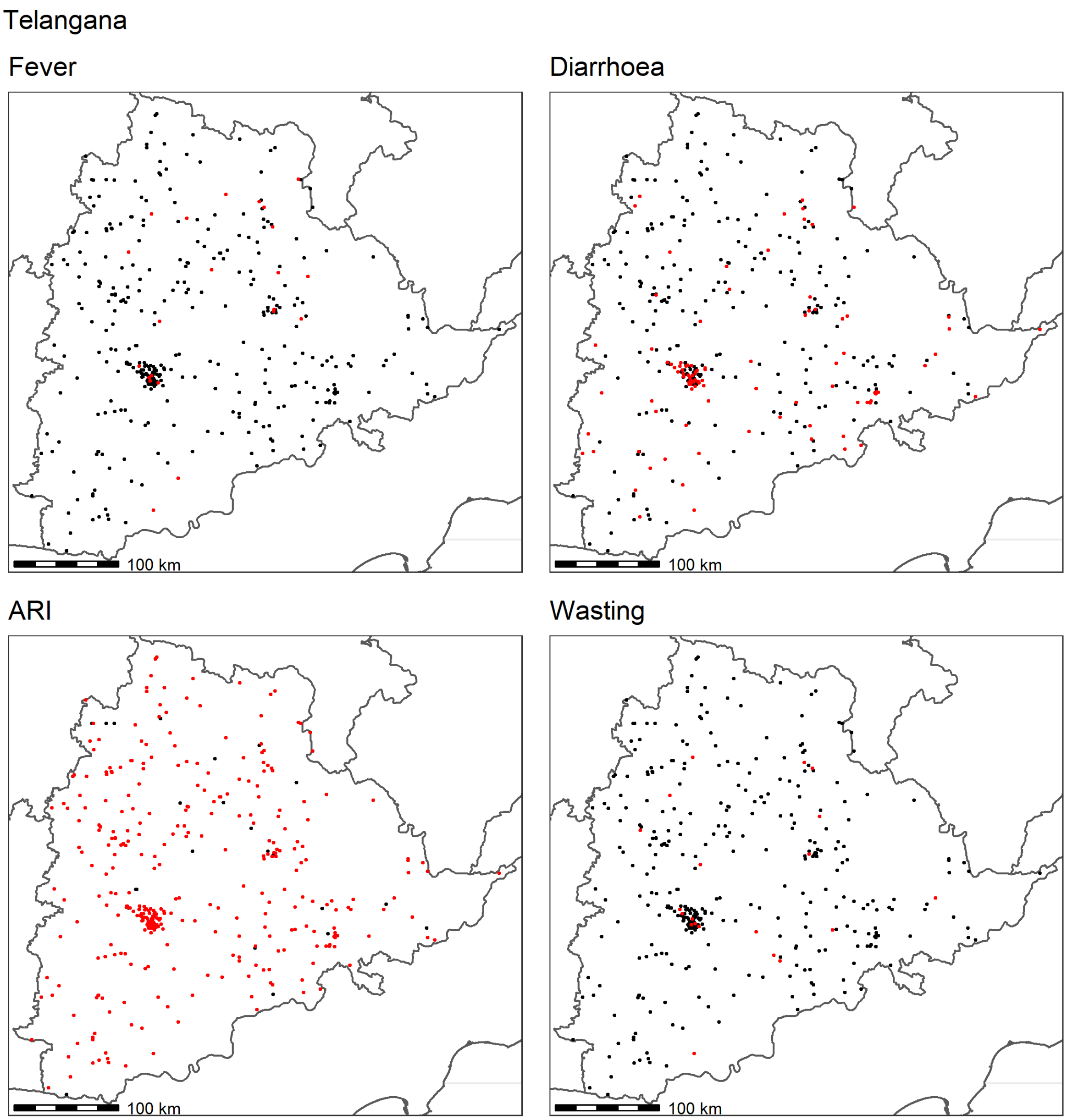
Telangana
Fever
Diarrhoea
100 km
100 km
ARI
Wasting
100 km
100 km

Timor-Leste

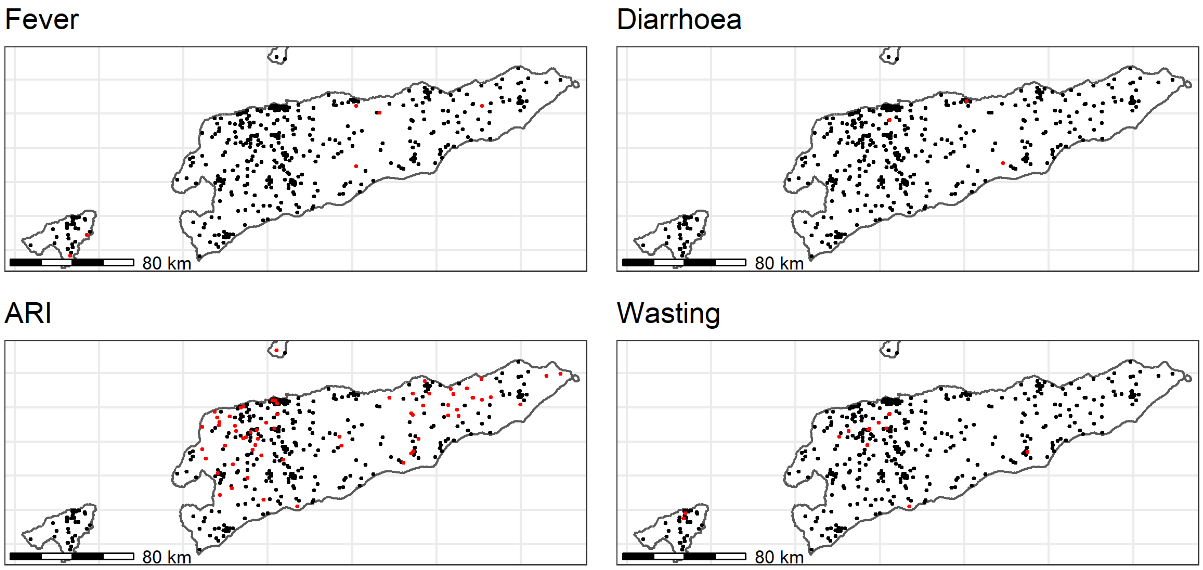

Togo

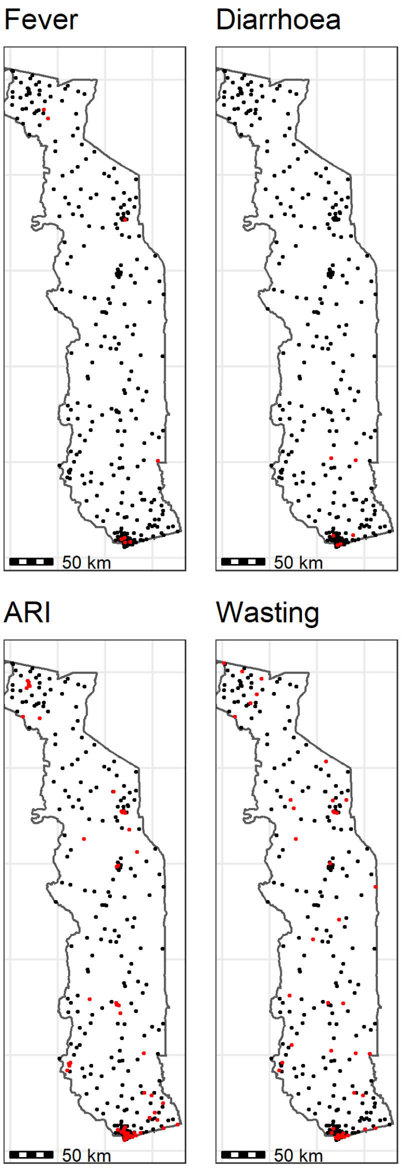

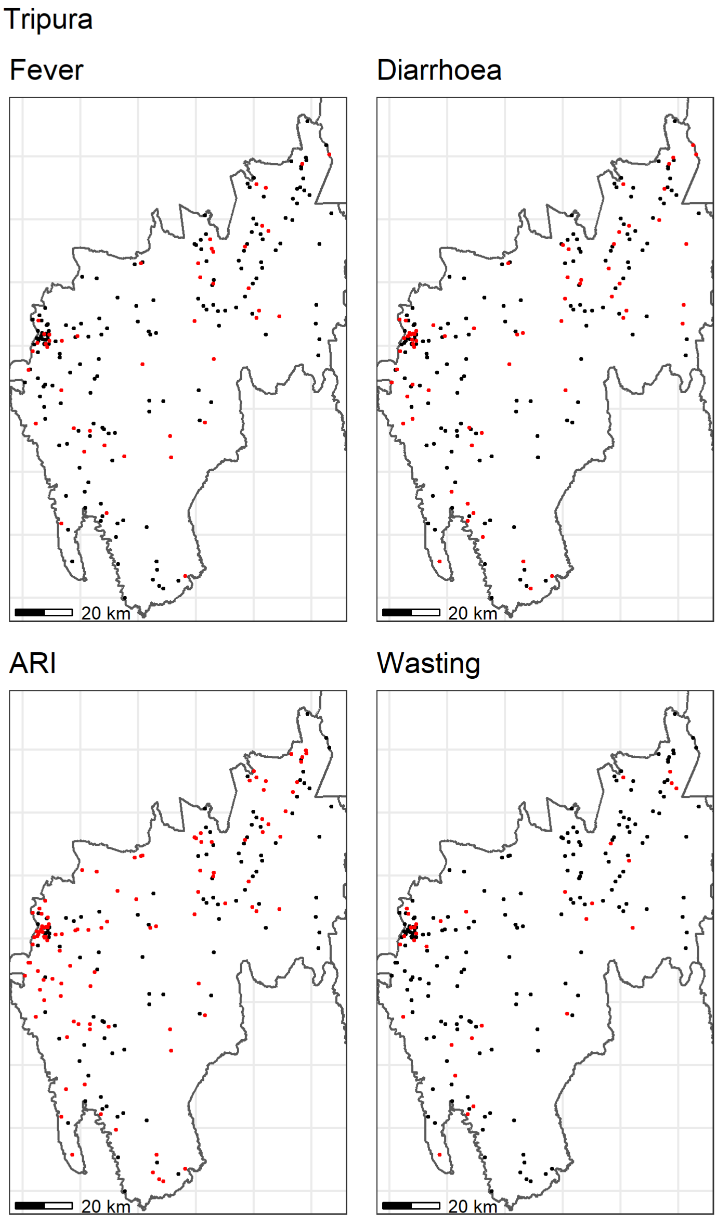
Tripura
Fever
Diarrhoea
ARI
Wasting
20 km
20 km
20 km
20 km

Uganda

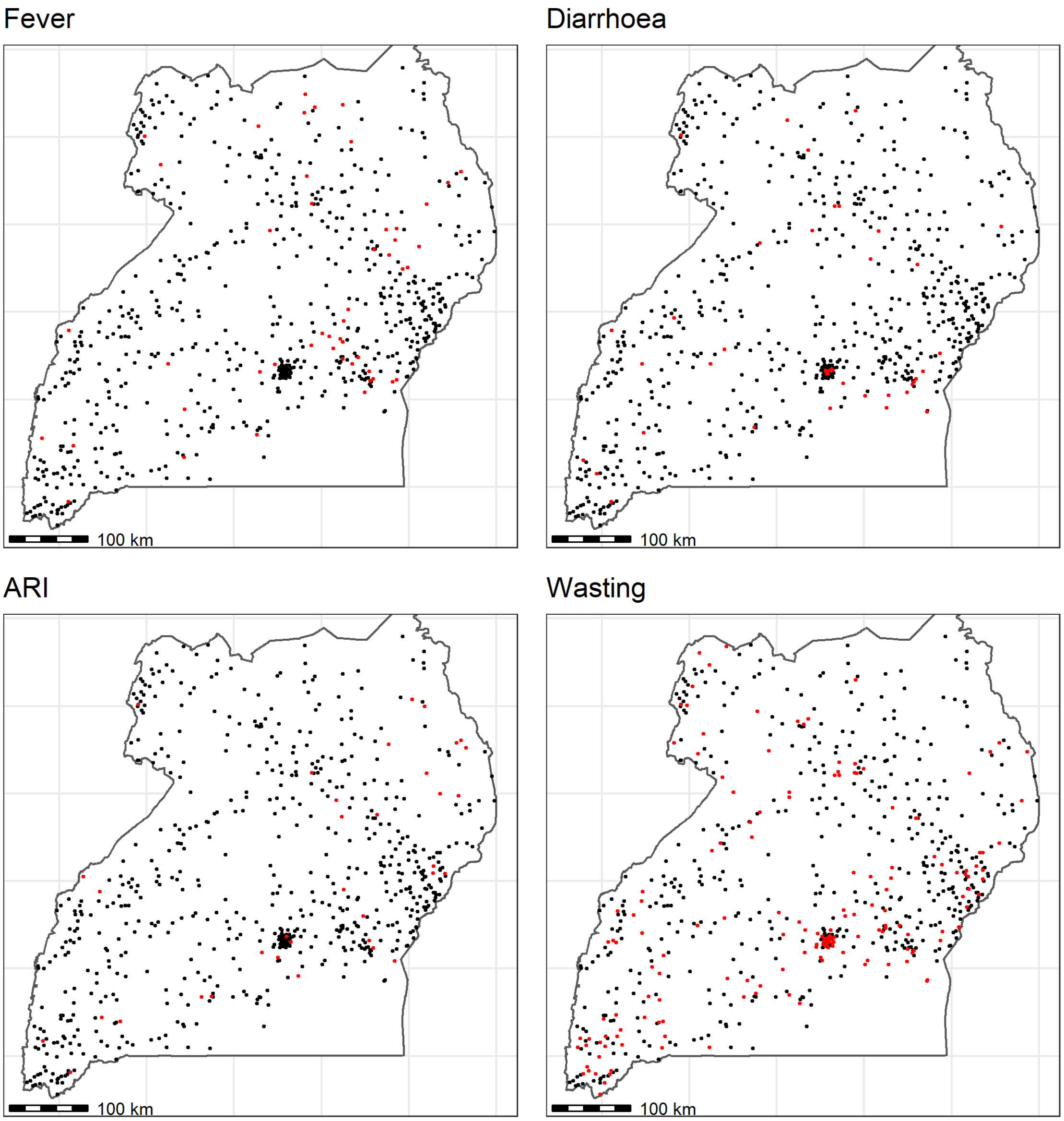

Benin

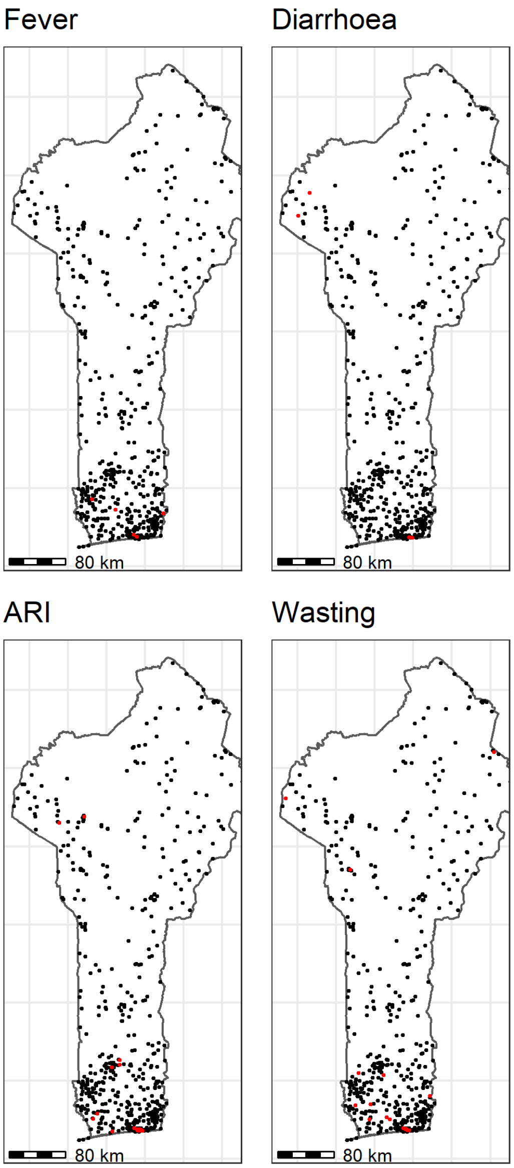

Uttar Pradesh

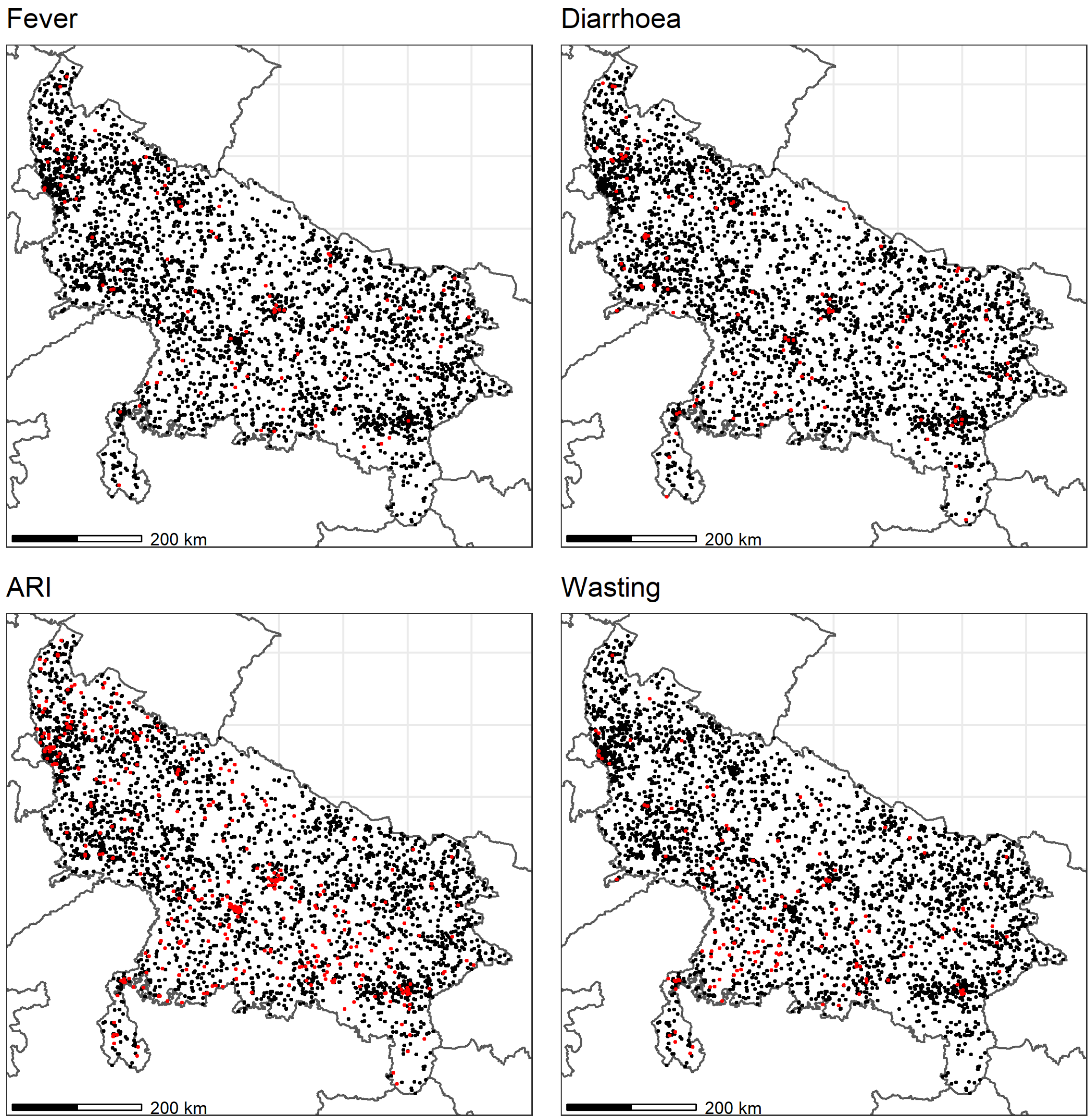

Uttarakhand

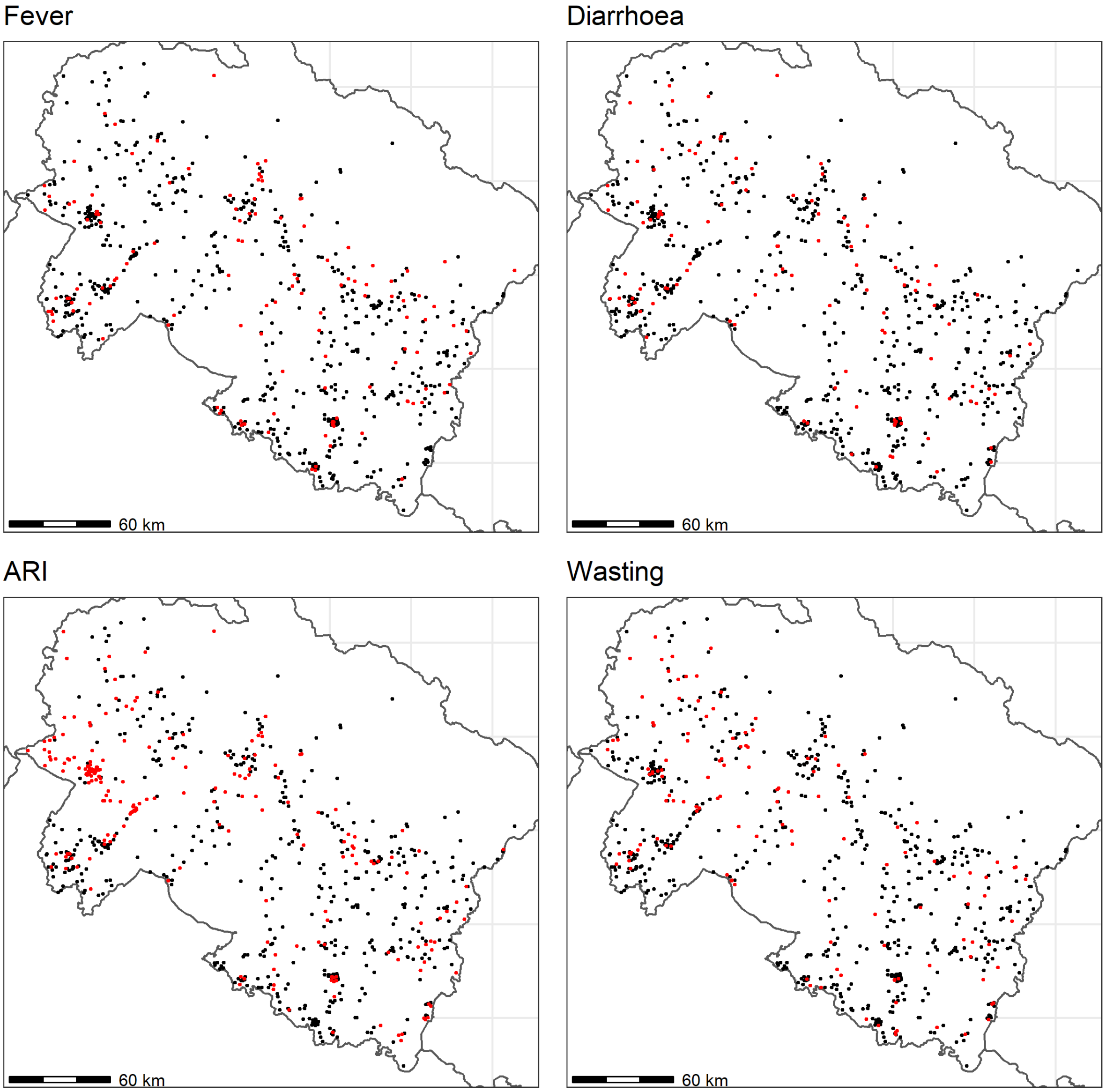

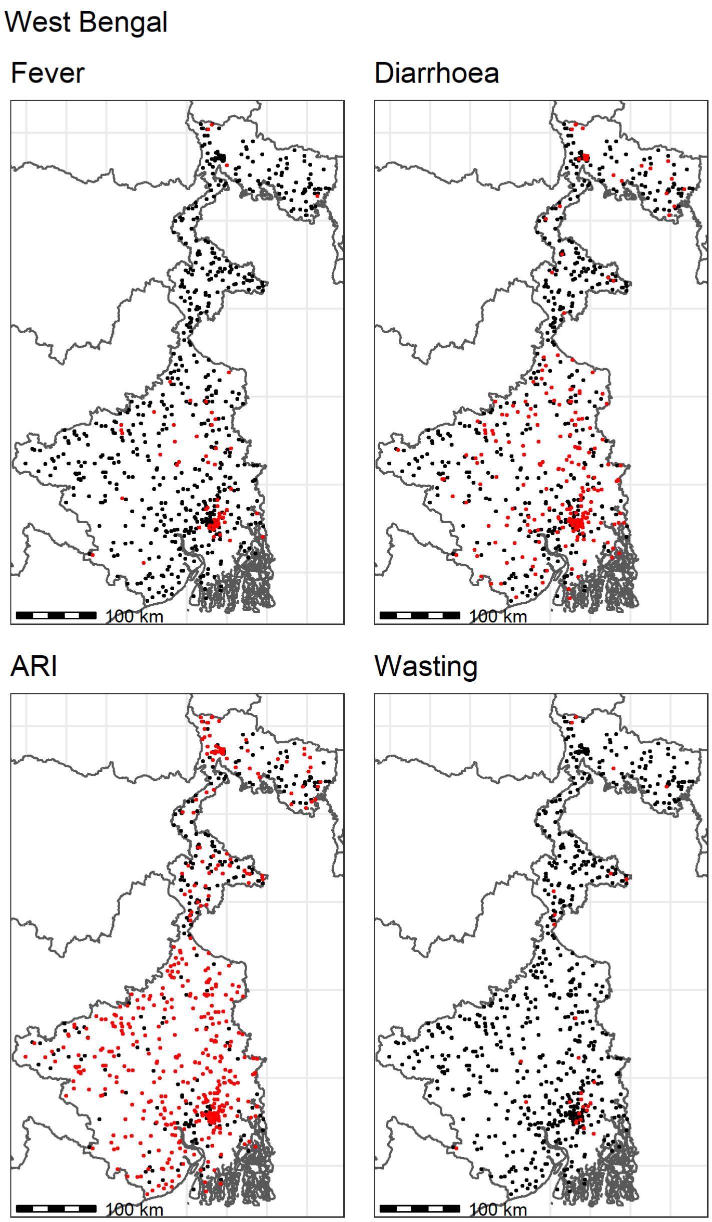
West Bengal
Fever
Diarrhoea
ARI
Wasting
100 km
100 km
100 km
100 km

Zambia

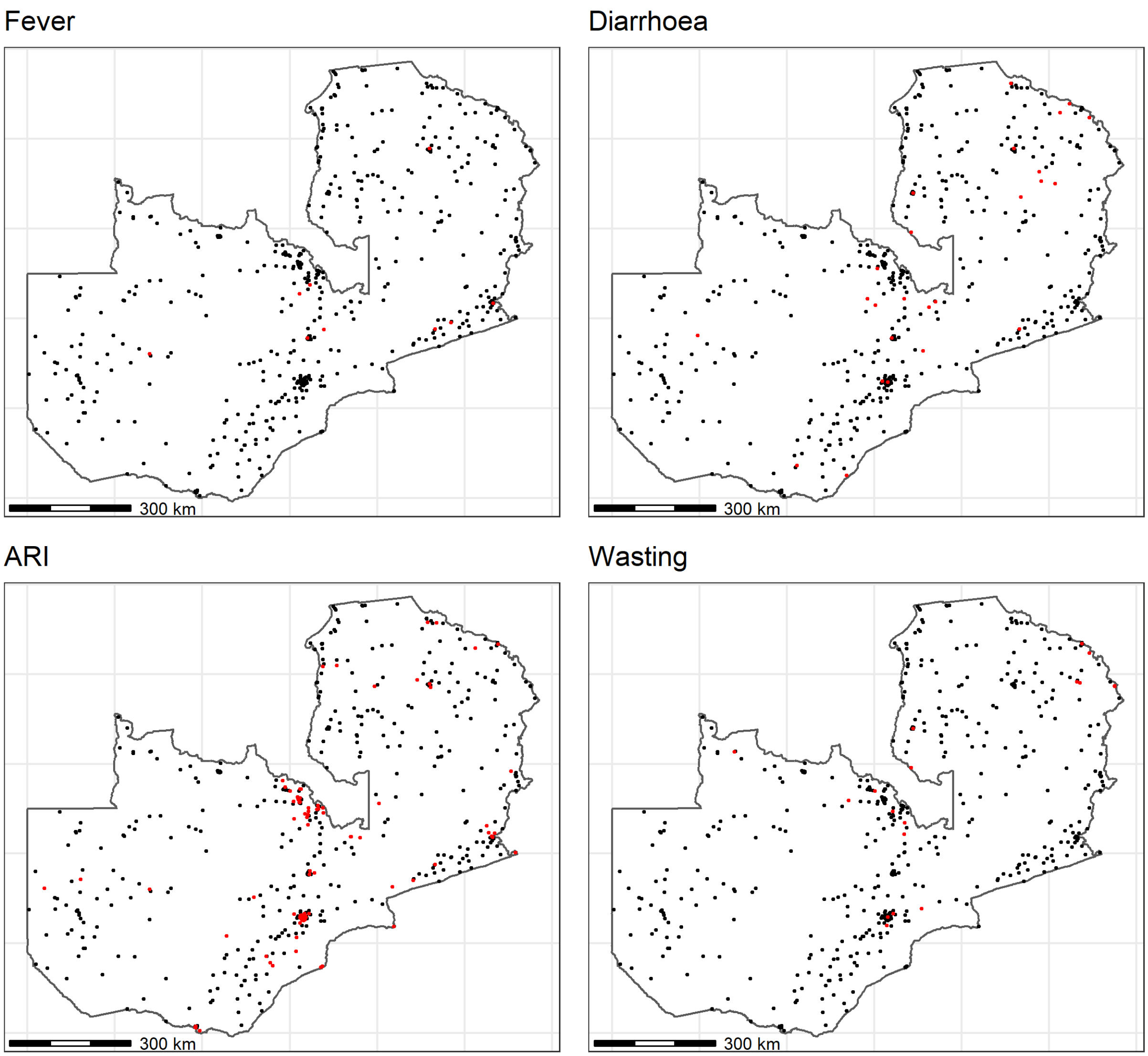

Zimbabwe

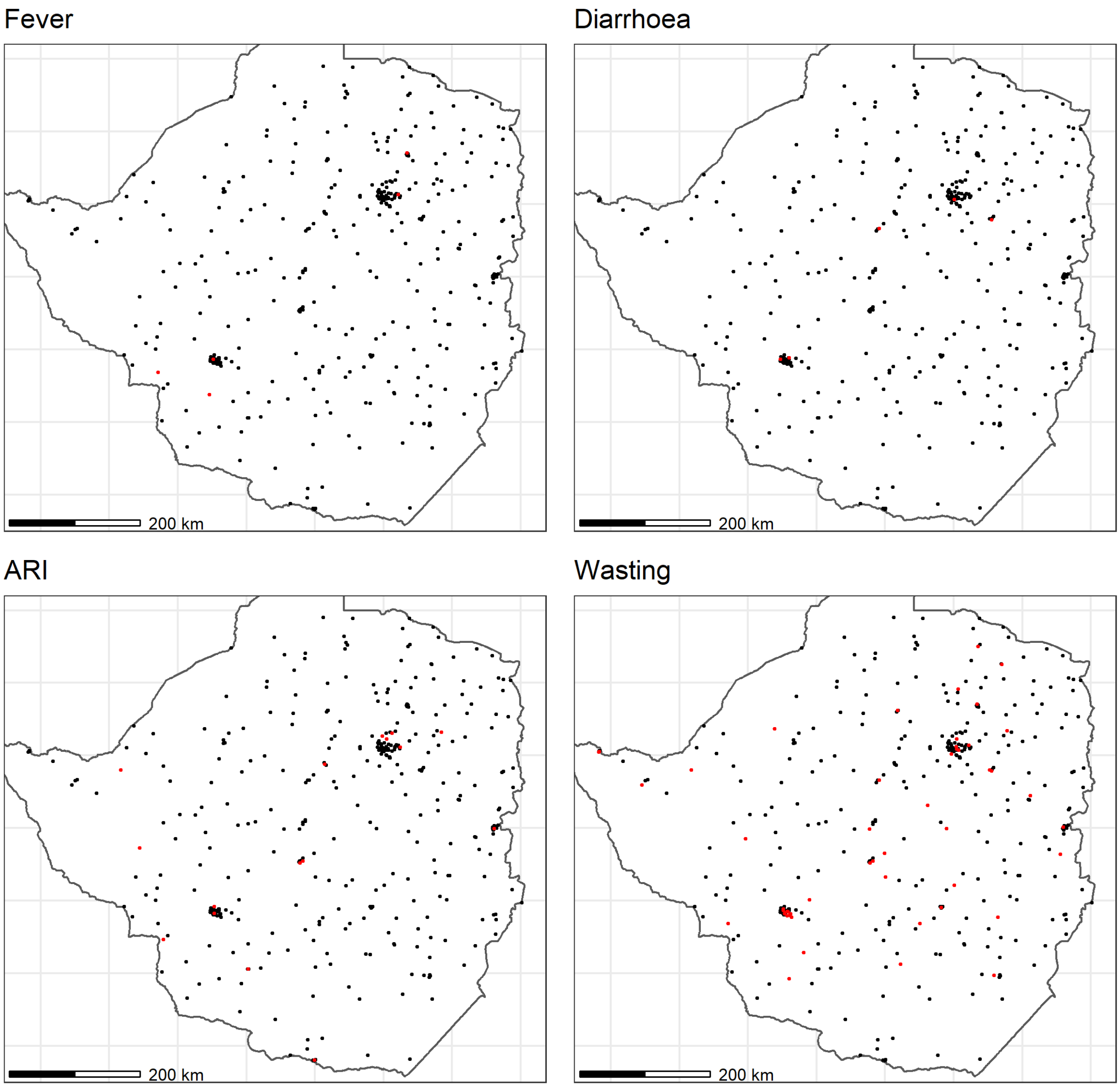

Bihar

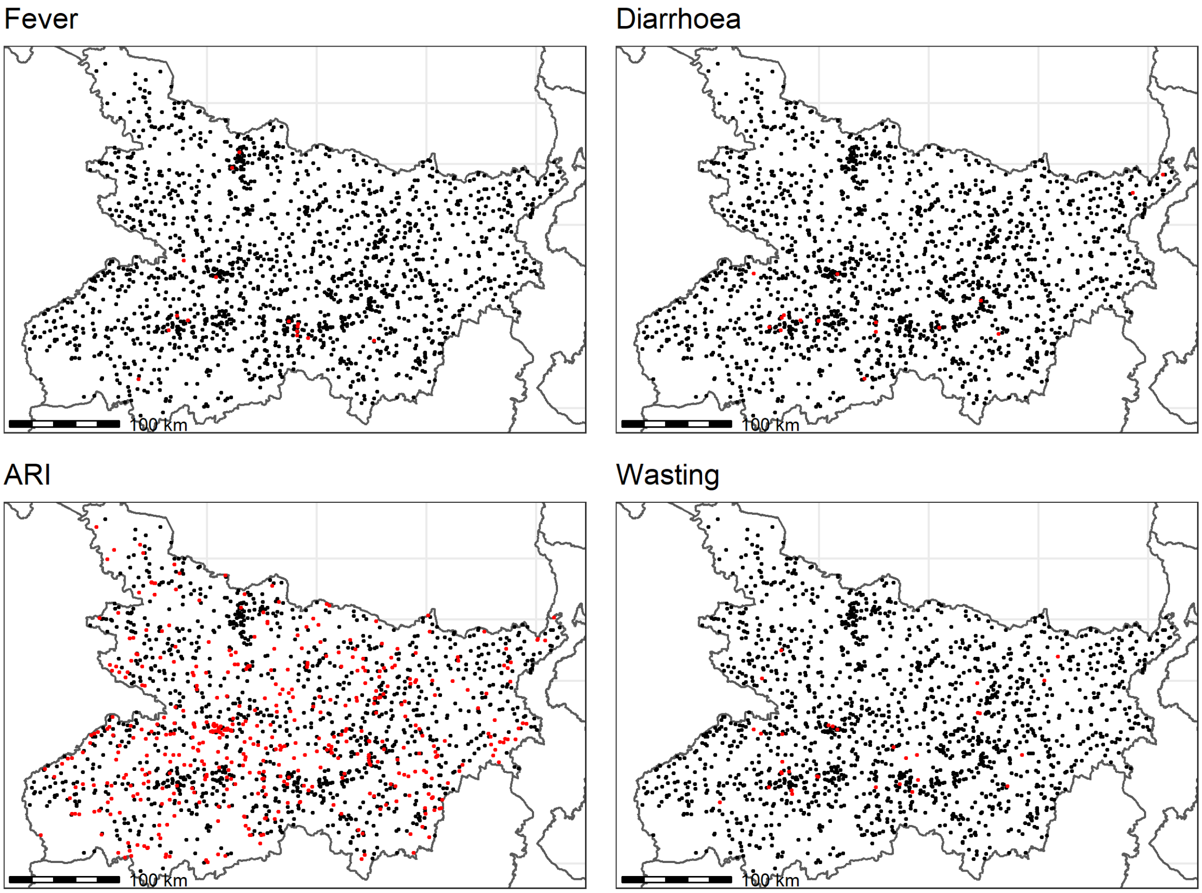

Burkina Faso

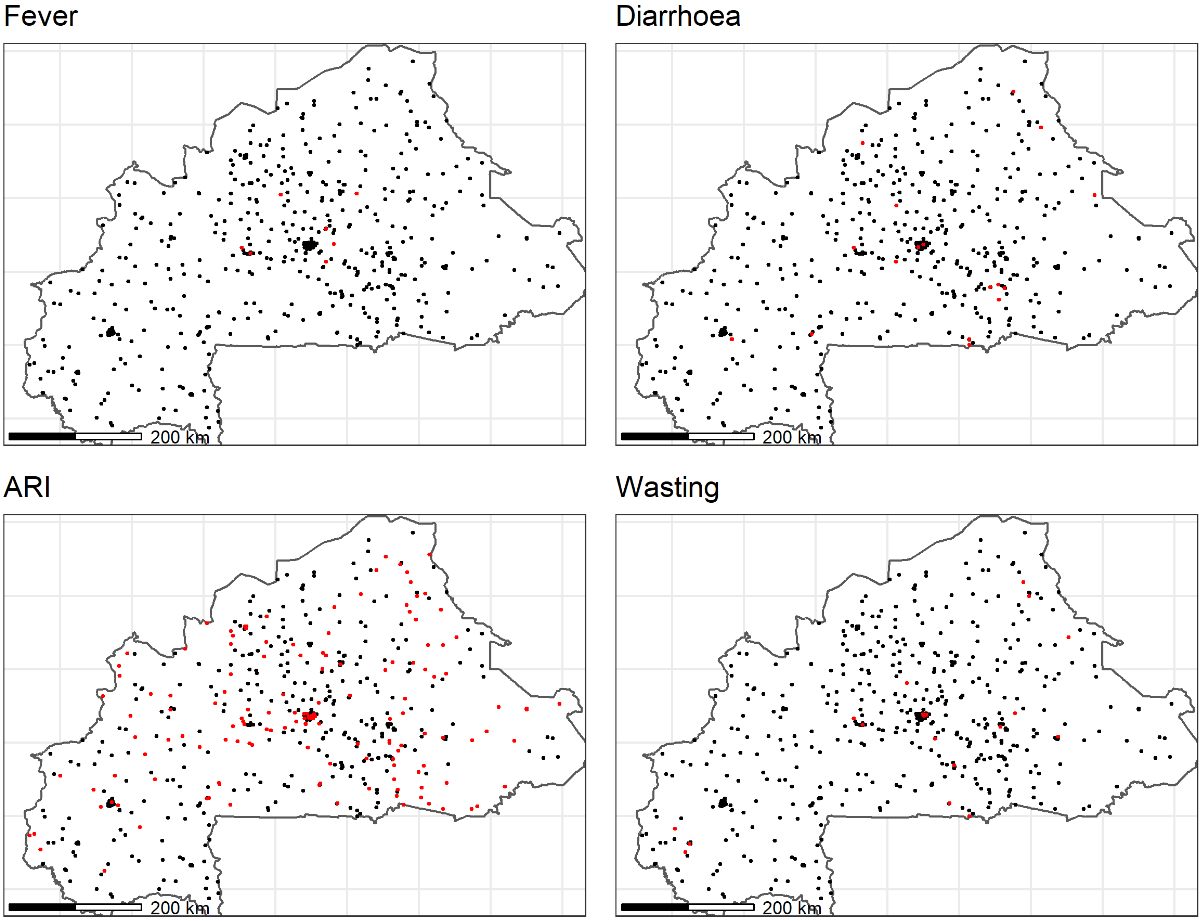

Supplement: Supplementary file 6 — Additional file 6. Model fit. Posterior predictive checks, AUC and posterior predictive spatial checks. [file 12916_2021_2018_MOESM6_ESM.pdf]
